# Atroposelective Synthesis of Axially Chiral N‐Arylpyrroles by Chiral‐at‐Rhodium Catalysis

**DOI:** 10.1002/anie.202004799

**Published:** 2020-06-03

**Authors:** Chen‐Xi Ye, Shuming Chen, Feng Han, Xiulan Xie, Sergei Ivlev, K. N. Houk, Eric Meggers

**Affiliations:** ^1^ Fachbereich Chemie Philipps-Universität Marburg Hans-Meerwein-Straße 4 35043 Marburg Germany; ^2^ University of California, Los Angeles Department of Chemistry and Biochemistry Los Angeles CA 90095-1569 USA

**Keywords:** atroposelective reactions, chiral biaryls, chiral-at-metal complexes, homogeneous catalysis, N-arylpyrroles

## Abstract

A transformation of fluxional into configurationally stable axially chiral N‐arylpyrroles was achieved with a highly atroposelective electrophilic aromatic substitution catalyzed by a chiral‐at‐metal rhodium Lewis acid. Specifically, N‐arylpyrroles were alkylated with N‐acryloyl‐1*H*‐pyrazole electrophiles in up to 93 % yield and with up to >99.5 % *ee*, and follow‐up conversions reveal the synthetic utility of this new method. DFT calculations elucidate the origins of the observed excellent atroposelectivity.

## Graphical abstract

**Freeze, don't move**: A transformation of fluxional into configurationally stable axially chiral N‐arylpyrroles was achieved through a highly atroposelective electrophilic aromatic substitution catalyzed by a chiral‐at‐metal rhodium Lewis acid, with follow‐up transformations leading to structurally diverse axially chiral N‐arylpyrroles.

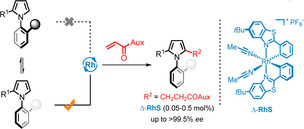

Axially chiral biaryls play a pivotal role in organic chemistry as chiral reagents and catalysts, and as structural motifs in bioactive compounds and functional materials.^1^, ^2^, ^3^, ^4^, ^5^, ^6^ Among them, axially chiral N‐arylpyrroles are increasingly popular building blocks due to their specific structural and electronic properties among biaryls, in addition to a straightforward functionalization of the electron‐rich aromatic pyrrole moiety. For example, axially chiral N‐arylpyrroles have been used as reagents for resolving racemates,^7^ as chiral coordinating ligands,^8^ and as chiral organocatalysts.^9^ Interestingly, Beller and co‐workers introduced 2‐phosphino‐1‐arylpyrroles as highly effective phosphine ligands, some of which are commercially available (e.g. cataCXium P).^10^ Their merit has been demonstrated, for example, for palladium‐catalyzed cross couplings,^10^ direct aminations of alcohols with ammonia,^11^ C(sp^3^)−H arylations,^12^ and aminocarbonylations of olefins.^13^ Although not used as non‐racemic atropisomers, these studies herald untapped opportunities for applying axially chiral N‐arylpyrroles as chiral phosphine ligands in asymmetric transition metal catalysis.

Despite their undeniable importance in organic synthesis, methods for accessing axially chiral N‐arylpyrroles are surprisingly scarce, and most methods developed to date rely on optical resolution of racemic starting materials.^7^, ^8^, ^9^ Only two catalytic asymmetric strategies have been reported, both by Tan and co‐workers. One of them is a combined Lewis acid and chiral phosphoric acid (CPA) catalyzed asymmetric Paal–Knorr reaction for constructing the pyrrole ring from anilines and 1,4‐diketones (Figure 1 a).^14^ However, the reaction suffers from high catalyst loading, sluggish rates, and a limited substrate scope. Very recently, the same group reported a CPA catalyzed desymmetrization or kinetic resolution of prochiral or racemic 2,5‐disubstituted N‐arylpyrroles to provide highly enantioenriched products (Figure 1 b).^15^ Although this is an impressive achievement, this reaction also features a limited scope since it relies on the introduction of bulky ketomalonates at the 3‐ or 4‐position of the pyrrole moiety. Furthermore, in the case of unsymmetrical 2,5‐disubstituted pyrrole substrates, the reaction represents a kinetic resolution with the drawback of converting only half of the substrate. Clearly, developing general and efficient catalytic methodologies towards axially chiral N‐arylpyrroles remains underdeveloped and highly desirable.

**Figure 1:**
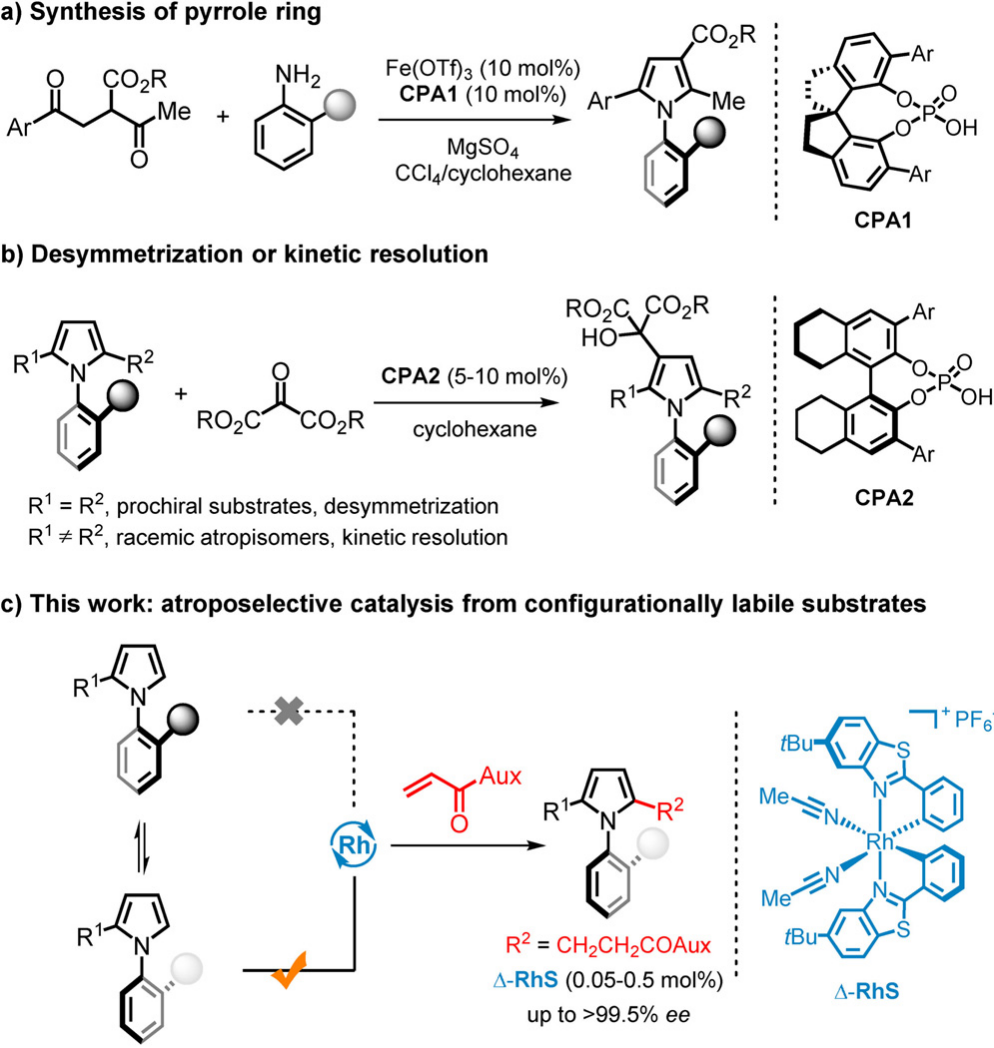
Catalytic asymmetric strategies for the synthesis of non‐racemic axially chiral N‐arylpyrroles, and the method developed in this study.

Herein, we report a highly atroposelective catalytic strategy to axially chiral N‐arylpyrroles (Figure 1 c). The method capitalizes on the unique helical chirality of the employed chiral‐at‐metal rhodium catalyst, which strongly discriminates between configurationally labile N‐arylpyrrole substrates. This method provides a general access to structurally diverse non‐racemic N‐arylpyrrole atropisomers.

Electrophilic aromatic substitution is arguably the most important reaction for functionalizing pyrrole rings. We envisioned that by introducing an electrophile into the most nucleophilic α‐position of pyrroles, the rotation around the C−N axis of N‐arylpyrroles could be modulated and configurationally stable axial chirality implemented in the course of the reaction. Using a chiral catalyst would then allow the development of a catalytic atroposelective procedure.

We commenced our study by using *N*‐(2‐isopropylphenyl)‐2‐methylpyrrole (**1 a**) as a model substrate. The compound displays axial chirality, but at room temperature, the two atropisomers interconvert rapidly. N‐Arylpyrrole **1 a** was initially reacted with *N*‐acryloyl‐1*H*‐pyrazole (**2 a**) in the presence of the bis‐cyclometalated rhodium catalyst Δ‐**RhS**. Chiral‐at‐metal bis‐cyclometalated iridium and rhodium complexes have been demonstrated to serve as very versatile chiral Lewis acid catalysts for conjugate additions to chelating enone derivatives.^16^, ^17^, ^18^ Indeed, reaction of **1 a** with **2 a** at room temperature in the presence of 1 mol % Δ‐**RhS** provided the desired product **3 aa** in 53 % yield with very encouraging 98 % *ee* (Table 1, entry 1). The major side product that diminished the reaction yield derived from multiple substitution on the pyrrole ring by **2 a** (see the Supporting Information for more details). Under the same conditions, methyl‐substituted pyrazole auxiliaries **2 b** and **2 c** slowed down the reaction significantly and gave products with lower *ee*, despite the fact that multiple substituted side products were inhibited to some extent due to their increased steric hindrance (entries 2 and 3). When the reaction temperature was lowered to 0 °C, both yield and *ee* improved at the expense of slightly elongated reaction time (entry 4 vs. 1). Next we investigated the effect of solvents and found that the reaction tolerates a broad range of solvents to give products with excellent *ee* values (entries 5 to 8). Except for acetone, similar yields were obtained (±6 %), while CH_2_Cl_2_ endowed the reaction highest rate and was thus chosen as the optimal solvent. Moreover, the study of catalyst loading revealed that 0.5 mol % Δ‐**RhS** was superior to others (entries 4, 9 to 11), while the catalyst loading could be further lowered to 0.05 mol % to allow completion of the reaction within a reasonable reaction time with negligible decrease of the yield and *ee*. In the absence of Δ‐**RhS**, no reaction occurred and both reactants remained intact, thus revealing the absence of any background reaction (entry 12).

**Table 1:** Initial experiments and optimization of the reaction conditions.^[a]^ 
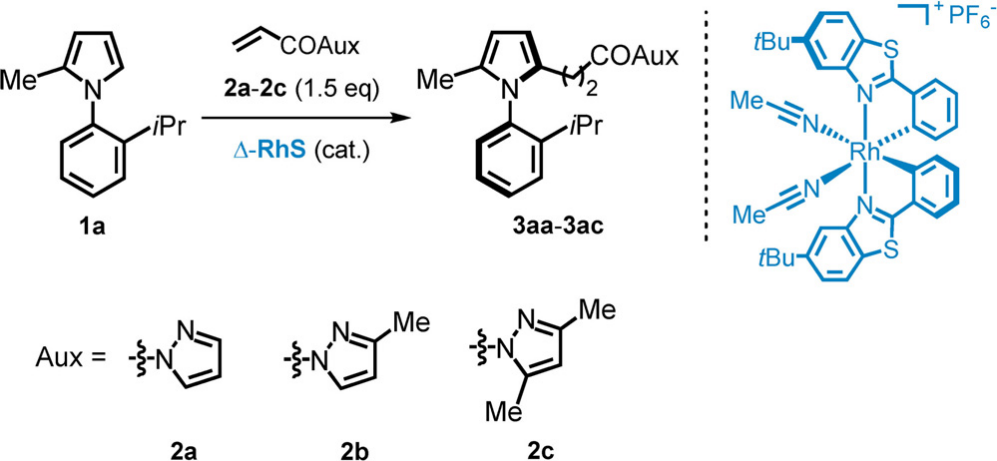

Entry	Auxiliary	Cat. [mol %]	Solvent	*T* [°C]	*t* [h]	Yield [%]^[b]^	*ee* [%]^[c]^
1	**2 a**	1	CH_2_Cl_2_	25	0.5	53	98
2	**2 b**	1	CH_2_Cl_2_	25	16	73	96
3	**2 c**	1	CH_2_Cl_2_	25	16	62	97
4	**2 a**	1	CH_2_Cl_2_	0	1	75	99
5	**2 a**	1	DCE	0	3	81	98
6	**2 a**	1	THF	0	6	70	99
7	**2 a**	1	acetone	0	2	53	99
8	**2 a**	1	toluene	0	4	80	99
9	**2 a**	0.5	CH_2_Cl_2_	0	2	93(92^[d]^)	99
10	**2 a**	0.1	CH_2_Cl_2_	0	8	90	98
11	**2 a**	0.05	CH_2_Cl_2_	0	16	90	98
12	**2 a**	none	CH_2_Cl_2_	25	24	n.d.	–

[a] General reaction conditions for optimization: a mixture of N‐arylpyrrole **1 a** (0.1 mmol), pyrazoles **2 a**–**2 c** (0.15 mmol) and Δ‐**RhS** (0.05–1 mol %) in the indicated solvent (1 mL) was stirred at the indicated temperature until full conversion of **1 a**. [b] Reaction yield was determined by ^1^H‐NMR analysis using hexamethylbenzene as an internal standard. [c] Enantiomeric excess (*ee*) was determined by HPLC on chiral stationary phases. [d] Yield of isolated product.

Having established the optimal reaction conditions, we next investigated the substrate scope and started with N‐arylpyrroles containing a variety of substituents at the pyrrole ring (Figure 2). As for 2‐substituted pyrroles, the reaction generally gave high‐yielding products (**3 aa**–**3 d**) with excellent atroposelectivity values of up to 99 % *ee*. It is noteworthy that the *ee* values tend to decrease with bulkier R^1^. When 2,3‐dialkyl pyrroles were employed, the reaction gave only moderate yields of the axially chiral N‐arylpyrroles (**3 e** and **3 f**). This can be traced back to an increased electron density on these pyrrole rings which accelerates multiple substitution side reactions to erode the yield of the desired monosubstituted product.

**Figure 2:**
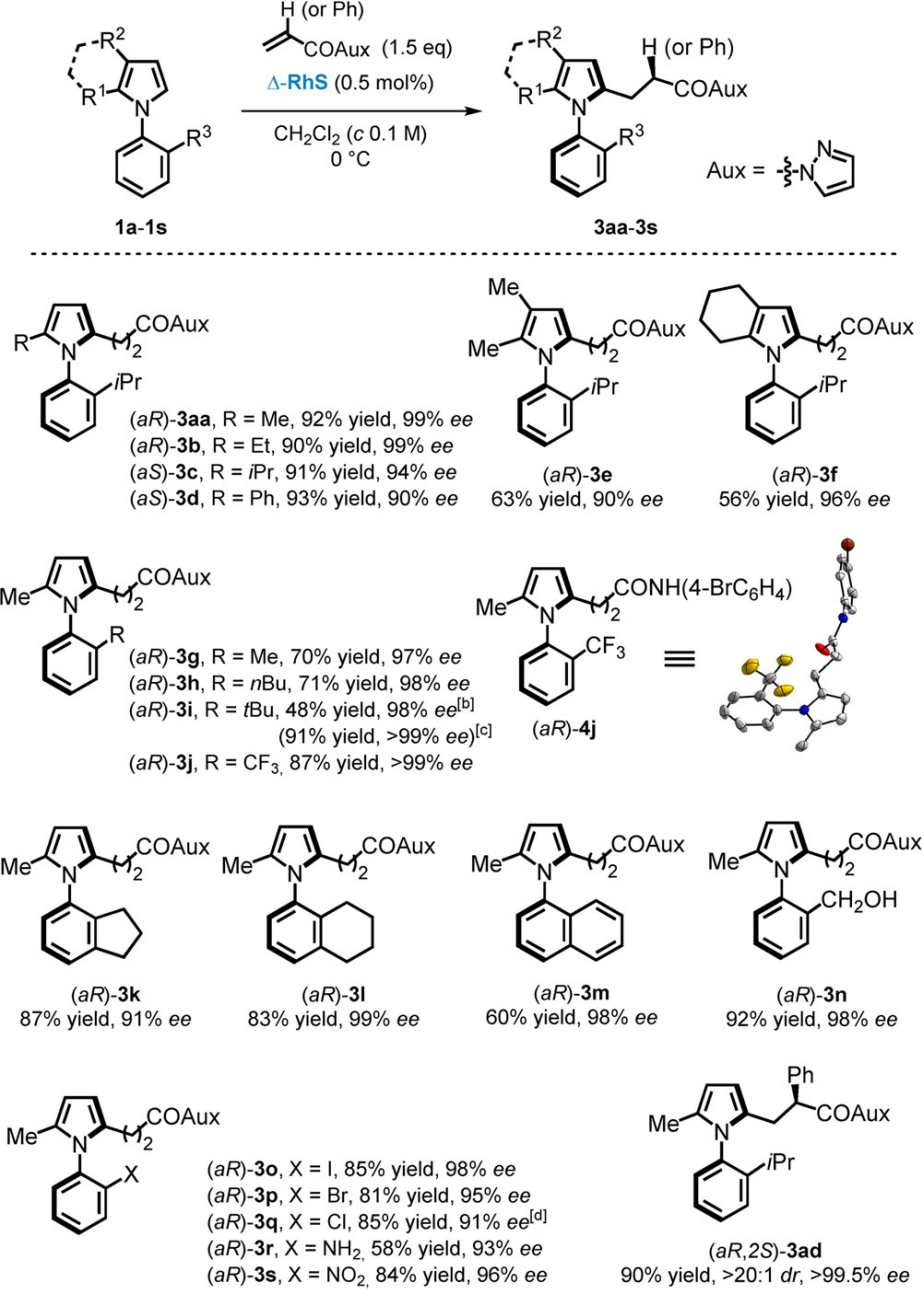
Substrate scope. [a] General reaction conditions: A mixture of N‐arylpyrrole **1** (0.1 mmol), pyrazole **2 a** or **2 d** (0.15 mmol), and Δ‐**RhS** (0.5 mol %) in CH_2_Cl_2_ (1 mL) was stirred at 0 °C until full conversion of **1**. [b] Under general reaction conditions, kinetic resolution of **1 i** has a selectivity factor of *S*=458, calculated as *S*=ln[(1−*C*)(1−*ee*(**1 i**))]/ln[(1−*C*)(1+*ee*(**1 i**))], in which *C*=*ee*(**1 i**)/(*ee*(**1 i**)+*ee*(**3 i**)). [c] Modified reaction conditions: a mixture of N‐arylpyrrole **1 i** (0.25 mmol), pyrazole **2 a** (0.1 mmol), and Δ‐**RhS** (0.5 mol %) in CH_2_Cl_2_ (1 mL) was stirred at 0 °C until full conversion of **2 a**. Reaction yield was calculated according to the amount of **2 a**. [d] Reaction temperature was −20 °C.

We also investigated the substituent effect on the phenyl ring through which axially chiral N‐arylpyrroles with different R^3^ (**3 g**–**3 s**) were obtained in high yields and with excellent atroposelectivity of up to >99 % *ee*. It is worth mentioning that under general reaction conditions, *N*‐(2‐*tert*‐butylphenyl)‐2‐methylpyrrole (**1 i**) exhibits stable atropisomerism due to the very bulky *tert*‐butyl substituent, and thus the reaction proceeded in a manner of kinetic resolution by converting half of the **1 i** with a selectivity factor of *S*=458 (see the Supporting Information for details). However, high yield and a high *ee* value for the alkylated N‐arylpyrrole **3 i** were obtained by using an excess amount (2.5 equivalents) of *N*‐(2‐*tert*‐butylphenyl)pyrrole substrate (modified reaction conditions). Polycyclic substrates gave products featuring N‐indanyl (**3 k**), N‐(tetrahydro)naphthyl (**3 l**), and N‐(1‐naphthyl) (**3 m**) pyrrole scaffolds in high yields and with excellent *ee* values. Halogen‐containing substrates reacted smoothly to give products **3 o**–**3 q**, which are excellent coupling partners in transition‐metal‐catalyzed reactions, thereby allowing a potential late‐stage introduction of various R^3^ substituents. For the less bulky chlorine, the reaction was performed at lower temperature for obtaining higher than 90 % *ee*. This reaction is also compatible with unprotected aniline to provide the corresponding product **3 r** as a candidate for axially chiral organocatalysts. Substrates that contain a sensitive free hydroxy group (product **3 n**) and a strong deactivating nitro group (product **3 s**) were well tolerated under standard conditions. An interesting result was obtained when 2‐phenyl‐1‐(1*H*‐pyrazol‐1‐yl)‐propenone was employed as the reaction partner, for which product **3 ad** was obtained with very high stereoselectivity of >20:1 *dr* and >99.5 % *ee*. This represents a special example for an atroposelective reaction in which axial and point chirality are simultaneously constructed within a single‐step transformation. We also investigated the configurational stability of products **3 g**, **3 q**, and **3 r**, which have less bulky R^3^ substituents. Heating in toluene at 80 °C for 18 hours did not affect the enantiomeric excess, thus indicating that the rotation energies around the chiral axis of N‐arylpyrroles after electrophilic aromatic substitution are sufficiently high (see the Supporting Information for details).

For determining the absolute configuration of the products, CF_3_‐containing **3 j** was converted into crystallizable secondary amide **4 j**. Single‐crystal XRD analysis of **4 j** attributed an *aR* configuration to its chiral axis linkage and the configurations of other products were assigned accordingly. Furthermore, the relative stereochemistry of **3 ad** was determined as *aR*,*2S* based on 2D NOESY experiments (see the Supporting Information for details).

Density functional theory (DFT) calculations were performed to understand the origins of stereoselectivity of this atroposelective reaction (Figure 3). N‐Arylpyrrole substrates exist as transient and interconvertible atropisomers that racemize rapidly under standard condition. While Δ‐**RhS** activated acrylpyrazole can react with both atropisomers, the calculated free energy barrier is 2.1 kcal mol^−1^ lower for the reaction with (*aS*)‐N‐arylpyrrole (**TS‐1 a**) than with (*aR*)‐N‐arylpyrrole (**TS‐1 b**). In **TS‐1 b**, a steric clash (closest H⋅⋅⋅H distance 2.35 Å) exists between the isopropyl substituent on the N‐arylpyrrole and the *tert*‐butyl group on the ligand, a destabilizing interaction absent that is in **TS‐1 a**. Assuming that *k*_rac_≫*k*_Δ*R*_ (the rate constants of racemization and reactions of Δ‐**RhS** activated acrylpyrazole with (*aS*)‐N‐arylpyrrole or (*aR*)‐N‐arylpyrrole were denoted as *k*_rac_, *k*_Δ*S*_ and *k*_Δ*R*_, respectively), the reaction can convert all substrate into a single atropisomer.

**Figure 3:**
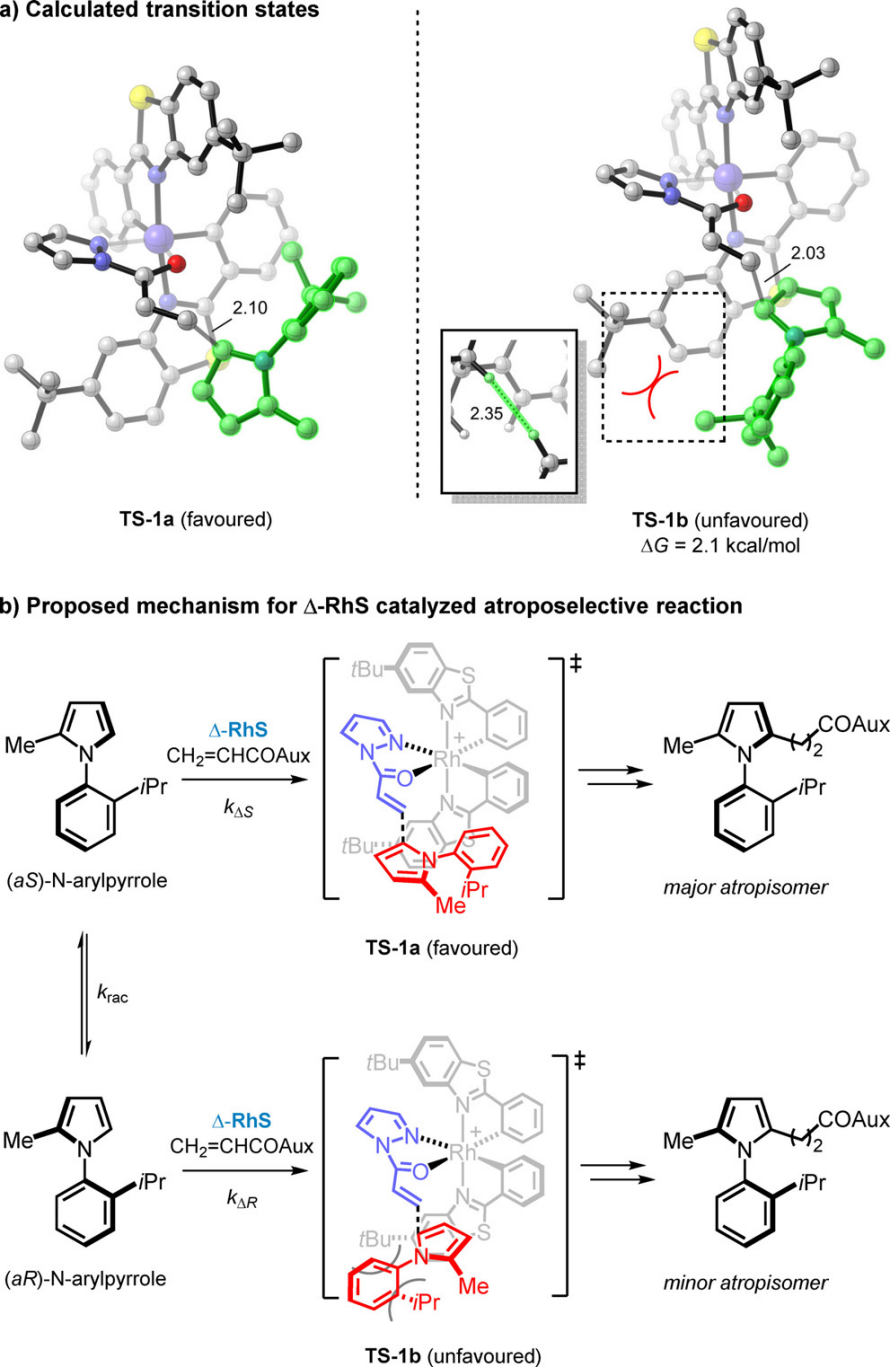
Transition states and proposed mechanism for the reaction between **1 a** and **2 a** catalyzed by Δ‐**RhS** calculated at the M06‐D3/6–311++G(d,p)‐SDD(Rh), SMD(CH_2_Cl_2_)//B3LYP‐D3BJ/6‐31G(d)‐LANL2DZ(Rh) level of theory. Hydrogen atoms are omitted for clarity. Interatomic distances are in ångströms.

To demonstrate the synthetic value of our method, a gram‐scale synthesis of **1 o**→**3 o** was tested under our standard conditions, which provided the desired product **3 o** in 88 % yield of isolated product with 98 % *ee*. Furthermore, we conducted a thorough study on follow‐up chemistry by employing **3 o** as a model product (Figure 4). N‐Acylpyrazoles are highly inclined to nucleophilic substitutions. For instance, reacting **3 o** with aniline resulted in a quantitative formation of the secondary amide **4 o**, and the configuration of the chiral axis remained stable in refluxing toluene for 16 hours. In the presence of catalytic amounts of DBU, the N‐acylpyrazole moiety could be smoothly converted into the ester **5 o** at room temperature. Following a sequential hydrolysis and Friedel–Crafts acylation, product **6 o** (note the rearrangement), which is equipped with a versatile ketone moiety near the chiral axis, was obtained.^19^, ^20^ Furthermore, the N‐acylpyrazole could also be reduced to different states by utilizing appropriate reductants. As examples, NaBH_4_‐mediated reduction of **3 o** resulted in the formation of the primary alcohol **7 o**, which could be converted into phosphino‐substituted N‐arylpyrrole **8 o** under palladium‐catalyzed conditions, while selective reduction towards an aldehyde required much lower reaction temperature using DIBAL‐H as the reducing agent to provide aldehyde **9 o**. Aldehyde **9 o** then served as an ideal substrate for a dehydroformylation to produce the vinylpyrrole **10 o**, following a literature procedure.^21^

**Figure 4:**
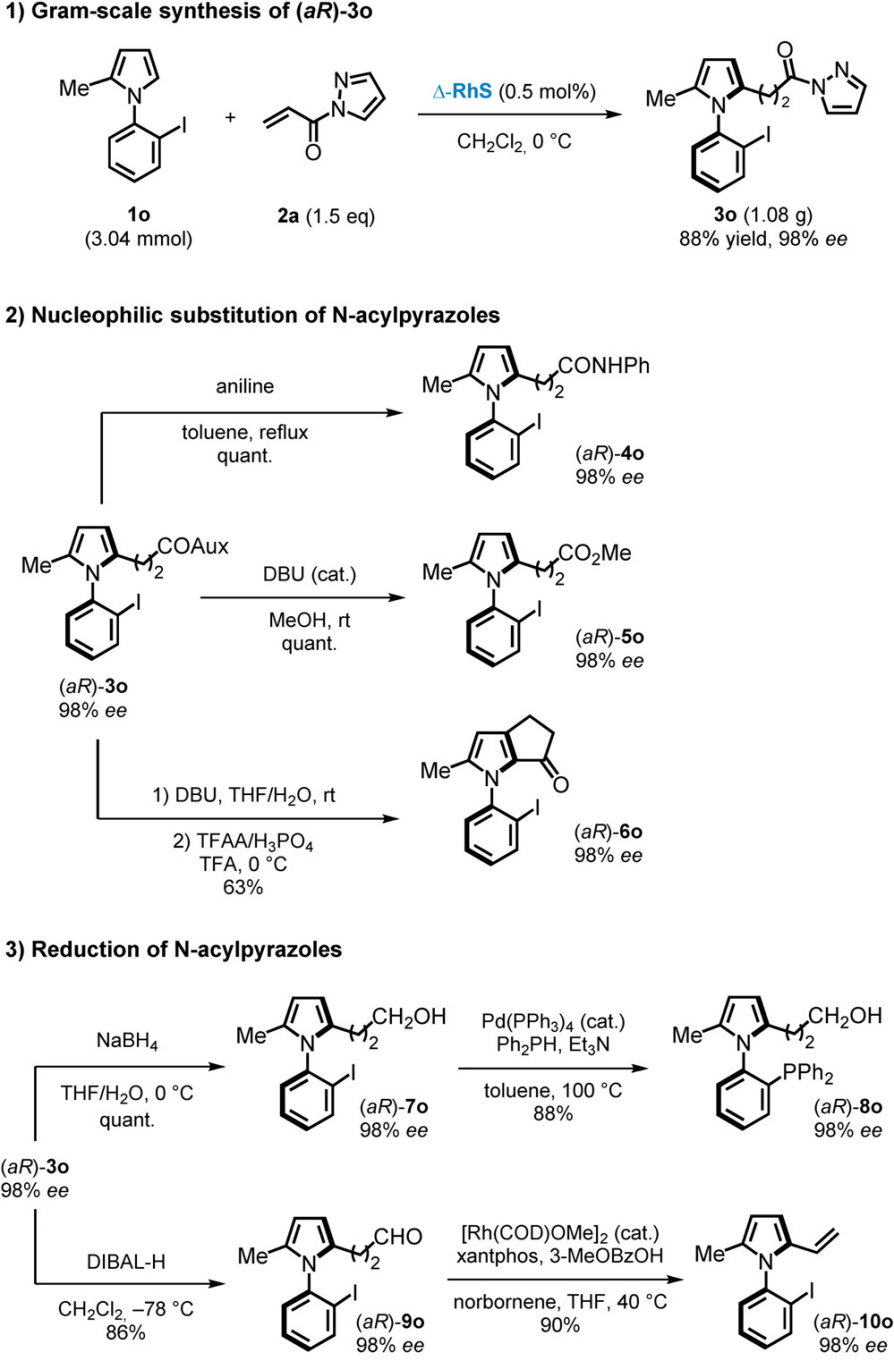
Gram‐scale synthesis and synthetic elaborations of axially chiral N‐arylpyrrole product **3 o**.

In conclusion, we have developed a method to access axially chiral N‐arylpyrroles in high yields, with excellent stereoselectivity, and with a broad substrate scope. The method relies on the highly atroposelective alkylation of configurationally fluxional chiral N‐arylpyrroles with *N*‐acryloyl‐1*H*‐pyrazole as the electrophile catalyzed by a chiral‐at‐metal rhodium catalyst. The alkylated products are significant building blocks towards structurally diverse N‐arylpyrroles by versatile transformations of acylpyrazole moiety. Furthermore, DFT calculations were used to characterize the reaction transition states and to reveal the origins of the stereoselectivity, which establishes foundations for future studies of related reactions.

## Conflict of interest

The authors declare no conflict of interest.

## Supporting information

As a service to our authors and readers, this journal provides supporting information supplied by the authors. Such materials are peer reviewed and may be re‐organized for online delivery, but are not copy‐edited or typeset. Technical support issues arising from supporting information (other than missing files) should be addressed to the authors.

Supplementary
