# Supplementary material for: Atroposelective Synthesis of Axially Chiral N‐Arylpyrroles by Chiral‐at‐Rhodium Catalysis
Source: Angew Chem Int Ed Engl. 2020 Jun 3;59(32):13552–6. doi: 10.1002/anie.202004799 (PMC7496906; doi:10.1002/anie.202004799)
Supplement: Supplementary file 1 — Supplementary [file ANIE-59-13552-s001.pdf]

Angewandte Chemie
Eine Zeitschrift der Gesellschaft Deutscher Chemiker

Supporting Information

Atroposelective Synthesis of Axially Chiral N-Arylpyrroles by Chiral-at-Rhodium Catalysis

Chen-Xi Ye, Shuming Chen, Feng Han, Xiulan Xie, Sergei Ivlev, K. N. Houk, and Eric Meggers**

anie_202004799_sm_miscellaneous_information.pdf

Table of Contents

1. General Information ········ S-1
2. General Procedure for Synthesizing *N*-Arylpyrrole Substrates ········ S-1
3. Synthesis of Acrylpyrazoles ········ S-9
4. Initial Conditions and Optimization of Pyrazole Auxiliaries ········ S-10
5. General Procedure for Δ-RhS Catalyzed Reactions ········ S-12
6. Determination of the Selectivity Factor for Kinetic Resolution of 1i ········ S-21
7. Determination of the Absolute Configuration of a Derivative of *N*-Arylpyrrole 3j ········ S-24
8. Determination of the Relative Configuration of *N*-Arylpyrrole 3ad ········ S-25
9. Configurational Stability Investigations of 3g, 3q and 3r ········ S-28
10. Gram-Scale Synthesis and Derivations of *N*-Arylpyrrole 3o ········ S-29
11. Computational Studies of the Transition States ········ S-34
12. Single-Crystal X-Ray Diffraction Analysis of 4j ········ S-58
13. Reference ········ S-75
14. NMR and HPLC Spectra ········ S-78

1. General Information

Catalytic reactions were performed in Schlenk tubes (10 mL) under air with magnetic stirring. Δ-**RhS** was prepared according to a literature procedure.[1] Chemicals were used as received from commercial suppliers unless stated otherwise. Solvents were distilled under nitrogen from calcium hydride (for CH_3CN and CH_2Cl_2) or sodium/benzophenone (for THF). Flash column chromatography was performed with silica gel 60 M from Macherey-Nagel (230-400 mesh). Infrared (IR) spectra were measured on a Bruker Alpha FT-IR spectrometer. ^{1}H and ^{13}C NMR spectra were recorded on a Bruker Advance 300 MHz or 250 MHz spectrometer at ambient temperature. Chemical shifts are expressed in parts per million (δ) referenced to chloroform (7.26 ppm or 77.0 ppm). The NMR data are presented as follows: chemical shift, multiplicity (s = singlet, d = doublet, t = triplet, q = quartet, quint = quintet, hept = heptet, dd = doublet of doublet, dt = doublet of triplet, td = triplet of doublet, ddd = doublet of doublet of doublet, m = multiplet, br = broad), coupling constant (Hz) and integration. High resolution mass spectra (HRMS) were recorded on a Bruker En Apex Ultra 7.0 T FT-MS mass spectrometer. Optical rotations were measured using a Perkin-Elmer 241 polarimeter with $[\alpha]_D^{25}$ values reported in degrees with concentrations reported in g/100 mL. Enantiomeric excesses (*ee*) were determined by HPLC analysis using an Agilent HPLC 1260 with chiral stationary phases.

2. General Procedure for Synthesizing *N*-Arylpyrrole Substrates

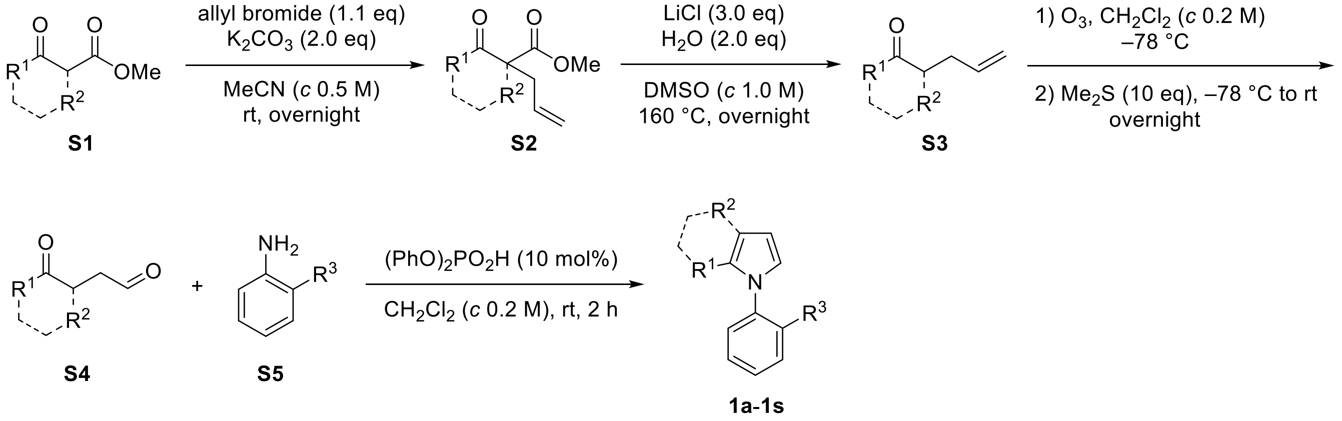

N-arylpyrroles were prepared by Paal-Knorr reaction of 4-oxoaldehydes **S4** with anilines **S5** in the presence of catalytic amount of diphenylphosphinic acid. **S4** were synthesized from commercially available β-ketoesters **S1** through a sequential allylation, dealkoxycarbonylation and reductive ozonolysis. The synthetic procedures are shown below.

To a 250 mL round bottom flask containing K_2CO_3 (5.53 g, 40 mmol, 2.0 eq) and CH_3CN (40 mL, *c* 0.5 M) was added β-ketoester **S1** (20 mmol, 1.0 eq) and allyl bromide (1.9 mL, 22 mmol, 1.1 eq). The reaction mixture was stirred at room temperature for overnight. To quench the reaction, hydrochloric acid (10%) was added to the flask slowly until the pH reached 6-7. The reaction mixture was further diluted with 50 mL water and was extracted with EtOAc (3×50 mL). The combined organic layer was washed with brine and dried over anhydrous sodium sulfate. After filtration, the solvent was evaporated under reduced pressure, and the residue was purified by column chromatography (silica gel, eluted by a mixture of EtOAc and *n*-hexane) to afford compound **S2**.

To a 50 mL round bottom flask equipped with a reflux condenser was added **S2** (10 mmol, 1.0 eq) and DMSO (10 mL, *c* 1.0 M), followed by addition of lithium chloride (1.27 g, 30 mmol, 3.0 eq) and water (0.36 mL, 20 mmol, 2.0 eq). The reaction mixture was stirred at 160 °C for overnight using an oil bath. After being cooled to room temperature, the mixture was diluted with 50 mL water and was extracted with Et_2O (5×50 mL). The combined organic layer was washed with brine and dried over anhydrous sodium sulfate. After filtration, the solvent was evaporated under reduced pressure, and the residue was purified by column chromatography (silica gel, eluted by a mixture of Et_2O and *n*-pentane) to afford compound **S3** as a volatile liquid.

A solution of **S3** (10 mmol, 1.0 eq) in CH_2Cl_2 (50 mL, *c* 0.2 M) in a 250 mL round bottom flask was cooled to −78 °C by using a dry ice bath. A stream of O_3/O_2 was bubbled into the flask until the solution became pale blue. Excess ozone was removed by bubbling O_2 for 5 min and then bubbling N_2 for another 5 min. Me_2S (7.3 mL, 100 mmol, 10 eq) was added to the flask before removal of the dry ice bath. The mixture was then warmed to room temperature and stirred for overnight. After completion, the solvent was evaporated under reduced pressure, and the residue was purified by column chromatography (silica gel, eluted by a mixture of Et_2O and *n*-pentane) to afford compound **S4** as a volatile liquid.

To a solution of 2-substituted aniline **S5** (5 mmol, 1.0 eq) in CH_2Cl_2 (25 mL, *c* 0.2 M) in a 100 mL round bottom flask was added **S4** (6 mmol, 1.2 eq) and $(PhO)_2PO_2H$ (125 mg, 0.5 mmol, 10 mol%). The mixture was stirred at room temperature until full conversion of aniline, which was monitored by TLC analysis. Then the solvent was evaporated under reduced pressure, and the residue was purified by column chromatography (silica gel, eluted by a mixture of EtOAc and *n*-hexane) to afford the desired *N*-arylpyrroles.

1-(2-Isopropylphenyl)-2-methyl-1*H*-pyrrole (1a)

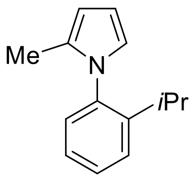

Following the **general procedure**, **1a** was obtained as a colorless oil (chromatography on silica gel, eluent: EtOAc/*n*-hexane = 1/60): IR (film) ν_{max}: 2963, 1496, 756, 701 cm^{-1}; ^{1}H NMR (300 MHz, $CDCl_3$) δ 1.14 (d, *J* = 6.9 Hz, 3H), 1.15 (d, *J* = 6.9 Hz, 3H), 2.00 (s, 3H), 2.59 (hept, *J* = 6.9 Hz, 1H), 5.98−6.07 (m, 1H), 6.20 (t, *J* = 3.0 Hz, 1H), 6.57−6.68 (m, 1H), 7.14−7.21 (m, 1H), 7.21−7.30 (m, 1H), 7.37−7.45 (m, 2H); ^{13}C NMR (75 MHz, $CDCl_3$) δ 12.2, 23.2, 24.8, 27.5, 106.4, 107.3, 121.9, 126.1, 126.4, 128.4, 128.7, 129.9, 137.9, 146.9; HRMS calcd for $[C_{14}H_{18}N]^+$ $(M + H)^+$: 200.1434; found: 200.1439.

2-Ethyl-1-(2-isopropylphenyl)-1*H*-pyrrole (1b)

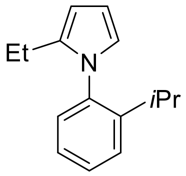

Following the **general procedure**, **1b** was obtained as a colorless oil (chromatography on silica gel, eluent: EtOAc/*n*-hexane = 1/60): IR (film) ν_{max}: 2964, 1495, 756, 703 cm^{-1}; ^{1}H NMR (300 MHz, $CDCl_3$) δ 1.127 (t, *J* = 7.5 Hz, 3H), 1.132 (d, *J* = 6.9 Hz, 3H), 1.14 (d, *J* = 6.9 Hz, 3H), 2.19−2.41 (m, 2H), 2.54 (hept, *J* = 6.9 Hz, 1H), 6.01−6.10 (m, 1H), 6.23 (t, *J* = 3.1 Hz, 1H), 6.57−6.66 (m, 1H), 7.15−7.21 (m, 1H), 7.21−7.29 (m, 1H), 7.36−7.46 (m, 2H); ^{13}C NMR (75 MHz, $CDCl_3$) δ 13.3, 19.7, 23.1, 25.0, 27.5, 104.5, 107.2, 121.9, 126.0, 126.4, 128.5, 128.7, 136.4, 137.9, 147.0; HRMS calcd for $[C_{15}H_{20}N]^+$ $(M + H)^+$: 214.1590; found: 214.1596.

2-Isopropyl-1-(2-isopropylphenyl)-1*H*-pyrrole (1c)

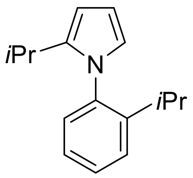

Following the **general procedure**, **1c** was obtained as a colorless oil (chromatography on silica gel, eluent: EtOAc/*n*-hexane = 1/70): IR (film) ν_{max}: 2963, 1496, 756, 700 cm^{-1}; ^{1}H NMR (300 MHz, $CDCl_3$) δ 1.11 (d, *J* = 6.9 Hz, 3H), 1.12 (d, *J* = 6.9 Hz, 3H), 1.13 (d, *J* = 6.9 Hz, 3H), 1.16 (d, *J* = 6.9 Hz, 3H), 2.50 (hept, *J* = 6.9 Hz, 1H), 2.56 (hept, *J* = 6.9 Hz, 1H), 6.03−6.10 (m, 1H), 6.23 (t, *J* = 3.1 Hz, 1H), 6.53−6.60 (m, 1H), 7.18−7.23 (m, 1H), 7.23−7.30 (m, 1H), 7.36−7.45 (m, 2H); ^{13}C NMR (75 MHz, $CDCl_3$) δ 22.8, 23.0, 24.1, 25.3, 25.5, 27.4, 102.8, 107.2, 121.7, 125.9, 126.4, 128.5, 128.7, 137.9, 141.5, 147.0; HRMS calcd for $[C_{16}H_{22}N]^+$ $(M + H)^+$: 228.1747; found: 228.1753.

1-(2-Isopropylphenyl)-2-phenyl-1*H*-pyrrole (1d)

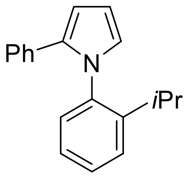

Following the **general procedure**, **1d** was obtained as a white solid (chromatography on silica gel, eluent: EtOAc/*n*-hexane = 1/60): IR (film) ν_{max}: 2975, 1490, 724, 698

cm^{-1}; ^{1}H NMR (300 MHz, CDCl$_3$) δ 0.74 (d, *J* = 6.9 Hz, 3H), 1.06 (d, *J* = 6.9 Hz, 3H), 2.61 (hept, *J* = 6.9 Hz, 1H), 6.36 (dd, *J* = 3.5, 2.8 Hz, 1H), 6.49 (dd, *J* = 3.5, 1.9 Hz, 1H), 6.78 (dd, *J* = 2.8, 1.9 Hz, 1H), 7.04–7.18 (m, 5H), 7.18–7.40 (m, 4H); ^{13}C NMR (75 MHz, CDCl$_3$) δ 22.3, 25.0, 27.5, 108.57, 108.61, 125.1, 126.0, 126.1, 126.6, 127.5, 128.0, 128.4, 128.6, 133.0, 135.1, 138.5, 146.2; HRMS calcd for [$C_{19}H_{20}N$]$^+$ (M + H)$^+$: 262.1590; found: 262.1597.

1-(2-Isopropylphenyl)-2,3-dimethyl-1*H*-pyrrole (1e)

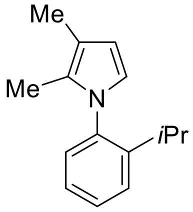

Following the **general procedure**, **1e** was obtained as a colorless oil (chromatography on silica gel, eluent: EtOAc/*n*-hexane = 1/100): IR (film) ν_{max}: 2962, 2922, 1496, 1355, 770, 639 cm^{-1}; ^{1}H NMR (300 MHz, CDCl$_3$) δ 1.13 (d, *J* = 6.9 Hz, 3H), 1.14 (d, *J* = 6.9 Hz, 3H), 1.90 (s, 3H), 2.11 (s, 3H), 2.60 (hept, *J* = 6.9 Hz, 1H), 6.07 (d, *J* = 2.5 Hz, 1H), 6.54 (d, *J* = 2.5 Hz, 1H), 7.10–7.18 (m, 1H), 7.19–7.27 (m, 1H), 7.35–7.45 (m, 2H); ^{13}C NMR (75 MHz, CDCl$_3$) δ 9.9, 11.5, 23.2, 24.8, 27.5, 108.9, 114.5, 120.4, 126.0, 126.2, 126.3, 128.5 (2C), 138.3, 147.0; HRMS calcd for [$C_{15}H_{20}N$]$^+$ (M + H)$^+$: 214.1590; found: 214.1596.

1-(2-Isopropylphenyl)-4,5,6,7-tetrahydro-1*H*-indole (1f)

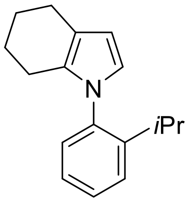

Following the **general procedure**, **1f** was obtained as a colorless oil (chromatography on silica gel, eluent: EtOAc/*n*-hexane = 1/80): IR (film) ν_{max}: 2963, 2929, 1496, 757 cm^{-1}; ^{1}H NMR (300 MHz, CDCl$_3$) δ 1.13 (d, *J* = 6.9 Hz, 3H), 1.14 (d, *J* = 6.9 Hz, 3H), 1.69–1.83 (m, 4H), 2.06–2.21 (m, 1H), 2.25–2.41 (m, 1H), 2.54–2.64 (m, 2H), 2.66 (hept, *J* = 6.9 Hz, 1H), 6.07 (d, *J* = 2.6 Hz, 1H), 6.57 (d, *J* = 2.6 Hz, 1H), 7.12–7.19 (m, 1H), 7.19–7.26 (m, 1H), 7.32–7.45 (m, 2H); ^{13}C NMR (75 MHz, CDCl$_3$) δ 22.3, 23.2, 23.3, 23.4, 23.7, 24.8, 27.5, 106.7, 117.1, 120.8, 126.0, 126.3, 128.37, 128.44, 129.4, 137.8, 146.8; HRMS calcd for [$C_{17}H_{22}N$]$^+$ (M + H)$^+$: 240.1747; found: 240.1754.

2-Methyl-1-(*o*-tolyl)-1*H*-pyrrole (1g)

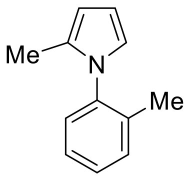

Following the **general procedure**, **1g** was obtained as a colorless oil (chromatography on silica gel, eluent: EtOAc/*n*-hexane = 1/40): IR (film) ν_{max}: 2916, 1498, 765, 699 cm^{-1}; ^{1}H NMR (300 MHz, CDCl$_3$) δ 2.00 (s, 3H), 2.04 (s, 3H), 6.00–6.06 (m, 1H), 6.20 (t, *J* = 3.1 Hz, 1H), 6.60 (dd, *J* = 2.6, 1.9 Hz, 1H), 7.18–7.23 (m, 1H), 7.23–7.36 (m, 3H); ^{13}C NMR (75 MHz, CDCl$_3$) δ 12.0, 17.2, 106.6, 107.5, 121.0, 126.4, 128.1, 128.2, 129.5, 130.6, 136.2, 139.5; HRMS calcd for [$C_{12}H_{14}N$]$^+$ (M + H)$^+$: 172.1121; found: 172.1125.

1-(2-*n*-Butylphenyl)-2-methyl-1*H*-pyrrole (1h)

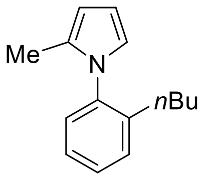

Following the **general procedure**, **1h** was obtained as a colorless oil (chromatography on silica gel, eluent: EtOAc/*n*-hexane = 1/100): IR (film) ν_{max}: 2956, 2928, 1497, 766, 701 cm^{-1}; ^1H NMR (300 MHz, $CDCl_3$) δ 0.83 (t, *J* = 7.3 Hz, 3H), 1.15−1.31 (m, 2H), 1.37−1.50 (m, 2H), 1.99 (s, 3H), 2.20−2.33 (m, 1H), 2.33−2.47 (m, 1H), 5.98−6.05 (m, 1H), 6.18 (t, *J* = 3.1 Hz, 1H), 6.57−6.65 (m, 1H), 7.15−7.21 (m, 1H), 7.21−7.29 (m, 1H), 7.30−7.39 (m, 2H); ^{13}C NMR (75 MHz, $CDCl_3$) δ 12.2, 13.8, 22.5, 30.4, 32.5, 106.5, 107.3, 121.6, 126.2, 128.2, 128.4, 129.6, 129.7, 139.0, 140.8; HRMS calcd for $[C_{15}H_{20}N]^+$ $(M + H)^+$: 214.1590; found: 214.1596.

1-(2-(*tert*-Butyl)phenyl)-2-methyl-1*H*-pyrrole (1i)

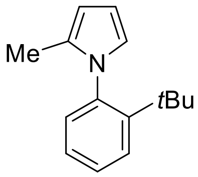

Following the **general procedure**, **1i** was obtained as a colorless oil (chromatography on silica gel, eluent: EtOAc/*n*-hexane = 1/100): IR (film) ν_{max}: 2962, 1492, 1443, 766, 757 cm^{-1}; ^1H NMR (300 MHz, $CDCl_3$) δ 1.21 (s, 9H), 2.01 (s, 3H), 5.95−6.03 (m, 1H), 6.17 (t, *J* = 3.1 Hz, 1H), 6.68 (dd, *J* = 2.7, 1.9 Hz, 1H), 7.01 (dd, *J* = 7.7, 1.5 Hz, 1H), 7.24 (td, *J* = 7.6, 1.5 Hz, 1H), 7.38 (td, *J* = 7.5, 1.5 Hz, 1H), 7.60 (dd, *J* = 8.2, 1.4 Hz, 1H); ^{13}C NMR (75 MHz, $CDCl_3$) δ 12.7, 31.5, 35.8, 106.4, 106.9, 123.2, 126.3, 128.3, 128.4, 130.7, 131.4, 138.3, 147.5; HRMS calcd for $[C_{15}H_{20}N]^+$ $(M + H)^+$: 214.1590; found: 214.1596.

2-Methyl-1-(2-(trifluoromethyl)phenyl)-1*H*-pyrrole (1j)

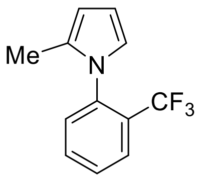

Following the **general procedure**, **1j** was obtained as a colorless oil (chromatography on silica gel, eluent: EtOAc/*n*-hexane = 1/100): IR (film) ν_{max}: 1502, 1312, 768, 701 cm^{-1}; ^1H NMR (300 MHz, $CDCl_3$) δ 2.00 (s, 3H), 5.99−6.07 (m, 1H), 6.16−6.24 (m, 1H), 6.65−6.72 (m, 1H), 7.32 (d, *J* = 7.8 Hz, 1H), 7.56 (t, *J* = 7.8 Hz, 1H), 7.64 (t, *J* = 7.8 Hz, 1H), 7.80 (d, *J* = 7.8 Hz, 1H); ^{13}C NMR (75 MHz, $CDCl_3$) δ 12.0, 106.9, 107.7, 122.9, 123.0 (q, *J* = 273.6 Hz), 127.0 (q, *J* = 5.0 Hz), 128.5, 130.6, 131.1, 132.4, 138.5; HRMS calcd for $[C_{12}H_{11}F_3N]^+$ $(M + H)^+$: 226.0838; found: 226.0844.

1-(2,3-Dihydro-1*H*-inden-4-yl)-2-methyl-1*H*-pyrrole (1k)

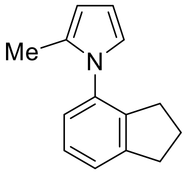

Following the **general procedure**, **1k** was obtained as a colorless oil (chromatography on silica gel, eluent: EtOAc/*n*-hexane = 1/80): IR (film) ν_{max}: 2944, 1489, 1330, 781, 699 cm^{-1}; ^1H NMR (300 MHz, $CDCl_3$) δ 2.07 (quint, *J* = 7.4 Hz, 2H), 2.09 (s, 3H), 2.73 (t, *J* = 7.4 Hz, 2H), 3.01 (t, *J* = 7.4 Hz, 2H), 5.98−6.07 (m, 1H), 6.19 (t, *J* = 3.1 Hz,

1H), 6.66 (dd, J = 2.7, 1.9 Hz, 1H), 7.00−7.08 (m, 1H), 7.21 (t, J = 7.4 Hz, 1H), 7.24−7.31 (m, 1H); ^{13}C NMR (75 MHz, $CDCl_3$) δ 12.3, 25.1, 30.8, 33.3, 106.8, 107.4, 121.0, 123.8, 124.9, 126.9, 129.2, 136.8, 141.5, 146.1; HRMS calcd for $[C_{14}H_{16}N]^+$ $(M + H)^+$: 198.1277; found: 198.1283.

2-Methyl-1-(5,6,7,8-tetrahydronaphthalen-1-yl)-1*H*-pyrrole (1l)

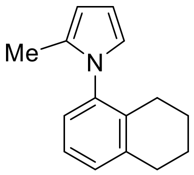

Following the **general procedure**, **1l** was obtained as a colorless oil (chromatography on silica gel, eluent: EtOAc/*n*-hexane = 1/100): IR (film) ν_{max}: 2930, 2858, 1485, 780, 699 cm^{-1}; ^{1}H NMR (300 MHz, $CDCl_3$) δ 1.65−1.85 (m, 4H), 1.99 (s, 3H), 2.21 (dt, J = 17.3, 6.0 Hz, 1H), 2.37 (dt, J = 17.3, 6.3 Hz, 1H), 2.85 (t, J = 6.3 Hz, 2H), 5.98−6.07 (m, 1H), 6.19 (t, J = 3.1 Hz, 1H), 6.58 (dd, J = 2.7, 1.9 Hz, 1H), 6.99−7.07 (m, 1H), 7.11−7.21 (m, 2H); ^{13}C NMR (75 MHz, $CDCl_3$) δ 12.1, 22.7, 22.8, 24.6, 29.5, 106.3, 107.3, 120.9, 125.4, 125.6, 129.2, 129.3, 135.6, 138.7, 139.2; HRMS calcd for $[C_{15}H_{18}N]^+$ $(M + H)^+$: 212.1434; found: 212.1439.

2-Methyl-1-(naphthalen-1-yl)-1*H*-pyrrole (1m)

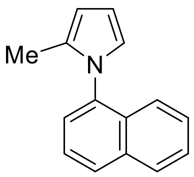

Following the **general procedure**, **1m** was obtained as a colorless oil (chromatography on silica gel, eluent: EtOAc/*n*-hexane = 1/60): IR (film) ν_{max}: 3092, 2977, 1507, 1446, 776, 720 cm^{-1}; ^{1}H NMR (300 MHz, $CDCl_3$) δ 2.01 (s, 3H), 6.11−6.19 (m, 1H), 6.32 (t, J = 3.1 Hz, 1H), 6.81 (dd, J = 2.7, 1.9 Hz, 1H), 7.31−7.40 (m, 1H), 7.43−7.62 (m, 4H), 7.90−7.99 (m, 2H); ^{13}C NMR (75 MHz, $CDCl_3$) δ 12.2, 106.8, 107.7, 122.7, 123.4, 125.1, 125.2, 126.5, 127.1, 128.0, 128.4, 130.8, 131.4, 134.1, 137.1; HRMS calcd for $[C_{15}H_{14}N]^+$ $(M + H)^+$: 208.1121; found: 208.1126.

1-(2-Iodophenyl)-2-methyl-1*H*-pyrrole (1o)

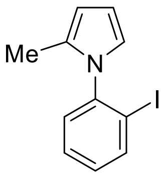

Following the **general procedure**, **1o** was obtained as a white solid (chromatography on silica gel, eluent: EtOAc/*n*-hexane = 1/60): IR (film) ν_{max}: 3066, 2907, 1485, 1049, 771, 708 cm^{-1}; ^{1}H NMR (300 MHz, $CDCl_3$) δ 2.04 (s, 3H), 6.02−6.08 (m, 1H), 6.23 (t, J = 3.1 Hz, 1H), 6.60 (dd, J = 2.8, 1.9 Hz, 1H), 7.13 (td, J = 7.7, 1.7 Hz, 1H), 7.32 (dd, J = 7.8, 1.7 Hz, 1H), 7.43 (td, J = 7.6, 1.4 Hz, 1H), 7.94 (dd, J = 7.9, 1.4 Hz, 1H); ^{13}C NMR (75 MHz, $CDCl_3$) δ 12.4, 99.1, 107.1, 108.0, 120.8, 128.9, 129.1, 129.4, 129.8, 139.5, 143.2; HRMS calcd for $[C_{11}H_{11}IN]^+$ $(M + H)^+$: 283.9931; found: 283.9939.

1-(2-Bromophenyl)-2-methyl-1*H*-pyrrole (1p)

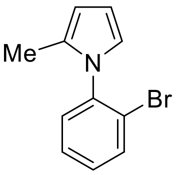

Following the **general procedure**, **1p** was obtained as a colorless oil (chromatography on silica gel, eluent: EtOAc/*n*-hexane = 1/30): IR (film) ν_{max}: 2914, 1489, 1330, 760, 696 cm^{-1}; ^{1}H NMR (300 MHz, $CDCl_3$) δ 2.05 (s, 3H), 6.02−6.09 (m, 1H), 6.23 (t, *J* = 3.1 Hz, 1H), 6.64 (dd, *J* = 2.7, 1.9 Hz, 1H), 7.25−7.37 (m, 2H), 7.37−7.46 (m, 1H), 7.70 (dd, *J* = 7.9, 1.3 Hz, 1H); ^{13}C NMR (75 MHz, $CDCl_3$) δ 12.2, 107.0, 108.0, 121.1, 123.2, 128.0, 129.6, 129.8 (2C), 133.3, 139.7; HRMS calcd for $[C_{11}H_{11}BrN]^+$ $(M + H)^+$: 236.0069 and 238.0049; found: 236.0076 and 238.0056.

1-(2-Chlorophenyl)-2-methyl-1*H*-pyrrole (1q)

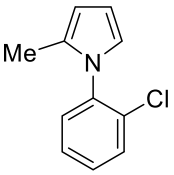

Following the **general procedure**, **1q** was obtained as a colorless oil (chromatography on silica gel, eluent: EtOAc/*n*-hexane = 1/30): IR (film) ν_{max}: 2916, 1492, 1331, 760, 697 cm^{-1}; ^{1}H NMR (300 MHz, $CDCl_3$) δ 2.06 (s, 3H), 6.01−6.10 (m, 1H), 6.24 (t, *J* = 3.1 Hz, 1H), 6.65 (dd, *J* = 2.8, 1.9 Hz, 1H), 7.29−7.42 (m, 3H), 7.48−7.58 (m, 1H); ^{13}C NMR (75 MHz, $CDCl_3$) δ 12.0, 107.0, 108.1, 121.3, 127.3, 129.3, 129.8, 130.0, 130.2, 132.9, 138.0; HRMS calcd for $[C_{11}H_{11}ClN]^+$ $(M + H)^+$: 192.0575; found: 192.0580.

2-(2-Methyl-1*H*-pyrrol-1-yl)aniline (1r)

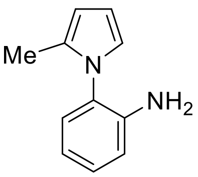

Following the **general procedure**, **1r** was obtained as a white solid (chromatography on silica gel, eluent: EtOAc/*n*-hexane = 1/10): IR (film) ν_{max}: 3442, 3347, 1616, 1504, 1321, 754, 705 cm^{-1}; ^{1}H NMR (300 MHz, $CDCl_3$) δ 2.06 (s, 3H), 3.02−3.87 (br, 2H), 6.01−6.09 (m, 1H), 6.23 (t, *J* = 3.1 Hz, 1H), 6.63 (dd, *J* = 2.7, 1.8 Hz, 1H), 6.75−6.85 (m, 2H), 7.06−7.15 (m, 1H), 7.16−7.25 (m, 1H); ^{13}C NMR (75 MHz, $CDCl_3$) δ 11.8, 107.1, 108.4, 115.7, 118.1, 120.8, 126.1, 128.6, 129.2, 129.8, 143.6; HRMS calcd for $[C_{11}H_{13}N_2]^+$ $(M + H)^+$: 173.1073; found: 173.1078.

2-Methyl-1-(2-nitrophenyl)-1*H*-pyrrole (1s)

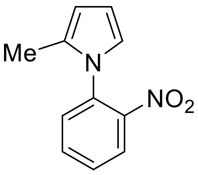

Following the **general procedure**, **1s** was obtained as an orange oil (chromatography on silica gel, eluent: EtOAc/*n*-hexane = 1/15): IR (film) ν_{max}: 2917, 1526, 1495, 1350, 755, 699 cm^{-1}; ^{1}H NMR (300 MHz, $CDCl_3$) δ 2.05 (s, 3H), 6.03−6.09 (m, 1H), 6.23 (t, *J* = 3.2 Hz, 1H), 6.62 (dd, *J* = 2.8, 1.9 Hz, 1H), 7.44 (dd, *J* = 7.9, 1.3 Hz, 1H), 7.56 (td, *J* = 8.0, 1.5 Hz, 1H), 7.67 (td, *J* = 7.7, 1.5 Hz, 1H), 7.95 (dd, *J* = 8.0, 1.5 Hz, 1H); ^{13}C NMR (75 MHz,

$CDCl_3$) δ 11.9, 108.3, 109.4, 121.3, 124.8, 128.8, 130.1, 130.5, 133.1, 133.7, 147.2; HRMS calcd for $[C_{11}H_{11}N_2O_2]^+$ $(M + H)^+$: 203.0815; found: 203.0820.

In a deviation from the standard synthesis route, **1n** was prepared by following procedure:

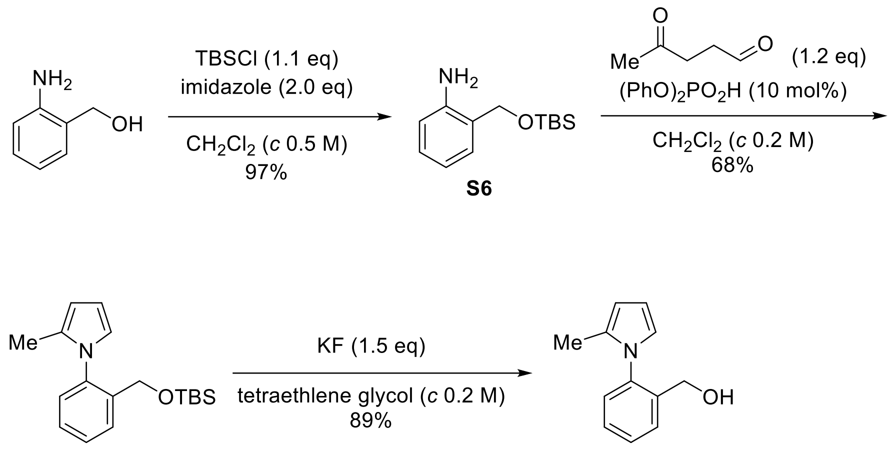

S6 **S7** **1n**

To a 0 °C solution of (2-aminophenyl)methanol (1.23 g, 10 mmol, 1.0 eq) in CH_2Cl_2 (20 mL, *c* 0.5 M) was added imidazole (1.36 g, 20 mmol, 2.0 eq) and TBSCl (1.66 g, 11 mmol, 1.1 eq). The reaction mixture was stirred at 0 °C for 1 hour. After completion, the reaction mixture was diluted with 100 mL CH_2Cl_2, washed with aqueous $NaHCO_3$ (sat.) and brine, and was dried over anhydrous sodium sulfate. After filtration, the solvent was evaporated under reduced pressure, and the residue was purified by column chromatography (silica gel, eluted by EtOAc/*n*-hexane = 1/20) to afford compound **S6** as a colorless oil (2.30 g, 97% yield).

To a solution of **S6** (665 mg, 2.8 mmol, 1.0 eq) in CH_2Cl_2 (14 mL, *c* 0.2 M) was added 4-oxopentanal (336 mg, 3.36 mmol, 1.2 eq) and $(PhO)_2PO_2H$ (70 mg, 0.28 mmol, 10 mol%). The reaction mixture was stirred at room temperature until full conversion of **S6** was reached as monitored by TLC analysis. Then the solvent was evaporated under reduced pressure, and the residue was purified by column chromatography (silica gel, eluted by EtOAc/*n*-hexane = 1/80) to afford compound **S7** as a colorless oil (576 mg, 68% yield).

Following a literature procedure,[2] a mixture of **S7** (576 mg, 1.9 mmol, 1.0 eq) and KF (166 mg, 2.86 mmol, 1.5 eq) in tetraethylene glycol (9.0 mL, *c* 0.2 M) was stirred at 80 °C for 4 hours. After being cooled to room temperature, the reaction mixture was diluted with 100 mL H_2O and was extracted with Et_2O (5×50 mL). The combined organic layer was washed with brine and was dried over anhydrous sodium sulfate. After filtration, the solvent was evaporated under reduced pressure, and the residue was purified by column chromatography (silica gel, eluted by EtOAc/*n*-hexane = 1/5) to afford **1n** as a white solid (320 mg, 89% yield): IR (film) ν_{max}: 3353, 2971, 1265, 950, 735 cm^{-1}; ^1H NMR

(300 MHz, $CDCl_3$) δ 1.52−1.69 (br, 1H), 2.01 (s, 3H), 4.40 (d, *J* = 3.1 Hz, 2H), 6.00−6.08 (m, 1H), 6.21 (t, *J* = 3.1 Hz, 1H), 6.64 (dd, *J* = 2.6, 1.8 Hz, 1H), 7.25 (dd, *J* = 7.6, 1.4 Hz, 1H), 7.38 (td, *J* = 7.6, 1.6 Hz, 1H), 7.45 (td, *J* = 7.4, 1.6 Hz, 1H), 7.58 (dd, *J* = 7.6, 1.4 Hz, 1H); ^{13}C NMR (75 MHz, $CDCl_3$) δ 12.1, 61.3, 107.0, 108.1, 121.4, 128.2, 128.3, 128.59, 128.64, 129.8, 138.2, 138.6; HRMS calcd for $[C_{12}H_{14}NO]^+$ $(M + H)^+$: 188.1070; found: 188.1075.

3. Synthesis of Acrylpyrazoles

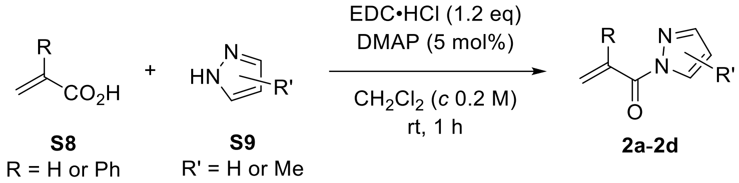

To a solution of commercially available pyrazoles **S9** (10 mmol, 1.0 eq) and acrylic acids **S8** (12 mmol, 1.2 eq) in CH_2Cl_2 (50 mL, *c* 0.2 M) in a 250 mL round bottom flask was added DMAP (61 mg, 0.5 mmol, 5 mol%) and EDC hydrochloride (2.29 g, 12 mmol, 1.2 eq). The reaction mixture was stirred at room temperature for 1 hour. After full conversion of pyrazole was reached as monitored by TLC, the solvent was evaporated under reduced pressure and the residue was purified by column chromatography (silica gel, eluted by a mixture of EtOAc and *n*-hexane) to afford compounds **2a-2d**.

1-(1*H*-Pyrazol-1-yl)prop-2-en-1-one (2a)

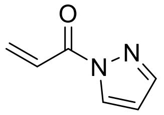

Following the **general procedure**, **2a** was obtained as a colorless oil (chromatography on silica gel, eluent: EtOAc/*n*-hexane = 1/20): IR (film) ν_{max}: 3130, 1714, 1380, 1338, 915, 764 cm^{-1}; ^{1}H NMR (300 MHz, $CDCl_3$) δ 6.08 (dd, *J* = 10.5, 1.6 Hz, 1H), 6.47 (dd, *J* = 2.8, 1.5 Hz, 1H), 6.74 (dd, *J* = 17.3, 1.6 Hz, 1H), 7.56 (dd, *J* = 17.3, 10.5 Hz, 1H), 7.70−7.81 (m, 1H), 8.33 (d, *J* = 2.8 Hz, 1H); ^{13}C NMR (75 MHz, $CDCl_3$) δ 110.0, 126.4, 128.7, 133.4, 144.1, 163.1; HRMS calcd for $[C_6H_6N_2NaO]^+$ $(M + Na)^+$: 145.0372; found: 145.0376.

1-(3-Methyl-1*H*-pyrazol-1-yl)prop-2-en-1-one (2b)

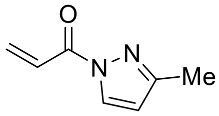

Following the **general procedure**, **2b** was obtained as a colorless oil (chromatography on silica gel, eluent: EtOAc/*n*-hexane = 1/20): IR (film) ν_{max}: 3123, 2930, 1709, 1415, 1342, 926, 792 cm^{-1}; ^{1}H NMR (300 MHz, $CDCl_3$) δ 2.34 (s, 3H),

6.04 (dd, J = 10.5, 1.7 Hz, 1H), 6.28 (d, J = 2.8, 1H), 6.70 (dd, J = 17.4, 1.7 Hz, 1H), 7.52 (dd, J = 17.4, 10.5 Hz, 1H), 8.22 (d, J = 2.8 Hz, 1H); ^{13}C NMR (75 MHz, $CDCl_3$) δ 13.9, 110.8, 126.7, 129.4, 132.8, 154.0, 162.8; HRMS calcd for $[C_7H_9N_2O]^+$ $(M + H)^+$: 137.0709; found: 137.0713.

1-(3,5-Dimethyl-1*H*-pyrazol-1-yl)prop-2-en-1-one (2c)

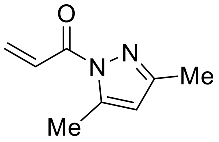

Following the **general procedure**, **2c** was obtained as a white solid (chromatography on silica gel, eluent: EtOAc/*n*-hexane = 1/20): IR (film) ν_{max}: 2983, 2925, 1715, 1408, 1346, 988, 785 cm^{-1}; ^{1}H NMR (300 MHz, $CDCl_3$) δ 2.25 (s, 3H), 2.58 (s, 3H), 5.96 (dd, J = 10.5, 1.8 Hz, 1H), 5.99 (s, 1H), 6.61 (dd, J = 17.4, 1.8 Hz, 1H), 7.58 (dd, J = 17.4, 10.5 Hz, 1H); ^{13}C NMR (75 MHz, $CDCl_3$) δ 13.8, 14.5, 111.5, 128.4, 131.5, 144.5, 152.1, 164.9; HRMS calcd for $[C_8H_{11}N_2O]^+$ $(M + H)^+$: 151.0866; found: 151.0870.

2-Phenyl-1-(1*H*-pyrazol-1-yl)prop-2-en-1-one (2d)

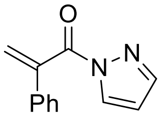

Following the **general procedure**, **2d** was obtained as a colorless oil (chromatography on silica gel, eluent: EtOAc/*n*-hexane = 1/5): IR (film) ν_{max}: 3128, 1710, 1379, 916, 762, 695 cm^{-1}; ^{1}H NMR (300 MHz, $CDCl_3$) δ 5.89 (s, 1H), 6.14 (s, 1H), 6.48 (dd, J = 2.8, 1.5 Hz, 1H), 7.30−7.47 (m, 5H), 7.68−7.74 (m, 1H), 8.32 (d, J = 2.8 Hz, 1H); ^{13}C NMR (75 MHz, $CDCl_3$) δ 109.9, 122.0, 126.4, 128.6, 128.7, 129.6, 136.0, 142.6, 144.7, 167.4; HRMS calcd for $[C_{12}H_{11}N_2O]^+$ (M + H)$^+$: 199.0866; found: 199.0871.

4. Initial Conditions and Optimization of Pyrazole Auxiliaries

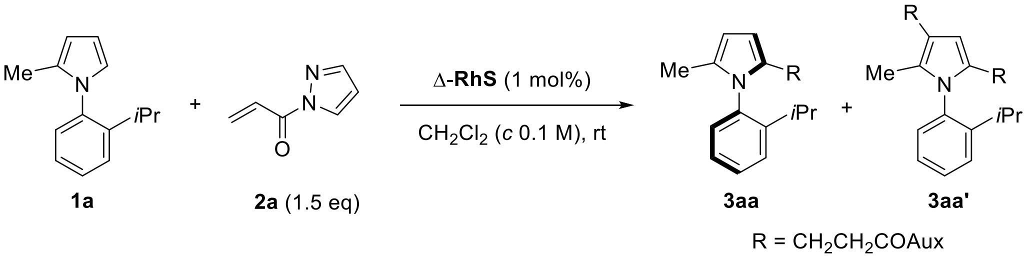

Initial reaction conditions: to a 10 mL Schlenk tube was added Δ-**RhS** (0.9 mg, 1 μmol, 1 mol%), *N*-arylpyrrole **1a** (19.9 mg, 0.1 mmol, 1.0 eq), acrylpyrazole **2a** (18.3 mg, 0.15 mmol, 1.5 eq) and CH_2Cl_2 (1 mL, c 0.1 M). The reaction mixture was stirred at room temperature until full conversion of **1a** was reached monitored by TLC. Then the reaction was quenched by addition of CH_3CN (1 mL) before removal of solvent under reduced pressure. The yield of desired product **3aa** was determined by

NMR analysis to be 53% (using hexamethylbenzene as an internal standard), and the *ee* was determined by chiral HPLC analysis to be 98%. In addition to **3aa**, we observed the by-product **3aa'** formed in 36% yield, and the formation of which diminished the yield by eroding the formed product. The reactions of **1a** with **2b** or **2c** that have different pyrazole auxiliaries were conducted under similar condition.

3,3'-(1-(2-Isopropylphenyl)-5-methyl-1*H*-pyrrole-2,4-diyl)bis(1-(1*H*-pyrazol-1-yl)propan-1-one) (3aa')

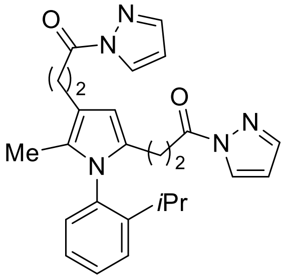

3aa' was a colorless oil (chromatography on silica gel, eluent: EtOAc/*n*-hexane = 1/10): IR (film) ν_{max}: 2962, 1733, 1382, 764 cm^{-1}; ^1H NMR (300 MHz, $CDCl_3$) δ 1.10 (d, *J* = 6.9 Hz, 3H), 1.13 (d, *J* = 6.9 Hz, 3H), 1.86 (s, 3H), 2.42 (hept, *J* = 6.9 Hz, 1H), 2.56−2.78 (m, 2H), 2.93 (t, *J* = 7.7 Hz, 2H), 3.27−3.49 (m, 4H), 5.93 (s, 1H), 6.41 (dd, *J* = 2.8, 1.5 Hz, 1H), 6.44 (dd, *J* = 2.8, 1.5 Hz, 1H), 7.11−7.17 (m, 1H), 7.22−7.29 (m, 1H), 7.38−7.44 (m, 2H), 7.66−7.70 (m, 1H), 7.70−7.75 (m, 1H), 8.20 (d, *J* = 2.8 Hz, 1H), 8.27 (d, *J* = 2.8 Hz, 1H); ^{13}C NMR (75 MHz, $CDCl_3$) δ 10.2, 21.4, 21.5, 23.7, 23.8, 27.3, 33.4, 35.7, 105.2, 109.3, 109.4, 116.9, 125.5, 126.4, 126.6, 128.1 (2C), 129.0, 129.2, 130.4, 136.2, 143.7, 143.8, 147.6, 171.4, 172.0; HRMS calcd for $[C_{26}H_{29}N_5O_2Na]^+$ $(M + Na)^+$: 466.2213; found: 466.2225.

(*aR*)-3-(1-(2-Isopropylphenyl)-5-methyl-1*H*-pyrrol-2-yl)-1-(3-methyl-1*H*-pyrazol-1-yl)propan-1-one (3ab)

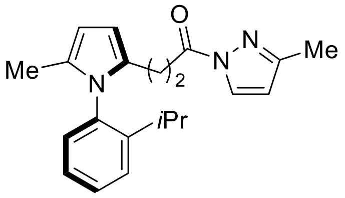

3ab was obtained as a colorless oil (chromatography on silica gel, eluent: EtOAc/*n*-hexane = 1/20, 24.4 mg, 73% yield) with 96% *ee* [DAICEL CHIRALCEL OD-H column, Agilent HPLC 1260, *i*PrOH/hexane = 2/98 (v/v), 1.0 mL/min, 25 °C, 254 nm; t_1 = 5.0 min, t_2 = 6.0 min]: $[\alpha]_D^{25}$ = −92.6 (*c* 1.0, CH_2Cl_2, 96% *ee*); IR (film) ν_{max}: 2963, 1727, 1408, 917, 756 cm^{-1}; ^1H NMR (300 MHz, $CDCl_3$) δ 1.14 (d, *J* = 6.9 Hz, 3H), 1.18 (d, *J* = 6.9 Hz, 3H), 1.96 (s, 3H), 2.31 (s, 3H), 2.45 (hept, *J* = 6.9 Hz, 1H), 2.60−2.83 (m, 2H), 3.26−3.47 (m, 2H), 5.96 (d, *J* = 3.3 Hz, 1H), 6.01 (d, *J* = 3.3 Hz, 1H), 6.22 (d, *J* = 2.8 Hz, 1H), 7.16−7.22 (m, 1H), 7.23−7.34 (m, 1H), 7.40−7.50 (m, 2H), 8.10 (d, *J* = 2.8 Hz, 1H); ^{13}C NMR (75 MHz, $CDCl_3$) δ 12.6, 13.8, 21.8, 23.7, 23.8, 27.3, 33.3, 104.6, 105.5, 110.1, 126.4, 126.7, 128.8, 128.99, 129.05, 129.2, 131.6, 136.1, 147.5, 153.6, 171.0; HRMS calcd for $[C_{21}H_{26}N_3O]^+$ $(M + H)^+$: 336.2070; found: 336.2079.

(*aR*)-1-(3,5-Dimethyl-1*H*-pyrazol-1-yl)-3-(1-(2-isopropylphenyl)-5-methyl-1*H*-pyrrol-2-yl)propan-1-one (3ac)

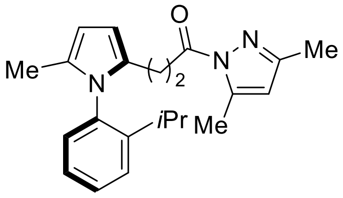

3ac was obtained as a colorless oil (chromatography on silica gel, eluent: EtOAc/*n*-hexane = 1/20, 21.7 mg, 62% yield) with 97% *ee* [DAICEL CHIRALCEL OD-H column, Agilent HPLC 1260, *i*PrOH/hexane = 1/99 (v/v), 1.0 mL/min, 25 °C, 254 nm; t_1 = 4.9 min, t_2 = 5.7 min]: $[\alpha]_D^{25}$ = −80.5 (*c* 1.0, CH_2Cl_2, 97% *ee*); IR (film) ν_{max}: 2962, 2927, 1725, 1378, 960, 756 cm^{-1}; ^1H NMR (300 MHz, $CDCl_3$) δ 1.14 (d, *J* = 6.9 Hz, 3H), 1.17 (d, *J* = 6.9 Hz, 3H), 1.95 (s, 3H), 2.22 (s, 3H), 2.48 (hept, *J* = 6.9 Hz, 1H), 2.50 (s, 3H), 2.57−2.79 (m, 2H), 3.28−3.48 (m, 2H), 5.93 (s, 1H), 5.96 (d, *J* = 3.3 Hz, 1H), 6.01 (d, *J* = 3.3 Hz, 1H), 7.15−7.22 (m, 1H), 7.24−7.33 (m, 1H), 7.39−7.48 (m, 2H); ^{13}C NMR (75 MHz, $CDCl_3$) δ 12.6, 13.7, 14.4, 21.9, 23.76, 23.80, 27.3, 34.6, 104.5, 105.4, 110.9, 126.4, 126.6, 128.95, 129.05, 129.1, 131.9, 136.1, 143.8, 147.5, 151.7, 173.1; HRMS calcd for $[C_{22}H_{28}N_3O]^+$ (M + H)$^+$: 350.2227; found: 350.2236.

5. General Procedure for Δ-RhS Catalyzed Reactions

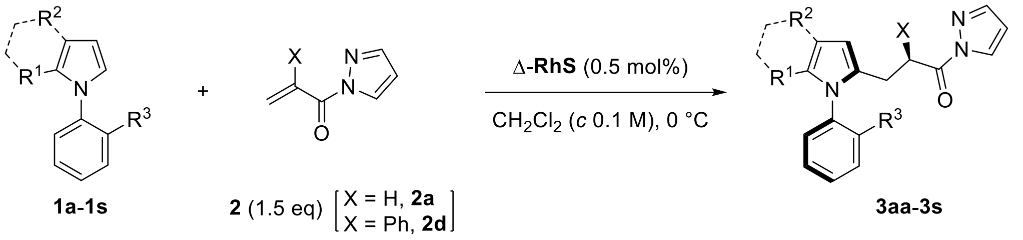

To a 10 mL Schlenk tube was added Δ-**RhS** (0.4 mg, 0.5 μmol, 0.5 mol%), *N*-arylpyrrole **1** (0.1 mmol, 1.0 eq), acrylpyrazole **2** (0.15 mmol, 1.5 eq) and CH_2Cl_2 (1 mL, *c* 0.1 M). The reaction mixture was stirred at 0 °C (or at −20 °C for **1q**) until full conversion of **1** was reached monitored by TLC. Then the reaction was quenched by addition of CH_3CN (1 mL) before removal of solvent under reduced pressure. The residue was purified by column chromatography (silica gel, eluted by a mixture of EtOAc and *n*-hexane) to afford desired products **3**.

(*aR*)-3-(1-(2-Isopropylphenyl)-5-methyl-1*H*-pyrrol-2-yl)-1-(1*H*-pyrazol-1-yl)propan-1-one (3aa)

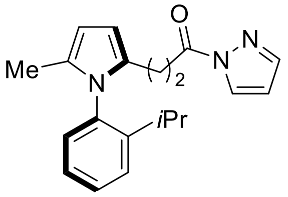

Following the **general procedure**, the reaction of *N*-arylpyrrole **1a** (19.9 mg, 0.1 mmol) with acrylpyrazole **2a** (18.3 mg, 0.15 mmol) gave the desired product **3aa** as a colorless oil (chromatography on silica gel, eluent: EtOAc/*n*-hexane = 1/20, 29.6 mg, 92% yield) with 99% *ee* [DAICEL CHIRALCEL OD-

H column, Agilent HPLC 1260, *i*PrOH/hexane = 2/98 (v/v), 1.0 mL/min, 25 °C, 254 nm; t_1 = 5.3 min, t_2 = 6.7 min]: $[\alpha]_D^{25}$ = −89.4 (*c* 1.0, CH_2Cl_2, 99% *ee*); IR (film) ν_{max}: 2963, 1734, 1349, 756 cm^{-1}; ^1H NMR (300 MHz, $CDCl_3$) δ 1.13 (d, *J* = 6.9 Hz, 3H), 1.16 (d, *J* = 6.9 Hz, 3H), 1.94 (s, 3H), 2.46 (hept, *J* = 6.9 Hz, 1H), 2.59−2.84 (m, 2H), 3.31−3.50 (m, 2H), 5.94 (d, *J* = 3.3 Hz, 1H), 5.99 (d, *J* = 3.3 Hz, 1H), 6.41 (dd, *J* = 2.8, 1.5 Hz, 1H), 7.12−7.21 (m, 1H), 7.23−7.33 (m, 1H), 7.38−7.49 (m, 2H), 7.64−7.72 (m, 1H), 8.21 (d, *J* = 2.8 Hz, 1H); ^{13}C NMR (75 MHz, $CDCl_3$) δ 12.6, 21.7, 23.7, 23.9, 27.4, 33.3, 104.6, 105.5, 109.4, 126.4, 126.7, 128.2, 129.1 (2C), 129.4, 131.4, 136.1, 143.8, 147.6, 171.4; HRMS calcd for $[C_{20}H_{23}N_3ONa]^+$ $(M + Na)^+$: 344.1733; found: 344.1742.

(*aR*)-3-(5-Ethyl-1-(2-isopropylphenyl)-1*H*-pyrrol-2-yl)-1-(1*H*-pyrazol-1-yl)propan-1-one (3b)

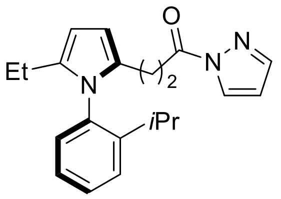

Following the **general procedure**, the reaction of *N*-arylpyrrole **1b** (21.3 mg, 0.1 mmol) with acrylpyrazole **2a** (18.3 mg, 0.15 mmol) gave the desired product **3b** as a colorless oil (chromatography on silica gel, eluent: EtOAc/*n*-hexane = 1/20, 30.2 mg, 90% yield) with 99% *ee* [DAICEL CHIRALCEL OD-H column, Agilent HPLC 1260, *i*PrOH/hexane = 2/98 (v/v), 1.0 mL/min, 25 °C, 254 nm; t_1 = 4.8 min, t_2 = 5.4 min]: $[\alpha]_D^{25}$ = −62.5 (*c* 1.0, CH_2Cl_2, 99% *ee*); IR (film) ν_{max}: 2964, 1734, 1382, 921, 758 cm^{-1}; ^1H NMR (250 MHz, $CDCl_3$) δ 1.03−1.23 (m, 9H), 2.21 (q, *J* = 7.5 Hz, 2H), 2.42 (hept, *J* = 6.9 Hz, 1H), 2.57−2.85 (m, 2H), 3.30−3.53 (m, 2H), 5.97 (d, *J* = 3.4 Hz, 1H), 6.03 (d, *J* = 3.4 Hz, 1H), 6.41 (dd, *J* = 2.8, 1.5 Hz, 1H), 7.14−7.22 (m, 1H), 7.22−7.33 (m, 1H), 7.36−7.49 (m, 2H), 7.62−7.74 (m, 1H), 8.21 (d, *J* = 2.8 Hz, 1H); ^{13}C NMR (63 MHz, $CDCl_3$) δ 12.9, 20.0, 21.6, 23.80, 23.83, 27.3, 33.2, 103.6, 104.5, 109.4, 126.4, 126.7, 128.2, 129.0, 129.2, 131.3, 135.96, 136.02, 143.8, 147.5, 171.4; HRMS calcd for $[C_{21}H_{25}N_3ONa]^+$ $(M + Na)^+$: 358.1890; found: 358.1900.

(*aS*)-3-(5-Isopropyl-1-(2-isopropylphenyl)-1*H*-pyrrol-2-yl)-1-(1*H*-pyrazol-1-yl)propan-1-one (3c)

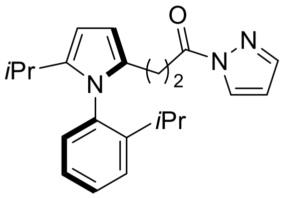

Following the **general procedure**, the reaction of *N*-arylpyrrole **1c** (22.7 mg, 0.1 mmol) with acrylpyrazole **2a** (18.3 mg, 0.15 mmol) gave the desired product **3c** as a colorless oil (chromatography on silica gel, eluent: EtOAc/*n*-hexane = 1/25, 31.9 mg, 91% yield) with 94% *ee* [DAICEL CHIRALCEL OD-H column, Agilent HPLC 1260, *i*PrOH/hexane = 1/99 (v/v), 1.0 mL/min, 25 °C, 254 nm; t_1 = 4.9 min, t_2 = 5.6 min]: $[\alpha]_D^{25}$ = −61.0 (*c* 1.0, CH_2Cl_2, 94% *ee*); IR (film) ν_{max}: 2962, 2926, 1735, 1383, 758 cm^{-1}; ^1H NMR (300 MHz, $CDCl_3$) δ 1.02 (d, *J* = 6.9 Hz, 3H), 1.12 (d, *J* = 6.9 Hz, 3H), 1.14 (d, *J* = 6.9 Hz, 3H), 1.19 (d, *J* = 6.9 Hz, 3H), 2.35 (hept, *J* = 6.9 Hz, 1H), 2.39 (hept, *J* = 6.9 Hz, 1H), 2.58−2.81 (m, 2H), 3.30−3.52 (m, 2H), 5.98 (d, *J* = 3.5 Hz, 1H), 6.03 (d, *J* = 3.5 Hz, 1H), 6.40 (dd, *J* = 2.8, 1.5 Hz, 1H),

7.20−7.33 (m, 2H), 7.38−7.48 (m, 2H), 7.64−7.72 (m, 1H), 8.20 (d, *J* = 2.8 Hz, 1H); ^{13}C NMR (75 MHz, $CDCl_3$) δ 21.6, 22.2, 23.6, 24.0, 25.1, 25.9, 27.2, 33.2, 101.9, 104.7, 109.4, 126.2, 126.7, 128.1, 129.0, 129.4, 130.8, 136.0, 141.4, 143.8, 147.7, 171.4; HRMS calcd for $[C_{22}H_{27}N_3ONa]^+$ $(M + Na)^+$: 372.2046; found: 372.2057.

(*aS*)-3-(1-(2-Isopropylphenyl)-5-phenyl-1*H*-pyrrol-2-yl)-1-(1*H*-pyrazol-1-yl)propan-1-one (3d)

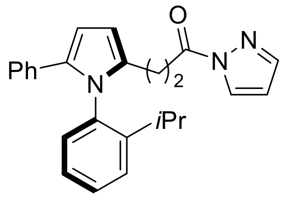

Following the **general procedure**, the reaction of *N*-arylpyrrole **1d** (26.1 mg, 0.1 mmol) with acrylpyrazole **2a** (18.3 mg, 0.15 mmol) gave the desired product **3d** as a yellow oil (chromatography on silica gel, eluent: EtOAc/*n*-hexane = 1/15, 35.8 mg, 93% yield) with 90% *ee* [DAICEL CHIRALCEL OD-H column, Agilent HPLC 1260, *i*PrOH/hexane = 1/99 (v/v), 1.0 mL/min, 25 °C, 254 nm; t_1 = 7.3 min, t_2 = 7.8 min]: $[\alpha]_D^{25}$ = −131.8 (*c* 1.0, CH_2Cl_2, 90% *ee*); IR (film) ν_{max}: 2962, 1733, 1382, 749, 695 cm^{-1}; ^{1}H NMR (250 MHz, $CDCl_3$) δ 0.58 (d, *J* = 6.9 Hz, 3H), 1.02 (d, *J* = 6.9 Hz, 3H), 2.31 (hept, *J* = 6.9 Hz, 1H), 2.68−2.95 (m, 2H), 3.47 (t, *J* = 7.7 Hz, 2H), 6.19 (d, *J* = 3.6 Hz, 1H), 6.38−6.48 (m, 2H), 6.99−7.18 (m, 5H), 7.23−7.35 (m, 2H), 7.35−7.49 (m, 2H), 7.65−7.74 (m, 1H), 8.23 (d, *J* = 2.8 Hz, 1H); ^{13}C NMR (63 MHz, $CDCl_3$) δ 21.8, 22.6, 24.2, 27.3, 33.1, 106.2, 107.8, 109.5, 125.8, 126.2, 127.0, 127.5, 127.9, 128.2, 129.1, 129.6, 133.3, 134.2, 135.0, 136.4, 143.9, 147.2, 171.2; HRMS calcd for $[C_{25}H_{25}N_3ONa]^+$ $(M + Na)^+$: 406.1890; found: 406.1900.

(*aR*)-3-(1-(2-Isopropylphenyl)-4,5-dimethyl-1*H*-pyrrol-2-yl)-1-(1*H*-pyrazol-1-yl)propan-1-one (3e)

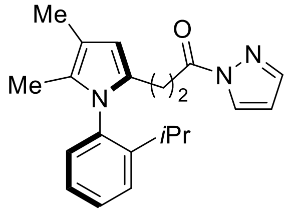

Following the **general procedure**, the reaction of *N*-arylpyrrole **1e** (21.3 mg, 0.1 mmol) with acrylpyrazole **2a** (18.3 mg, 0.15 mmol) gave the desired product **3e** as a colorless oil (chromatography on silica gel, eluent: EtOAc/*n*-hexane = 1/20, 21.2 mg, 63% yield) with 90% *ee* [DAICEL CHIRALCEL OD-H column, Agilent HPLC 1260, *i*PrOH/hexane = 2/98 (v/v), 1.0 mL/min, 25 °C, 254 nm; t_1 = 4.8 min, t_2 = 7.1 min]: $[\alpha]_D^{25}$ = −87.2 (*c* 1.0, CH_2Cl_2, 90% *ee*); IR (film) ν_{max}: 2963, 2922, 1594, 1382, 758 cm^{-1}; ^{1}H NMR (300 MHz, $CDCl_3$) δ 1.12 (d, *J* = 6.9 Hz, 3H), 1.15 (d, *J* = 6.9 Hz, 3H), 1.84 (s, 3H), 2.06 (s, 3H), 2.50 (hept, *J* = 6.9 Hz, 1H), 2.58−2.84 (m, 2H), 3.28−3.51 (m, 2H), 5.88 (s, 1H), 6.41 (dd, *J* = 2.8, 1.5 Hz, 1H), 7.11−7.19 (m, 1H), 7.22−7.31 (m, 1H), 7.36−7.47 (m, 2H), 7.64−7.71 (m, 1H), 8.21 (d, *J* = 2.8 Hz, 1H); ^{13}C NMR (75 MHz, $CDCl_3$) δ 10.2, 11.3, 21.4, 23.76, 23.84, 27.3, 33.4, 106.5, 109.4, 113.6, 125.4, 126.4, 126.6, 128.1, 128.9, 129.2, 130.1, 136.4, 143.8, 147.7, 171.4; HRMS calcd for $[C_{21}H_{26}N_3O]^+$ $(M + H)^+$: 336.2070; found: 336.2079.

(*aR*)-3-(1-(2-Isopropylphenyl)-4,5,6,7-tetrahydro-1*H*-indol-2-yl)-1-(1*H*-pyrazol-1-yl)propan-1-one (3f)

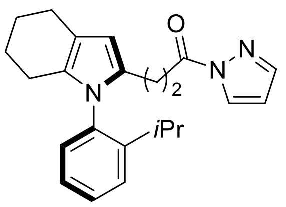

Following the **general procedure**, the reaction of *N*-arylpyrrole **1f** (23.9 mg, 0.1 mmol) with acrylpyrazole **2a** (18.3 mg, 0.15 mmol) gave the desired product **3f** as a colorless oil (chromatography on silica gel, eluent: EtOAc/*n*-hexane = 1/20, 20.1 mg, 56% yield) with 96% *ee* [DAICEL CHIRALCEL OD-H column, Agilent HPLC 1260, *i*PrOH/hexane = 2/98 (v/v), 1.0 mL/min, 25 °C, 254 nm; t_1 = 4.7 min, t_2 = 5.2 min]: $[\alpha]_D^{25}$ = −58.1 (*c* 1.0, CH_2Cl_2, 96% *ee*); IR (film) ν_{max}: 2925, 2845, 1381, 758 cm^{-1}; ^1H NMR (300 MHz, $CDCl_3$) δ 1.12 (d, *J* = 6.9 Hz, 3H), 1.16 (d, *J* = 6.9 Hz, 3H), 1.65−1.86 (m, 4H), 2.00−2.16 (m, 1H), 2.16−2.31 (m, 1H), 2.45−2.62 (m, 3H), 2.62−2.87 (m, 2H), 3.29−3.53 (m, 2H), 5.89 (s, 1H), 6.41 (dd, *J* = 2.8, 1.5 Hz, 1H), 7.12−7.20 (m, 1H), 7.21−7.30 (m, 1H), 7.35−7.47 (m, 2H), 7.62−7.72 (m, 1H), 8.21 (d, *J* = 2.8 Hz, 1H); ^{13}C NMR (75 MHz, $CDCl_3$) δ 21.3, 22.5, 23.0, 23.4, 23.7, 23.9, 24.0, 27.4, 33.4, 104.2, 109.4, 116.3, 126.3, 126.6, 128.2, 128.8 (2C), 129.1, 130.8, 135.9, 143.8, 147.6, 171.4; HRMS calcd for $[C_{23}H_{27}N_3ONa]^+$ $(M + Na)^+$: 384.2046; found: 384.2056.

(*aR*)-3-(5-Methyl-1-(*o*-tolyl)-1*H*-pyrrol-2-yl)-1-(1*H*-pyrazol-1-yl)propan-1-one (3g)

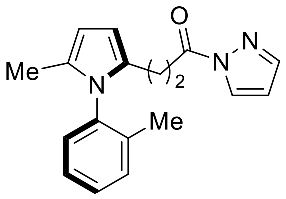

Following the **general procedure**, the reaction of *N*-arylpyrrole **1g** (17.1 mg, 0.1 mmol) with acrylpyrazole **2a** (18.3 mg, 0.15 mmol) gave the desired product **3g** as a colorless oil (chromatography on silica gel, eluent: EtOAc/*n*-hexane = 1/20, 20.5 mg, 70% yield) with 97% *ee* [DAICEL CHIRALCEL OD-H column, Agilent HPLC 1260, *i*PrOH/hexane = 2/98 (v/v), 1.0 mL/min, 25 °C, 254 nm; t_1 = 6.9 min, t_2 = 8.9 min]: $[\alpha]_D^{25}$ = −52.3 (*c* 1.0, CH_2Cl_2, 97% *ee*); IR (film) ν_{max}: 2920, 1732, 1383, 760 cm^{-1}; ^1H NMR (300 MHz, $CDCl_3$) δ 1.92 (s, 3H), 1.96 (s, 3H), 2.62−2.81 (m, 2H), 3.26−3.47 (m, 2H), 5.94 (d, *J* = 3.3 Hz, 1H), 5.99 (d, *J* = 3.3 Hz, 1H), 6.41 (dd, *J* = 2.8, 1.5 Hz, 1H), 7.18−7.25 (m, 1H), 7.25−7.41 (m, 3H), 7.64−7.72 (m, 1H), 8.20 (d, *J* = 2.8 Hz, 1H); ^{13}C NMR (75 MHz, $CDCl_3$) δ 12.4, 17.0, 21.6, 33.3, 104.8, 105.6, 109.4, 126.7, 128.1, 128.6, 128.8, 128.9, 130.7, 130.8, 137.1, 137.7, 143.8, 171.3; HRMS calcd for $[C_{18}H_{20}N_3O]^+$ $(M + H)^+$: 294.1601; found: 294.1609.

(*aR*)-3-(1-(2-Butylphenyl)-5-methyl-1*H*-pyrrol-2-yl)-1-(1*H*-pyrazol-1-yl)propan-1-one (3h)

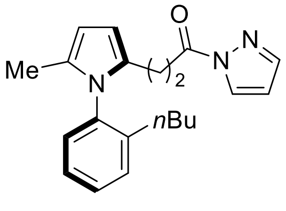

Following the **general procedure**, the reaction of *N*-arylpyrrole **1h** (21.3 mg, 0.1 mmol) with acrylpyrazole **2a** (18.3 mg, 0.15 mmol) gave the desired product **3h** as a colorless oil (chromatography on silica gel, eluent: EtOAc/*n*-hexane = 1/25, 23.9 mg, 71% yield) with 98% *ee* [DAICEL CHIRALCEL OD-

H column, Agilent HPLC 1260, *i*PrOH/hexane = 2/98 (v/v), 1.0 mL/min, 25 °C, 254 nm; t_1 = 5.1 min, t_2 = 7.3 min]: $[\alpha]_D^{25}$ = −96.6 (*c* 1.0, CH_2Cl_2, 98% *ee*); IR (film) ν_{max}: 2927, 1734, 1382, 750 cm^{-1}; ^1H NMR (300 MHz, $CDCl_3$) δ 0.84 (t, *J* = 7.3 Hz, 3H), 1.20−1.33 (m, 2H), 1.39−1.56 (m, 2H), 1.92 (s, 3H), 2.16−2.31 (m, 2H), 2.59−2.83 (m, 2H), 3.26−3.48 (m, 2H), 5.93 (d, *J* = 3.4 Hz, 1H), 5.98 (d, *J* = 3.4 Hz, 1H), 6.41 (dd, *J* = 2.8, 1.5 Hz, 1H), 7.14−7.23 (m, 1H), 7.23−7.33 (m, 1H), 7.33−7.44 (m, 2H), 7.62−7.73 (m, 1H), 8.20 (d, *J* = 2.8 Hz, 1H); ^{13}C NMR (75 MHz, $CDCl_3$) δ 12.6, 13.8, 21.7, 22.6, 29.9, 31.8, 33.3, 104.8, 105.6, 109.4, 126.6, 128.1, 128.6, 129.0, 129.2, 129.6, 131.0, 137.2, 141.5, 143.8, 171.4; HRMS calcd for $[C_{21}H_{26}N_3O]^+$ $(M + H)^+$: 336.2070; found: 336.2079.

(*aR*)-3-(1-(2-(*tert*-Butyl)phenyl)-5-methyl-1*H*-pyrrol-2-yl)-1-(1*H*-pyrazol-1-yl)propan-1-one (3i)

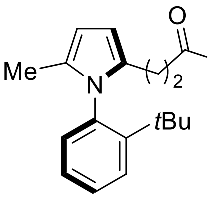

The reaction was conducted under **modified reaction condition**: to a 10 mL Schlenk tube was added Δ-**RhS** (0.4 mg, 0.5 μmol, 0.5 mol%), *N*-arylpyrrole **1i** (53.3 mg, 0.25 mmol, 2.5 eq), acrylpyrazole **2a** (12.2 mg, 0.1 mmol, 1.0 eq) and CH_2Cl_2 (1 mL, *c* 0.1 M). The reaction mixture was stirred at 0 °C for 2 hours until full conversion of **2a** was reached monitored by TLC. Then the solvent was removed under reduced pressure, and the residue was purified by column chromatography (silica gel, eluent: EtOAc/*n*-hexane = 1/25, 30.4 mg, 91% yield) to give desired product **3i** as a colorless oil with >99% *ee* [DAICEL CHIRALCEL OD-H column, Agilent HPLC 1260, *i*PrOH/hexane = 2/98 (v/v), 1.0 mL/min, 25 °C, 254 nm; t_1 = 5.2 min, t_2 = 7.2 min]: $[\alpha]_D^{25}$ = −86.5 (*c* 1.0, CH_2Cl_2, >99% *ee*); IR (film) ν_{max}: 2969, 1739, 1385, 758 cm^{-1}; ^1H NMR (300 MHz, $CDCl_3$) δ 1.16 (s, 9H), 1.94 (s, 3H), 2.55−2.71 (m, 1H), 2.74−2.90 (m, 1H), 3.31−3.50 (m, 2H), 5.89 (d, *J* = 3.4 Hz, 1H), 5.93 (d, *J* = 3.4 Hz, 1H), 6.41 (dd, *J* = 2.8, 1.5 Hz, 1H), 6.97 (dd, *J* = 7.8, 1.5 Hz, 1H), 7.23 (td, *J* = 7.3, 1.5 Hz, 1H), 7.38 (td, *J* = 7.7, 1.5 Hz, 1H), 7.61 (dd, *J* = 8.2, 1.5 Hz, 1H), 7.65−7.71 (m, 1H), 8.21 (d, *J* = 2.9 Hz, 1H); ^{13}C NMR (75 MHz, $CDCl_3$) δ 13.1, 22.3, 31.6, 33.2, 36.1, 104.6, 105.6, 109.4, 126.6, 128.2, 128.6, 129.8, 130.1, 131.6, 132.3, 135.6, 143.8, 147.7, 171.4; HRMS calcd for $[C_{21}H_{26}N_3O]^+$ $(M + H)^+$: 336.2070; found: 336.2080.

(*aR*)-3-(5-Methyl-1-(2-(trifluoromethyl)phenyl)-1*H*-pyrrol-2-yl)-1-(1*H*-pyrazol-1-yl)propan-1-one (3j)

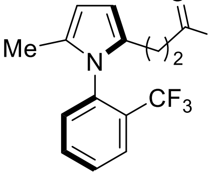

Following the **general procedure**, the reaction of *N*-arylpyrrole **1j** (22.5 mg, 0.1 mmol) with acrylpyrazole **2a** (18.3 mg, 0.15 mmol) gave the desired product **3j** as a colorless oil (chromatography on silica gel, eluent: EtOAc/*n*-hexane = 1/8, 30.2 mg, 87% yield) with >99% *ee* [DAICEL CHIRALCEL OD-H column, Agilent HPLC 1260, *i*PrOH/hexane = 5/95 (v/v), 1.0 mL/min, 25 °C, 254 nm; t_1 = 6.1 min, t_2

= 11.9 min]: $[\alpha]_D^{25}$ = −72.7 (*c* 1.0, CH_2Cl_2, >99% *ee*); IR (film) ν_{max}: 2971, 1266, 949, 734 cm^{-1}; ^{1}H NMR (300 MHz, $CDCl_3$) δ 1.92 (s, 3H), 2.54−2.69 (m, 1H), 2.76 (ddd, *J* = 15.4, 9.0, 6.4 Hz, 1H), 3.29−3.52 (m, 2H), 5.93 (d, *J* = 3.3 Hz, 1H), 5.99 (d, *J* = 3.3 Hz, 1H), 6.41 (dd, *J* = 2.8, 1.5 Hz, 1H), 7.30−7.39 (m, 1H), 7.54−7.64 (m, 1H), 7.64−7.75 (m, 2H), 7.79−7.88 (m, 1H), 8.20 (d, *J* = 2.8 Hz, 1H); ^{13}C NMR (75 MHz, $CDCl_3$) δ 12.3, 21.4, 33.1, 105.1, 106.1, 109.4, 122.9 (q, *J* = 273.8 Hz), 127.3 (q, *J* = 5.1 Hz), 128.1, 129.0, 129.4 (q, *J* = 30.4 Hz), 130.2, 131.9, 132.5, 132.9, 137.0 (q, *J* = 1.6 Hz), 143.9, 171.3; HRMS calcd for $[C_{18}H_{16}F_3N_3ONa]^+$ $(M + Na)^+$: 370.1138; found: 370.1147.

(*aR*)-3-(1-(2,3-Dihydro-1*H*-inden-4-yl)-5-methyl-1*H*-pyrrol-2-yl)-1-(1*H*-pyrazol-1-yl)propan-1-one (3k)

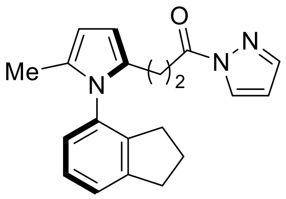

Following the **general procedure**, the reaction of *N*-arylpyrrole **1k** (19.7 mg, 0.1 mmol) with acrylpyrazole **2a** (18.3 mg, 0.15 mmol) gave the desired product **3k** as a colorless oil (chromatography on silica gel, eluent: EtOAc/*n*-hexane = 1/20, 27.9 mg, 87% yield) with 91% *ee* [DAICEL CHIRALCEL OD-H column, Agilent HPLC 1260, *i*PrOH/hexane = 2/98 (v/v), 1.0 mL/min, 25 °C, 254 nm; t_1 = 7.2 min, t_2 = 7.8 min]: $[\alpha]_D^{25}$ = −57.5 (*c* 1.0, CH_2Cl_2, 91% *ee*); IR (film) ν_{max}: 2921, 1733, 1382, 762 cm^{-1}; ^{1}H NMR (300 MHz, $CDCl_3$) δ 1.96 (s, 3H), 2.00−2.17 (m, 2H), 2.51−2.71 (m, 2H), 2.77 (t, *J* = 7.6 Hz, 2H), 3.02 (t, *J* = 7.6 Hz, 2H), 3.24−3.47 (m, 2H), 5.93 (d, *J* = 3.4 Hz, 1H), 5.98 (d, *J* = 3.4 Hz, 1H), 6.41 (dd, *J* = 2.8, 1.5 Hz, 1H), 6.97−7.08 (m, 1H), 7.20−7.27 (m, 1H), 7.27−7.34 (m, 1H), 7.63−7.72 (m, 1H), 8.20 (d, *J* = 2.8 Hz, 1H); ^{13}C NMR (75 MHz, $CDCl_3$) δ 12.4, 21.6, 25.0, 30.6, 33.2, 33.4, 104.8, 105.5, 109.4, 124.4, 126.1, 127.2, 128.1, 128.8, 130.6, 135.0, 143.1, 143.7, 146.1, 171.3; HRMS calcd for $[C_{20}H_{21}N_3ONa]^+$ $(M + Na)^+$: 342.1577; found: 342.1585.

(*aR*)-3-(5-Methyl-1-(5,6,7,8-tetrahydronaphthalen-1-yl)-1*H*-pyrrol-2-yl)-1-(1*H*-pyrazol-1-yl)propan-1-one (3l)

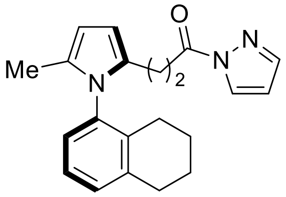

Following the **general procedure**, the reaction of *N*-arylpyrrole **1l** (21.1 mg, 0.1 mmol) with acrylpyrazole **2a** (18.3 mg, 0.15 mmol) gave the desired product **3l** as a colorless oil (chromatography on silica gel, eluent: EtOAc/*n*-hexane = 1/20, 27.6 mg, 83% yield) with 99% *ee* [DAICEL CHIRALCEL OD-H column, Agilent HPLC 1260, *i*PrOH/hexane = 2/98 (v/v), 1.0 mL/min, 25 °C, 254 nm; t_1 = 6.0 min, t_2 = 7.5 min]: $[\alpha]_D^{25}$ = −70.5 (*c* 1.0, CH_2Cl_2, 99% *ee*); IR (film) ν_{max}: 2929, 1733, 1382, 768 cm^{-1}; ^{1}H NMR (300 MHz, $CDCl_3$) δ 1.66−1.88 (m, 4H), 1.92 (s, 3H), 2.08−2.28 (m, 2H), 2.62−2.80 (m, 2H), 2.81−2.93 (m, 2H), 3.26−3.49 (m, 2H), 5.94 (d, *J* = 3.4 Hz, 1H), 5.99 (d, *J* = 3.4 Hz, 1H), 6.41 (dd, *J* =

2.8, 1.5 Hz, 1H), 7.04 (dd, J = 7.1, 2.0 Hz, 1H), 7.13−7.28 (m, 2H), 7.63−7.73 (m, 1H), 8.21 (d, J = 2.8 Hz, 1H); ^{13}C NMR (75 MHz, $CDCl_3$) δ 12.4, 21.6, 22.6, 22.8, 24.5, 29.4, 33.3, 104.7, 105.4, 109.4, 125.9, 126.1, 128.1, 128.5, 129.5, 130.4, 136.2, 137.4, 138.9, 143.8, 171.4; HRMS calcd for $[C_{21}H_{23}N_3ONa]^+$ $(M + Na)^+$: 356.1733; found: 356.1742.

(*aR*)-3-(5-Methyl-1-(naphthalen-1-yl)-1*H*-pyrrol-2-yl)-1-(1*H*-pyrazol-1-yl)propan-1-one (3m)

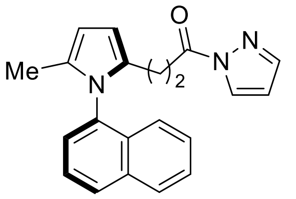

Following the **general procedure**, the reaction of *N*-arylpyrrole **1m** (20.7 mg, 0.1 mmol) with acrylpyrazole **2a** (18.3 mg, 0.15 mmol) gave the desired product **3m** as a colorless oil (chromatography on silica gel, eluent: EtOAc/*n*-hexane = 1/10, 19.6 mg, 60% yield) with 98% *ee* [DAICEL CHIRALCEL OD-H column, Agilent HPLC 1260, *i*PrOH/hexane = 5/95 (v/v), 1.0 mL/min, 25 °C, 254 nm; t_1 = 6.5 min, t_2 = 10.2 min]: $[\alpha]_D^{25}$ = −93.6 (c 1.0, CH_2Cl_2, 98% *ee*); IR (film) ν_{max}: 2915, 1733, 1347, 770 cm^{-1}; ^{1}H NMR (300 MHz, $CDCl_3$) δ 1.91 (s, 3H), 2.61−2.82 (m, 2H), 3.22−3.44 (m, 2H), 6.06 (d, J = 3.3 Hz, 1H), 6.11 (d, J = 3.3 Hz, 1H), 6.37 (dd, J = 2.8, 1.5 Hz, 1H), 7.15 (d, J = 8.3 Hz, 1H), 7.42−7.62 (m, 4H), 7.62−7.68 (m, 1H), 7.90−8.02 (m, 2H), 8.15 (d, J = 2.8 Hz, 1H); ^{13}C NMR (75 MHz, $CDCl_3$) δ 12.3, 21.6, 33.5, 105.1, 105.7, 109.3, 123.1, 125.4, 126.4, 126.6, 127.3, 128.0 (2C), 128.8, 130.3, 131.9, 132.3, 134.2, 135.3, 143.7, 171.2; HRMS calcd for $[C_{21}H_{19}N_3ONa]^+$ $(M + Na)^+$: 352.1420; found: 352.1429.

(*aR*)-3-(1-(2-(Hydroxymethyl)phenyl)-5-methyl-1*H*-pyrrol-2-yl)-1-(1*H*-pyrazol-1-yl)propan-1-one (3n)

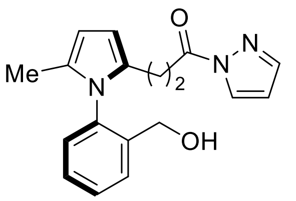

Following the **general procedure**, the reaction of *N*-arylpyrrole **1n** (18.7 mg, 0.1 mmol) with acrylpyrazole **2a** (18.3 mg, 0.15 mmol) gave the desired product **3n** as a colorless oil (chromatography on silica gel, eluent: EtOAc/*n*-hexane = 1/3, 28.5 mg, 92% yield) with 98% *ee* [DAICEL CHIRALPAK IG column, Agilent HPLC 1260, *i*PrOH/hexane = 20/80 (v/v), 1.0 mL/min, 25 °C, 254 nm; t_1 = 10.8 min, t_2 = 13.0 min]: $[\alpha]_D^{25}$ = −27.9 (c 1.0, CH_2Cl_2, 98% *ee*); IR (film) ν_{max}: 3299, 2920, 1731, 1383, 754 cm^{-1}; ^{1}H NMR (300 MHz, $CDCl_3$) δ 1.89 (s, 3H), 2.56−2.88 (m, 3H), 3.20 (ddd, J = 17.2, 7.6, 6.6 Hz, 1H), 3.42 (dt, J = 17.2, 7.6 Hz, 1H), 4.25 (d, J = 13.4 Hz, 1H), 4.32 (d, J = 13.4 Hz, 1H), 5.91 (d, J = 3.3 Hz, 1H), 5.94 (d, J = 3.3 Hz, 1H), 6.40 (dd, J = 2.8, 1.5 Hz, 1H), 7.21 (dd, J = 7.6, 1.0 Hz, 1H), 7.39 (td, J = 7.6, 1.4 Hz, 1H), 7.48 (td, J = 7.5, 1.1 Hz, 1H), 7.61−7.75 (m, 2H), 8.18 (d, J = 2.8 Hz, 1H); ^{13}C NMR (75 MHz, $CDCl_3$) δ 12.4, 21.5, 33.2, 60.4, 105.2, 105.9, 109.6, 128.3, 128.4, 128.9, 129.0 (2C), 129.4,

131.0, 136.2, 140.0, 144.0, 171.4; HRMS calcd for $[C_{18}H_{19}N_3O_2Na]^+$ $(M + Na)^+$: 332.1369; found: 332.1378.

(*aR*)-3-(1-(2-Iodophenyl)-5-methyl-1*H*-pyrrol-2-yl)-1-(1*H*-pyrazol-1-yl)propan-1-one (3o)

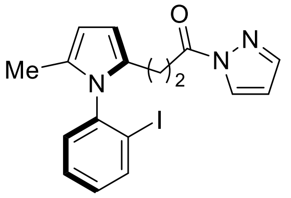

Following the **general procedure**, the reaction of *N*-arylpyrrole **1o** (28.3 mg, 0.1 mmol) with acrylpyrazole **2a** (18.3 mg, 0.15 mmol) gave the desired product **3o** as a colorless oil (chromatography on silica gel, eluent: EtOAc/*n*-hexane = 1/10, 34.5 mg, 85% yield) with 98% *ee* [DAICEL CHIRALCEL OD-H column, Agilent HPLC 1260, *i*PrOH/hexane = 5/95 (v/v), 1.0 mL/min, 25 °C, 254 nm; t_1 = 7.7 min, t_2 = 11.4 min]: $[\alpha]_D^{25}$ = −105.9 (*c* 1.0, CH_2Cl_2, 98% *ee*); IR (film) ν_{max}: 2917, 1735, 949, 734 cm^{-1}; ^1H NMR (300 MHz, $CDCl_3$) δ 1.95 (s, 3H), 2.61−2.86 (m, 2H), 3.30−3.54 (m, 2H), 5.96 (d, *J* = 3.4 Hz, 1H), 6.01 (d, *J* = 3.4 Hz, 1H), 6.41 (dd, *J* = 2.8, 1.5 Hz, 1H), 7.16 (td, *J* = 7.8, 1.6 Hz, 1H), 7.34 (dd, *J* = 7.8, 1.6 Hz, 1H), 7.48 (td, *J* = 7.8, 1.3 Hz, 1H), 7.64−7.71 (m, 1H), 7.96 (dd, *J* = 8.1, 1.2 Hz, 1H), 8.20 (d, *J* = 2.8 Hz, 1H); ^{13}C NMR (75 MHz, $CDCl_3$) δ 12.7, 21.7, 33.4, 100.6, 105.4, 106.1, 109.4, 128.1, 128.6, 129.2, 129.9, 130.1, 130.5, 139.6, 141.7, 143.8, 171.3; HRMS calcd for $[C_{17}H_{16}IN_3ONa]^+$ $(M + Na)^+$: 428.0230; found: 428.0241.

(*aR*)-3-(1-(2-Bromophenyl)-5-methyl-1*H*-pyrrol-2-yl)-1-(1*H*-pyrazol-1-yl)propan-1-one (3p)

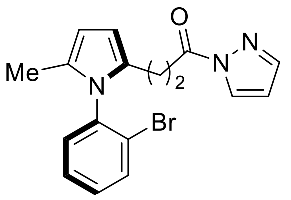

Following the **general procedure**, the reaction of *N*-arylpyrrole **1p** (23.6 mg, 0.1 mmol) with acrylpyrazole **2a** (18.3 mg, 0.15 mmol) gave the desired product **3p** as a colorless oil (chromatography on silica gel, eluent: EtOAc/*n*-hexane = 1/10, 29.0 mg, 81% yield) with 95% *ee* [DAICEL CHIRALCEL OD-H column, Agilent HPLC 1260, *i*PrOH/hexane = 5/95 (v/v), 1.0 mL/min, 25 °C, 254 nm; t_1 = 7.2 min, t_2 = 11.3 min]: $[\alpha]_D^{25}$ = −65.9 (*c* 1.0, CH_2Cl_2, 95% *ee*); IR (film) ν_{max}: 2919, 1731, 1382, 759 cm^{-1}; ^1H NMR (300 MHz, $CDCl_3$) δ 1.96 (s, 3H), 2.64−2.86 (m, 2H), 3.29−3.50 (m, 2H), 5.96 (d, *J* = 3.4 Hz, 1H), 6.02 (d, *J* = 3.4 Hz, 1H), 6.41 (dd, *J* = 2.8, 1.5 Hz, 1H), 7.29−7.40 (m, 2H), 7.40−7.51 (m, 1H), 7.64−7.70 (m, 1H), 7.73 (dd, *J* = 7.9, 1.2 Hz, 1H), 8.20 (d, *J* = 2.8 Hz, 1H); ^{13}C NMR (75 MHz, $CDCl_3$) δ 12.4, 21.5, 33.4, 105.3, 106.0, 109.4, 124.5, 128.1, 128.3, 129.0, 130.0, 130.7, 131.0, 133.4, 138.1, 143.8, 171.3; HRMS calcd for $[C_{17}H_{16}BrN_3ONa]^+$ $(M + Na)^+$: 380.0369 and 382.0348; found: 380.0379 and 382.0359.

(*aR*)-3-(1-(2-Chlorophenyl)-5-methyl-1*H*-pyrrol-2-yl)-1-(1*H*-pyrazol-1-yl)propan-1-one (3q)

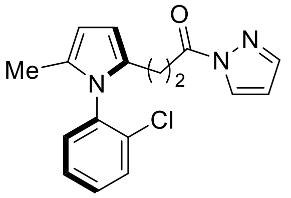

Following the **general procedure**, the reaction of *N*-arylpyrrole **1q** (19.2 mg, 0.1 mmol) with acrylpyrazole **2a** (18.3 mg, 0.15 mmol) gave the desired product **3q** as a colorless oil (chromatography on silica gel, eluent: EtOAc/*n*-hexane = 1/10, 26.7 mg, 85% yield) with 91% *ee* [DAICEL CHIRALCEL OD-H column, Agilent HPLC 1260, *i*PrOH/hexane = 5/95 (v/v), 1.0 mL/min, 25 °C, 254 nm; t_1 = 7.0 min, t_2 = 11.1 min]: $[\alpha]_D^{25}$ = −55.1 (*c* 1.0, CH_2Cl_2, 91% *ee*); IR (film) ν_{max}: 2914, 1733, 1383, 764, 740 cm^{-1}; ^{1}H NMR (300 MHz, $CDCl_3$) δ 1.96 (s, 3H), 2.66−2.87 (m, 2H), 3.27−3.50 (m, 2H), 5.97 (d, *J* = 3.4 Hz, 1H), 6.03 (d, *J* = 3.4 Hz, 1H), 6.41 (dd, *J* = 2.8, 1.5 Hz, 1H), 7.31−7.47 (m, 3H), 7.51−7.61 (m, 1H), 7.63−7.73 (m, 1H), 8.20 (d, *J* = 2.8 Hz, 1H); ^{13}C NMR (75 MHz, $CDCl_3$) δ 12.3, 21.5, 33.3, 105.3, 106.0, 109.4, 127.6, 128.1, 129.2, 129.8, 130.3, 130.8, 131.1, 134.1, 136.4, 143.8, 171.3; HRMS calcd for $[C_{17}H_{17}ClN_3O]^+$ $(M + H)^+$: 314.1055; found: 314.1064.

(*aR*)-3-(1-(2-Aminophenyl)-5-methyl-1*H*-pyrrol-2-yl)-1-(1*H*-pyrazol-1-yl)propan-1-one (3r)

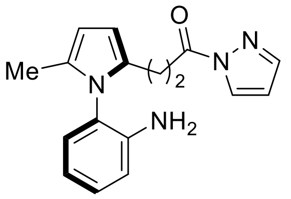

Following the **general procedure**, the reaction of *N*-arylpyrrole **1r** (17.2 mg, 0.1 mmol) with acrylpyrazole **2a** (18.3 mg, 0.15 mmol) gave the desired product **3r** as a yellow oil (chromatography on silica gel, eluent: EtOAc/*n*-hexane = 1/5, 17.0 mg, 58% yield) with 93% *ee* [DAICEL CHIRALCEL OD-H column, Agilent HPLC 1260, *i*PrOH/hexane = 10/90 (v/v), 1.0 mL/min, 25 °C, 254 nm; t_1 = 8.6 min, t_2 = 10.3 min]: $[\alpha]_D^{25}$ = −111.4 (*c* 0.5, CH_2Cl_2, 93% *ee*); IR (film) ν_{max}: 3185, 3061, 2978, 1677, 1503, 749 cm^{-1}; ^{1}H NMR (300 MHz, $CDCl_3$) δ 1.98 (s, 3H), 2.80 (t, *J* = 7.5 Hz, 2H), 3.24−3.61 (m, 4H), 5.94 (d, *J* = 3.4 Hz, 1H), 5.97 (d, *J* = 3.4 Hz, 1H), 6.41 (dd, *J* = 2.8, 1.5 Hz, 1H), 6.75−6.88 (m, 2H), 7.09 (dd, *J* = 8.2, 1.6 Hz, 1H), 7.22 (td, *J* = 7.8, 1.5 Hz, 1H), 7.62−7.73 (m, 1H), 8.20 (d, *J* = 2.8 Hz, 1H); ^{13}C NMR (75 MHz, $CDCl_3$) δ 12.1, 21.5, 33.4, 105.6, 106.2, 109.4, 115.7, 118.3, 123.9, 128.2, 129.1, 129.4, 129.5, 130.8, 143.8, 144.3, 171.4; HRMS calcd for $[C_{17}H_{19}N_4O]^+$ $(M + H)^+$: 295.1553; found: 295.1561.

(*aR*)-3-(5-Methyl-1-(2-nitrophenyl)-1*H*-pyrrol-2-yl)-1-(1*H*-pyrazol-1-yl)propan-1-one (3s)

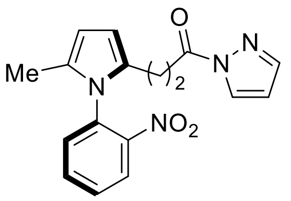

Following the **general procedure**, the reaction of *N*-arylpyrrole **1s** (20.2 mg, 0.1 mmol) with acrylpyrazole **2a** (18.3 mg, 0.15 mmol) gave the desired product **3s** as an orange oil (chromatography on silica gel, eluent: EtOAc/*n*-hexane = 1/4, 27.2 mg, 84% yield) with 96% *ee* [DAICEL CHIRALCEL OD-H column, Agilent HPLC 1260, *i*PrOH/hexane = 20/80 (v/v), 1.0 mL/min, 25 °C, 254 nm; t_1 = 8.8 min, t_2 = 16.0 min]: $[\alpha]_D^{25}$ = −40.1 (*c* 1.0, CH_2Cl_2, 96% *ee*); IR (film) ν_{max}: 2971, 1265, 949, 734 cm^{-1}; ^{1}H

NMR (300 MHz, $CDCl_3$) δ 1.94 (s, 3H), 2.66−2.86 (m, 2H), 3.27−3.52 (m, 2H), 5.95 (d, *J* = 3.4 Hz, 1H), 6.01 (d, *J* = 3.4 Hz, 1H), 6.40 (dd, *J* = 2.8, 1.5 Hz, 1H), 7.45 (dd, *J* = 7.8, 1.3 Hz, 1H), 7.62 (td, *J* = 7.8, 1.3 Hz, 1H), 7.65−7.69 (m, 1H), 7.74 (td, *J* = 7.7, 1.4 Hz, 1H), 8.00 (dd, *J* = 8.1, 1.4 Hz, 1H), 8.19 (d, *J* = 2.8 Hz, 1H); ^{13}C NMR (75 MHz, $CDCl_3$) δ 12.2, 21.4, 33.0, 106.2, 107.1, 109.5, 124.9, 128.1, 129.5, 129.6, 131.7, 131.8, 132.2, 133.4, 143.9, 148.2, 171.2; HRMS calcd for $[C_{17}H_{17}N_4O_3]^+$ $(M + H)^+$: 325.1295; found: 325.1304.

(*aR*,2*S*)-3-(1-(2-Isopropylphenyl)-5-methyl-1*H*-pyrrol-2-yl)-2-phenyl-1-(1*H*-pyrazol-1-yl)propan-1-one (3ad)

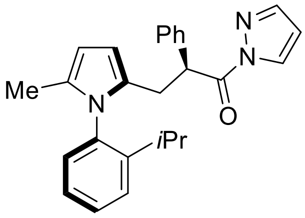

Following the **general procedure**, the reaction of *N*-arylpyrrole **1a** (19.9 mg, 0.1 mmol) with acrylpyrazole **2d** (29.7 mg, 0.15 mmol) gave the desired product **3ad** as a white solid (chromatography on silica gel, eluent: EtOAc/*n*-hexane = 1/15, 36.0 mg, 90% yield) with >20/1 *dr* and >99.5% *ee* [DAICEL CHIRALCEL OD-H column, Agilent HPLC 1260, *i*PrOH/hexane = 0.6/99.4 (v/v), 1.0 mL/min, 25 °C, 254 nm; t_1 = 6.9 min, t_2 = 7.2 min]: $[\alpha]_D^{25}$ = −103.0 (*c* 0.5, CH_2Cl_2, >99.5% *ee*); IR (film) ν_{max}: 2971, 1380, 1266, 950, 736 cm^{-1}; ^{1}H NMR (300 MHz, $CDCl_3$) δ 1.09 (d, *J* = 6.9 Hz, 3H), 1.12 (d, *J* = 6.9 Hz, 3H), 1.91 (s, 3H), 2.45 (hept, *J* = 6.9 Hz, 1H), 2.75 (dd, *J* = 15.6, 5.4 Hz, 1H), 3.23 (dd, *J* = 15.6, 10.0 Hz, 1H), 5.53 (dd, *J* = 10.0, 5.4 Hz, 1H), 5.82 (d, *J* = 3.4 Hz, 1H), 5.84 (d, *J* = 3.4 Hz, 1H), 6.37 (dd, *J* = 2.8, 1.5 Hz, 1H), 7.17−7.33 (m, 5H), 7.36−7.48 (m, 4H), 7.66−7.73 (m, 1H), 8.17 (d, *J* = 2.8 Hz, 1H); ^{13}C NMR (75 MHz, $CDCl_3$) δ 12.6, 23.7, 24.0, 27.3, 30.9, 47.7, 105.2, 105.6, 109.7, 126.5, 126.6, 127.4, 128.4, 128.55, 128.62, 129.0, 129.2, 129.4, 130.4, 136.0, 138.2, 143.9, 147.5, 171.9; HRMS calcd for $[C_{26}H_{27}N_3ONa]^+$ $(M + Na)^+$: 420.2046; found: 420.2057.

6. Determination of the Selectivity Factor for Kinetic Resolution of 1i

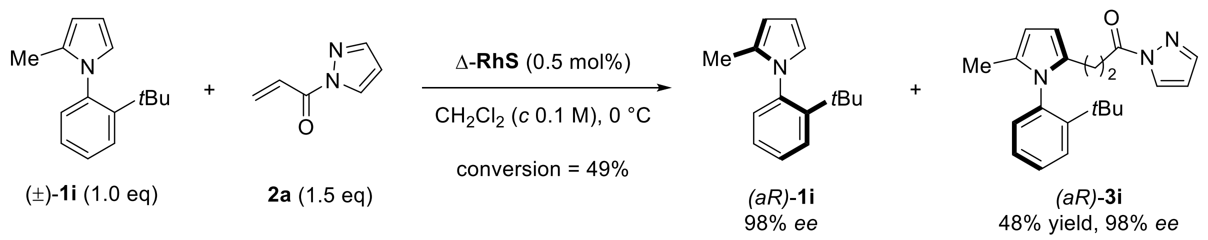

Following the **general procedure**, the reaction mixture of *N*-arylpyrrole **1i** (12.6 mg, 0.06 mmol, 1.0 eq) with acrylpyrazole **2a** (11.0 mg, 0.09 mmol, 1.5 eq) in the presence of **Δ-RhS** (0.3 mg, 0.3 μmol, 0.5 mol%) in CH_2Cl_2 (0.6 mL, *c* 0.1 M) was stirred at 0 °C for 2 hours. After that, TLC showed a nearly 1/1 ratio of unreacted **1i** and product **3i**. To prevent the racemization of **1i** during column purification,

the *ee* of unreacted **1i** was measured by HPLC analysis of crude mixture using a chiral stationary phase. The conversion of **1i** and the yield of **3i** was determined by ^{1}H NMR analysis of crude mixture using hexamethylbenzene as an internal standard. The *ee* of **3i** was measured by HPLC analysis of purified product using a chiral stationary phase.

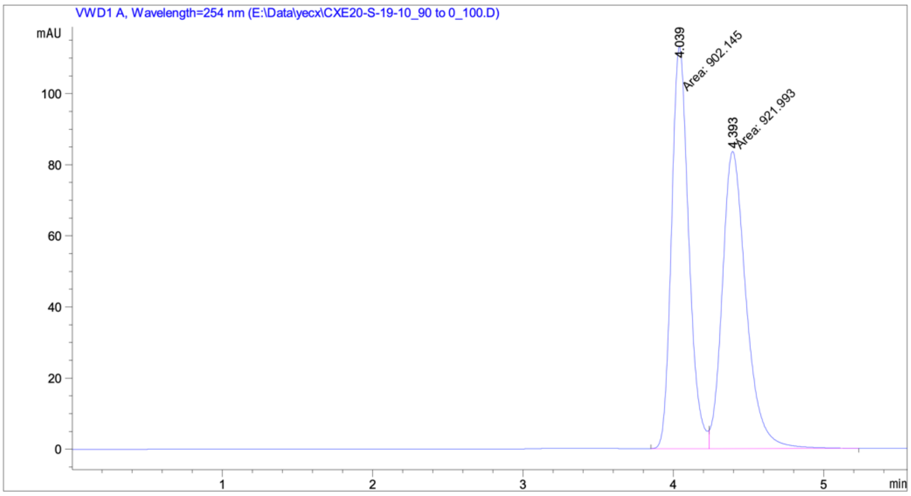

Peak #	RetTime [min]	Type	Width [min]	Area [mAU*s]	Height [mAU]	Area %
1	4.039	MF	0.1330	902.14508	113.07035	49.4560
2	4.393	FM	0.1838	921.99274	83.59541	50.5440

Figure S1. Racemate of compound **1i** (CHIRALCEL OJ, 25 °C, hexane, 1.0 mL/min, 254 nm).

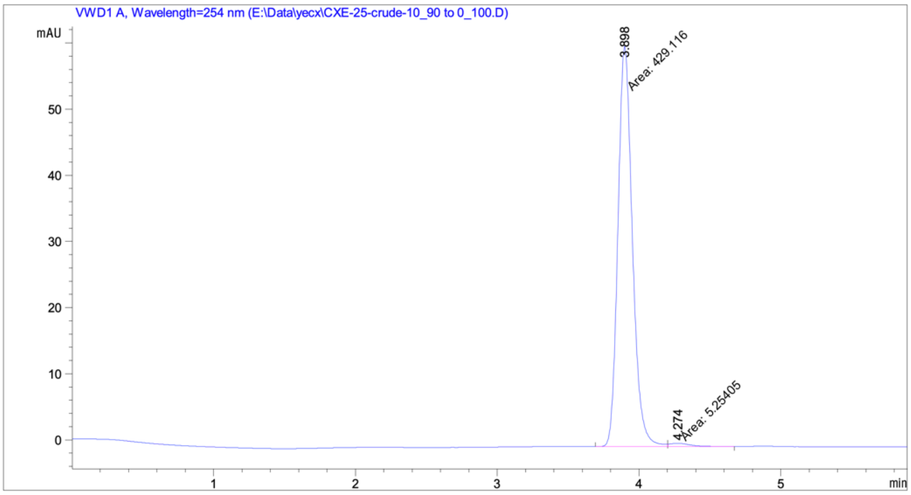

Peak #	RetTime [min]	Type	Width [min]	Area [mAU*s]	Height [mAU]	Area %
1	3.898	MF	0.1182	429.11560	60.50664	98.7904
2	4.274	FM	0.1750	5.25405	5.00461e-1	1.2096

Figure S2. Unreacted **1i** after reaction (CHIRALCEL OJ, 25 °C, hexane, 1.0 mL/min, 254 nm).

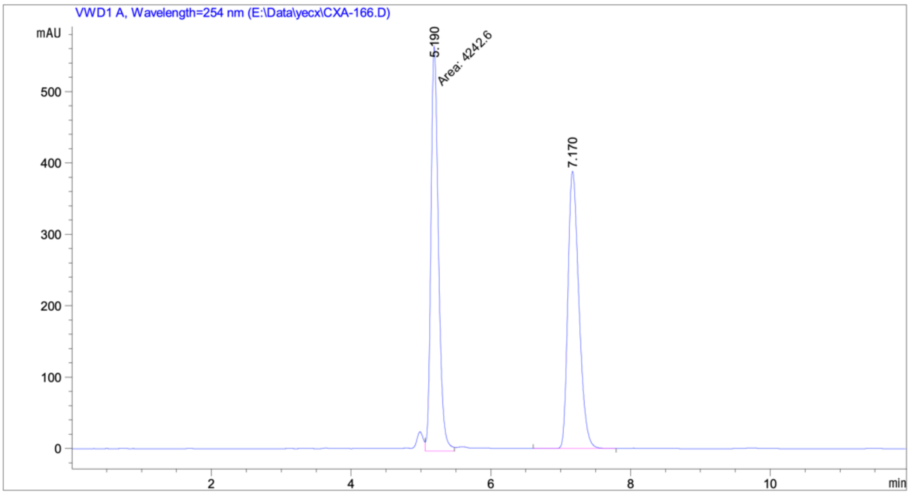

Peak #	RetTime [min]	Type	Width [min]	Area [mAU*s]	Height [mAU]	Area %
1	5.190	MM	0.1248	4242.59668	566.56812	49.9442
2	7.170	VB R	0.1698	4252.08057	388.16129	50.0558

Figure S3. Racemate of compound **3i** (CHIRALCEL OD-H, 25 °C, *i*PrOH/hexane = 2/98, 1.0 mL/min, 254 nm).

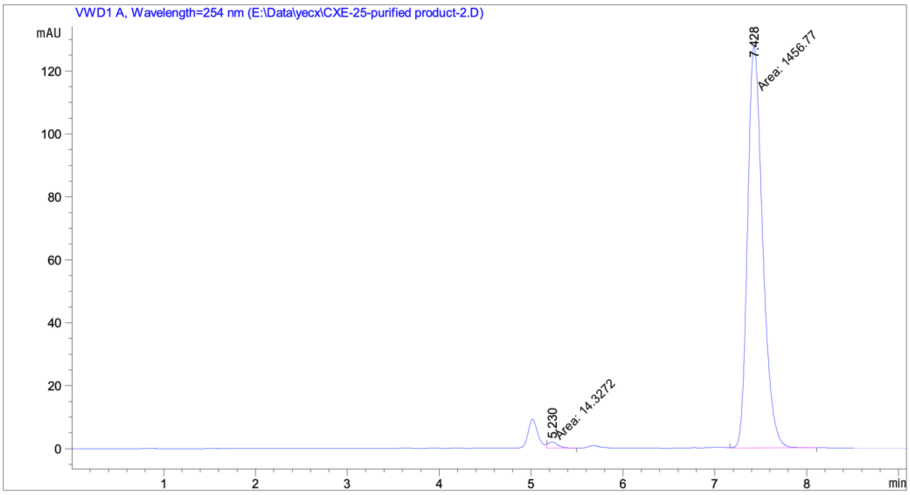

Peak #	RetTime [min]	Type	Width [min]	Area [mAU*s]	Height [mAU]	Area %
1	5.230	FM	0.1203	14.32720	1.98511	0.9739
2	7.428	FM	0.1904	1456.76965	127.53378	99.0261

Figure S4. Purified **3i** after reaction (CHIRALCEL OD-H, 25 °C, *i*PrOH/hexane = 2/98, 1.0 mL/min, 254 nm).

The *ee* of unreacted **1i** is 98%, and the *ee* of product **3i** is 98%. Based on the result, the selectivity factor (*S*) for kinetic resolution of **1i** is:

$$S = \frac{\ln((1-C)(1-ee(\mathbf{1i}))}{\ln((1-C)(1+ee(\mathbf{1i}))} = 458\text{, in which } C = \frac{ee(\mathbf{1i})}{ee(\mathbf{1i})+ee(\mathbf{3i})} = 0.5$$

7. Determination of the Absolute Configuration of a Derivative of *N*-Arylpyrrole 3j

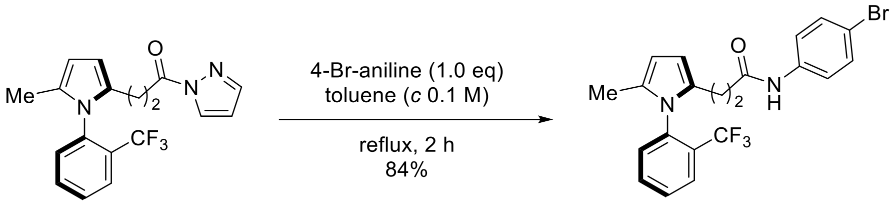

3j (>99% *ee*) **4j** (>99% *ee*)

In order to determine the absolute configuration of the products, *N*-arylpyrrole **3j** was converted into crystallizable secondary amide **4j**, and crystallization of which in EtOAc and *n*-pentane gave suitable single crystals. The absolute configuration of **4j** was determined by X-ray diffraction analysis (see Section 12). Accordingly, a solution of **3j** (29.2 mg, 0.084 mmol, 1.0 eq, >99% *ee*) and 4-bromoaniline (14.4 mg, 0.084 mmol, 1.0 eq) in toluene (0.8 mL, *c* 0.1 M) was heated to reflux for 2 hours. After being cooled to room temperature, solvent was removed under reduced pressure. The residue was purified by column chromatography (silica gel, eluted by EtOAc/*n*-hexane = 1/5) to give **4j** as a white solid (32.0 mg, 84% yield) with >99% *ee* [DAICEL CHIRALCEL OD-H column, Agilent HPLC 1260, *i*PrOH/hexane = 30/70 (v/v), 1.0 mL/min, 25 °C, 254 nm; t_1 = 5.1 min, t_2 = 12.6 min]: $[\alpha]_D^{25}$ = −42.8 (*c* 1.0, CH_2Cl_2, >99% *ee*); IR (film) ν_{max}: 3353, 2971, 1265, 950, 735 cm^{-1}; ^{1}H NMR (300 MHz, $CDCl_3$) δ 1.91 (s, 3H), 2.49−2.71 (m, 4H), 5.91−6.03 (m, 2H), 7.14−7.24 (m, 1H), 7.26−7.43 (m, 5H), 7.53−7.60 (m, 1H), 7.64 (td, *J* = 7.7, 1.5 Hz, 1H), 7.81 (dd, *J* = 7.7, 1.5 Hz, 1H); ^{13}C NMR (75 MHz, $CDCl_3$) δ 12.3, 22.5, 36.5, 105.0, 106.2, 116.7, 121.2, 122.9 (q, *J* = 273.6 Hz), 127.4 (q, *J* = 5.0 Hz), 129.17 (q, *J* = 30.3 Hz), 129.20, 130.6, 131.7, 131.9, 132.5, 133.0, 136.7 (q, *J* = 1.6 Hz), 136.9, 170.4; HRMS calcd for $[C_{21}H_{18}BrF_3N_2ONa]^+$ $(M + Na)^+$: 473.0447 and 475.0426; found: 473.0458 and 475.0438.

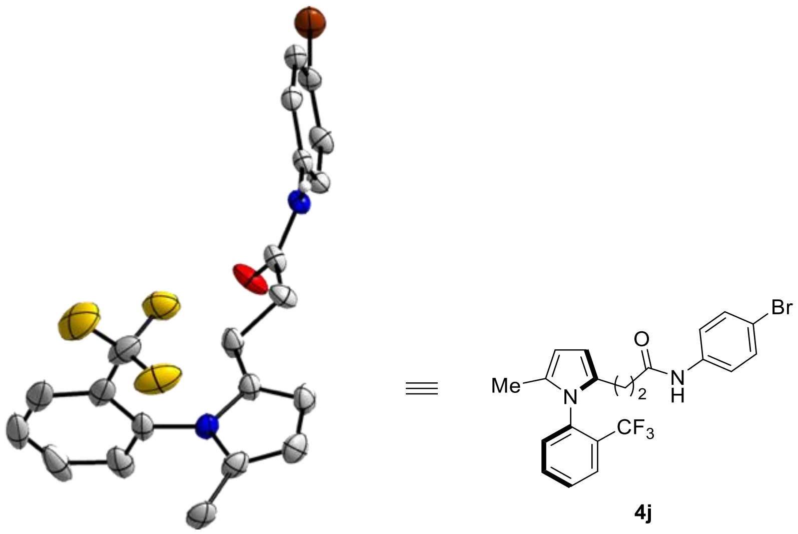

4j

Figure S5. Structure of **(*aR*)-4j** (CCDC: 1991853) determined by single-crystal XRD analysis.

8. Determination of the Relative Configuration of *N*-Arylpyrrole 3ad

The relative configuration of (*aR*,*2S*)-**3ad** was determined by NMR analysis of the preferred conformation of the side chain (by NOE interactions and the size of vicinal coupling constants) in combination with NOE interactions of the *i*Pr group with that side chain, which connects the axial chirality with the stereochemical information in the side chain.

Sample for NMR measurements was 10 mg of **3ad** dissolved in 0.6 mL of $CDCl_3$. Experiments were performed on a Bruker AVIII 600 MHz spectrometer equipped with a 5 mm TXI probe with z-gradient. Two-dimensional correlation spectra of ^1H, ^1H DQF-COSY, ^1H, ^{13}C HSQC and HMBC were recorded with standard pulse programs. NOESY and ROESY experiments were performed with mixing time of 1.5 s and 300 ms, respectively. Chemical shifts are referenced with the residue solvent signal.

The compound **3ad** showed a single set of neat NMR spectra. Based on the DQF-COSY, HSQC and HMBC spectra, unambiguous assignment to ^1H and ^{13}C signals were obtained (Figure S6 to S10).

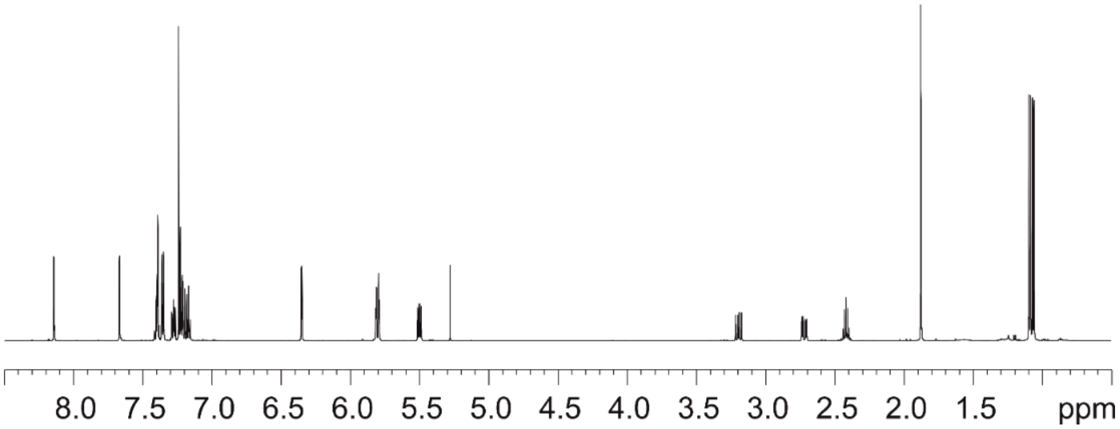

Figure S6. ^1H spectrum of **3ad** in $CDCl_3$ at 300 K.

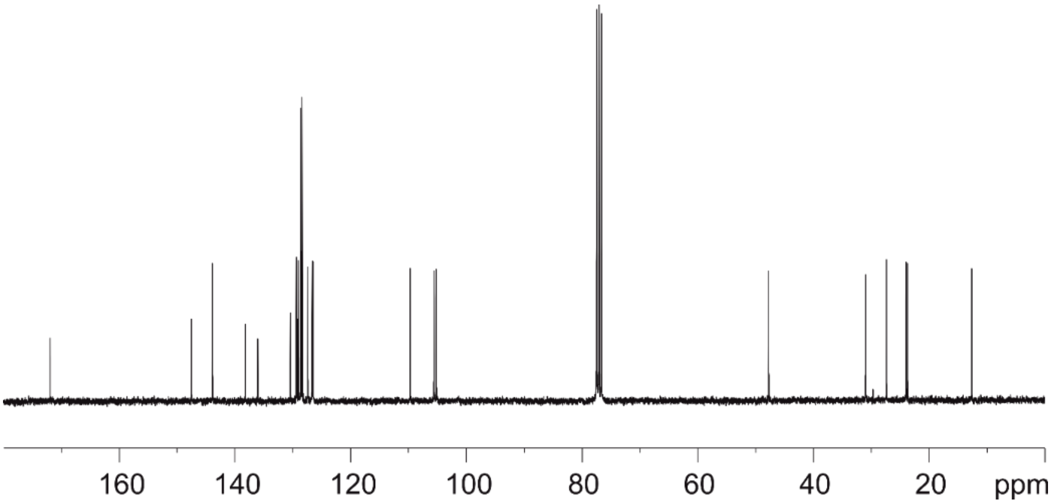

Figure S7. ^{13}C spectrum of **3ad** in $CDCl_3$ at 300 K.

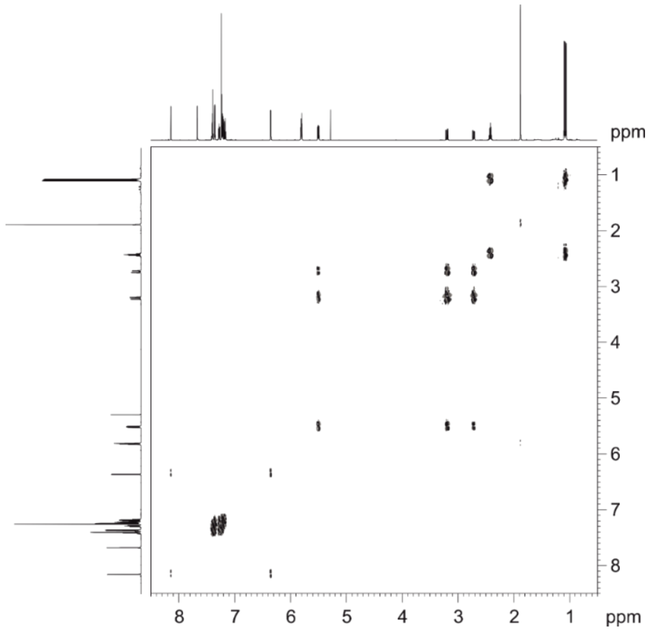

Figure S8. ^{1}H, ^{1}H DQF-COSY of **3ad** in $CDCl_3$ at 300 K.

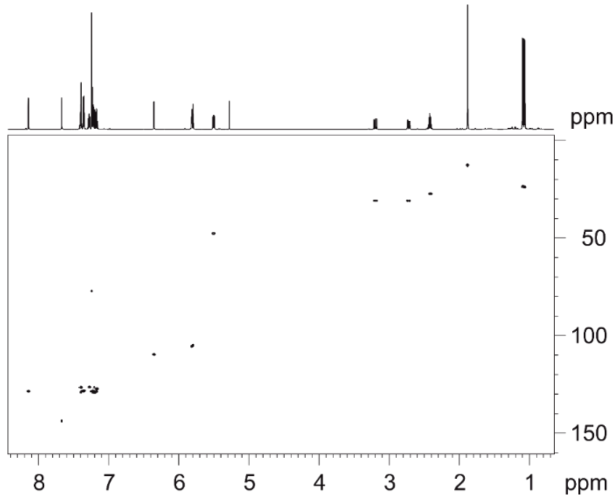

Figure S9. ^{1}H, ^{13}C HSQC spectrum of **3ad** in $CDCl_3$ at 300 K.

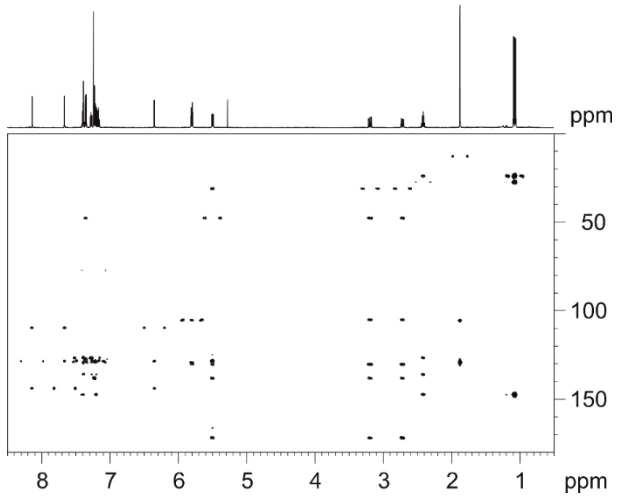

Figure S10. ^{1}H, ^{13}C HMBC spectrum of **3ad** in $CDCl_3$ at 300 K.

With the defined orientation of the chiral axis between *N*-1 and *C*-6, the two hydrogen atoms at position *C*-15 are diastereotopic and the corresponding ^{1}H signals at 3.194 ppm (dd: 15.5, 10.2 Hz) and 2.719 ppm (dd: 15.9, 5.4 Hz) were assigned, respectively. Furthermore, the hydrogen atom at position *C*-16 showed a ^{1}H signal at 5.500 ppm (dd: 9.9, 5.5 Hz). An unambiguous stereospecific assignment to H-15^{proR}, H-15^{proS}, and *H*-16 was fulfilled by analyzing the intramolecular NOE interactions (Figure S11). Therefore, NOE cross peak was observed between 2.719 − 1.065 ppm, which was assigned to be a correlation between H-15^{proS} and *H*-13’ (one of the CH_3 groups contained in *i*Pr), while there was no such NOE cross peak between H-15^{proR} and H-13’. Stronger NOE contact was observed between *H*-16 − H-15^{proS} than *H*-16 − H-15^{proR}, whereas those between *H*-22, *H*-26 − H-15^{porR} was observed to be stronger than *H*-22, *H*-26 − H-15^{proS}. Accordingly, the absolute configuration of *C*-16 was determined as *S*. The key NOE contacts which lead to the determined stereo structure are presented in Table S1.

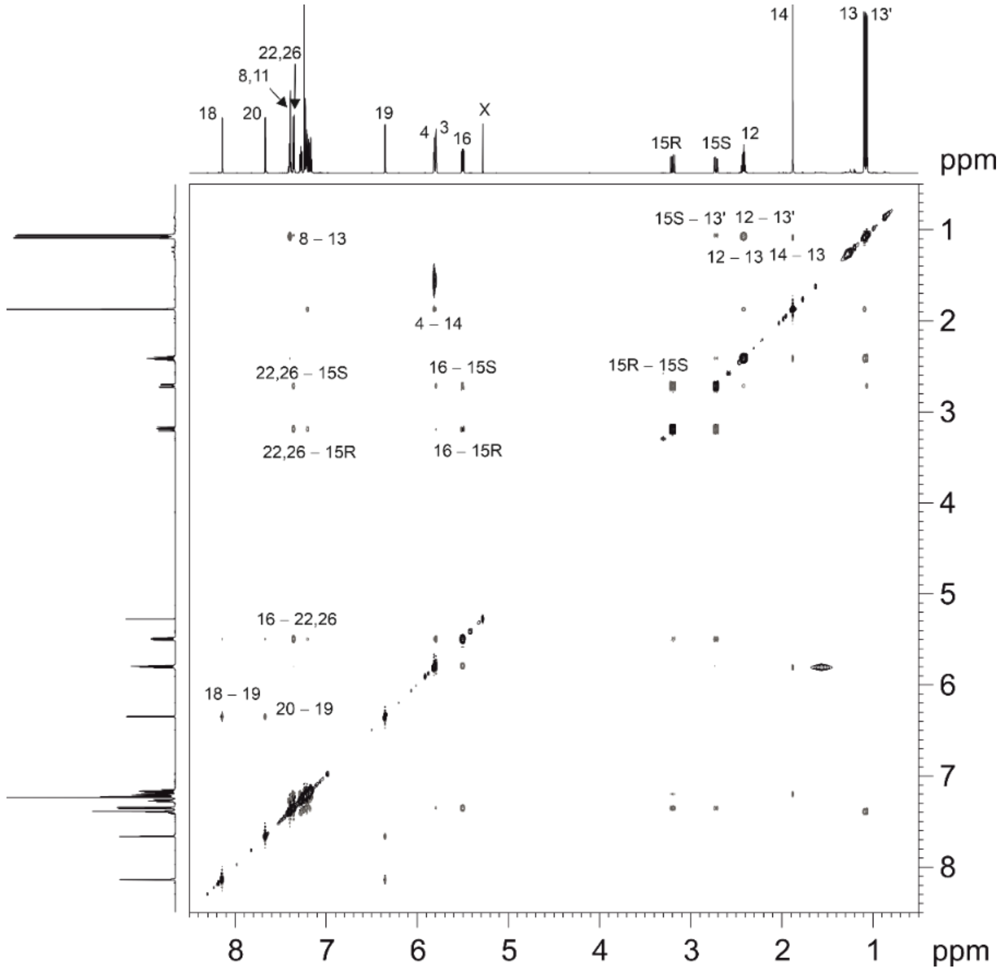

Figure S11. NOESY spectrum of **3ad** in $CDCl_3$ at 300 K, mixing time 1.5 s.

Table S1. Key NOESY cross peaks of **3ad** in $CDCl_3$.

No.	Positions	Strength	No.	Positions	Strength
1	*H*-3 – *H*-15^{proS}	medium	5	*H*-15^{proR} – *H*-16	weak
2	*H*-3 – *H*-15^{proR}	weak	6	*H*-15^{proS} – *H*-16	medium
3	CH_3-13' – *H*-15^{proR}	not detected	7	*H*-15^{proR} – *H*-22, *H*-26	medium
4	CH_3-13' – *H*-15^{proS}	medium	8	*H*-15^{proS} – *H*-22, *H*-26	weak

The vicinal coupling constants ^3J are dependent on the corresponding torsion angle and reveals conformation of the molecule. According to Karplus relation, $^3J_g \approx 5$ Hz and $^3J_t \approx 10$ Hz for gauche and trans conformation, respectively. The observed vicinal coupling constants between *H*-15^{proR} – *H*-16 and *H*-15^{proS} – *H*-16 were 10.2 and 5.4 Hz and lead to a conformation between positions *C*-15 and *C*-16 as shown in the sketch in Figure S12.

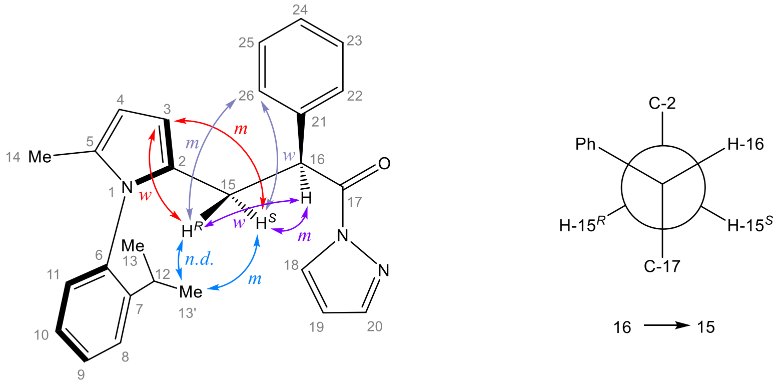

Figure S12. Structural scheme of **3ad**, with partial conformation sketch.

9. Configurational Stability Investigations of 3g, 3q and 3r

The configurational stability of axially chiral *N*-arylpyrroles (*aR*)-**3g**, (*aR*)-**3q** and (*aR*)-**3r** was studied by heating their toluene solution (*c* 0.05 M) under N_2 at indicated temperature for indicated time. Then the *ee* values were determined by HPLC analysis using a chiral stationary phase. The results were summarized in Table S2.

Table S2. Configurational stability investigations of **3g**, **3q** and **3r** in toluene.

Compound	Temperature (°C)	Time (h)	*ee* (%)
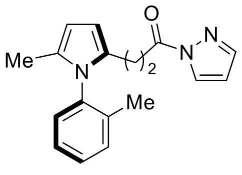 **3g** (97% *ee*)	80	18	97
	120	18	96
	160	18	94
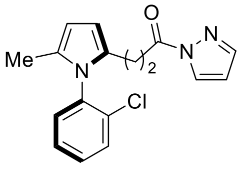 **3q** (91% *ee*)	80	18	91
	120	18	89
	160	18	64
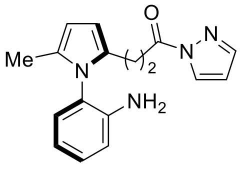 **3r** (93% *ee*)	80	18	93
	120	18	92 (partially decomposed)
	160	18	- (totally decomposed)

10. Gram-Scale Synthesis and Derivations of *N*-Arylpyrrole 3o

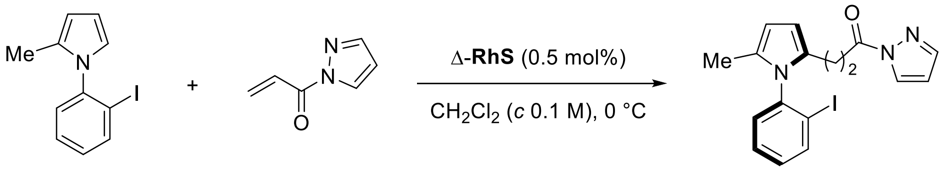

1o (3.04 mmol) **2a** (4.56 mmol, 1.5 eq) **3o** 88% yield, 98% *ee*

To a 100 mL round bottom flask was added Δ-**RhS** (13 mg, 15 μmol, 0.5 mol%), *N*-arylpyrrole **1o** (860 mg, 3.04 mmol, 1.0 eq), acrylpyrazole **2a** (557 mg, 4.56 mmol, 1.5 eq) and CH_2Cl_2 (30 mL, *c* 0.1 M). The reaction mixture was stirred at 0 °C for 4 hours until full conversion of **1o** was reached monitored by TLC. Then the reaction was quenched by addition of CH_3CN (10 mL) before removal of solvent under reduced pressure. The residue was purified by column chromatography (silica gel, eluted by EtOAc/*n*-hexane = 1/10) to afford desired products **3o** (1.08 g, 88% yield) with 98% *ee*.

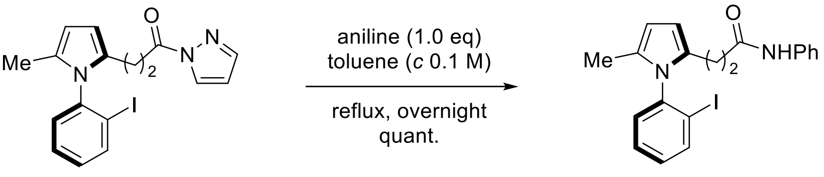

3o (98% *ee*) **4o** (98% *ee*)

A solution of **3o** (15.4 mg, 0.038 mmol, 1.0 eq, 98% *ee*) and aniline (3.5 μL, 0.038 mmol, 1.0 eq) in toluene (0.4 mL, *c* 0.1 M) was heated to reflux for overnight. After being cooled to room temperature, the solvent was removed under reduced pressure. The residue was purified by column chromatography (silica gel, eluted by EtOAc/*n*-hexane = 1/4) to give **4o** as a yellow oil (16.6 mg, quantitative yield) with 98% *ee* [DAICEL CHIRALCEL OD-H column, Agilent HPLC 1260, *i*PrOH/hexane = 20/80 (v/v), 1.0 mL/min, 25 °C, 254 nm; t_1 = 10.1 min, t_2 = 16.5 min]: $[\alpha]_D^{25}$ = −41.2 (*c* 1.0, CH_2Cl_2, 98% *ee*); IR (film) ν_{max}: 3350, 2970, 1160, 950, 736 cm^{-1}; ^1H NMR (300 MHz, $CDCl_3$) δ 1.97 (s, 3H), 2.47−2.62 (m, 2H), 2.62−2.78 (m, 2H), 5.89−6.10 (m, 2H), 6.99−7.11 (m, 1H), 7.15 (td, *J* = 7.8, 1.4 Hz, 1H), 7.20−7.33 (m, 3H), 7.33−7.57 (m, 4H), 7.95 (dd, *J* = 7.9, 1.0 Hz, 1H); ^{13}C NMR (75 MHz, $CDCl_3$) δ 12.7, 22.9, 36.8, 100.5, 105.6, 106.2, 119.7, 124.1, 128.9, 129.0, 129.4, 129.9, 130.2, 130.6, 137.9, 139.7, 141.5, 170.4; HRMS calcd for $[C_{20}H_{19}IN_2ONa]^+$ $(M + Na)^+$: 453.0434; found: 453.0445.

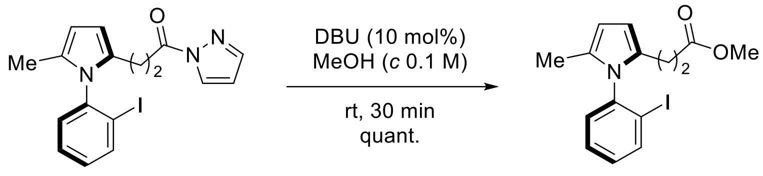

3o (98% *ee*) **5o** (98% *ee*)

A solution of **3o** (17.0 mg, 0.04 mmol, 1.0 eq, 98% *ee*) and DBU (0.6 μL, 0.004 mmol, 10 mol%) in methanol (0.4 mL, *c* 0.1 M) was stirred at room temperature for 30 min before removal of solvent under reduced pressure. The residue was purified by column chromatography (silica gel, eluted by EtOAc/*n*-hexane = 1/12) to give **5o** as a colorless oil (15.6 mg, quantitative yield) with 98% *ee* [DAICEL CHIRALCEL OD-H column, Agilent HPLC 1260, *i*PrOH/hexane = 20/80 (v/v), 1.0 mL/min, 25 °C, 254 nm; t_1 = 10.0 min, t_2 = 13.8 min]: $[\alpha]_D^{25}$ = −60.3 (*c* 1.0, CH_2Cl_2, 98% *ee*); IR (film) ν_{max}: 2918, 1734, 1476, 762 cm^{-1}; ^1H NMR (300 MHz, $CDCl_3$) δ 1.94 (s, 3H), 2.41−2.68 (m, 4H), 3.64 (s, 3H), 5.84−6.03 (m, 2H), 7.16 (td, *J* = 7.9, 1.5 Hz, 1H), 7.30 (dd, *J* = 7.8, 1.4 Hz, 1H), 7.47 (td, *J* = 7.8, 1.4 Hz, 1H), 7.96 (dd, *J* = 8.0, 1.2 Hz, 1H); ^{13}C NMR (75 MHz, $CDCl_3$) δ 12.7, 22.4, 33.3, 51.5, 100.6, 105.0, 106.0, 128.6, 129.2, 129.9, 130.1, 130.8, 139.7, 141.7, 173.2; HRMS calcd for $[C_{15}H_{16}INO_2Na]^+$ $(M + Na)^+$: 392.0118; found: 392.0127.

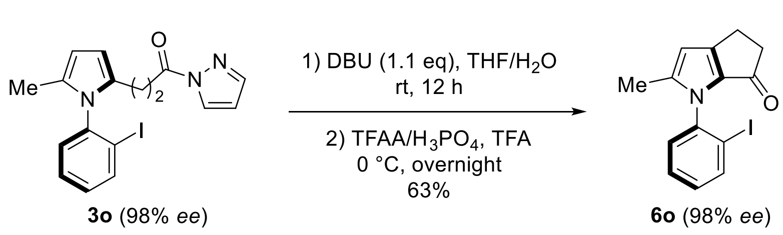

3o (98% *ee*) **6o** (98% *ee*)

A solution of **3o** (1.0 eq) and DBU (1.1 eq) in a mixed solvent of THF/H_2O (v/v = 5/1, *c* 0.1 M) was stirred at room temperature until TLC showed that **3o** disappeared (after about 12 hours). Then the

solvent was removed under reduced pressure to give the hydrolysis intermediate. The residue was used in the next step without purification.

Preparation of racemic **6o**: To the residue from the first step that started from racemic **3o** (40.5 mg, 0.1 mmol) was added toluene (0.5 mL, *c* 0.2 M) and PPA (100 mg).[3] The mixture was stirred at 100 °C for overnight. After being cooled to room temperature, the reaction was quenched by addition of 10 mL H_2O and the mixture was extracted with EtOAc (3×10 mL). The combined organic layer was washed with brine and dried over anhydrous sodium sulfate. After filtration, the solvent was evaporated under reduced pressure, and the residue was purified by column chromatography (silica gel, eluted by EtOAc/*n*-hexane = 1/2) to give racemic **6o** as a white solid (31.8 mg, 94% yield for two steps). However, when non-racemic **3o** was subjected to these reaction conditions, partial racemization of product happened due to the configurational instability of intermediate **S11** under high temperature.

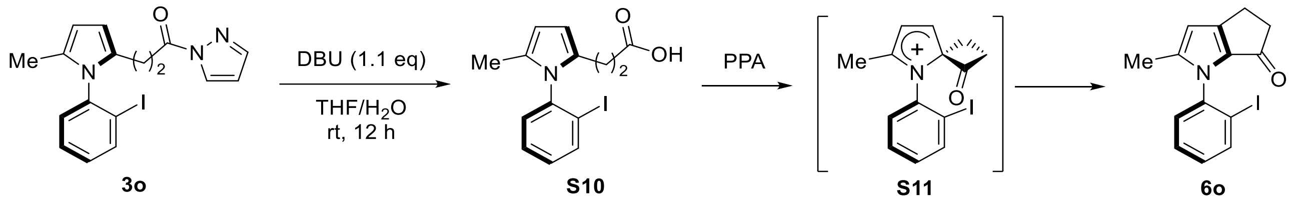

Figure S13. Reaction mechanism for the intramolecular Friedel-Crafts acylation towards **6o**.

Preparation of non-racemic **6o**: The residue from the first step that started from non-racemic **3o** (24.0 mg, 0.059 mmol, 98% *ee*) was dissolved in TFA (0.2 mL, *c* 0.3 M). To the solution at 0 °C was added 80 μL supernatant of a mixture of TFAA/H_3PO_4 (85%) (v/v = 4/1, mixed and stirred at room temperature for 30 min, during which white precipitate formed).[4] The reaction mixture was stirred at 0 °C for overnight and was then quenched by addition of 2 mL methanol. The solvent was evaporated under reduced pressure, and the residue was purified by column chromatography (silica gel, eluted by EtOAc/*n*-hexane = 1/2) to give **6o** as a white solid (12.6 mg, 63% yield for two steps) with 98% *ee* [DAICEL CHIRALPAK IG column, Agilent HPLC 1260, *i*PrOH/hexane = 30/70 (v/v), 1.0 mL/min, 25 °C, 254 nm; t_1 = 17.6 min, t_2 = 19.7 min]: $[\alpha]_D^{25}$ = −62.3 (*c* 1.0, CH_2Cl_2, 98% *ee*); IR (film) ν_{max}: 2970, 1671, 1265, 950, 734 cm^{-1}; ^1H NMR (300 MHz, $CDCl_3$) δ 2.00 (d, *J* = 1.0 Hz, 3H), 2.60 (ddd, *J* = 16.7, 5.6, 3.7 Hz, 1H), 2.72 (ddd, *J* = 16.7, 6.0, 3.6 Hz, 1H), 2.77−2.95 (m, 2H), 6.17 (q, *J* = 1.0 Hz, 1H), 7.21 (td, *J* = 7.8, 1.6 Hz, 1H), 7.32 (dd, *J* = 7.8, 1.6 Hz, 1H), 7.50 (td, *J* = 7.7, 1.4 Hz, 1H), 7.98 (dd, *J* = 8.0, 1.3 Hz, 1H); ^{13}C NMR (75 MHz, $CDCl_3$) δ 13.0, 21.0, 40.8, 98.3, 100.8, 126.6, 128.8, 129.5, 130.9, 137.5, 140.0, 140.1, 160.1, 196.4; HRMS calcd for $[C_{14}H_{13}INO]^+$ $(M + H)^+$: 338.0036; found: 338.0044.

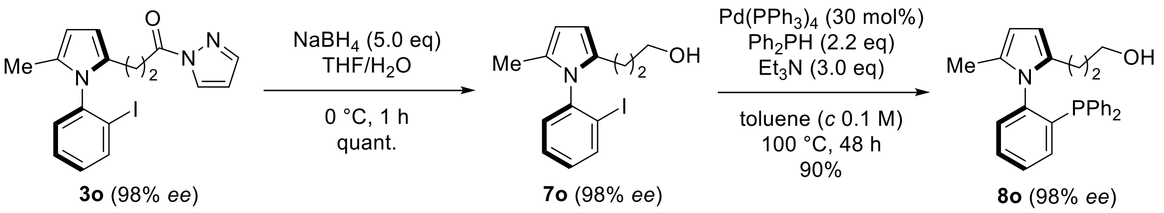

3o (98% *ee*) **7o** (98% *ee*) **8o** (98% *ee*)

To a solution of **3o** (60.8 mg, 0.15 mmol, 1.0 eq, 98% *ee*) in a mixed solvent of THF/H_2O (v/v = 4/1) (1.5 mL, *c* 0.1 M) at 0 °C was added $NaBH_4$ (28.5 mg, 0.75 mmol, 5.0 eq) in one portion. The reaction mixture was stirred at 0 °C for 1 hour and then was quenched by addition of 10 mL saturated aqueous $NaHCO_3$ solution. The mixture was extracted with EtOAc (3×20 mL). The combined organic layer was washed with brine and dried over anhydrous sodium sulfate. After filtration, the solvent was evaporated under reduced pressure, and the residue was purified by column chromatography (silica gel, eluted by EtOAc/*n*-hexane = 1/2) to give **7o** as a colorless oil (51.0 mg, quantitative yield) with 98% *ee* [DAICEL CHIRALCEL OD-H column, Agilent HPLC 1260, *i*PrOH/hexane = 20/80 (v/v), 1.0 mL/min, 25 °C, 254 nm; t_1 = 5.2 min, t_2 = 6.1 min]: $[\alpha]_D^{25}$ = −24.3 (*c* 1.0, CH_2Cl_2, 98% *ee*); IR (film) ν_{max}: 3337, 2919, 1475, 758 cm^{-1}; ^1H NMR (300 MHz, $CDCl_3$) δ 1.47−1.69 (br, 1H), 1.70−1.86 (m, 2H), 1.95 (s, 3H), 2.21−2.44 (m, 2H), 3.64 (t, *J* = 6.3 Hz, 2H), 5.89−6.03 (m, 2H), 7.15 (td, *J* = 7.8, 1.5 Hz, 1H), 7.29 (dd, *J* = 7.8, 1.4 Hz, 1H), 7.46 (td, *J* = 7.7, 1.0 Hz, 1H), 7.96 (dd, *J* = 8.0, 1.0 Hz, 1H); ^{13}C NMR (75 MHz, $CDCl_3$) δ 12.7, 23.5, 31.7, 62.6, 100.7, 105.1, 106.0, 128.4, 129.1, 130.0 (2C), 132.0, 139.6, 141.9; HRMS calcd for $[C_{14}H_{17}INO]^+$ $(M + H)^+$: 342.0349; found: 342.0358.

To a mixture of **7o** (33.8 mg, 0.1 mmol, 1.0 eq, 98% *ee*) and Pd(PPh_3)$_4$ (34.7 mg, 0.03 mmol, 30 mol%) in toluene (1 mL, *c* 0.1 M) under N_2 was added Ph_2PH (38 μL, 0.22 mmol, 2.2 eq) and Et_3N (41 μL, 0.3 mmol, 3.0 eq). The reaction mixture was stirred at 100 °C for 48 hours. Then the solvent was evaporated under reduced pressure, and the residue was purified by column chromatography (silica gel, eluted by EtOAc/*n*-hexane = 1/2) to give **8o** as a yellow oil (35.2 mg, 88% yield) with 98% *ee* [DAICEL CHIRALCEL OD-H column, Agilent HPLC 1260, *i*PrOH/hexane = 20/80 (v/v), 1.0 mL/min, 25 °C, 254 nm; t_1 = 4.3 min, t_2 = 5.5 min]: $[\alpha]_D^{25}$ = −53.5 (*c* 1.0, CH_2Cl_2, 98% *ee*); IR (film) ν_{max}: 3330, 3054, 2919, 1498, 1472, 741, 694 cm^{-1}; ^1H NMR (300 MHz, $CDCl_3$) δ 1.50−1.63 (m, 1H), 1.63−1.73 (m, 1H), 1.75 (s, 3H), 2.01 (ddd, *J* = 15.7, 8.7, 7.0 Hz, 1H), 2.16 (ddd, *J* = 15.7, 9.0, 5.8 Hz, 1H), 3.50 (t, *J* = 6.3 Hz, 2H), 5.87−5.96 (m, 2H), 7.16−7.36 (m, 12H), 7.39 (td, *J* = 7.7, 1.6 Hz, 1H), 7.46 (td, *J* = 7.4, 1.7 Hz, 1H); ^{13}C NMR (75 MHz, $CDCl_3$) δ 12.7 (d, *J* = 4.1 Hz), 23.6 (d, *J* = 4.1 Hz), 31.2, 62.5, 104.4, 105.6, 128.3, 128.4 (d, *J* = 1.1 Hz), 128.5, 128.6, 128.7 (d, *J* = 3.4 Hz), 129.0, 129.6 (d, *J* = 3.1 Hz), 130.0, 132.9, 133.8 (d, *J* = 8.7 Hz), 134.1 (d, *J* = 8.6 Hz), 134.9 (d, *J* = 2.2 Hz), 136.1 (d, *J* = 11.4 Hz), 136.2 (d, *J* = 11.4 Hz), 139.1 (d, *J* = 14.9 Hz), 143.4 (d, *J* = 26.1 Hz); HRMS calcd for $[C_{26}H_{26}NOPNa]^+$ $(M + Na)^+$: 422.1644; found: 422.1655.

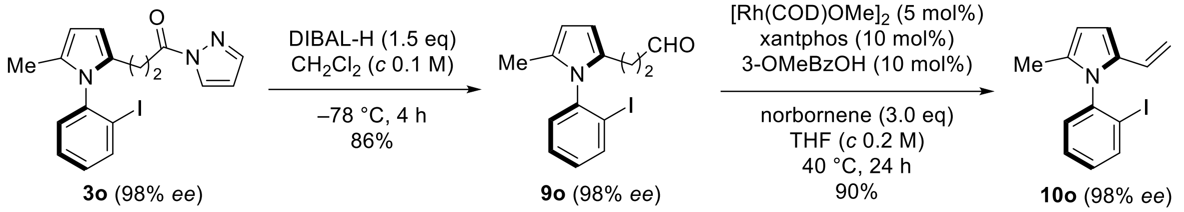

A solution of **3o** (113 mg, 0.28 mmol, 1.0 eq, 98% *ee*) in CH_2Cl_2 (3 mL, *c* 0.1 M) was cooled to −78 °C under N_2. DIBAL-H solution (1.0 M in heptane, 0.42 mL, 0.42 mmol, 1.5 eq) was added dropwise. The reaction mixture was stirred at −78 °C for 4 hours before being quenched by addition of 10 mL saturated aqueous potassium sodium tartrate. The mixture was warmed to room temperature and was extracted with Et_2O (5×20 mL) and the combined organic layer was washed with brine and dried over anhydrous sodium sulfate. After filtration, the solvent was evaporated under reduced pressure, and the residue was purified by column chromatography (silica gel, eluted by EtOAc/*n*-hexane = 1/8) to give **9o** as a colorless oil (81.4 mg, 86% yield) with 98% *ee* [DAICEL CHIRALPAK IG column, Agilent HPLC 1260, MTBE/hexane = 25/75 (v/v), 1.0 mL/min, 25 °C, 254 nm; t_1 = 10.3 min, t_2 = 10.8 min]: $[\alpha]_D^{25}$ = −31.4 (*c* 0.5, CH_2Cl_2, 98% *ee*); IR (film) ν_{max}: 2916, 2723, 1720, 1475, 755 cm^{-1}; ^1H NMR (300 MHz, $CDCl_3$) δ 1.95 (s, 3H), 2.51−2.62 (m, 2H), 2.64−2.73 (m, 2H), 5.90−5.94 (m, 1H), 5.94−5.99 (m, 1H), 7.17 (td, *J* = 7.8, 1.7 Hz, 1H), 7.30 (dd, *J* = 7.8, 1.6 Hz, 1H), 7.48 (td, *J* = 7.7, 1.4 Hz, 1H), 7.97 (dd, *J* = 8.0, 1.4 Hz, 1H), 9.74 (t, *J* = 1.4 Hz, 1H); ^{13}C NMR (75 MHz, $CDCl_3$) δ 12.7, 19.8, 42.6, 100.6, 105.4, 106.1, 128.8, 129.3, 129.8, 130.2, 130.4, 139.7, 141.6, 201.8; HRMS calcd for $[C_{14}H_{14}INONa]^+$ $(M + Na)^+$: 362.0012; found: 362.0023.

To a 10 mL Schlenk tube under N_2 was added **9o** (61.0 mg, 0.18 mmol, 1.0 eq, 98% *ee*), $[Rh(COD)OMe]_2$ (4.4 mg, 0.009 mmol, 5.0 mol%), xantphos (10.4 mg, 0.018 mmol, 10 mol%) and 3-methoxybenzoic acid (2.7 mg, 0.018 mmol, 10 mol%). THF (0.4 mL, *c* 0.5 M) was added, and the mixture was stirred for 10 min before addition of norbornene (50.9 mg, 0.54 mmol, 3.0 eq) in one portion. The reaction mixture was stirred at 40 °C for 24 hours. After a full conversion of **9o** was reached monitored by TLC, the reaction mixture was diluted with 10 mL *n*-hexane and filtered through a syringe filter, and the filtrate was concentrated under reduced pressure to remove the solvent as well as excess norbornene. The residue was purified by column chromatography (5 cm silica gel prepared by 1% Et_3N in *n*-hexane before loading the sample, eluted by 1% Et_3N in *n*-hexane. *Caution: **10o** is fairly unstable on silica column, the chromatography should be completed within 5 min to avoid significant decomposition of product*) to give **10o** as a colorless oil (50.0 mg, 90 % yield) with 98% *ee* [DAICEL CHIRALCEL OD-H column, Agilent HPLC 1260, *i*PrOH/hexane = 2/98 (v/v), 1.0 mL/min, 25 °C, 254 nm; t_1 = 5.0 min, t_2 = 5.6 min]: $[\alpha]_D^{25}$ = −54.0 (*c* 0.5, CH_2Cl_2, 98% *ee*); IR (film) ν_{max}: 2917, 1474, 1019, 758, 731 cm^{-1}; ^1H NMR (300 MHz, $CDCl_3$) δ 1.98 (s, 3H), 4.76 (dd, *J* = 11.5, 1.3 Hz, 1H), 5.17 (dd, *J* = 17.7, 1.3 Hz, 1H), 6.02 (dd, *J* = 17.7, 11.5 Hz, 1H), 6.04 (d, *J* = 3.5 Hz, 1H), 6.43 (d, *J* = 3.5 Hz, 1H),

7.17 (td, *J* = 7.8, 1.6 Hz, 1H), 7.30 (dd, *J* = 7.8, 1.6 Hz, 1H), 7.47 (dd, *J* = 7.6, 1.3 Hz, 1H), 7.96 (dd, *J* = 8.0, 1.3 Hz, 1H); ^{13}C NMR (75 MHz, $CDCl_3$) δ 12.7, 100.4, 106.4, 107.6, 109.1, 126.2, 129.1, 130.0, 130.1, 130.4, 131.7, 139.6, 141.6; HRMS calcd for $[C_{13}H_{13}IN]^+$ $(M + H)^+$: 310.0087; found: 310.0096.

11. Computational Studies of the Transition States

Computational Methods: Density functional theory (DFT) computations were performed in Gaussian 16, Revision C.01.[5] Molecular geometries were optimized using the B3LYP[6] functional augmented with Grimme's D3[7] empirical dispersion term with Becke–Johnson damping.[8] The LANL2DZ basis set (including the effective core potential)[9] was used for Rh, and the 6-31G(d) basis set was used for all other atoms. Frequency calculations were performed at the same level of theory as that used for geometry optimization to characterize the stationary points as either minima (no imaginary frequencies) or first-order saddle points (one imaginary frequency). Intrinsic Reaction Coordinate (IRC) calculations were performed to confirm that the first-order saddle points found were real transition states connecting the reactants and the products. Thermal contributions to free energies were calculated from vibrational frequencies using the quasi-rigid rotor-harmonic oscillator (RRHO) approach of Grimme.[10] Single point energies were calculated with the M06[11] functional augmented with Grimme's D3 empirical dispersion term, with the SDD[12] basis set for Rh and the 6-311++G(d,p) basis set for all other atoms. Solvation effects were incorporated using the SMD[13] model with dichloromethane as the solvent. Molecular structure visualizations were obtained using CYLview.[14] Monte Carlo conformational searches were performed with the Merck molecular force field (MMFF) implemented in Spartan '18[15] to ensure that the lowest energy conformations are presented.

Calculated Energies: The calculated free energy diagram of the Rh-catalyzed atroposelective reaction between **1a** and **2a** is shown below. Bidentate coordination of **2a** to replace two MeCN ligands on Δ-**RhS** yields Rh complex **S12** in an overall exergonic process. Complex **S12** subsequently forms pre-reaction complex **S13** with *N*-arylpyrrole **1a**. The C–C bond forming TS, **TS-1a**, proceeds with a barrier of 14.5 kcal/mol to give rise to post-reaction complex **S14**. Product (*aR*)-**3aa** is furnished after dissociation (possibly ligand replacement by another equivalent of **2a**) and tautomerization.

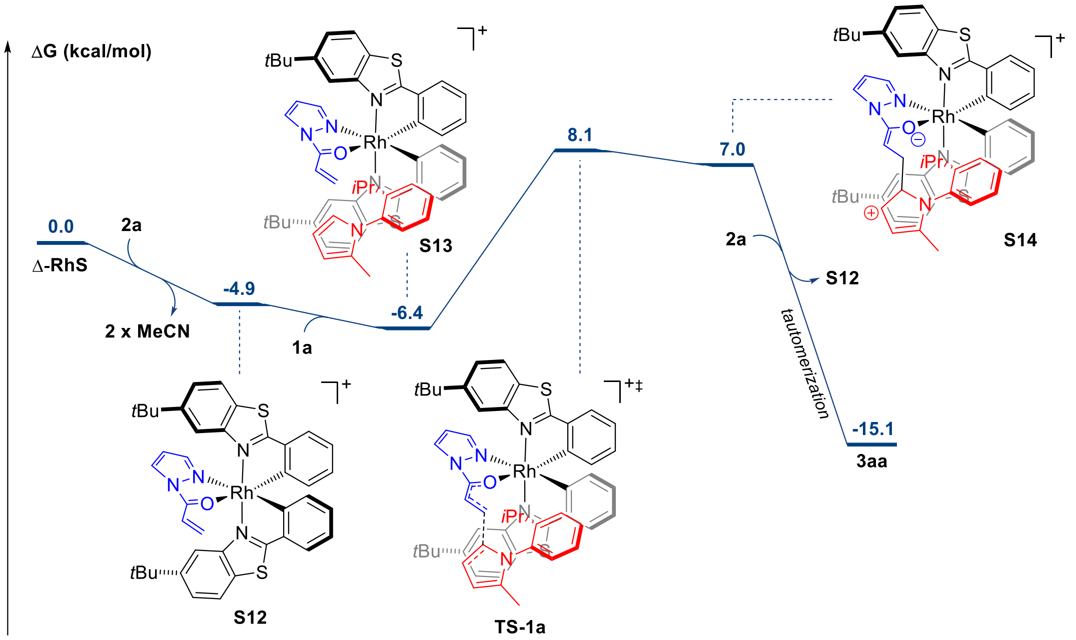

Figure S14. Calculated free energy diagram of the reaction between **1a** and **2a** catalyzed by **Δ-RhS**.

Table S3. Calculated energies in Hartrees.

Structure	E(CH_2Cl_2)	ΔG	G(CH_2Cl_2) [= E(CH_2Cl_2) + ΔG]
**1a**	-598.181533	0.234655	-597.946878
**2a**	-416.776887	0.081565	-416.695322
**3aa**	-1015.009703	0.343402	-1014.666301
**S12**	-2747.385031	0.617249	-2746.767782
**S13**	-3345.596985	0.879881	-3344.717104
**S14**	-3345.601986	0.883155	-3344.718831
**MeCN**	-132.703433	0.022659	-132.680774
**Δ-RhS**	-2596.018450	0.592217	-2595.426233
**TS-1a**	-3345.576073	0.881999	-3344.694074
**TS-1b**	-3345.570399	0.879707	-3344.690692

Cartesian Coordinates of Computed Structures:

1a

C	2.96238300	-1.37410700	-0.02616900
C	2.52072000	-0.05398200	0.04382800
C	1.15981400	0.26948200	-0.03147400
C	0.25052400	-0.79092400	-0.18665500
C	0.68491200	-2.11634900	-0.25445000

C	2.04338600	-2.41314300	-0.17583100
H	4.02516900	-1.59075200	0.03602700
H	3.24952200	0.74332800	0.15585200
H	-0.05676200	-2.90069000	-0.36917600
H	2.38027100	-3.44410300	-0.22959800
C	0.68741500	1.71381200	0.00023700
C	1.33811400	2.52287000	1.13215900
H	0.89874600	3.52538300	1.17857900
H	1.18894700	2.03948900	2.10391100
H	2.41668400	2.64490200	0.98025100
C	0.92272700	2.38054100	-1.36698900
H	1.99055900	2.39954500	-1.61587900
H	0.39824600	1.83743100	-2.15962800
H	0.55573600	3.41331700	-1.36020900
N	-1.14919200	-0.52458700	-0.28089300
C	-1.99826200	-0.29793000	0.79242700
C	-1.84138100	-0.33868900	-1.46401400
C	-3.23566500	0.02394400	0.27170300
C	-3.13773200	-0.00104300	-1.15018800
H	-1.33852100	-0.46903100	-2.41079700
H	-4.11833700	0.24386600	0.85770300
H	-3.93024800	0.19666200	-1.85903000
H	-0.39212200	1.70565700	0.17717500
C	-1.52513100	-0.41449600	2.20413200
H	-2.36288800	-0.24647700	2.88635200
H	-1.10683400	-1.40592900	2.41810500
H	-0.74478200	0.31960400	2.44433300

2a

C	-3.17223200	-0.56839300	0.00004800
H	-3.58460100	0.43652000	0.00002700
H	-3.86630300	-1.40296400	0.00018900
C	-1.85013800	-0.76009100	-0.00002500
H	-1.39673400	-1.74333300	0.00004500

C	-0.94645100	0.41178100	-0.00001600
O	-1.30031500	1.57639200	0.00000300
N	0.43979900	0.11204400	-0.00001400
C	1.45082300	1.04155600	-0.00007000
C	2.23128200	-1.03107200	0.00001300
C	2.62685900	0.33777300	0.00011300
H	1.22138600	2.09515600	-0.00013000
H	2.86152900	-1.91107000	0.00002900
H	3.62989800	0.73827500	0.00024600
N	0.91684200	-1.17105000	-0.00010200

3aa

C	-0.41927000	-0.44986500	-1.98746800
H	-0.71832000	-1.22154500	-2.70359000
H	0.55029400	-0.05647400	-2.31272500
C	-1.45160600	0.71312200	-2.06426400
H	-1.13156500	1.54162800	-1.43382200
C	-2.80581100	0.19877500	-1.64580000
O	-3.53875200	-0.45133600	-2.36314900
N	-3.18854600	0.43309900	-0.30265300
C	-4.27723600	-0.13356900	0.31392500
C	-3.06146900	1.06170000	1.72454400
C	-4.22940000	0.25307000	1.62725100
H	-4.95493600	-0.75650600	-0.24733500
H	-2.66393800	1.56489500	2.59651400
H	-4.92386300	-0.00605800	2.41274100
N	-2.43484000	1.17245700	0.56646700
C	3.10725400	2.70964300	-0.05570100
C	3.53927400	1.40088900	-0.25989100
C	2.67059100	0.31072300	-0.11438800
C	1.33888700	0.58661400	0.23884300
C	0.89629600	1.89581400	0.44749100
C	1.78125900	2.96107400	0.30050400
H	3.80605000	3.53293500	-0.17560100

H	4.57405800	1.21954300	-0.53727000
H	-0.14665700	2.04802300	0.70504100
H	1.43777900	3.97908700	0.45972300
C	3.15827700	-1.11086900	-0.34123300
C	4.32389700	-1.46458200	0.59710900
H	4.61805000	-2.51134900	0.46109400
H	5.20409200	-0.84230500	0.39802500
H	4.04498200	-1.32271800	1.64668400
C	3.54769600	-1.33473800	-1.81239600
H	2.70461200	-1.12922800	-2.48018900
H	3.86221700	-2.37233100	-1.97201600
N	0.40736600	-0.48533600	0.39697300
C	0.19665600	-1.18273800	1.57663800
C	-0.32707500	-1.06488600	-0.62943000
C	-0.68380400	-2.20689800	1.29506000
C	-1.01183600	-2.13378400	-0.08876900
H	-1.04988000	-2.92893200	2.01280700
H	-1.69521000	-2.77287800	-0.63233500
C	0.87221600	-0.79284800	2.84919800
H	0.54492600	-1.45544600	3.65512400
H	1.96556800	-0.86127100	2.78058200
H	0.63603100	0.23864000	3.13968700
H	2.33003000	-1.78732000	-0.11175200
H	4.37750000	-0.68197900	-2.10795000
H	-1.53845300	1.05087100	-3.10110700

S12

Rh	-0.03917800	-0.66268300	-0.01398900
N	-1.87724200	-0.74421000	0.93741300
C	-1.90962000	-1.61794000	1.93086100
S	-3.44006200	-1.68283400	2.76655700
C	-4.06598100	-0.44939600	1.67729300
C	-5.33498300	0.12792100	1.62803800
H	-6.10939000	-0.15343800	2.33385000

C	-5.58490700	1.07303000	0.64062700
H	-6.57269000	1.51897200	0.59956000
C	-4.61043700	1.46110500	-0.30975400
C	-3.34764400	0.87551000	-0.24422100
H	-2.56955600	1.11528700	-0.95513500
C	-3.07487300	-0.07099700	0.75051400
C	-0.72052100	-2.39984100	2.17749000
C	0.33721000	-2.12695000	1.27199300
C	1.53277000	-2.83021100	1.40858000
H	2.35807500	-2.64869500	0.72965200
C	1.67315300	-3.77713800	2.42974500
H	2.60910900	-4.32035400	2.52585100
C	0.62794800	-4.03850900	3.32293800
H	0.75250900	-4.77829100	4.10686700
C	-0.57352700	-3.34946900	3.19976500
H	-1.39225600	-3.54633400	3.88728200
C	-4.97519100	2.49431200	-1.38291800
N	1.77867900	-0.85693100	-1.02742000
C	1.71155600	-1.72235600	-2.02917700
S	3.19654800	-1.89188200	-2.93040000
C	3.95005400	-0.70046600	-1.87854200
C	5.24784600	-0.19949300	-1.90475700
H	5.96496000	-0.53735300	-2.64555900
C	5.60962600	0.75346500	-0.95390400
H	6.62032000	1.14039000	-0.98180800
C	4.70922700	1.21409000	0.02742700
C	3.41308000	0.69127700	0.04113400
H	2.69669600	1.00169000	0.78660400
C	3.02647200	-0.25611700	-0.90717700
C	0.46540300	-2.41068800	-2.26507600
C	-0.56319600	-2.05493200	-1.35713500
C	-1.80890500	-2.66476500	-1.50419500
H	-2.62239300	-2.42590800	-0.82816900
C	-2.02450500	-3.59209100	-2.53013100
H	-3.00101100	-4.05775400	-2.63123000

C	-1.00225300	-3.93247600	-3.42250400
H	-1.18349500	-4.65539600	-4.21117200
C	0.24894800	-3.34047900	-3.29302500
H	1.04952200	-3.59599500	-3.98270200
C	5.09026000	2.28462600	1.06049800
C	4.79490800	1.76478700	2.48427500
H	5.07564200	2.52028900	3.22657700
H	5.36437500	0.85318000	2.69365600
H	3.73283000	1.53930600	2.62641400
C	6.57734400	2.66803800	0.98610300
H	6.83912200	3.10437700	0.01625200
H	7.22704700	1.80426600	1.16356300
H	6.80259600	3.41574300	1.75331700
C	4.24573400	3.55038000	0.79089900
H	4.48972300	4.33416500	1.51718800
H	3.17438800	3.33733500	0.87148600
H	4.43902300	3.94208500	-0.21374400
C	-3.78516000	2.81934400	-2.30099900
H	-3.42827500	1.93306000	-2.83736500
H	-2.94669300	3.24420000	-1.73751900
H	-4.09119500	3.55845900	-3.04841000
C	-6.12348000	1.93634300	-2.25235700
H	-6.40188100	2.66351800	-3.02322600
H	-7.01667800	1.72304600	-1.65703100
H	-5.82081300	1.00858100	-2.74983000
C	-5.42983100	3.80340500	-0.70097500
H	-6.30891900	3.65171400	-0.06741400
H	-5.69174100	4.55125300	-1.45761600
H	-4.63120100	4.21597500	-0.07394500
C	-0.18935200	3.94114600	-2.41034800
H	-0.54816500	3.11863100	-3.02099100
H	-0.11802300	4.92140300	-2.86959000
C	0.15509800	3.74735300	-1.13050200
H	0.52257100	4.56307700	-0.51919600
C	0.06065100	2.40433100	-0.54612900

O	-0.24389800	1.39899100	-1.18365100
N	0.34582000	2.28049800	0.83126700
C	0.52832000	3.23163600	1.81538700
C	0.64796700	1.17875600	2.65503300
C	0.72260200	2.55837200	2.99266400
H	0.48773900	4.28764500	1.60407100
H	0.75430700	0.30620500	3.28460400
H	0.89451000	2.98860900	3.96771400
N	0.42400500	1.02233400	1.36341200

S13

Rh	0.75240300	-1.22386700	0.32378200
N	2.79026900	-1.26507700	-0.08118000
C	3.32834500	-2.45423300	0.13814100
S	5.04887600	-2.51941500	-0.13885600
C	5.02592600	-0.81091500	-0.55612400
C	6.08649500	0.02317300	-0.90812300
H	7.10120400	-0.35474500	-0.97596500
C	5.80890000	1.36052000	-1.16009300
H	6.63494400	2.01202200	-1.42251800
C	4.50267300	1.89799100	-1.07683400
C	3.45364100	1.04929000	-0.72385600
H	2.43825700	1.40686200	-0.62525100
C	3.71434800	-0.30264900	-0.46593100
C	2.46835000	-3.51247300	0.61368300
C	1.12993200	-3.10366900	0.84258000
C	0.21585800	-4.04457800	1.31452700
H	-0.81463200	-3.76872400	1.50354600
C	0.62572300	-5.36316900	1.54441100
H	-0.09626800	-6.08657700	1.91282900
C	1.94688500	-5.76128400	1.31308500
H	2.24893700	-6.78660900	1.49961300
C	2.87327800	-4.83548700	0.84622900
H	3.90271600	-5.13347100	0.66400200

C	4.29040300	3.39487800	-1.33730900
N	-1.21263600	-1.31263200	1.03690400
C	-1.29511100	-1.05178700	2.33458700
S	-2.87935500	-1.29319400	3.01703400
C	-3.46047900	-1.81013600	1.44341600
C	-4.73346300	-2.22966600	1.07162300
H	-5.53560800	-2.29963100	1.79858800
C	-4.96463100	-2.53498000	-0.26891100
H	-5.96046700	-2.84847400	-0.55526700
C	-3.95577600	-2.42437400	-1.24734500
C	-2.67550800	-2.03553700	-0.84475000
H	-1.87068800	-1.95648600	-1.55908700
C	-2.42140500	-1.72918900	0.49097700
C	-0.09129400	-0.68829600	3.04312000
C	1.07529100	-0.72159700	2.23998900
C	2.29529700	-0.41914800	2.84607700
H	3.21506100	-0.43505700	2.27237400
C	2.34751300	-0.07720500	4.20206600
H	3.30564900	0.16152100	4.65533200
C	1.18627200	-0.03759600	4.98150900
H	1.24092200	0.22948100	6.03176400
C	-0.04031700	-0.34547700	4.40253700
H	-0.94891200	-0.32098900	4.99877400
C	-4.21482600	-2.67832200	-2.73852600
C	-3.30446900	-3.82337400	-3.23232900
H	-3.47255200	-4.00775600	-4.29953200
H	-3.51389000	-4.75112900	-2.68915900
H	-2.24437600	-3.58477700	-3.09689600
C	-5.67650500	-3.06041600	-3.02162100
H	-6.36913500	-2.26780300	-2.71870800
H	-5.96173500	-3.98496800	-2.50813400
H	-5.81167900	-3.22503800	-4.09540800
C	-3.89002100	-1.38479300	-3.52081200
H	-4.10010000	-1.52355600	-4.58740600
H	-2.83441300	-1.10909900	-3.42041800

H	-4.49018000	-0.54466100	-3.15550100
C	2.80073000	3.76487200	-1.38037400
H	2.29445100	3.55819300	-0.43305900
H	2.27827400	3.22176300	-2.17537600
H	2.68909200	4.83538100	-1.58034300
C	4.96382800	4.19337600	-0.19910700
H	4.83667000	5.26946000	-0.36326600
H	6.03733600	3.98654200	-0.14224400
H	4.52010200	3.93877500	0.76977300
C	4.91811400	3.79066900	-2.69141100
H	5.99899400	3.62413200	-2.71308400
H	4.74685800	4.85521400	-2.88507000
H	4.47221400	3.21827300	-3.51252900
C	-0.44581900	2.98707800	-2.56060800
H	-0.13660400	3.25048400	-1.55675500
H	-0.76579200	3.79376300	-3.20949700
C	-0.43552300	1.71570200	-2.98764700
H	-0.75102700	1.46011800	-3.99214800
C	-0.01025100	0.64373600	-2.08816700
O	0.20575300	0.78844700	-0.88454900
N	0.15198100	-0.64072600	-2.65729000
C	0.11672000	-1.06788000	-3.96943000
C	0.59709500	-2.74574000	-2.59592700
C	0.39708900	-2.40918000	-3.96269400
H	-0.08628300	-0.39290500	-4.78444500
H	0.83092900	-3.70030800	-2.14550900
H	0.45307300	-3.06673000	-4.81699500
N	0.44695100	-1.68445300	-1.82479300
C	-0.27461000	5.95716400	-0.35598200
C	-0.05785900	4.93879500	0.57512900
C	-1.00362400	3.93188100	0.81713300
C	-2.19512500	3.98553200	0.06377000
C	-2.41584800	5.00167900	-0.87248900
C	-1.46238800	5.99592500	-1.08300600
H	0.48404700	6.72029100	-0.50282000

H	0.87071600	4.93806500	1.13239300
H	-3.34367000	4.99118700	-1.43555100
H	-1.64545400	6.78412900	-1.80697500
C	-0.76428300	2.84142900	1.85630600
C	-1.72250500	2.99517900	3.05370500
H	-1.53457500	2.19859400	3.78202200
H	-1.56201800	3.95660500	3.55521000
H	-2.76873300	2.93333300	2.74618000
C	0.68464400	2.78377400	2.35036500
H	1.39096000	2.61470900	1.53007800
H	0.97220300	3.70484600	2.87045800
H	0.79894500	1.96385900	3.05930300
N	-3.22086300	3.00981600	0.22477500
C	-4.50662100	3.27192000	0.68913400
C	-3.14507100	1.69208800	-0.19866100
C	-5.22944700	2.10405600	0.56061500
C	-4.37668100	1.11207400	-0.00710900
H	-2.22249100	1.30201700	-0.59398500
H	-6.26352400	1.97964400	0.85293600
H	-4.64204700	0.09650300	-0.25245000
C	-4.90017400	4.60171800	1.24667400
H	-5.89938000	4.52839600	1.68399000
H	-4.21126700	4.93570500	2.03206300
H	-4.92694900	5.39205900	0.48673700
H	-0.98681900	1.87917600	1.38253700

S14

Rh	-1.54519100	0.47541100	0.37664700
N	-2.83532000	-1.02137700	-0.17898200
C	-4.09990100	-0.78569400	0.12511400
S	-5.17135300	-2.11247200	-0.26163500
C	-3.79054100	-3.03269500	-0.85948400
C	-3.73310200	-4.32635400	-1.38015800
H	-4.62649000	-4.93447700	-1.47640400

C	-2.49303700	-4.82392600	-1.77018100
H	-2.45194600	-5.83378000	-2.16410800
C	-1.30227200	-4.06554400	-1.67170900
C	-1.37747200	-2.77820000	-1.14737900
H	-0.51281600	-2.13822700	-1.04572500
C	-2.61394400	-2.26937200	-0.73824900
C	-4.40534000	0.47167700	0.77011900
C	-3.25344400	1.25786400	1.04198800
C	-3.42617000	2.48871200	1.67309600
H	-2.56434000	3.10768900	1.90228400
C	-4.70934600	2.93073100	2.01463400
H	-4.83025200	3.89241600	2.50608900
C	-5.83880500	2.15100500	1.73801200
H	-6.82710500	2.50677100	2.01082200
C	-5.69011100	0.91667200	1.11452000
H	-6.56109200	0.30385000	0.89541900
C	0.04201200	-4.67150100	-2.09899200
N	-0.22235100	1.88879500	1.19218400
C	0.18893100	1.54744400	2.40304500
S	1.47037400	2.56186000	3.03716200
C	1.44225400	3.49214000	1.54665600
C	2.24193300	4.56907100	1.16316900
H	2.98748200	4.98219600	1.83508400
C	2.06474000	5.09780000	-0.11081900
H	2.69360000	5.92776800	-0.41475800
C	1.09716800	4.59348200	-1.01269300
C	0.28812400	3.53571600	-0.59959600
H	-0.47524500	3.11938500	-1.23814100
C	0.46560400	2.97569500	0.67039500
C	-0.40649700	0.40200500	3.04541700
C	-1.33203700	-0.29014100	2.22290900
C	-1.99453300	-1.38633800	2.78490600
H	-2.71709400	-1.94631600	2.20167100
C	-1.73191500	-1.78495400	4.09798200
H	-2.26080300	-2.63872600	4.51303200

C	-0.79456100	-1.10469300	4.88741200
H	-0.59926300	-1.42654500	5.90527800
C	-0.12789900	-0.00506200	4.36100200
H	0.58778800	0.54404100	4.96863200
C	0.98580600	5.19546200	-2.41871900
C	-0.14155300	4.54508400	-3.23614200
H	-0.19217100	5.00998900	-4.22598000
H	-1.11930200	4.67235500	-2.75922700
H	0.02885900	3.47392100	-3.38171500
C	0.70797500	6.71039600	-2.31717000
H	1.50504600	7.24042200	-1.78636300
H	-0.23242900	6.89922700	-1.78840500
H	0.62972700	7.14648700	-3.31909200
C	2.31914000	4.95757300	-3.16273600
H	2.27105500	5.37829700	-4.17321200
H	2.52547000	3.88389200	-3.25089400
H	3.16404900	5.42498800	-2.64648900
C	1.16354400	-3.61635500	-2.13741100
H	1.35994400	-3.18858700	-1.15052200
H	0.92266500	-2.79532600	-2.82220400
H	2.09332100	-4.08376900	-2.47929100
C	0.43348100	-5.76467800	-1.08003700
H	1.39275800	-6.21837000	-1.35632700
H	-0.31760200	-6.56034400	-1.03691000
H	0.53174200	-5.33870700	-0.07534300
C	-0.07573400	-5.29302900	-3.50643100
H	-0.80712600	-6.10595700	-3.54100500
H	0.89028000	-5.70801200	-3.81525300
H	-0.37447800	-4.53895800	-4.24286900
C	2.87115100	-0.16169800	-2.06764700
H	2.83780700	-1.25648700	-2.01583300
H	3.73820900	0.11443500	-2.67498500
C	1.60590900	0.40615200	-2.62463900
H	1.63335700	0.96274700	-3.55226300
C	0.44617500	0.27739600	-1.92529200

O	0.30657400	-0.25590600	-0.75277500
N	-0.76743000	0.80082900	-2.51149100
C	-1.13014700	0.96814800	-3.81112300
C	-2.74861200	1.64735400	-2.45540900
C	-2.40018700	1.51846900	-3.81461100
H	-0.47556900	0.66699200	-4.61381800
H	-3.65757200	2.01378300	-1.99999900
H	-2.99661700	1.77533800	-4.67695000
N	-1.75402200	1.21452100	-1.68063700
C	4.53924200	-4.57466600	-0.08326300
C	3.64281300	-4.01300200	0.82204600
C	3.47232500	-2.62460300	0.93573200
C	4.26551200	-1.82869400	0.09703100
C	5.17532000	-2.37554700	-0.81439000
C	5.31316900	-3.75604300	-0.90856000
H	4.63608300	-5.65431400	-0.14317000
H	3.05789500	-4.66749500	1.45876800
H	5.75435100	-1.71257600	-1.44986800
H	6.01366600	-4.18596500	-1.61689700
C	2.51865400	-2.04192100	1.96563000
C	3.14230300	-2.11573300	3.37206700
H	2.46702100	-1.65852900	4.10272300
H	3.31382800	-3.15583200	3.67099800
H	4.10443400	-1.59226400	3.41601900
C	1.14429400	-2.72222000	1.93611700
H	0.64441800	-2.56530900	0.97752300
H	1.21892800	-3.79975200	2.11725200
H	0.51015300	-2.30360900	2.71660300
N	4.14403300	-0.39469200	0.11651600
C	4.99159900	0.45790700	0.67407600
C	3.08235800	0.32354000	-0.59843800
C	4.57703800	1.80135300	0.37136100
C	3.46037600	1.73289200	-0.40040200
H	2.10691500	0.10750700	-0.11584200
H	5.09281500	2.68792100	0.71443200

H	2.89289700	2.55524600	-0.81387100
C	6.17778100	0.04087300	1.46944900
H	6.27395800	0.67396700	2.35658400
H	6.11359900	-1.00628300	1.76996400
H	7.09069000	0.17303100	0.87529900
H	2.35936500	-0.98282600	1.73756900

MeCN

C	0.00000000	0.00000000	0.28024600
N	0.00000000	0.00000000	1.44034800
C	0.00000000	0.00000000	-1.18065400
H	-1.02654700	0.00000000	-1.55999600
H	0.51327300	0.88901600	-1.55999600
H	0.51327300	-0.88901600	-1.55999600

Δ-RhS

Rh	0.00546400	-0.39092800	0.00167400
N	-1.82369200	-0.54877600	0.99881600
C	-1.79373100	-1.43644100	1.98118800
S	-3.30759200	-1.60770600	2.83336300
C	-4.01889200	-0.39593300	1.77597400
C	-5.31971100	0.10784600	1.76016500
H	-6.06366400	-0.23516700	2.47151000
C	-5.64133800	1.06128500	0.80208800
H	-6.65332500	1.45136400	0.78771500
C	-4.70326400	1.52975700	-0.14744900
C	-3.40799100	1.01848000	-0.11177400
H	-2.65126100	1.33591000	-0.81141300
C	-3.06280200	0.05959000	0.84662600
C	-0.56437200	-2.15123100	2.22645200
C	0.48353700	-1.80358900	1.33919600
C	1.71731400	-2.43283100	1.49549300
H	2.54543000	-2.19378200	0.83800700
C	1.90074500	-3.38077800	2.50877000

H	2.86783200	-3.86414400	2.61690800
C	0.85887400	-3.71883300	3.37913400
H	1.01507600	-4.45892800	4.15719900
C	-0.37943700	-3.10222800	3.24128000
H	-1.19583100	-3.35623200	3.91276800
C	-5.13531200	2.57542800	-1.18279400
N	1.83508900	-0.58295600	-0.98802000
C	1.81389500	-1.51292300	-1.92969400
S	3.33227600	-1.71364100	-2.77049200
C	4.03534900	-0.45125600	-1.76499500
C	5.32966700	0.06066600	-1.76318800
H	6.08055300	-0.30803000	-2.45413900
C	5.64536500	1.06110100	-0.84405300
H	6.65508000	1.45218800	-0.84601100
C	4.70078100	1.56273300	0.07394700
C	3.40628700	1.03882800	0.05121100
H	2.64787800	1.38725700	0.73534500
C	3.06990700	0.03948900	-0.85951500
C	0.58881100	-2.24412800	-2.14472500
C	-0.46238300	-1.86187800	-1.27508700
C	-1.69255400	-2.50421400	-1.40487700
H	-2.52281400	-2.24030600	-0.75971400
C	-1.86925500	-3.49765300	-2.37480200
H	-2.83364400	-3.99048600	-2.46244000
C	-0.82428200	-3.86932800	-3.22773500
H	-0.97556900	-4.64455000	-3.97180900
C	0.41057900	-3.24086800	-3.11599900
H	1.22955300	-3.52068400	-3.77393900
C	5.03618500	2.64941300	1.10560100
C	4.82936600	2.07392200	2.52454600
H	5.04742600	2.83747000	3.28029300
H	5.49239100	1.22072300	2.70221200
H	3.80028800	1.72792700	2.67452000
C	6.48723900	3.14292800	0.98846300
H	6.68873300	3.58083600	0.00483400

H	7.20619200	2.33503200	1.16137600
H	6.67493100	3.91697700	1.73958000
C	4.09372900	3.85605300	0.90050300
H	4.31445600	4.63884300	1.63523000
H	3.04065100	3.57361200	1.01240300
H	4.21805700	4.28481800	-0.09967700
C	-3.98910800	2.94550800	-2.14037900
H	-3.64061300	2.07133000	-2.70298200
H	-3.13781600	3.37797100	-1.60106500
H	-4.34146500	3.68983400	-2.86205500
C	-6.30219200	2.01178900	-2.02301900
H	-6.62175100	2.74809000	-2.76894200
H	-7.17027900	1.77006200	-1.40242700
H	-6.00040500	1.09925500	-2.54872600
C	-5.59253200	3.85767300	-0.45376900
H	-6.43688200	3.66450900	0.21493100
H	-5.90948200	4.61450900	-1.18001300
H	-4.77788900	4.27804000	0.14644200
C	0.58814000	1.89487800	2.42682100
N	0.46473800	1.20670700	1.50602300
C	0.77431000	2.76233900	3.58274600
H	0.68085500	2.17877800	4.50372200
H	1.77025600	3.21488800	3.54793900
H	0.02023100	3.55506400	3.58433700
C	-0.48790700	1.78988700	-2.52574700
N	-0.44146000	1.14441800	-1.56769500
C	-0.55867500	2.60155300	-3.73362800
H	-1.43677100	3.25279200	-3.69232400
H	0.34151500	3.21677400	-3.82514500
H	-0.63750700	1.95282200	-4.61143500

TS-1a

Rh	-1.65488700	-0.25765700	0.32334300
N	-2.20133600	-2.13289900	-0.30219100

C	-3.45954000	-2.44353600	-0.04163900
S	-3.87531200	-4.08803400	-0.46229700
C	-2.21592700	-4.35395200	-0.99860400
C	-1.60902100	-5.50741400	-1.49775400
H	-2.16904000	-6.42779400	-1.62587000
C	-0.25612600	-5.45176100	-1.82006400
H	0.21386900	-6.35417800	-2.19561000
C	0.51593500	-4.27475500	-1.67252200
C	-0.10640600	-3.13417500	-1.17297800
H	0.42406000	-2.20497100	-1.03190500
C	-1.46254100	-3.17586700	-0.83630200
C	-4.26865900	-1.43532400	0.60627900
C	-3.54309800	-0.26069500	0.94133500
C	-4.21260200	0.77691000	1.58628800
H	-3.68013400	1.68138100	1.86149900
C	-5.57548300	0.65364900	1.88123000
H	-6.08820900	1.46898800	2.38421300
C	-6.28559100	-0.50449000	1.54394500
H	-7.34119600	-0.58522500	1.78215200
C	-5.63357500	-1.55438900	0.90544200
H	-6.17654200	-2.45822700	0.64056800
C	2.01233200	-4.27621200	-2.01481100
N	-1.05832000	1.55013800	1.23003600
C	-0.59735300	1.35469700	2.45695300
S	0.12138600	2.76746400	3.19354600
C	-0.23368600	3.68131900	1.73705500
C	0.03743200	5.01619800	1.45565600
H	0.52763800	5.65431000	2.18352200
C	-0.33671100	5.51946500	0.21088400
H	-0.12537400	6.55972600	-0.00137000
C	-0.98481500	4.72400900	-0.75623800
C	-1.24946200	3.38398200	-0.45171900
H	-1.76035800	2.74342900	-1.15496700
C	-0.87361600	2.85601600	0.78513800
C	-0.68424100	0.03844400	3.03900200

C	-1.17837400	-0.94875300	2.15046300
C	-1.27904700	-2.25691300	2.63118600
H	-1.64875500	-3.05086500	1.99221200
C	-0.89682000	-2.56751000	3.93960100
H	-0.98340600	-3.59186300	4.29171600
C	-0.40665800	-1.58052100	4.80380700
H	-0.11754200	-1.83507400	5.81832800
C	-0.29750800	-0.27040900	4.35305300
H	0.07900800	0.50632300	5.01429300
C	-1.38842800	5.27663200	-2.13141300
C	-2.87691200	4.96858400	-2.40424800
H	-3.17182700	5.37940100	-3.37615000
H	-3.51637300	5.41713200	-1.63663800
H	-3.07691700	3.89267600	-2.42726900
C	-1.18424500	6.79736700	-2.23328100
H	-0.13070700	7.07799000	-2.12684100
H	-1.76418300	7.33573400	-1.47586200
H	-1.51674500	7.14592000	-3.21620900
C	-0.52047300	4.59312800	-3.21050300
H	-0.77477400	4.97669100	-4.20514700
H	-0.68183400	3.51151000	-3.21474000
H	0.54488100	4.78006900	-3.03318700
C	2.59802600	-2.85189600	-2.03908600
H	2.55237300	-2.37227900	-1.05707500
H	2.07335300	-2.21499100	-2.76066500
H	3.65305100	-2.89303200	-2.32738200
C	2.75975100	-5.09468900	-0.93870200
H	3.83477300	-5.11373400	-1.15275400
H	2.40234900	-6.12911900	-0.90189000
H	2.61584800	-4.65178100	0.05315200
C	2.24438600	-4.91041400	-3.40223200
H	1.92308700	-5.95550400	-3.44042600
H	3.31142400	-4.88738400	-3.64975400
H	1.70190500	-4.36155900	-4.17988000
C	2.64011300	1.00591900	-2.12747100

H	2.92267100	0.02714300	-1.75468100
H	3.44539500	1.56457500	-2.59504500
C	1.33505700	1.18906200	-2.59747400
H	1.11518400	1.97132800	-3.31346600
C	0.27668000	0.54707400	-1.93907000
O	0.36964100	-0.08027600	-0.84983300
N	-1.03278600	0.67482300	-2.50383700
C	-1.42995000	0.98207800	-3.77577700
C	-3.18465700	0.64400100	-2.45869300
C	-2.80848100	0.97424000	-3.78240200
H	-0.70942200	1.14978900	-4.56059200
H	-4.16708800	0.52842900	-2.02322200
H	-3.45711200	1.16477500	-4.62396500
N	-2.10888000	0.46721500	-1.70146600
C	5.92385300	-2.40715900	-0.17740700
C	5.00476500	-2.17359300	0.84041800
C	4.39027100	-0.92376100	1.01956500
C	4.74213300	0.09343600	0.11993300
C	5.65946000	-0.13529200	-0.91547600
C	6.25621300	-1.38159100	-1.06534600
H	6.37922000	-3.38750900	-0.27934000
H	4.75462800	-2.98082800	1.52133400
H	5.88859000	0.67103400	-1.60480800
H	6.96714300	-1.55127300	-1.86761800
C	3.39002800	-0.73411400	2.14583900
C	4.04046800	-0.96254600	3.52110800
H	3.30909400	-0.77461400	4.31460500
H	4.39397700	-1.99354300	3.63185600
H	4.89540900	-0.29542400	3.67429800
C	2.17678000	-1.65682900	1.94591900
H	1.63132500	-1.40653900	1.03178800
H	2.48450300	-2.70645900	1.87957500
H	1.48973600	-1.56644500	2.78751200
N	4.15404300	1.40151700	0.19986000
C	4.85120800	2.56699500	0.33676400

C	2.81630500	1.68965100	-0.15361600
C	3.96059800	3.63417900	0.13251700
C	2.70565300	3.09973900	-0.15106500
H	2.03613300	0.98879100	0.12063700
H	4.23217300	4.67949800	0.18691700
H	1.79299600	3.64104800	-0.35736800
C	6.31082600	2.61046900	0.64293000
H	6.56023300	3.56604300	1.11119200
H	6.59868500	1.79959700	1.31892900
H	6.92490400	2.51377200	-0.26058300
H	3.03487900	0.30132900	2.12494600

TS-1b

Rh	1.89972800	-0.75535000	0.24384300
N	3.65179200	0.23970600	-0.19618800
C	4.69374800	-0.56232400	-0.33945100
S	6.17254900	0.27068700	-0.75234600
C	5.29798500	1.79961900	-0.72681900
C	5.76036800	3.09350300	-0.96949400
H	6.79940900	3.28265800	-1.21808600
C	4.84946800	4.14048000	-0.88532100
H	5.20900100	5.14604800	-1.07629000
C	3.48648600	3.94131200	-0.56026600
C	3.04388900	2.64455600	-0.31206400
H	2.01951500	2.42459800	-0.04878300
C	3.94535000	1.57855500	-0.40299400
C	4.48345700	-1.97792800	-0.14302400
C	3.14583000	-2.30776100	0.19951400
C	2.82710600	-3.64940300	0.40628200
H	1.81564200	-3.93628600	0.67312100
C	3.81104500	-4.63502100	0.26958600
H	3.54875900	-5.67657600	0.43442800
C	5.12676400	-4.30041500	-0.07094200
H	5.87895200	-5.07625200	-0.17046300

C	5.46723400	-2.96847300	-0.27968000
H	6.48599700	-2.69756400	-0.54564800
C	2.53866700	5.14490900	-0.48740200
N	0.24404500	-1.86067700	0.91061700
C	0.20653700	-1.96443700	2.23223800
S	-1.22145200	-2.76505500	2.84793900
C	-1.81488100	-2.93238400	1.20177700
C	-3.01371500	-3.47540500	0.75017100
H	-3.73390200	-3.89906300	1.44255500
C	-3.27088200	-3.46498800	-0.62014200
H	-4.20284400	-3.89310700	-0.96582400
C	-2.35928300	-2.92891900	-1.55196600
C	-1.15589900	-2.39723700	-1.07815100
H	-0.41894100	-1.99048100	-1.75444700
C	-0.88112600	-2.39177100	0.28995500
C	1.26283400	-1.36272300	3.00671700
C	2.22480500	-0.67793500	2.22050300
C	3.26282200	-0.02475500	2.88813800
H	4.02062700	0.51266800	2.32831100
C	3.33770700	-0.05312000	4.28426400
H	4.15407500	0.46022800	4.78526500
C	2.38254000	-0.73673400	5.04698700
H	2.45797200	-0.75441400	6.12945000
C	1.33766200	-1.39264200	4.40860700
H	0.58775800	-1.92416500	4.98977000
C	-2.64875000	-2.89162300	-3.05967200
C	-1.48828500	-3.56072200	-3.82844100
H	-1.69692900	-3.55325700	-4.90406600
H	-1.35960500	-4.60151200	-3.51310300
H	-0.53888800	-3.03872600	-3.67208400
C	-3.95420300	-3.61769700	-3.42375600
H	-4.82755100	-3.15035500	-2.95591200
H	-3.92674400	-4.67228600	-3.12896300
H	-4.10398600	-3.58046700	-4.50750600
C	-2.76765900	-1.41721700	-3.50469100

H	-2.96996900	-1.35814400	-4.58016700
H	-1.84416400	-0.86729900	-3.30333500
H	-3.58575600	-0.91227300	-2.97761000
C	1.09469600	4.72499000	-0.16004800
H	1.02744600	4.22024900	0.81006600
H	0.68154800	4.05583600	-0.92323700
H	0.45843700	5.61546700	-0.11771500
C	3.02839700	6.11063700	0.61362000
H	2.36668100	6.98207300	0.67585900
H	4.04107400	6.47458000	0.41336900
H	3.03616500	5.61544900	1.59079200
C	2.53194500	5.87285100	-1.84948200
H	3.52591500	6.24107900	-2.12159100
H	1.85648100	6.73510000	-1.81392300
H	2.19133100	5.20364800	-2.64756500
C	-2.07709100	2.28470400	-0.61014000
H	-1.57854200	2.73066000	0.24438000
H	-2.95585000	2.82415600	-0.95260900
C	-1.29115600	1.65736000	-1.59731200
H	-1.70839700	1.48816000	-2.58239000
C	-0.09633000	1.02377900	-1.24348500
O	0.38885800	0.95728300	-0.08327100
N	0.62138300	0.33268800	-2.28338200
C	0.58040100	0.46170500	-3.64282700
C	2.00058700	-1.13557400	-3.04615900
C	1.45491900	-0.46763700	-4.16670500
H	-0.03570000	1.20957100	-4.11629200
H	2.72683100	-1.93462400	-2.99788100
H	1.68100600	-0.63444500	-5.20899700
N	1.49053600	-0.64728100	-1.92134500
C	-7.59739900	2.93805200	-0.88956700
C	-7.45833700	1.58726800	-0.57866300
C	-6.33041400	1.09735700	0.09516300
C	-5.33685800	2.02945600	0.43389700
C	-5.46674200	3.38926500	0.13256900

C	-6.60147300	3.84827100	-0.52948400
H	-8.48641700	3.28218600	-1.40904400
H	-8.24821200	0.89572900	-0.85573400
H	-4.67465500	4.07409200	0.41993900
H	-6.70593100	4.90268100	-0.76392000
C	-6.22509700	-0.37555300	0.45893000
C	-7.35667600	-0.78863500	1.41641300
H	-7.23895800	-1.83615400	1.71435400
H	-8.33943600	-0.68364700	0.94420300
H	-7.35275500	-0.17296700	2.32217400
C	-6.20774600	-1.26217300	-0.79729900
H	-5.37414600	-1.00098300	-1.45811400
H	-7.13512700	-1.16047200	-1.37137200
H	-6.09900400	-2.31459500	-0.51668000
N	-4.14740800	1.58970100	1.10042100
C	-3.82314400	1.81390300	2.39969300
C	-3.09869300	0.89268400	0.45625000
C	-2.59231200	1.17824900	2.65916300
C	-2.17194400	0.56028300	1.49024100
H	-3.36620000	0.25823700	-0.37725500
H	-2.08167100	1.18819200	3.61206900
H	-1.25312600	0.02410700	1.33144000
H	-5.27947000	-0.53279500	0.98745900
C	-4.68813200	2.60910100	3.31671900
H	-4.39585000	2.43067300	4.35386300
H	-4.60124300	3.68471300	3.12112100
H	-5.74339400	2.34364700	3.19380600

12. Single-Crystal X-Ray Diffraction Analysis of 4j

A suitable crystal of $C_{21}H_{18}BrF_3N_2O$ (**4j**) was selected under inert oil and mounted using a MiTeGen loop. Intensity data of the crystal were recorded with a STADIVARI diffractometer (Stoe & Cie). The diffractometer was operated with Cu-K_α radiation (1.54186 Å, microfocus source) and equipped with a Dectris PILATUS 300K detector. Evaluation, integration and reduction of the diffraction data was carried out using the X-Area software suite.[16] Multi-scan and numerical absorption corrections were applied with the X-Red32 and LANA modules of the X-Area software suite.[17,18] The structure was solved using dual-space methods (SHELXT-2014/5) and refined against F^2 (SHELXL-2018/3 using ShelXle interface).[19-21] All non-hydrogen atoms were refined with anisotropic displacement parameters. The hydrogen atoms bonded to carbon atoms were refined using the "riding model" approach with isotropic displacement parameters 1.2 times (for CH_3 groups 1.5 times) of that of the preceding carbon atom. The hydrogen atoms bonded to nitrogen atoms were refined freely. CCDC 1991853 contains the supplementary crystallographic data for this paper. These data can be obtained free of charge from The Cambridge Crystallographic Data Centre via www.ccdc.cam.ac.uk/structures.

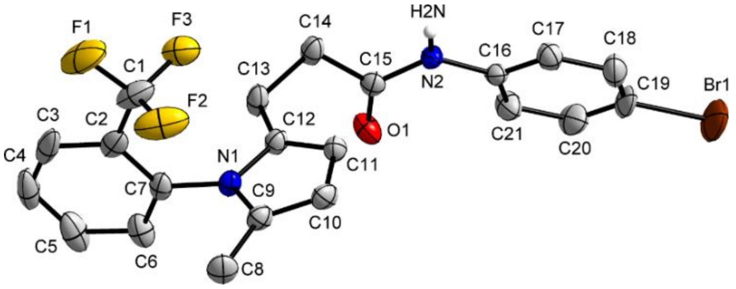

Figure S15. First molecule in the asymmetric unit of **4j**.

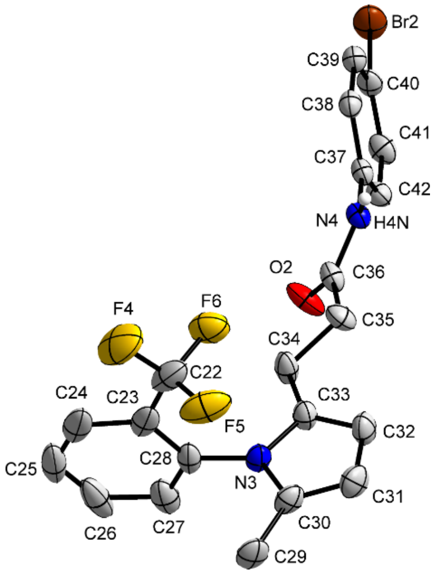

Figure S16. Second molecule in the asymmetric unit of **4j** that has a different conformation than the first one.

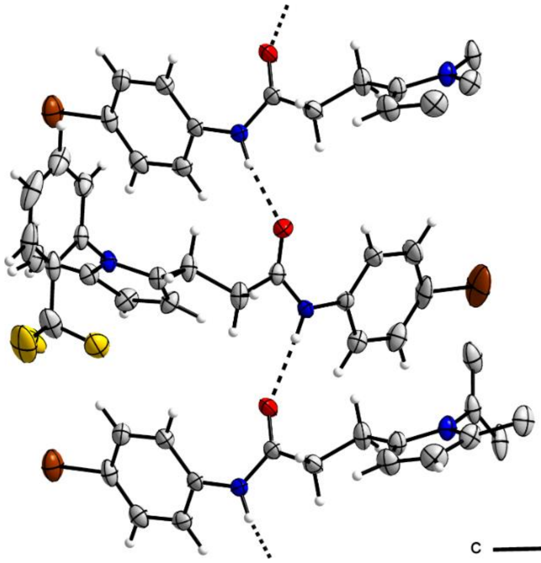

Figure S17. Hydrogen bonding in the structure of **4j**. The molecules are stacked with the help of H-bonding along the *a* axis.

Table S4. Crystal data and structure refinement for **4j**.

Identification code	CXD57
Empirical formula	$C_{21}H_{18}BrF_3N_2O$
Molar mass / g·mol^{-1}	451.28
Space group (No.)	$P2_1$ (4)
a / Å	9.3216(2)
b / Å	14.1189(2)
c / Å	15.3729(2)
β / °	91.2840(10)
V / Å^3	2022.73(6)
Z	4
$\rho_{calc.}$ / g·cm^{-3}	1.482
μ / mm^{-1}	3.133
Color	colorless
Crystal habitus	needle
Crystal size / mm^3	0.242 × 0.045 × 0.019
T / K	100
λ / Å	1.54186 (Cu-K$_\alpha$)
θ range / °	2.875 to 75.637
Range of Miller indices	$-11 \leq h \leq 5$
	$-17 \leq k \leq 17$

	$-19 \leq l \leq 19$
Absorption correction	multi-scan and numerical
T_{min}, T_{max}	0.4045, 0.8038
R_{int}, R_{σ}	0.0234, 0.0248
Completeness of the data set	0.993
No. of measured reflections	46527
No. of independent reflections	8244
No. of parameters	513
No. of restrains	1
S (all data)	1.041
$R(F)$ ($I \geq 2\sigma(I)$, all data)	0.0316, 0.0371
$wR(F^2)$ ($I \geq 2\sigma(I)$, all data)	0.0716, 0.0734
Extinction coefficient	not refined
Flack parameter x	–0.030(4)
$\Delta\rho_{max}$, $\Delta\rho_{min}$ / e·Å^{-3}	0.930, –0.790

Table S5. Atomic coordinates (×10^4) and equivalent isotropic displacement parameters (Å^2×10^3) for **4j**. U_{eq} is defined as 1/3 of the trace of the orthogonalized U_{IJ} tensor.

Atom	x	y	z	U(eq)
C(1)	-762(6)	3690(3)	11334(3)	48(1)
C(2)	850(5)	3604(3)	11293(2)	35(1)
C(3)	1554(7)	2863(3)	11715(2)	51(1)
C(4)	3005(7)	2759(3)	11661(3)	58(2)
C(5)	3807(6)	3407(3)	11194(3)	47(1)
C(6)	3125(5)	4162(3)	10772(2)	36(1)
C(7)	1653(4)	4252(2)	10816(2)	27(1)
C(8)	1074(5)	6185(3)	11572(2)	37(1)
C(9)	599(4)	5890(2)	10680(2)	27(1)
C(10)	-143(4)	6357(2)	10037(2)	31(1)
C(11)	-247(4)	5763(2)	9298(2)	30(1)
C(12)	443(4)	4934(2)	9499(2)	26(1)
C(13)	723(5)	4058(2)	8974(2)	32(1)

C(14)	-103(5)	4062(2)	8104(2)	32(1)
C(15)	489(4)	4760(2)	7455(2)	26(1)
C(16)	-321(4)	5868(2)	6286(2)	24(1)
C(17)	-1472(4)	6466(3)	6084(2)	32(1)
C(18)	-1370(5)	7112(3)	5407(3)	42(1)
C(19)	-120(6)	7145(3)	4939(2)	45(1)
C(20)	1018(5)	6555(3)	5132(3)	44(1)
C(21)	926(4)	5908(3)	5807(2)	35(1)
C(22)	4009(7)	4698(3)	3665(3)	55(1)
C(23)	5592(6)	4838(3)	3638(2)	46(1)
C(24)	6144(8)	5591(3)	3174(3)	65(2)
C(25)	7589(9)	5774(3)	3185(3)	72(2)
C(26)	8519(7)	5212(4)	3653(3)	63(2)
C(27)	8008(6)	4424(3)	4106(3)	46(1)
C(28)	6552(5)	4240(2)	4101(2)	35(1)
C(29)	6184(5)	2351(3)	3237(2)	42(1)
C(30)	5775(4)	2551(3)	4151(2)	35(1)
C(31)	5207(5)	1985(3)	4768(3)	43(1)
C(32)	5108(4)	2516(3)	5553(2)	39(1)
C(33)	5630(4)	3397(3)	5403(2)	32(1)
C(34)	5867(5)	4227(3)	5997(2)	36(1)
C(35)	5097(4)	4105(3)	6845(2)	34(1)
C(36)	5519(4)	4809(3)	7540(2)	29(1)
C(37)	4681(4)	5578(2)	8861(2)	26(1)
C(38)	3585(4)	6203(3)	9083(2)	29(1)
C(39)	3732(4)	6760(3)	9825(2)	33(1)
C(40)	4975(5)	6691(3)	10331(2)	33(1)
C(41)	6054(4)	6069(3)	10128(2)	34(1)
C(42)	5904(4)	5502(3)	9385(2)	30(1)
N(1)	951(3)	5005(2)	10348(2)	24(1)

N(2)	-519(3)	5213(2)	6970(2)	24(1)
N(3)	6027(4)	3429(2)	4540(2)	30(1)
N(4)	4487(3)	5014(2)	8107(2)	26(1)
O(1)	1777(3)	4859(2)	7357(2)	35(1)
O(2)	6727(3)	5143(2)	7611(2)	45(1)
F(1)	-1326(4)	2979(3)	11802(2)	79(1)
F(2)	-1203(3)	4497(2)	11688(2)	66(1)
F(3)	-1410(3)	3638(2)	10549(2)	49(1)
F(4)	3278(5)	5339(3)	3194(2)	90(1)
F(5)	3571(4)	3843(2)	3373(2)	69(1)
F(6)	3500(3)	4764(2)	4468(2)	57(1)
Br(1)	26(1)	8019(1)	4008(1)	81(1)
Br(2)	5223(1)	7506(1)	11305(1)	50(1)

Table S6. Bond lengths [Å] and angles [°] for **4j**.

C(1)-F(2)	1.332(5)
C(1)-F(3)	1.340(5)
C(1)-F(1)	1.347(5)
C(1)-C(2)	1.510(7)
C(2)-C(3)	1.388(6)
C(2)-C(7)	1.400(5)
C(3)-C(4)	1.365(8)
C(3)-H(3)	0.9500
C(4)-C(5)	1.391(7)
C(4)-H(4)	0.9500
C(5)-C(6)	1.394(5)
C(5)-H(5)	0.9500
C(6)-C(7)	1.381(6)
C(6)-H(6)	0.9500
C(7)-N(1)	1.433(4)
C(8)-C(9)	1.491(5)
C(8)-H(8A)	0.9800
C(8)-H(8B)	0.9800

C(8)-H(8C)	0.9800
C(9)-C(10)	1.364(5)
C(9)-N(1)	1.391(4)
C(10)-C(11)	1.414(5)
C(10)-H(10)	0.9500
C(11)-C(12)	1.367(5)
C(11)-H(11)	0.9500
C(12)-N(1)	1.383(4)
C(12)-C(13)	1.503(5)
C(13)-C(14)	1.528(5)
C(13)-H(13A)	0.9900
C(13)-H(13B)	0.9900
C(14)-C(15)	1.515(5)
C(14)-H(14A)	0.9900
C(14)-H(14B)	0.9900
C(15)-O(1)	1.222(4)
C(15)-N(2)	1.348(5)
C(16)-C(21)	1.391(5)
C(16)-C(17)	1.395(5)
C(16)-N(2)	1.416(4)
C(17)-C(18)	1.388(5)
C(17)-H(17)	0.9500
C(18)-C(19)	1.384(7)
C(18)-H(18)	0.9500
C(19)-C(20)	1.376(7)
C(19)-Br(1)	1.896(3)
C(20)-C(21)	1.387(5)
C(20)-H(20)	0.9500
C(21)-H(21)	0.9500
C(22)-F(6)	1.336(5)
C(22)-F(4)	1.338(5)
C(22)-F(5)	1.348(6)
C(22)-C(23)	1.490(8)
C(23)-C(24)	1.386(7)
C(23)-C(28)	1.412(6)
C(24)-C(25)	1.371(9)
C(24)-H(24)	0.9500

C(25)-C(26)	1.368(9)
C(25)-H(25)	0.9500
C(26)-C(27)	1.402(6)
C(26)-H(26)	0.9500
C(27)-C(28)	1.381(6)
C(27)-H(27)	0.9500
C(28)-N(3)	1.421(4)
C(29)-C(30)	1.491(5)
C(29)-H(29A)	0.9800
C(29)-H(29B)	0.9800
C(29)-H(29C)	0.9800
C(30)-C(31)	1.358(6)
C(30)-N(3)	1.394(5)
C(31)-C(32)	1.425(6)
C(31)-H(31)	0.9500
C(32)-C(33)	1.358(5)
C(32)-H(32)	0.9500
C(33)-N(3)	1.386(4)
C(33)-C(34)	1.500(5)
C(34)-C(35)	1.513(5)
C(34)-H(34A)	0.9900
C(34)-H(34B)	0.9900
C(35)-C(36)	1.504(5)
C(35)-H(35A)	0.9900
C(35)-H(35B)	0.9900
C(36)-O(2)	1.224(4)
C(36)-N(4)	1.344(5)
C(37)-C(42)	1.386(5)
C(37)-C(38)	1.398(5)
C(37)-N(4)	1.413(4)
C(38)-C(39)	1.390(5)
C(38)-H(38)	0.9500
C(39)-C(40)	1.385(6)
C(39)-H(39)	0.9500
C(40)-C(41)	1.376(6)
C(40)-Br(2)	1.898(3)
C(41)-C(42)	1.400(5)

C(41)-H(41)	0.9500
C(42)-H(42)	0.9500
N(2)-H(2N)	0.81(4)
N(4)-H(4N)	0.85(4)
F(2)-C(1)-F(3)	106.1(4)
F(2)-C(1)-F(1)	107.0(3)
F(3)-C(1)-F(1)	105.5(3)
F(2)-C(1)-C(2)	113.7(3)
F(3)-C(1)-C(2)	112.8(3)
F(1)-C(1)-C(2)	111.3(4)
C(3)-C(2)-C(7)	119.0(4)
C(3)-C(2)-C(1)	120.1(4)
C(7)-C(2)-C(1)	120.9(4)
C(4)-C(3)-C(2)	120.7(4)
C(4)-C(3)-H(3)	119.6
C(2)-C(3)-H(3)	119.6
C(3)-C(4)-C(5)	120.4(4)
C(3)-C(4)-H(4)	119.8
C(5)-C(4)-H(4)	119.8
C(4)-C(5)-C(6)	119.9(5)
C(4)-C(5)-H(5)	120.1
C(6)-C(5)-H(5)	120.1
C(7)-C(6)-C(5)	119.4(4)
C(7)-C(6)-H(6)	120.3
C(5)-C(6)-H(6)	120.3
C(6)-C(7)-C(2)	120.6(4)
C(6)-C(7)-N(1)	119.2(3)
C(2)-C(7)-N(1)	120.2(4)
C(9)-C(8)-H(8A)	109.5
C(9)-C(8)-H(8B)	109.5
H(8A)-C(8)-H(8B)	109.5
C(9)-C(8)-H(8C)	109.5
H(8A)-C(8)-H(8C)	109.5
H(8B)-C(8)-H(8C)	109.5
C(10)-C(9)-N(1)	106.8(3)
C(10)-C(9)-C(8)	131.8(3)

N(1)-C(9)-C(8)	121.3(3)
C(9)-C(10)-C(11)	108.6(3)
C(9)-C(10)-H(10)	125.7
C(11)-C(10)-H(10)	125.7
C(12)-C(11)-C(10)	107.6(3)
C(12)-C(11)-H(11)	126.2
C(10)-C(11)-H(11)	126.2
C(11)-C(12)-N(1)	107.5(3)
C(11)-C(12)-C(13)	132.0(3)
N(1)-C(12)-C(13)	120.4(3)
C(12)-C(13)-C(14)	112.1(3)
C(12)-C(13)-H(13A)	109.2
C(14)-C(13)-H(13A)	109.2
C(12)-C(13)-H(13B)	109.2
C(14)-C(13)-H(13B)	109.2
H(13A)-C(13)-H(13B)	107.9
C(15)-C(14)-C(13)	113.3(3)
C(15)-C(14)-H(14A)	108.9
C(13)-C(14)-H(14A)	108.9
C(15)-C(14)-H(14B)	108.9
C(13)-C(14)-H(14B)	108.9
H(14A)-C(14)-H(14B)	107.7
O(1)-C(15)-N(2)	123.5(3)
O(1)-C(15)-C(14)	122.0(3)
N(2)-C(15)-C(14)	114.4(3)
C(21)-C(16)-C(17)	120.4(3)
C(21)-C(16)-N(2)	123.0(3)
C(17)-C(16)-N(2)	116.6(3)
C(18)-C(17)-C(16)	119.9(4)
C(18)-C(17)-H(17)	120
C(16)-C(17)-H(17)	120
C(19)-C(18)-C(17)	119.0(4)
C(19)-C(18)-H(18)	120.5
C(17)-C(18)-H(18)	120.5
C(20)-C(19)-C(18)	121.4(3)
C(20)-C(19)-Br(1)	119.2(4)
C(18)-C(19)-Br(1)	119.4(3)

C(19)-C(20)-C(21)	120.0(4)
C(19)-C(20)-H(20)	120
C(21)-C(20)-H(20)	120
C(20)-C(21)-C(16)	119.2(4)
C(20)-C(21)-H(21)	120.4
C(16)-C(21)-H(21)	120.4
F(6)-C(22)-F(4)	105.5(4)
F(6)-C(22)-F(5)	105.0(4)
F(4)-C(22)-F(5)	106.2(4)
F(6)-C(22)-C(23)	112.9(4)
F(4)-C(22)-C(23)	112.8(5)
F(5)-C(22)-C(23)	113.7(4)
C(24)-C(23)-C(28)	118.7(5)
C(24)-C(23)-C(22)	119.7(5)
C(28)-C(23)-C(22)	121.5(4)
C(25)-C(24)-C(23)	121.0(5)
C(25)-C(24)-H(24)	119.5
C(23)-C(24)-H(24)	119.5
C(26)-C(25)-C(24)	120.5(5)
C(26)-C(25)-H(25)	119.7
C(24)-C(25)-H(25)	119.7
C(25)-C(26)-C(27)	120.2(6)
C(25)-C(26)-H(26)	119.9
C(27)-C(26)-H(26)	119.9
C(28)-C(27)-C(26)	119.4(5)
C(28)-C(27)-H(27)	120.3
C(26)-C(27)-H(27)	120.3
C(27)-C(28)-C(23)	120.1(4)
C(27)-C(28)-N(3)	119.8(4)
C(23)-C(28)-N(3)	120.0(4)
C(30)-C(29)-H(29A)	109.5
C(30)-C(29)-H(29B)	109.5
H(29A)-C(29)-H(29B)	109.5
C(30)-C(29)-H(29C)	109.5
H(29A)-C(29)-H(29C)	109.5
H(29B)-C(29)-H(29C)	109.5
C(31)-C(30)-N(3)	106.7(3)

C(31)-C(30)-C(29)	131.2(4)
N(3)-C(30)-C(29)	122.0(4)
C(30)-C(31)-C(32)	108.4(3)
C(30)-C(31)-H(31)	125.8
C(32)-C(31)-H(31)	125.8
C(33)-C(32)-C(31)	107.9(3)
C(33)-C(32)-H(32)	126
C(31)-C(32)-H(32)	126
C(32)-C(33)-N(3)	107.3(3)
C(32)-C(33)-C(34)	131.4(3)
N(3)-C(33)-C(34)	121.2(3)
C(33)-C(34)-C(35)	111.7(3)
C(33)-C(34)-H(34A)	109.3
C(35)-C(34)-H(34A)	109.3
C(33)-C(34)-H(34B)	109.3
C(35)-C(34)-H(34B)	109.3
H(34A)-C(34)-H(34B)	107.9
C(36)-C(35)-C(34)	114.6(3)
C(36)-C(35)-H(35A)	108.6
C(34)-C(35)-H(35A)	108.6
C(36)-C(35)-H(35B)	108.6
C(34)-C(35)-H(35B)	108.6
H(35A)-C(35)-H(35B)	107.6
O(2)-C(36)-N(4)	121.9(3)
O(2)-C(36)-C(35)	122.9(3)
N(4)-C(36)-C(35)	115.1(3)
C(42)-C(37)-C(38)	120.2(3)
C(42)-C(37)-N(4)	121.4(3)
C(38)-C(37)-N(4)	118.4(3)
C(39)-C(38)-C(37)	119.9(3)
C(39)-C(38)-H(38)	120
C(37)-C(38)-H(38)	120
C(40)-C(39)-C(38)	119.2(4)
C(40)-C(39)-H(39)	120.4
C(38)-C(39)-H(39)	120.4
C(41)-C(40)-C(39)	121.6(3)
C(41)-C(40)-Br(2)	119.2(3)

C(39)-C(40)-Br(2)	119.1(3)
C(40)-C(41)-C(42)	119.3(4)
C(40)-C(41)-H(41)	120.3
C(42)-C(41)-H(41)	120.3
C(37)-C(42)-C(41)	119.8(4)
C(37)-C(42)-H(42)	120.1
C(41)-C(42)-H(42)	120.1
C(12)-N(1)-C(9)	109.4(3)
C(12)-N(1)-C(7)	124.3(3)
C(9)-N(1)-C(7)	126.2(3)
C(15)-N(2)-C(16)	128.3(3)
C(15)-N(2)-H(2N)	115(3)
C(16)-N(2)-H(2N)	116(3)
C(33)-N(3)-C(30)	109.6(3)
C(33)-N(3)-C(28)	125.7(3)
C(30)-N(3)-C(28)	124.6(3)
C(36)-N(4)-C(37)	125.0(3)
C(36)-N(4)-H(4N)	117(3)
C(37)-N(4)-H(4N)	116(3)

Table S7. Anisotropic displacement parameters ($Å^2 \times 10^3$) for **4j**. The anisotropic displacement factor exponent takes the form: $-2\pi^2[h^2 a^{*2} U^{11} + ... + 2 h k a^* b^* U^{12}]$.

Atom	U^{11}	U^{22}	U^{33}	U^{23}	U^{13}	U^{12}
C(1)	66(3)	40(2)	37(2)	-9(2)	16(2)	-25(2)
C(2)	61(3)	27(2)	18(2)	-5(1)	1(2)	-9(2)
C(3)	108(5)	24(2)	22(2)	4(1)	-12(2)	-12(2)
C(4)	110(5)	25(2)	38(2)	-4(2)	-31(3)	20(2)
C(5)	60(3)	46(2)	35(2)	-12(2)	-11(2)	28(2)
C(6)	42(2)	38(2)	26(2)	-3(2)	-1(2)	12(2)
C(7)	43(2)	22(2)	16(1)	-2(1)	0(1)	3(1)
C(8)	52(3)	28(2)	32(2)	-10(2)	-2(2)	2(2)
C(9)	32(2)	22(2)	29(2)	-4(1)	2(1)	0(1)
C(10)	36(2)	19(2)	38(2)	-4(1)	-3(2)	2(1)
C(11)	35(2)	24(2)	29(2)	2(1)	-7(2)	1(1)
C(12)	34(2)	21(2)	23(2)	0(1)	1(1)	-1(1)

C(13)	54(3)	23(2)	20(2)	2(1)	-2(2)	5(2)
C(14)	51(3)	24(2)	22(2)	0(1)	-1(2)	-2(2)
C(15)	34(2)	23(2)	21(2)	-4(1)	-1(1)	-1(1)
C(16)	25(2)	26(2)	20(1)	2(1)	-1(1)	-7(1)
C(17)	37(2)	32(2)	27(2)	0(1)	-3(2)	2(2)
C(18)	58(3)	32(2)	34(2)	6(2)	-9(2)	5(2)
C(19)	75(3)	37(2)	24(2)	16(2)	-9(2)	-17(2)
C(20)	47(3)	53(3)	31(2)	11(2)	6(2)	-15(2)
C(21)	30(2)	47(2)	28(2)	8(2)	2(2)	-5(2)
C(22)	86(4)	43(2)	35(2)	-5(2)	-10(2)	27(3)
C(23)	90(4)	28(2)	20(2)	-5(1)	6(2)	9(2)
C(24)	135(6)	31(2)	30(2)	-2(2)	23(3)	15(3)
C(25)	149(7)	28(2)	40(2)	-3(2)	42(3)	-13(3)
C(26)	94(4)	54(3)	43(3)	-19(2)	34(3)	-36(3)
C(27)	68(3)	39(2)	31(2)	-9(2)	12(2)	-16(2)
C(28)	60(3)	23(2)	20(2)	-4(1)	6(2)	-9(2)
C(29)	65(3)	27(2)	35(2)	-12(2)	0(2)	2(2)
C(30)	45(2)	24(2)	36(2)	-8(2)	-4(2)	-1(2)
C(31)	51(3)	26(2)	53(2)	-3(2)	2(2)	-7(2)
C(32)	50(3)	29(2)	39(2)	4(2)	6(2)	-10(2)
C(33)	40(2)	30(2)	25(2)	2(1)	1(2)	-5(2)
C(34)	55(3)	32(2)	22(2)	-1(1)	5(2)	-10(2)
C(35)	28(2)	42(2)	34(2)	-4(2)	8(2)	-7(2)
C(36)	26(2)	38(2)	24(2)	6(1)	1(1)	-7(2)
C(37)	26(2)	30(2)	22(2)	6(1)	3(1)	-4(1)
C(38)	27(2)	29(2)	31(2)	7(1)	1(1)	-2(2)
C(39)	39(2)	27(2)	32(2)	4(1)	9(2)	0(2)
C(40)	51(3)	26(2)	22(2)	-4(1)	7(2)	-5(2)
C(41)	36(2)	44(2)	24(2)	0(1)	-1(2)	-3(2)
C(42)	28(2)	36(2)	25(2)	2(1)	4(1)	1(2)
N(1)	32(2)	21(1)	20(1)	0(1)	2(1)	3(1)
N(2)	24(2)	28(1)	20(1)	5(1)	2(1)	-5(1)
N(3)	42(2)	24(1)	25(1)	-3(1)	0(1)	-3(1)
N(4)	22(2)	33(2)	23(1)	4(1)	0(1)	-2(1)
O(1)	27(2)	52(2)	27(1)	2(1)	-1(1)	4(1)
O(2)	28(2)	78(2)	28(1)	-10(1)	8(1)	-15(2)
F(1)	103(3)	82(2)	52(2)	7(2)	21(2)	-57(2)

F(2)	60(2)	69(2)	72(2)	-38(2)	33(2)	-22(2)
F(3)	49(2)	53(2)	46(1)	-9(1)	5(1)	-15(1)
F(4)	118(3)	87(3)	65(2)	14(2)	-15(2)	52(2)
F(5)	74(2)	63(2)	69(2)	-35(2)	-20(2)	18(2)
F(6)	68(2)	60(2)	43(1)	-14(1)	1(1)	13(1)
Br(1)	127(1)	66(1)	49(1)	36(1)	-16(1)	-32(1)
Br(2)	72(1)	41(1)	36(1)	-11(1)	3(1)	-8(1)
C(1)	66(3)	40(2)	37(2)	-9(2)	16(2)	-25(2)

Table S8. Hydrogen coordinates (×10^4) and isotropic displacement parameters (Å^2×10^3) for **4j**.

Atom	x	y	z	U(eq)
H(3)	1019	2423	12045	62
H(4)	3470	2242	11945	70
H(5)	4818	3334	11163	57
H(6)	3667	4610	10457	43
H(8A)	2125	6212	11604	56
H(8B)	731	5725	11997	56
H(8C)	679	6811	11701	56
H(10)	-526	6978	10079	38
H(11)	-712	5912	8759	35
H(13A)	441	3494	9312	39
H(13B)	1763	4011	8865	39
H(14A)	-76	3418	7850	38
H(14B)	-1120	4220	8208	38
H(17)	-2325	6431	6409	39
H(18)	-2146	7524	5268	50
H(20)	1867	6591	4802	52
H(21)	1707	5496	5940	42
H(24)	5512	5987	2844	78
H(25)	7947	6295	2864	86
H(26)	9515	5355	3670	76
H(27)	8656	4020	4414	55
H(29A)	5840	1720	3068	63
H(29B)	7231	2373	3194	63
H(29C)	5752	2826	2848	63

H(31)	4925	1344	4690	52
H(32)	4741	2292	6087	47
H(34A)	5519	4810	5703	44
H(34B)	6908	4302	6119	44
H(35A)	4052	4159	6728	41
H(35B)	5289	3459	7069	41
H(38)	2741	6247	8727	35
H(39)	2989	7183	9983	39
H(41)	6892	6025	10489	41
H(42)	6639	5067	9240	36
H(2N)	-1340(50)	5160(30)	7140(30)	28
H(4N)	3620(50)	4910(30)	7940(30)	31

Table S9. Torsion angles [°] for **4j**.

	Angle/deg.
F(2)-C(1)-C(2)-C(3)	120.3(4)
F(3)-C(1)-C(2)-C(3)	-118.9(4)
F(1)-C(1)-C(2)-C(3)	-0.6(5)
F(2)-C(1)-C(2)-C(7)	-61.1(5)
F(3)-C(1)-C(2)-C(7)	59.7(5)
F(1)-C(1)-C(2)-C(7)	178.0(3)
C(7)-C(2)-C(3)-C(4)	-0.6(5)
C(1)-C(2)-C(3)-C(4)	178.0(3)
C(2)-C(3)-C(4)-C(5)	1.1(6)
C(3)-C(4)-C(5)-C(6)	-0.5(6)
C(4)-C(5)-C(6)-C(7)	-0.6(6)
C(5)-C(6)-C(7)-C(2)	1.2(5)
C(5)-C(6)-C(7)-N(1)	-177.2(3)
C(3)-C(2)-C(7)-C(6)	-0.6(5)
C(1)-C(2)-C(7)-C(6)	-179.1(3)
C(3)-C(2)-C(7)-N(1)	177.8(3)
C(1)-C(2)-C(7)-N(1)	-0.8(5)
N(1)-C(9)-C(10)-C(11)	-0.4(4)
C(8)-C(9)-C(10)-C(11)	177.1(4)
C(9)-C(10)-C(11)-C(12)	-0.2(5)

C(10)-C(11)-C(12)-N(1)	0.7(4)
C(10)-C(11)-C(12)-C(13)	-177.9(4)
C(11)-C(12)-C(13)-C(14)	-9.7(6)
N(1)-C(12)-C(13)-C(14)	171.8(3)
C(12)-C(13)-C(14)-C(15)	72.2(4)
C(13)-C(14)-C(15)-O(1)	42.1(5)
C(13)-C(14)-C(15)-N(2)	-141.4(3)
C(21)-C(16)-C(17)-C(18)	-0.7(5)
N(2)-C(16)-C(17)-C(18)	-178.5(3)
C(16)-C(17)-C(18)-C(19)	0.4(6)
C(17)-C(18)-C(19)-C(20)	-0.2(6)
C(17)-C(18)-C(19)-Br(1)	179.6(3)
C(18)-C(19)-C(20)-C(21)	0.2(7)
Br(1)-C(19)-C(20)-C(21)	-179.6(3)
C(19)-C(20)-C(21)-C(16)	-0.4(6)
C(17)-C(16)-C(21)-C(20)	0.7(6)
N(2)-C(16)-C(21)-C(20)	178.3(3)
F(6)-C(22)-C(23)-C(24)	-117.7(4)
F(4)-C(22)-C(23)-C(24)	1.7(5)
F(5)-C(22)-C(23)-C(24)	122.7(4)
F(6)-C(22)-C(23)-C(28)	59.9(5)
F(4)-C(22)-C(23)-C(28)	179.3(3)
F(5)-C(22)-C(23)-C(28)	-59.7(5)
C(28)-C(23)-C(24)-C(25)	-2.0(6)
C(22)-C(23)-C(24)-C(25)	175.6(4)
C(23)-C(24)-C(25)-C(26)	0.2(7)
C(24)-C(25)-C(26)-C(27)	1.9(7)
C(25)-C(26)-C(27)-C(28)	-2.3(6)
C(26)-C(27)-C(28)-C(23)	0.4(5)
C(26)-C(27)-C(28)-N(3)	178.4(3)
C(24)-C(23)-C(28)-C(27)	1.7(5)
C(22)-C(23)-C(28)-C(27)	-176.0(3)
C(24)-C(23)-C(28)-N(3)	-176.3(3)
C(22)-C(23)-C(28)-N(3)	6.1(5)
N(3)-C(30)-C(31)-C(32)	-0.1(5)
C(29)-C(30)-C(31)-C(32)	176.2(4)
C(30)-C(31)-C(32)-C(33)	-0.6(5)

C(31)-C(32)-C(33)-N(3)	1.0(5)
C(31)-C(32)-C(33)-C(34)	-176.0(5)
C(32)-C(33)-C(34)-C(35)	-14.5(7)
N(3)-C(33)-C(34)-C(35)	168.8(4)
C(33)-C(34)-C(35)-C(36)	168.3(3)
C(34)-C(35)-C(36)-O(2)	-32.1(6)
C(34)-C(35)-C(36)-N(4)	150.8(3)
C(42)-C(37)-C(38)-C(39)	0.9(5)
N(4)-C(37)-C(38)-C(39)	179.3(3)
C(37)-C(38)-C(39)-C(40)	0.5(5)
C(38)-C(39)-C(40)-C(41)	-1.5(5)
C(38)-C(39)-C(40)-Br(2)	176.3(3)
C(39)-C(40)-C(41)-C(42)	1.0(6)
Br(2)-C(40)-C(41)-C(42)	-176.8(3)
C(38)-C(37)-C(42)-C(41)	-1.4(5)
N(4)-C(37)-C(42)-C(41)	-179.7(3)
C(40)-C(41)-C(42)-C(37)	0.5(5)
C(11)-C(12)-N(1)-C(9)	-1.0(4)
C(13)-C(12)-N(1)-C(9)	177.8(3)
C(11)-C(12)-N(1)-C(7)	175.7(3)
C(13)-C(12)-N(1)-C(7)	-5.4(5)
C(10)-C(9)-N(1)-C(12)	0.9(4)
C(8)-C(9)-N(1)-C(12)	-176.9(4)
C(10)-C(9)-N(1)-C(7)	-175.8(3)
C(8)-C(9)-N(1)-C(7)	6.4(6)
C(6)-C(7)-N(1)-C(12)	89.3(4)
C(2)-C(7)-N(1)-C(12)	-89.0(4)
C(6)-C(7)-N(1)-C(9)	-94.5(4)
C(2)-C(7)-N(1)-C(9)	87.1(4)
O(1)-C(15)-N(2)-C(16)	-0.4(5)
C(14)-C(15)-N(2)-C(16)	-176.8(3)
C(21)-C(16)-N(2)-C(15)	23.0(5)
C(17)-C(16)-N(2)-C(15)	-159.3(3)
C(32)-C(33)-N(3)-C(30)	-1.2(5)
C(34)-C(33)-N(3)-C(30)	176.2(4)
C(32)-C(33)-N(3)-C(28)	176.3(4)
C(34)-C(33)-N(3)-C(28)	-6.3(6)

C(31)-C(30)-N(3)-C(33)	0.8(5)
C(29)-C(30)-N(3)-C(33)	-176.0(4)
C(31)-C(30)-N(3)-C(28)	-176.7(4)
C(29)-C(30)-N(3)-C(28)	6.5(6)
C(27)-C(28)-N(3)-C(33)	89.5(5)
C(23)-C(28)-N(3)-C(33)	-92.5(5)
C(27)-C(28)-N(3)-C(30)	-93.4(5)
C(23)-C(28)-N(3)-C(30)	84.5(5)
O(2)-C(36)-N(4)-C(37)	-3.8(6)
C(35)-C(36)-N(4)-C(37)	173.3(3)
C(42)-C(37)-N(4)-C(36)	-40.8(5)
C(38)-C(37)-N(4)-C(36)	140.9(4)

Table S10. Hydrogen bonds for **4j** [Å and °].

D-H...A	d(D-H)	d(H...A)	d(D...A)	<(DHA)
N(2)-H(2N)...O(2)#1	0.81(4)	1.96(4)	2.771(4)	175(4)
N(4)-H(4N)...O(1)	0.85(4)	1.93(5)	2.763(4)	167(4)

Symmetry transformations used to generate equivalent atoms: #1 *x*–1, *y*, *z*

13. References

[1] J. Ma, X. Zhang, X. Huang, S. Luo, E. Meggers, *Nat. Protoc.* **2018**, *13*, 605-632.

[2] H. Yan, J.-S. Oha, C. E. Song, *Org. Biomol. Chem.* **2011**, *9*, 8119-8121.

[3] M. H. Palmer, D. S. Leitch, C. W. Greenhalgh, *Tetrahedron* **1978**, *34*, 1015-1021.

[4] T. P. Smyth, B. W. Corby, *J. Org. Chem.* **1998**, *63*, 8946-8951.

[5] Gaussian 16, Revision C.01, M. J. Frisch, G. W. Trucks, H. B. Schlegel, G. E. Scuseria, M. A. Robb, J. R. Cheeseman, G. Scalmani, V. Barone, G. A. Petersson, H. Nakatsuji, X. Li, M. Caricato, A. V. Marenich, J. Bloino, B. G. Janesko, R. Gomperts, B. Mennucci, H. P. Hratchian, J. V. Ortiz, A. F. Izmaylov, J. L. Sonnenberg, D. Williams-Young, F. Ding, F. Lipparini, F. Egidi, J. Goings, B. Peng, A. Petrone, T. Henderson, D. Ranasinghe, V. G. Zakrzewski, J. Gao, N. Rega, G. Zheng, W. Liang, M. Hada, M. Ehara, K. Toyota, R. Fukuda, J. Hasegawa, M. Ishida, T. Nakajima, Y. Honda,

O. Kitao, H. Nakai, T. Vreven, K. Throssell, J. A. Montgomery, Jr., J. E. Peralta, F. Ogliaro, M. J. Bearpark, J. J. Heyd, E. N. Brothers, K. N. Kudin, V. N. Staroverov, T. A. Keith, R. Kobayashi, J. Normand, K. Raghavachari, A. P. Rendell, J. C. Burant, S. S. Iyengar, J. Tomasi, M. Cossi, J. M. Millam, M. Klene, C. Adamo, R. Cammi, J. W. Ochterski, R. L. Martin, K. Morokuma, O. Farkas, J. B. Foresman, and D. J. Fox, Gaussian, Inc., Wallingford CT, **2019**.

[6] (a) Head-Gordon, M.; Pople, J. A.; Frisch, M. J. MP2 Energy Evaluation by Direct Methods. *Chem. Phys. Lett.* **1988**, *153*, 503. (b) Becke, A. D. Density-Functional Thermochemistry. III. The Role of Exact Exchange. *J. Chem. Phys.* **1993**, *98*, 5648. (c) Lee, C.; Yang, W.; Parr, R. G. Development of the Colle-Salvetti Correlation-Energy Formula into a Functional of the Electron Density. *Phys. Rev. B: Condens. Matter Mater. Phys.* **1988**, *37*, 785. (d) Vosko, S. H.; Wilk, L.; Nusair, M. Accurate Spin-Dependent Electron Liquid Correlation Energies for Local Spin Density Calculations: a Critical Analysis. *Can. J. Phys.* **1980**, *58*, 1200. (e) Stephens, P. J.; Devlin, F. J.; Chabalowski, C. F.; Frisch, M. J. Ab Initio Calculation of Vibrational Absorption and Circular Dichroism Spectra Using Density Functional Force Fields. *J. Phys. Chem.* **1994**, *98*, 11623.

[7] Grimme, S.; Antony, J.; Ehrlich, S.; Krieg, H. A Consistent and Accurate ab initio Parametrization of Density Functional Dispersion Correction (DFT-D) for the 94 Elements H-Pu. *J. Chem. Phys.* **2010**, *132*, 154104.

[8] (BJ damping) Grimme, S.; Ehrlich, S.; Goerigk, L. Effect of the Damping Function in Dispersion Corrected Density Functional Theory. *J. Comput. Chem.* **2011**, *32*, 1456.

[9] Hay, P. J.; Wadt, W. R. Ab initio Effective Core Potentials for Molecular Calculations. Potentials for K to Au Including the Outermost Core Orbitals. *J. Chem. Phys.* **1985**, *82*, 299.

[10] Grimme, S., Supramolecular Binding Thermodynamics by Dispersion-Corrected Density Functional Theory. *Chem. Eur. J.* **2012**, *18*, 9955.

[11] Zhao, Y.; Truhlar, D. G. The M06 Suite of Density Functionals for Main Group Thermochemistry, Thermochemical Kinetics, Noncovalent Interactions, Excited States, and Transition Elements: Two New Functionals and Systematic Testing of Four M06-Class.

[12] (a) Haussermann, U.; Dolg, M.; Stoll, H.; Preuss, H.; Schwerdtfeger, P.; Pitzer, R. M. Accuracy of Energy-Adjusted Quasirelativistic Ab initio Pseudopotentials. *Mol. Phys.* **1993**, *78*, 1211. (b) K chle, W.; Dolg, M.; Stoll, H.; Preuss, H. Energy-Adjusted Pseudopotentials for the Actinides. Parameter Sets and Test Calculations for Thorium and Thorium Monoxide. *J. Chem. Phys.* **1994**, *100*, 7535.

[13] Marenich, A. V.; Cramer, C. J.; Truhlar, D. G. Universal Solvation Model Based on Solute Electron Density and on a Continuum Model of the Solvent Defined by the Bulk Dielectric Constant and Atomic Surface Tensions. *J. Phys. Chem. B* **2009**, *113*, 6378.

[14] Legault, C. Y. CYLview, 1.0b; Université de Sherbrooke, **2009**; http://www.cylview.org.

[15] Spartan '18, Wavefunction, Inc. Irvine, CA.

[16] *X-Area*, Stoe & Cie GmbH, Darmstadt, Germany, **2018**.

[17] *X-RED32*, Stoe & Cie GmbH, Darmstadt, Germany, **2018**.

[18] *LANA-Laue Analyzer*, Stoe & Cie GmbH, Darmstadt, Germany, **2019**.

[19] G. M. Sheldrick, *Acta Crystallogr., Sect. A: Found. Adv.* **2015**, *71*, 3-8.

[20] G. M. Sheldrick, *Acta Crystallogr., Sect. C: Struct. Chem.* **2015**, *71*, 3-8.

[21] C. B. Hübschle, G. M. Sheldrick, B. Dittrich, *J. Appl. Crystallogr.* **2011**, *44*, 1281-1284.

14. NMR and HPLC Spectra

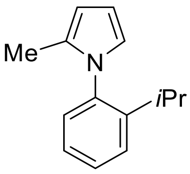

1a

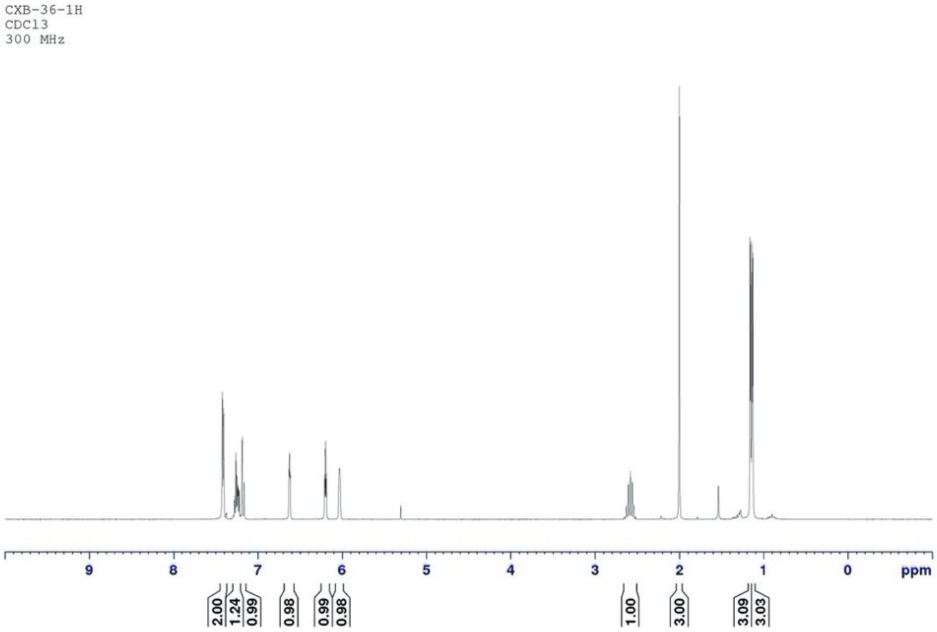

Figure S18. ^{1}H NMR ($CDCl_3$) of compound **1a**.

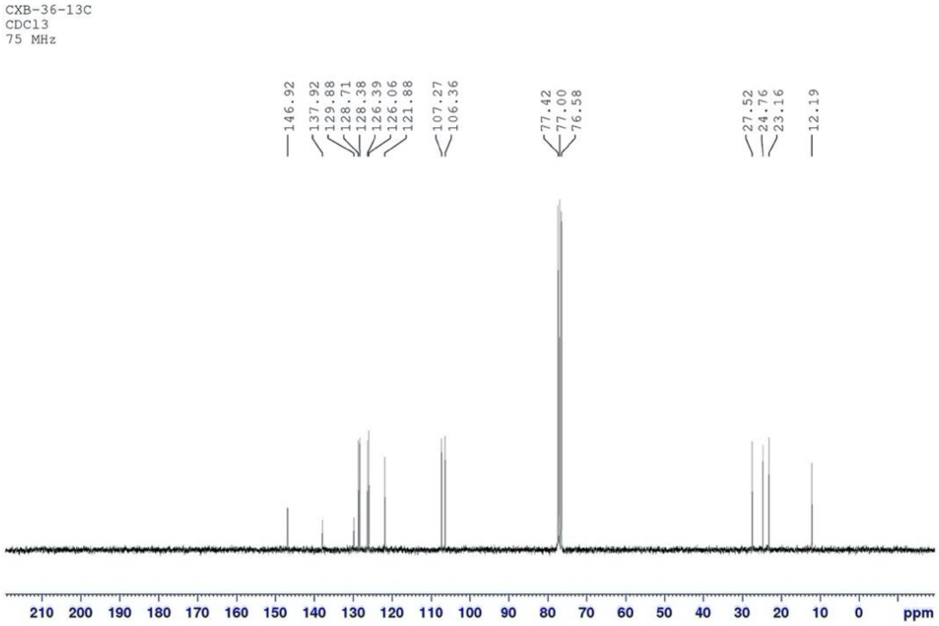

Figure S19. ^{13}C NMR ($CDCl_3$) of compound **1a**.

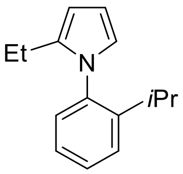

1b

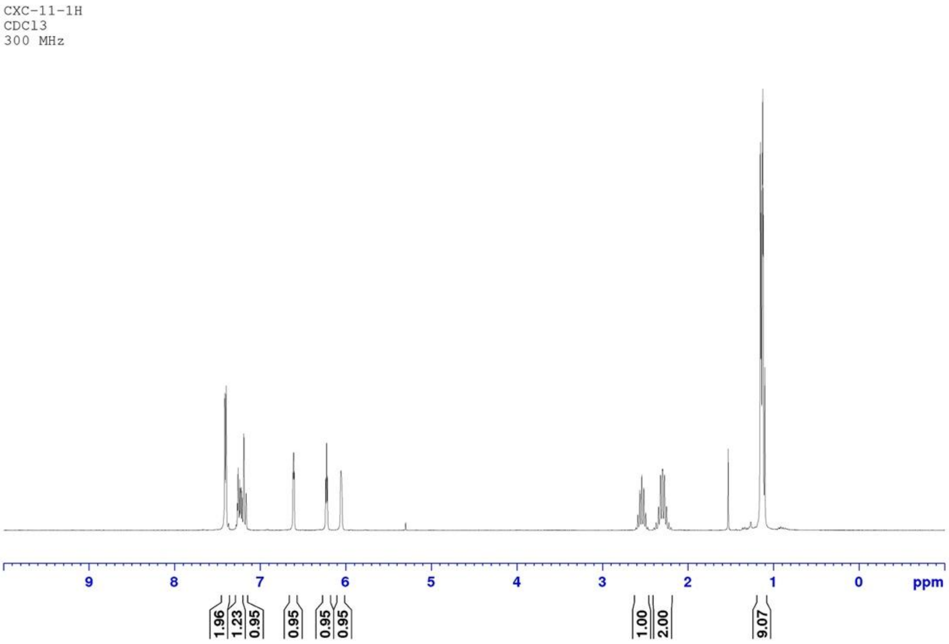

Figure S20. ^{1}H NMR ($CDCl_3$) of compound **1b**.

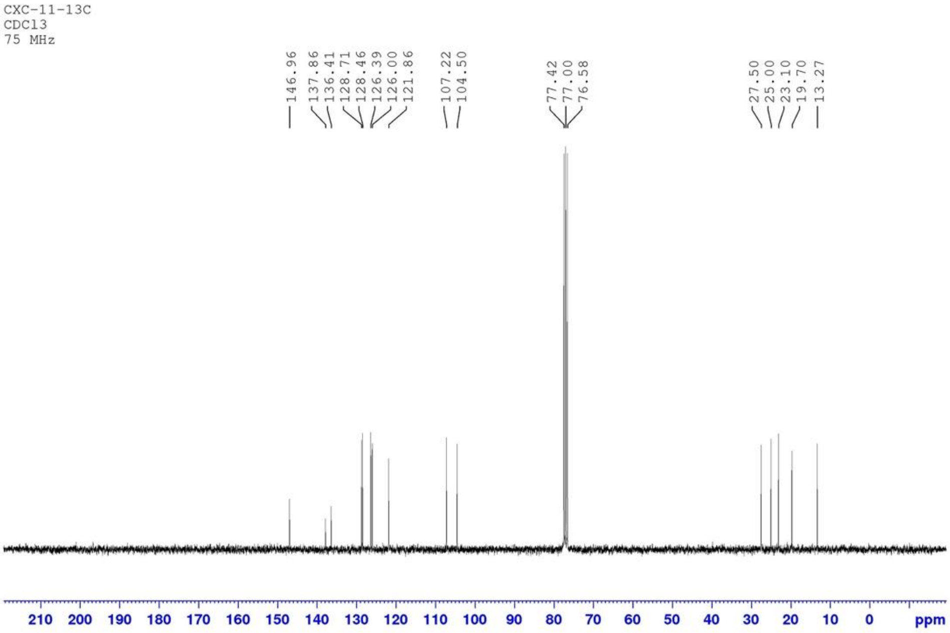

Figure S21. ^{13}C NMR ($CDCl_3$) of compound **1b**.

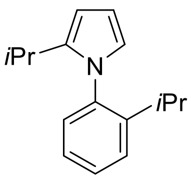

1c

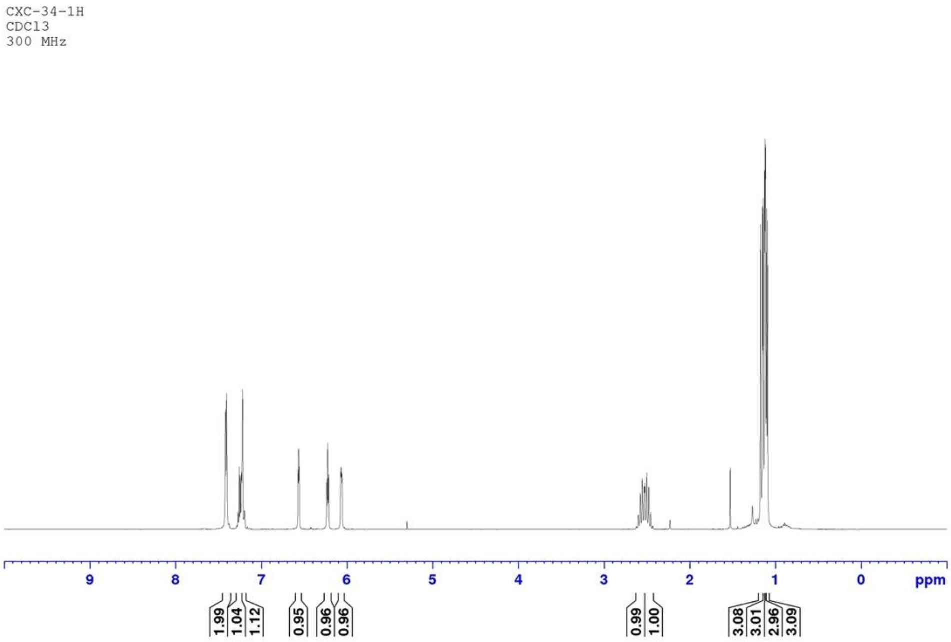

Figure S22. ^{1}H NMR ($CDCl_3$) of compound **1c**.

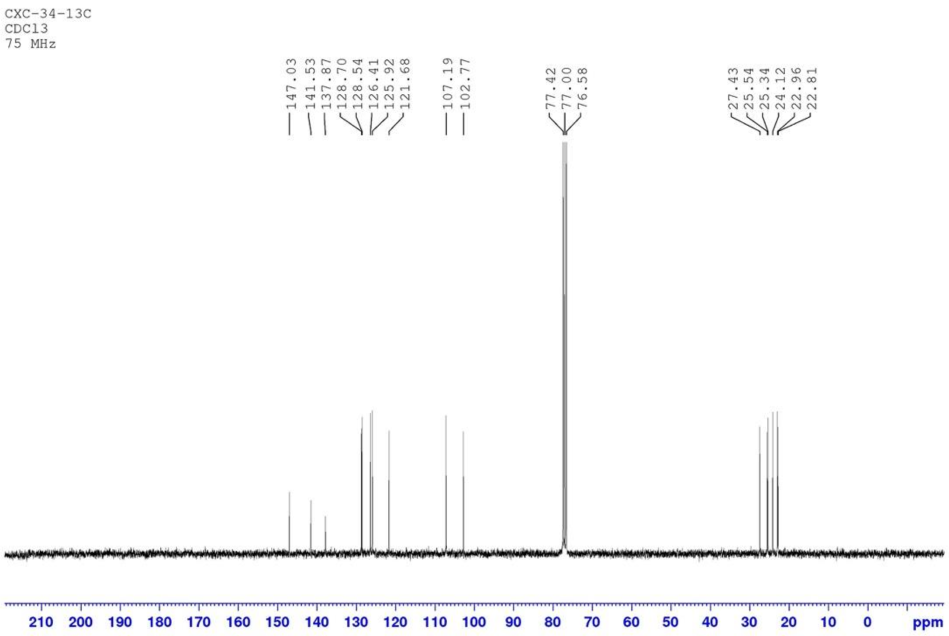

Figure S23. ^{13}C NMR ($CDCl_3$) of compound **1c**.

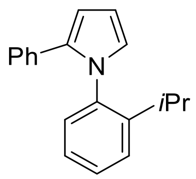

1d

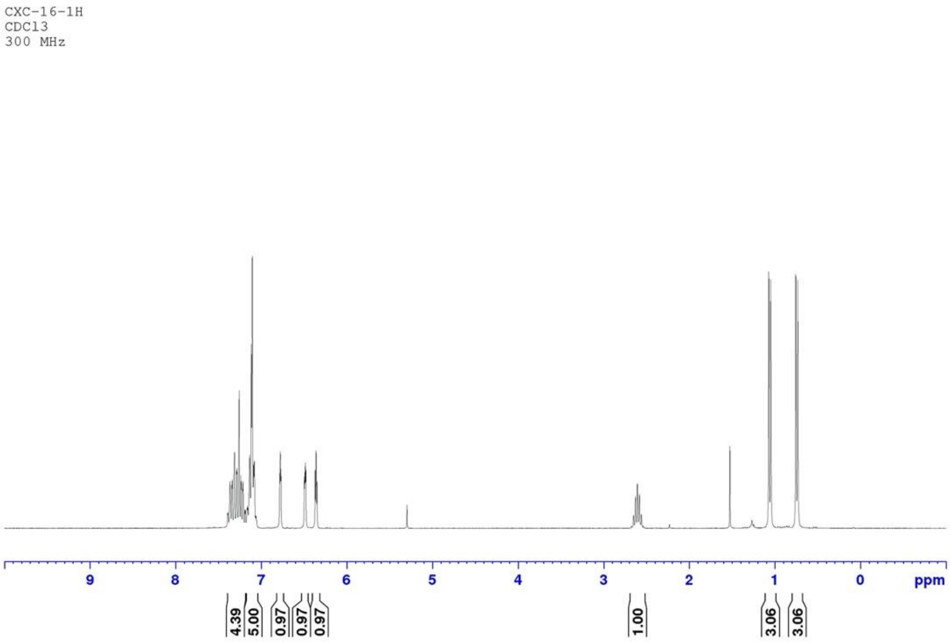

Figure S24. ^{1}H NMR ($CDCl_3$) of compound **1d**.

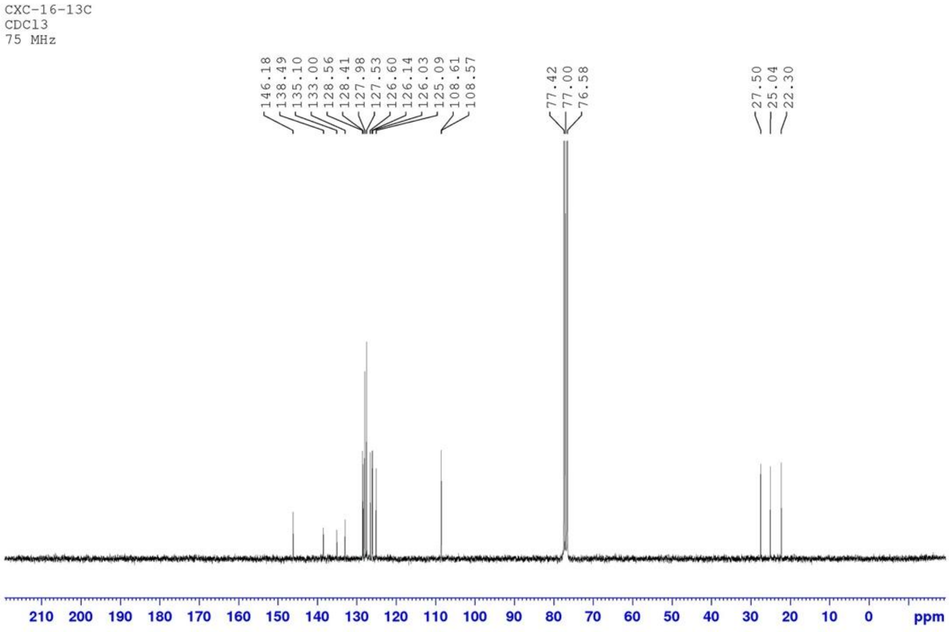

Figure S25. ^{13}C NMR ($CDCl_3$) of compound **1d**.

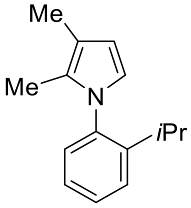

1e

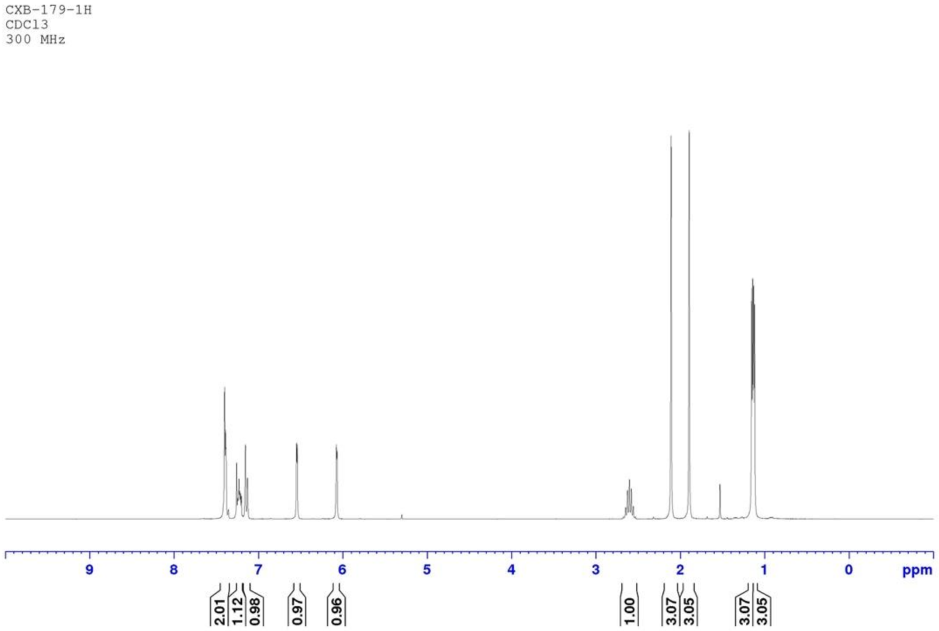

Figure S26. ^{1}H NMR ($CDCl_3$) of compound **1e**.

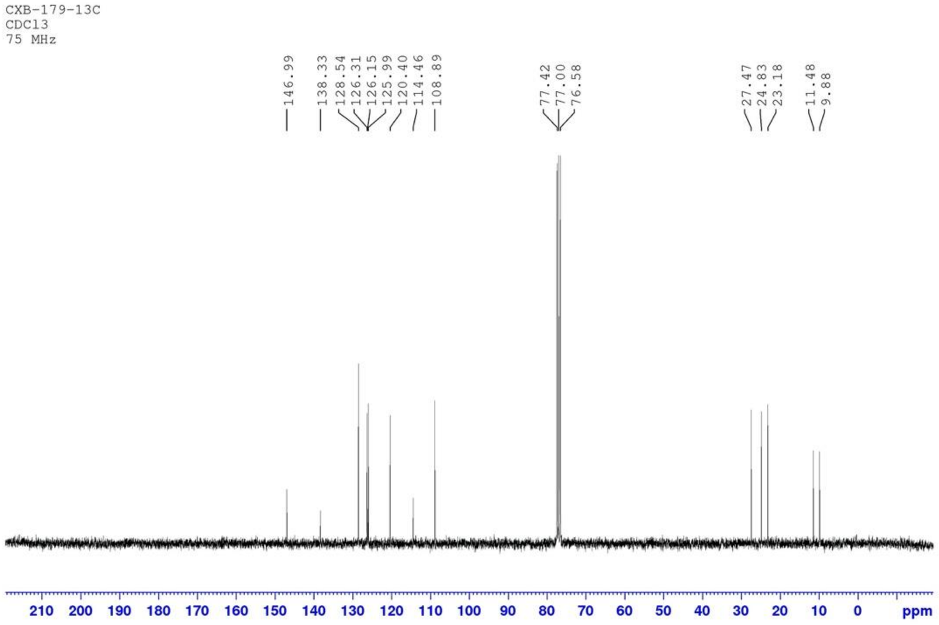

Figure S27. ^{13}C NMR ($CDCl_3$) of compound **1e**.

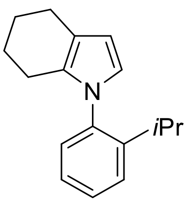

1f

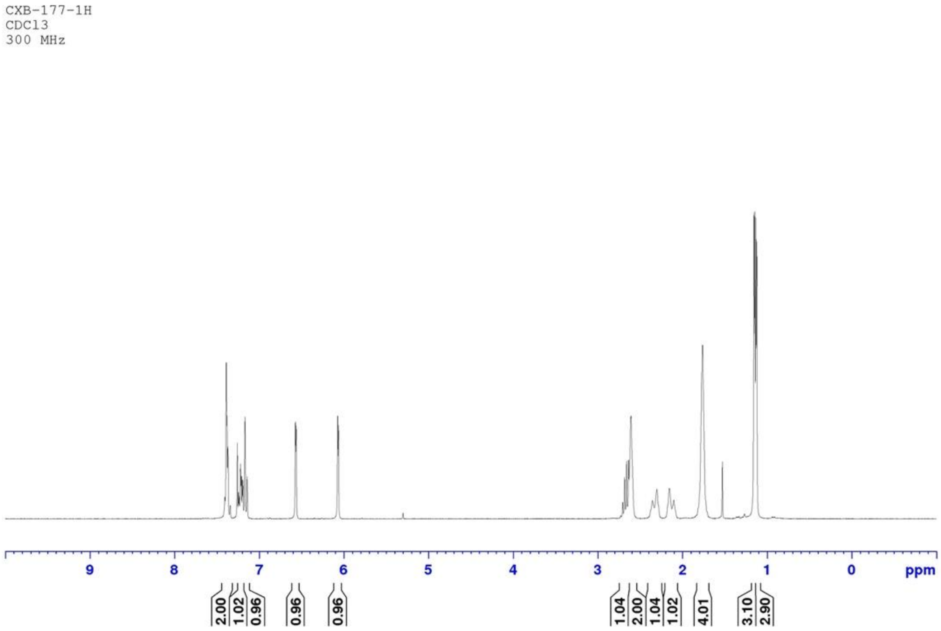

Figure S28. ^{1}H NMR ($CDCl_3$) of compound **1f**.

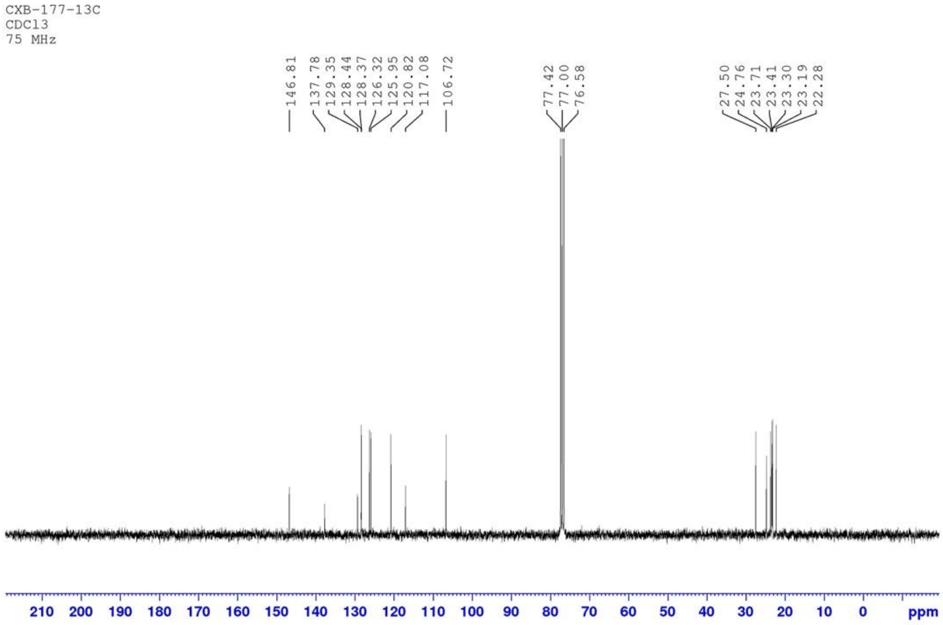

Figure S29. ^{13}C NMR ($CDCl_3$) of compound **1f**.

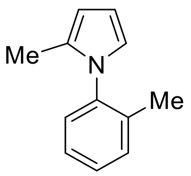

1g

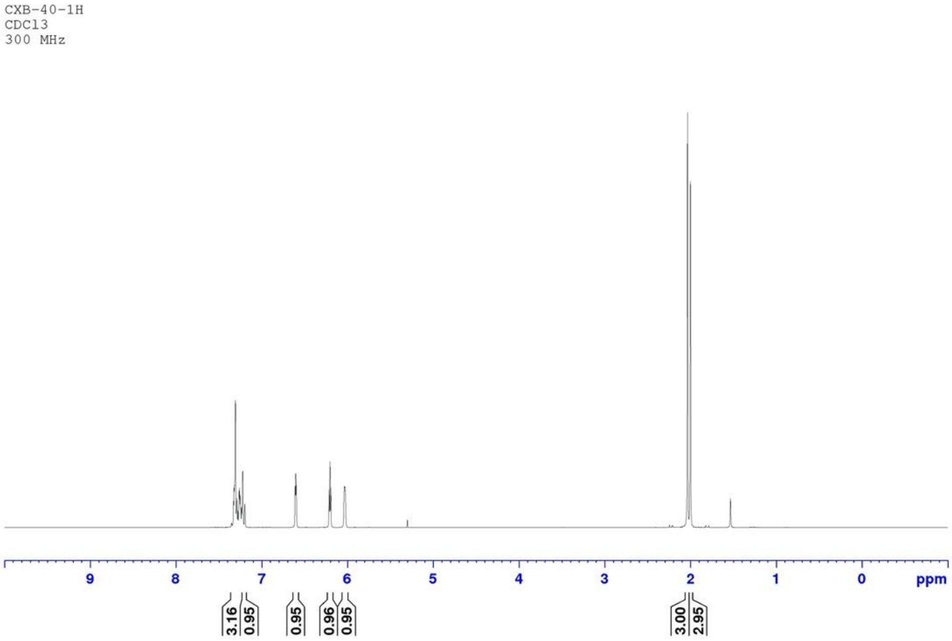

Figure S30. ^{1}H NMR ($CDCl_3$) of compound **1g**.

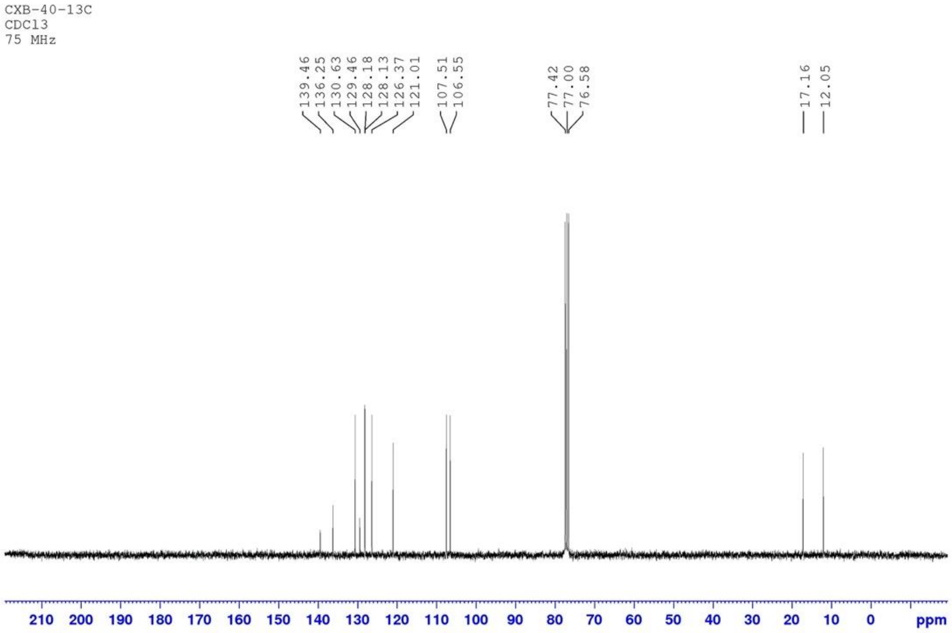

Figure S31. ^{13}C NMR ($CDCl_3$) of compound **1g**.

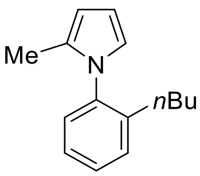

1h

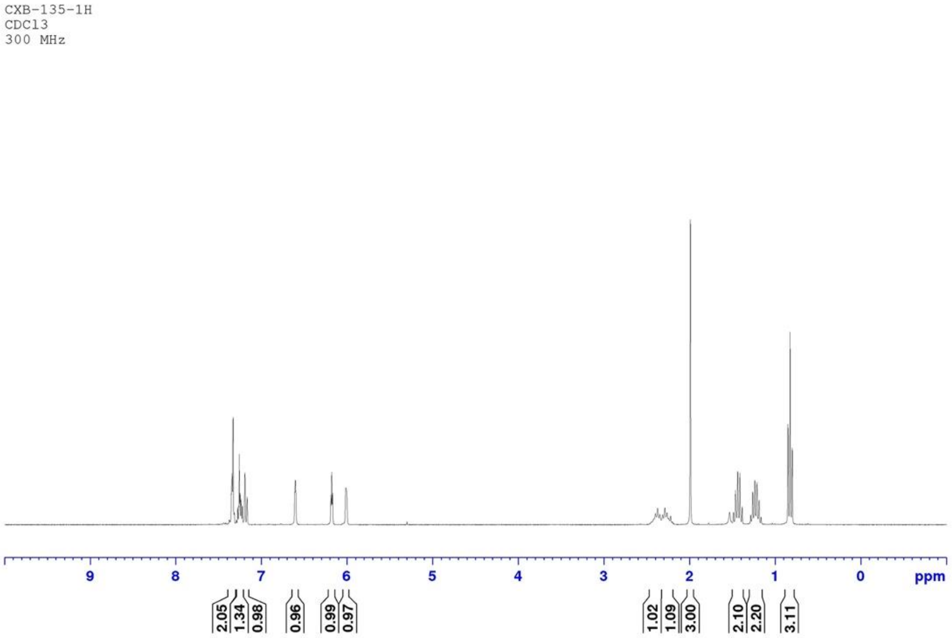

Figure S32. ^{1}H NMR ($CDCl_3$) of compound **1h**.

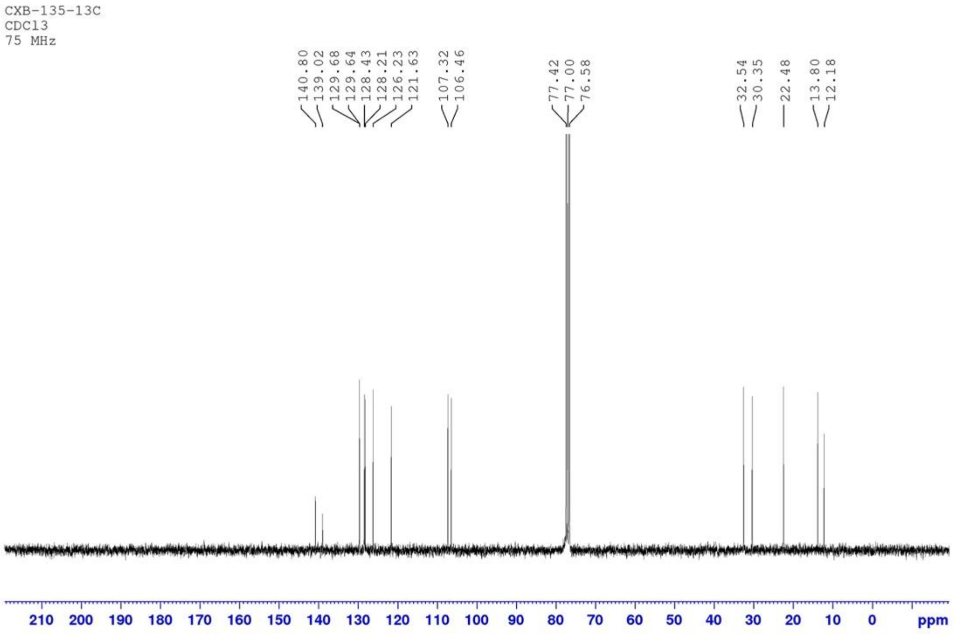

Figure S33. ^{13}C NMR ($CDCl_3$) of compound **1h**.

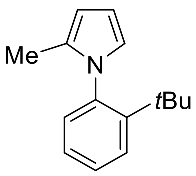

1i

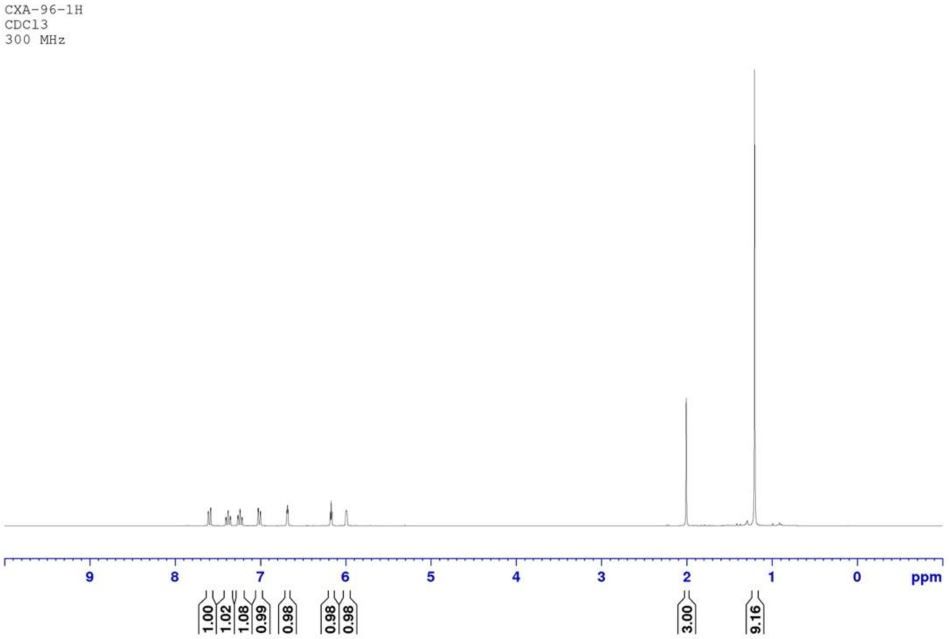

Figure S34. ^{1}H NMR ($CDCl_3$) of compound **1i**.

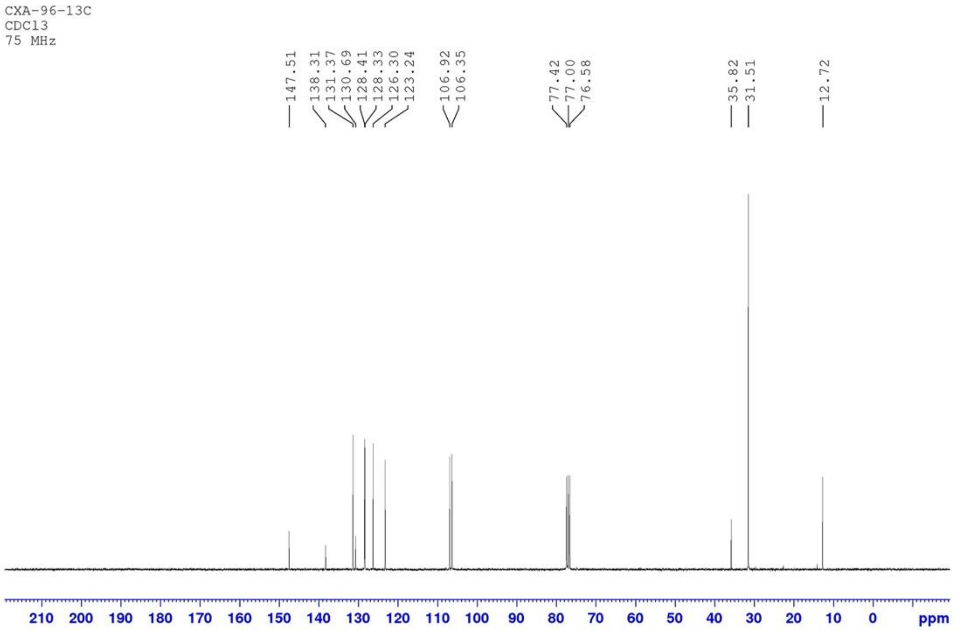

Figure S35. ^{13}C NMR ($CDCl_3$) of compound **1i**.

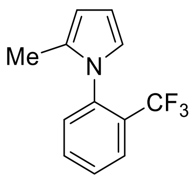

1j

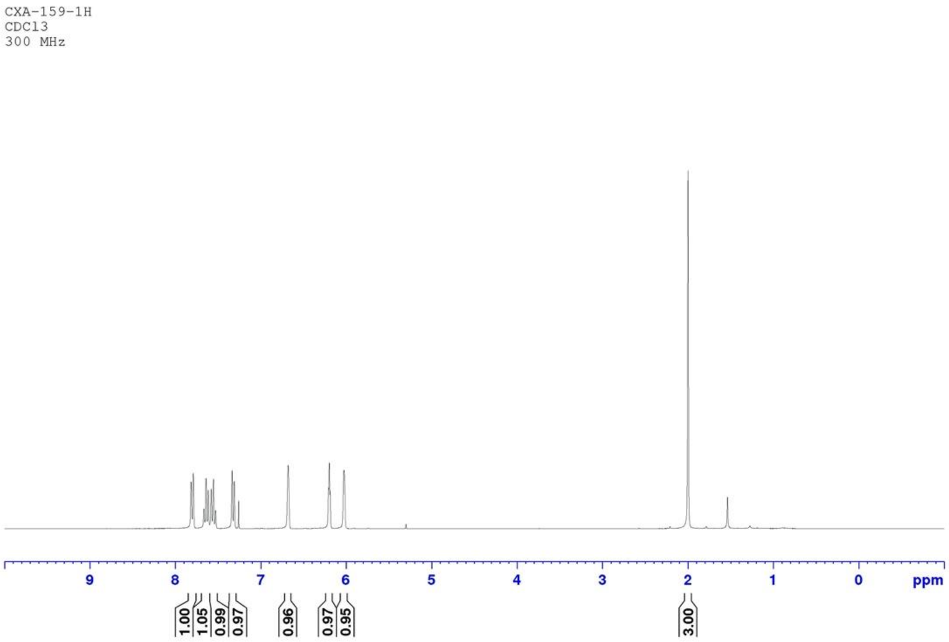

Figure S36. ^{1}H NMR ($CDCl_3$) of compound **1j**.

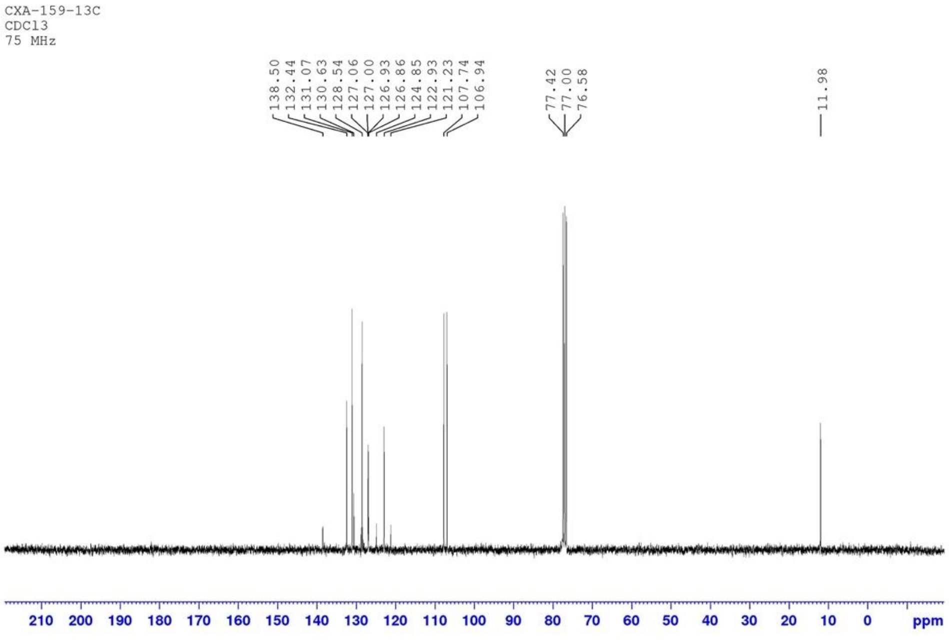

Figure S37. ^{13}C NMR ($CDCl_3$) of compound **1j**.

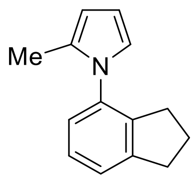

1k

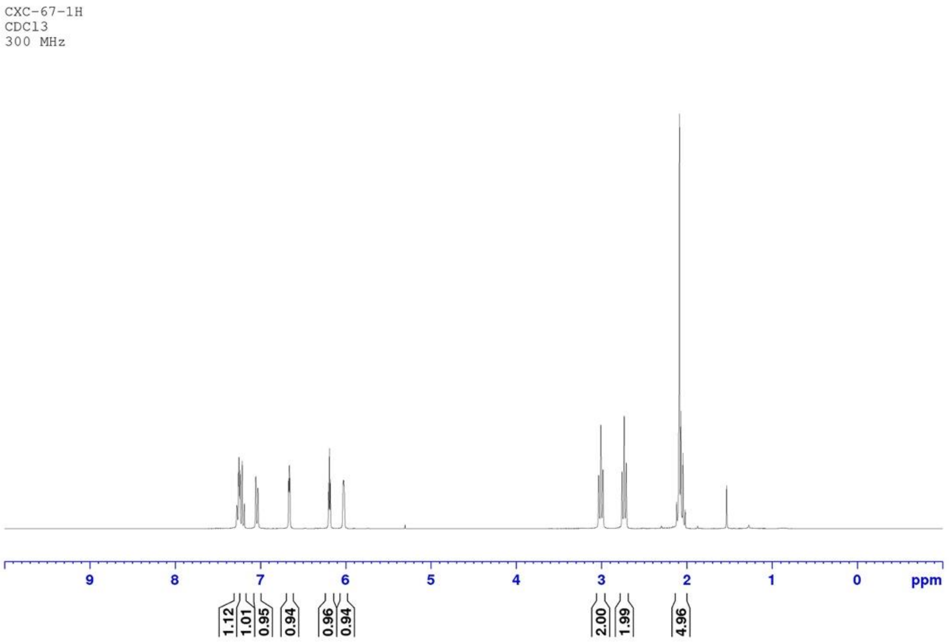

Figure S38. ^{1}H NMR ($CDCl_3$) of compound **1k**.

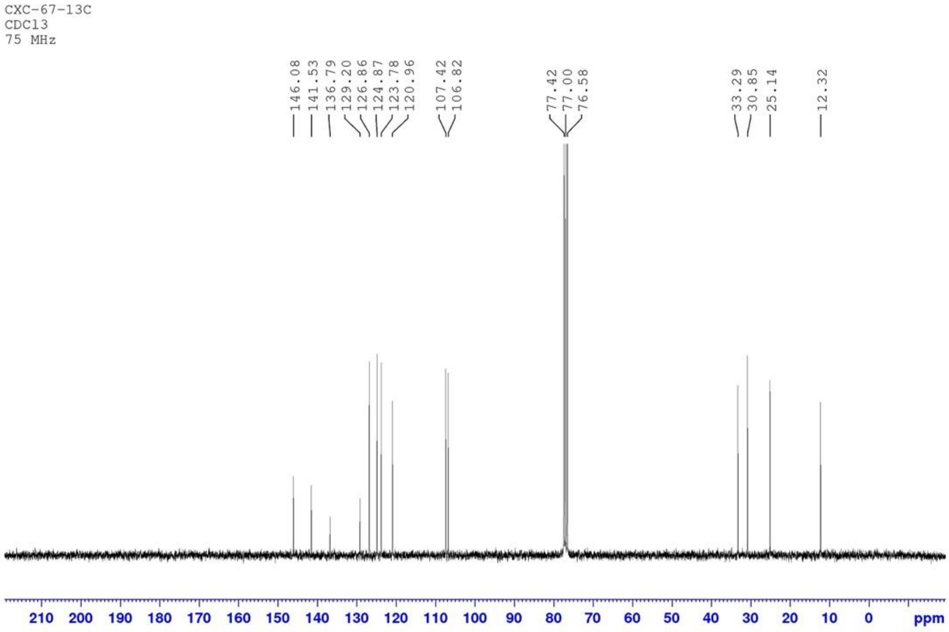

Figure S39. ^{13}C NMR ($CDCl_3$) of compound **1k**.

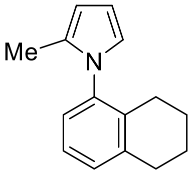

1l

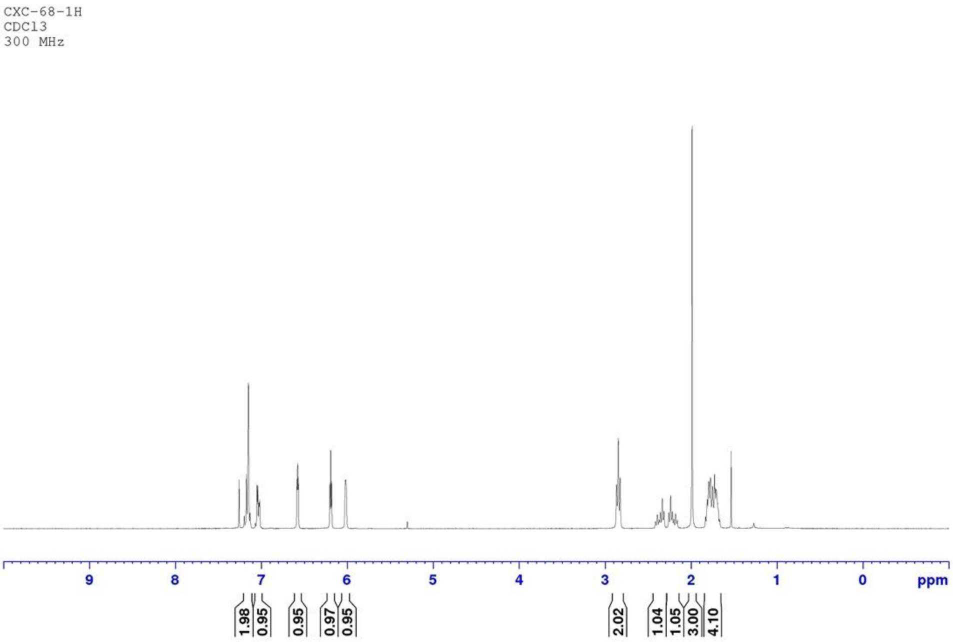

Figure S40. ^{1}H NMR ($CDCl_3$) of compound **1l**.

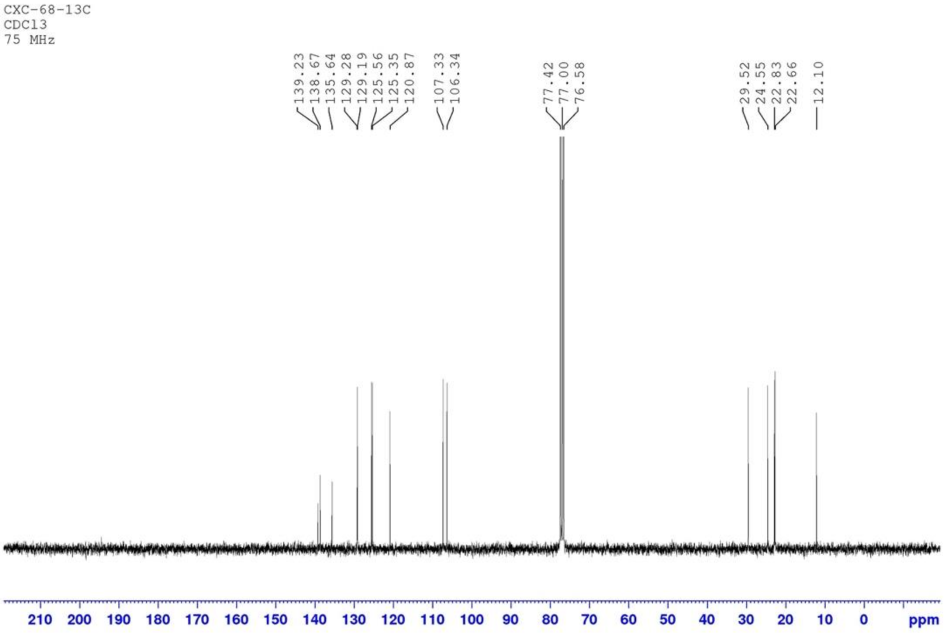

Figure S41. ^{13}C NMR ($CDCl_3$) of compound **1l**.

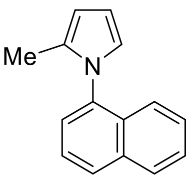

1m

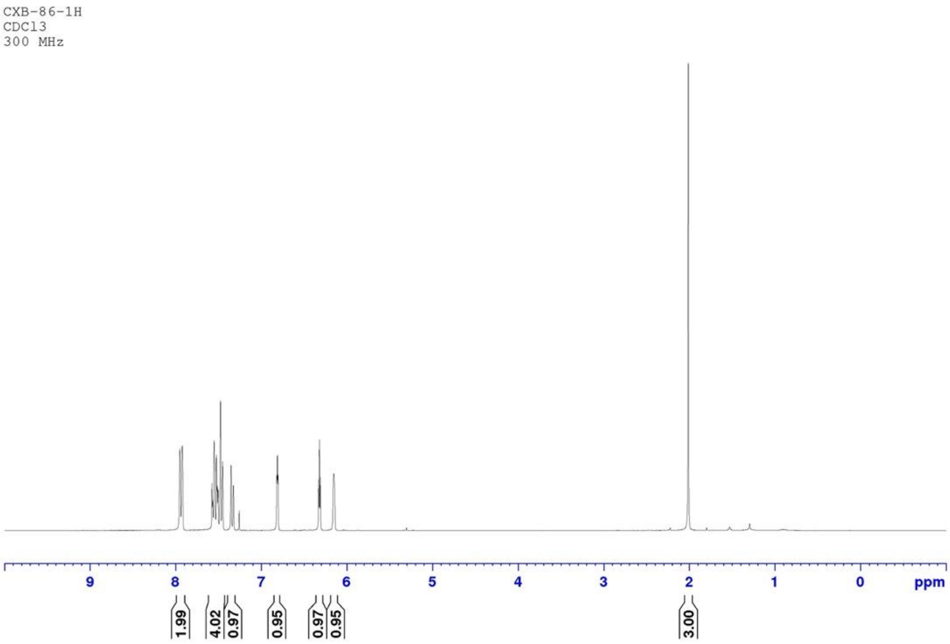

Figure S42. ^{1}H NMR ($CDCl_3$) of compound **1m**.

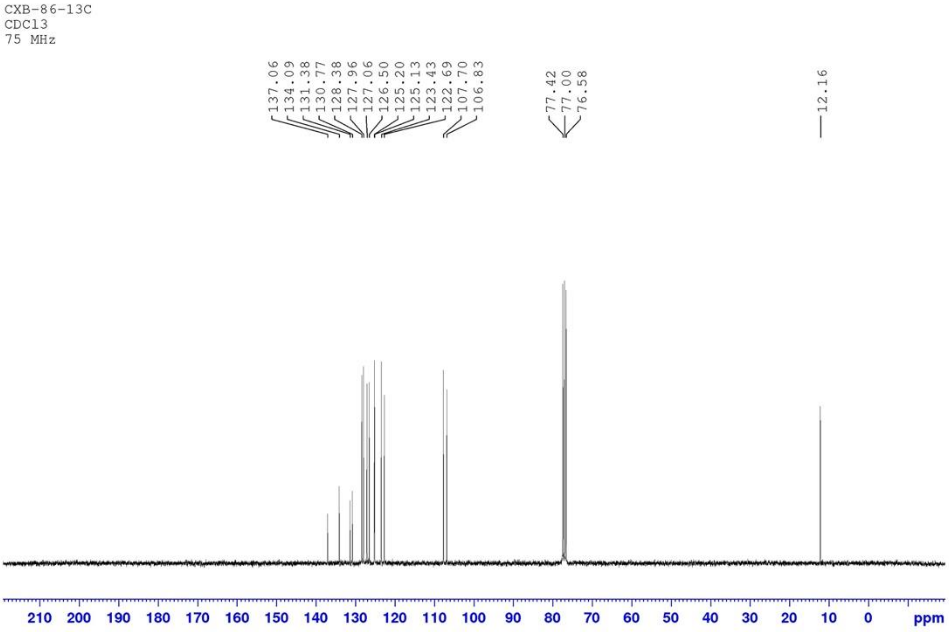

Figure S43. ^{13}C NMR ($CDCl_3$) of compound **1m**.

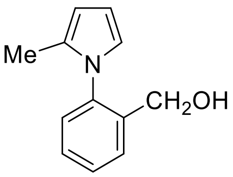

1n

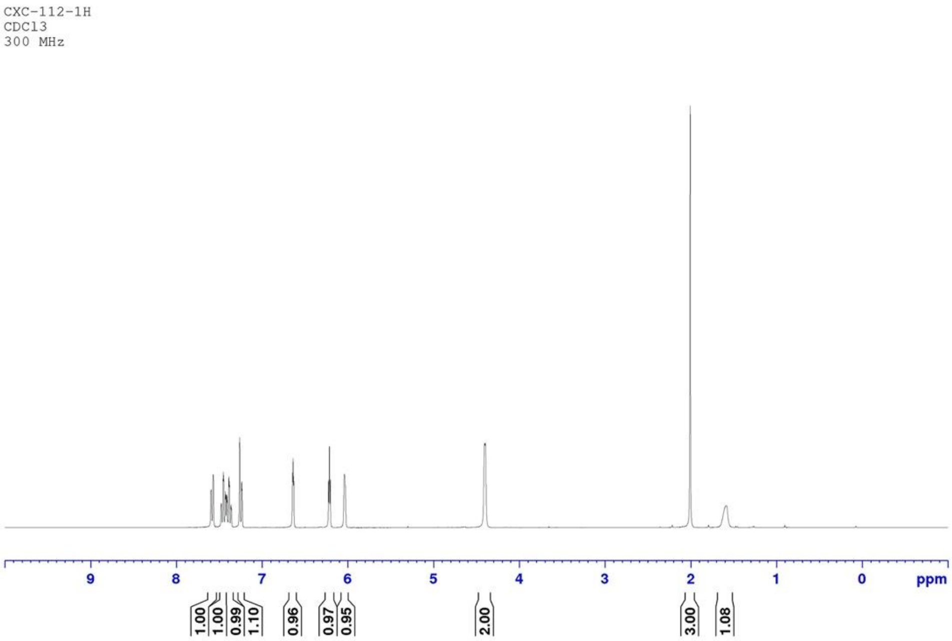

Figure S44. ^{1}H NMR ($CDCl_3$) of compound **1n**.

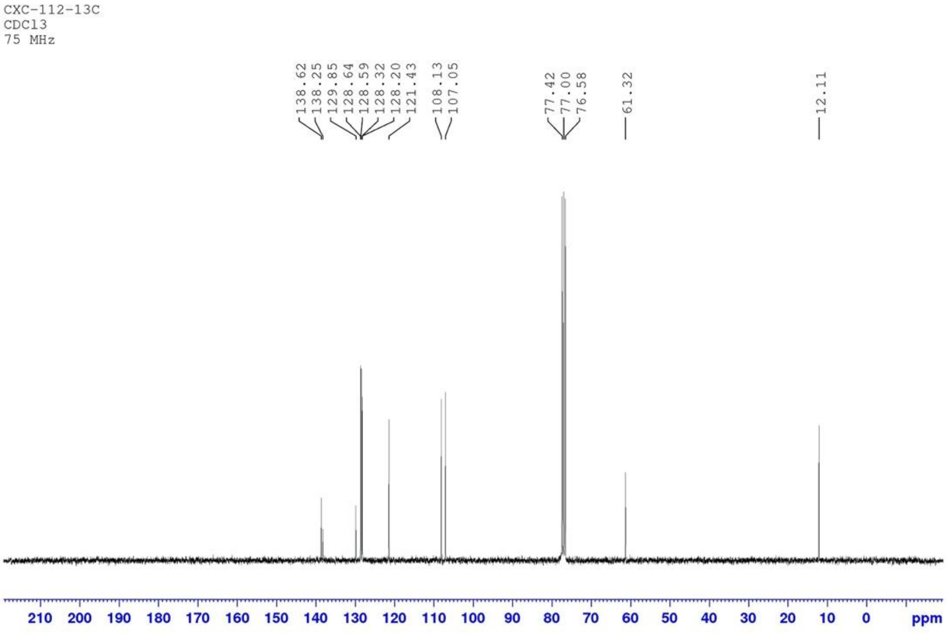

Figure S45. ^{13}C NMR ($CDCl_3$) of compound **1n**.

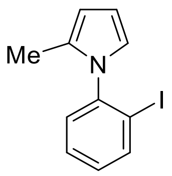

1o

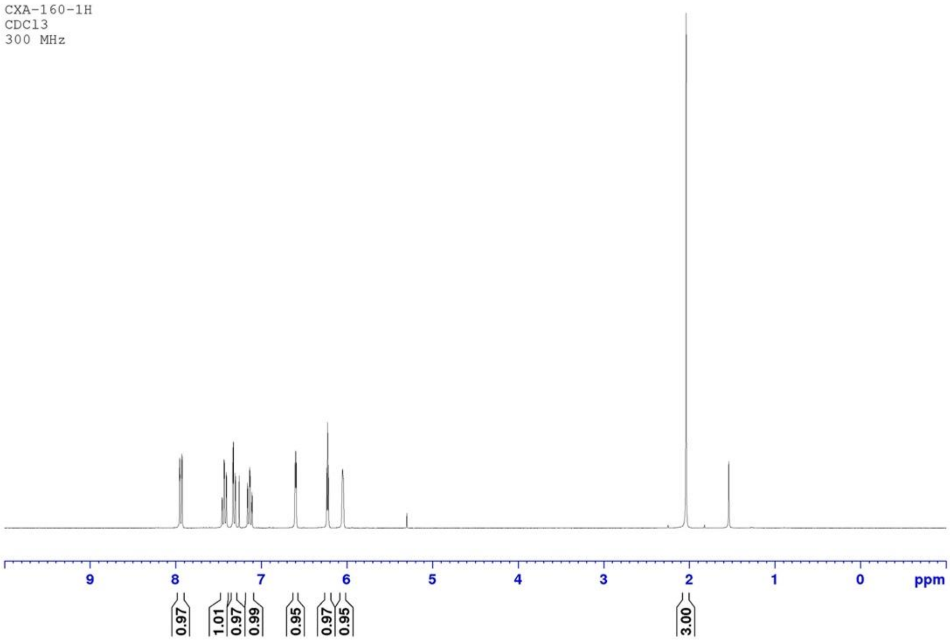

Figure S46. ^{1}H NMR ($CDCl_3$) of compound **1o**.

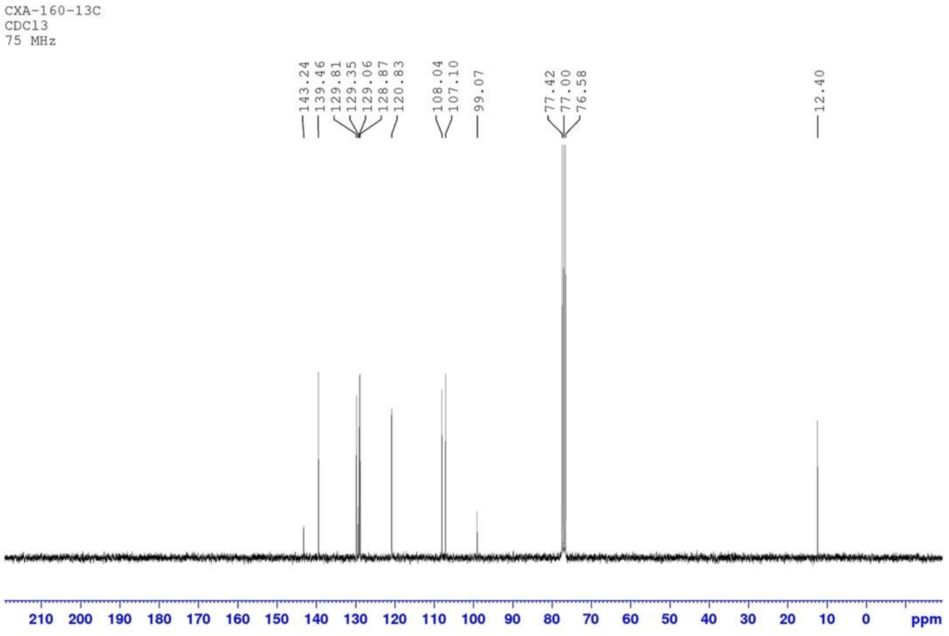

Figure S47. ^{13}C NMR ($CDCl_3$) of compound **1o**.

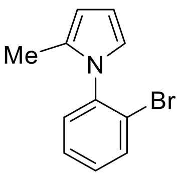

1p

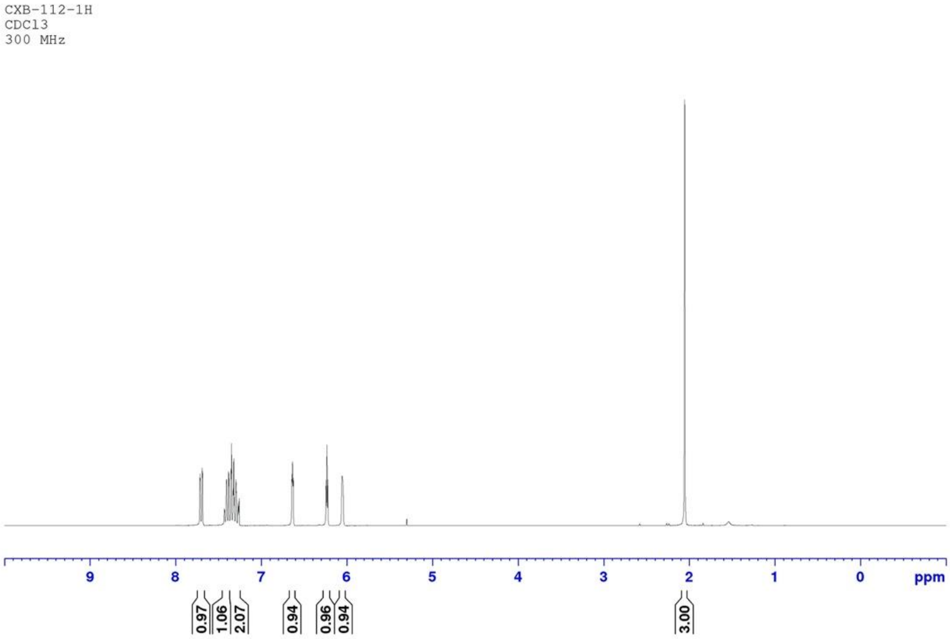

Figure S48. ^{1}H NMR ($CDCl_3$) of compound **1p**.

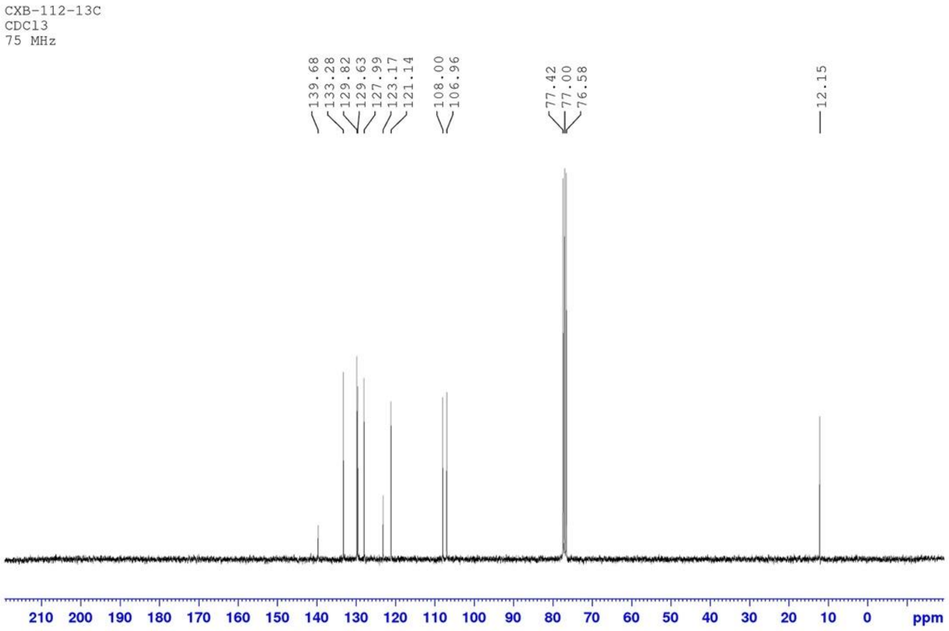

Figure S49. ^{13}C NMR ($CDCl_3$) of compound **1p**.

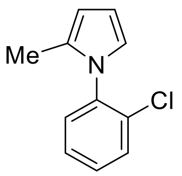

1q

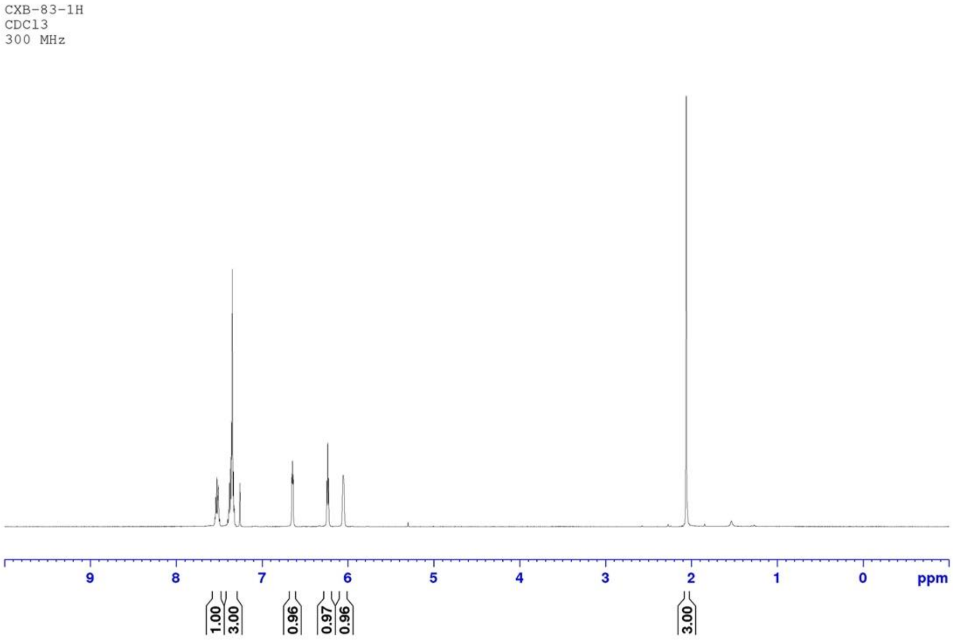

Figure S50. ^{1}H NMR ($CDCl_3$) of compound **1q**.

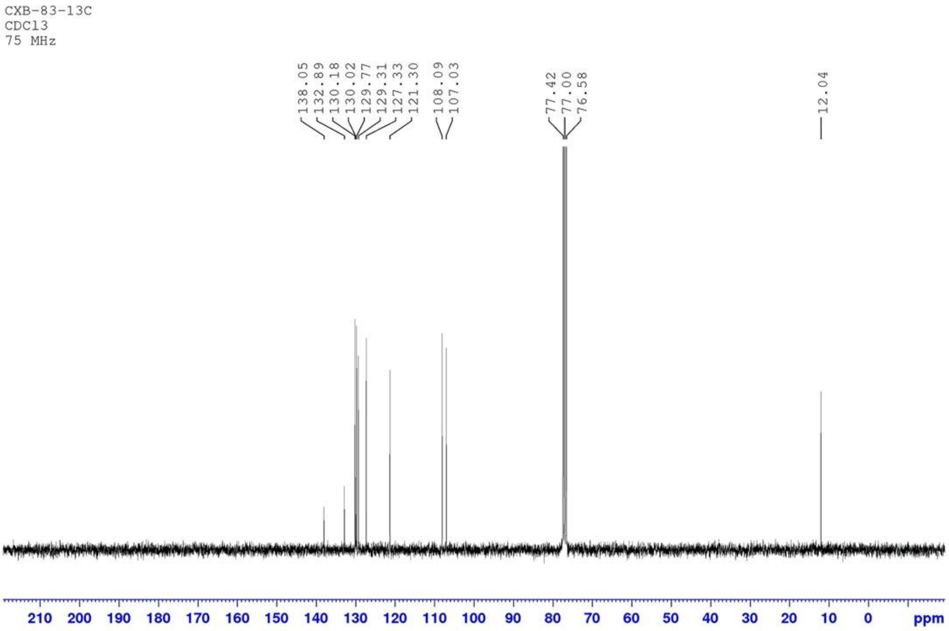

Figure S51. ^{13}C NMR ($CDCl_3$) of compound **1q**.

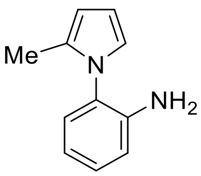

1r

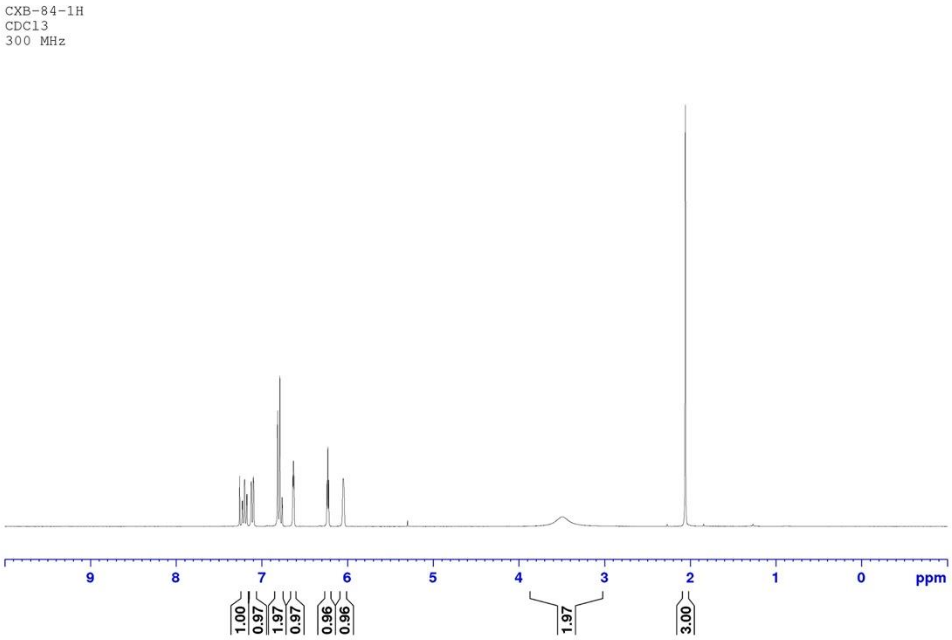

Figure S52. ^{1}H NMR ($CDCl_3$) of compound **1r**.

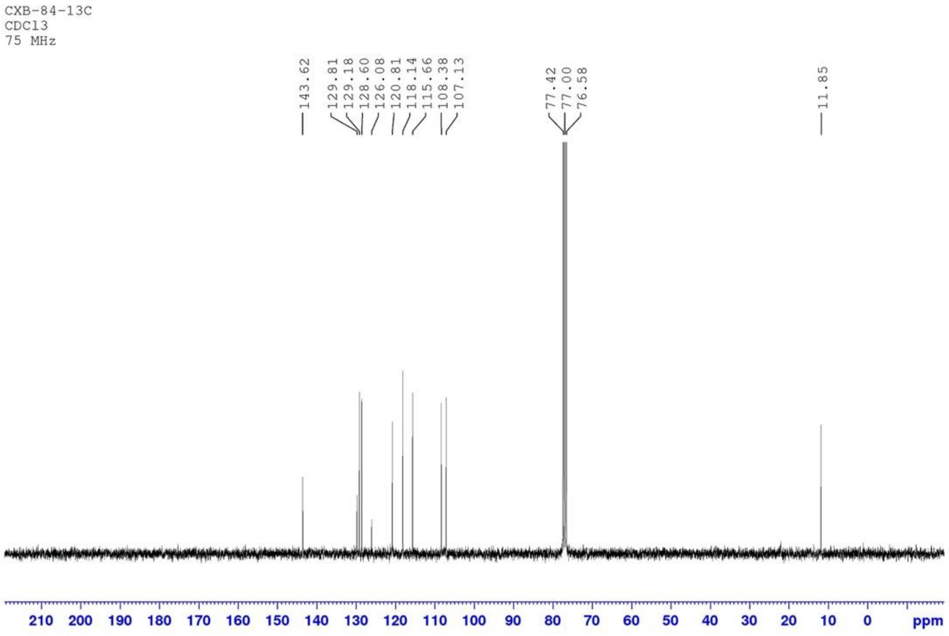

Figure S53. ^{13}C NMR ($CDCl_3$) of compound **1r**.

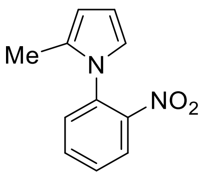

1s

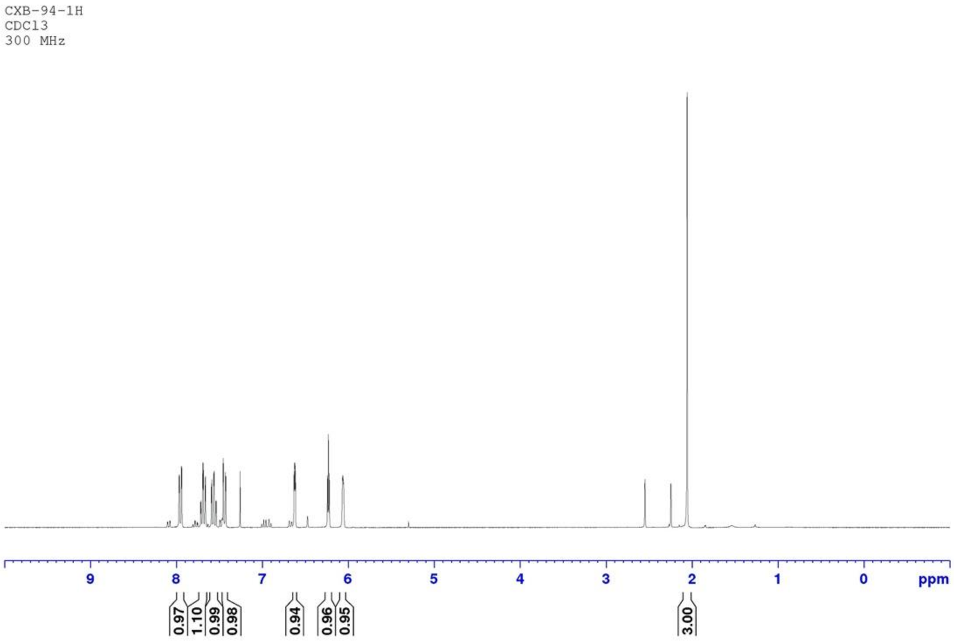

Figure S54. ^{1}H NMR ($CDCl_3$) of compound **1s**.

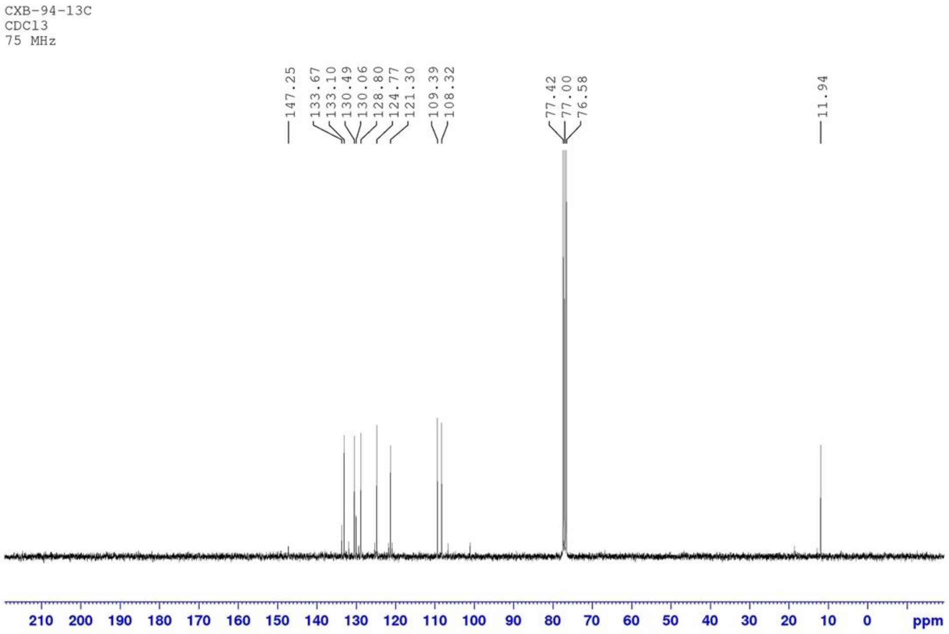

Figure S55. ^{13}C NMR ($CDCl_3$) of compound **1s**.

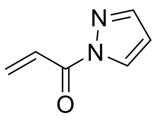

2a

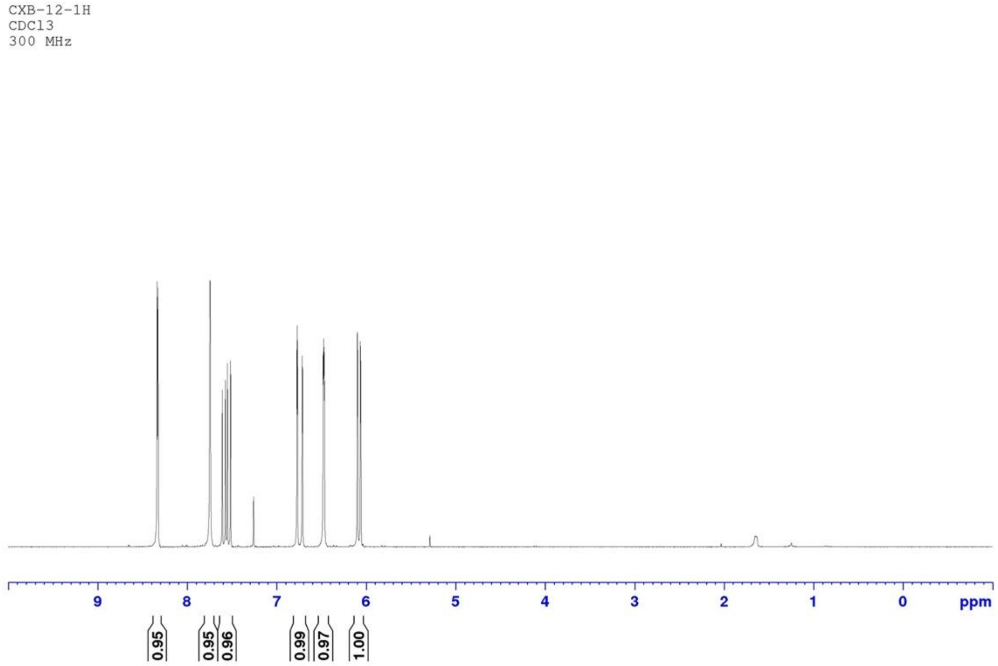

Figure S56. ^{1}H NMR ($CDCl_3$) of compound **2a**.

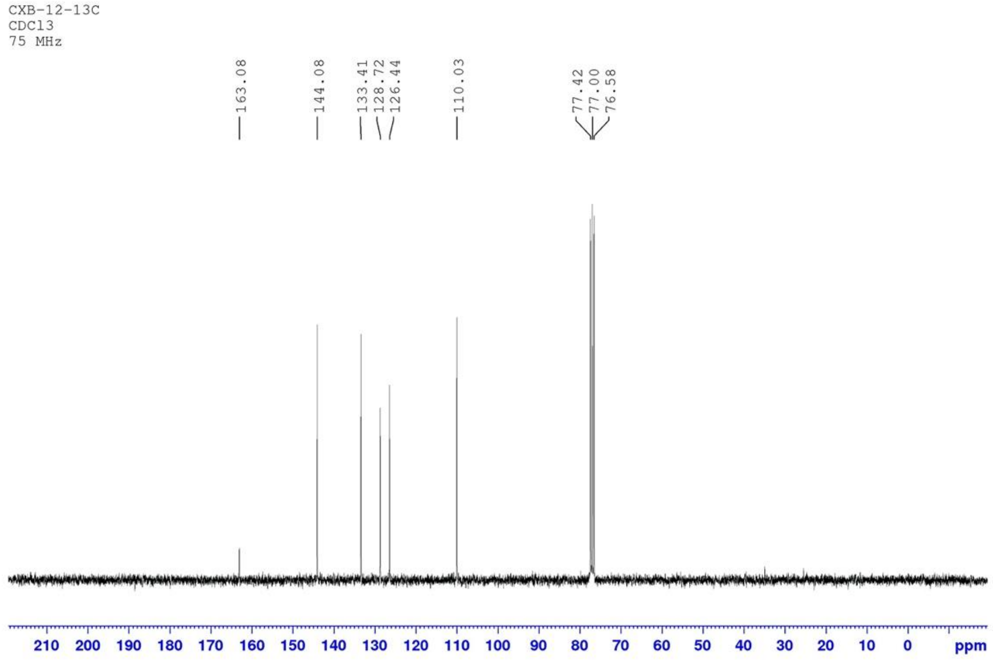

Figure S57. ^{13}C NMR ($CDCl_3$) of compound **2a**.

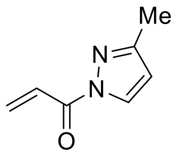

2b

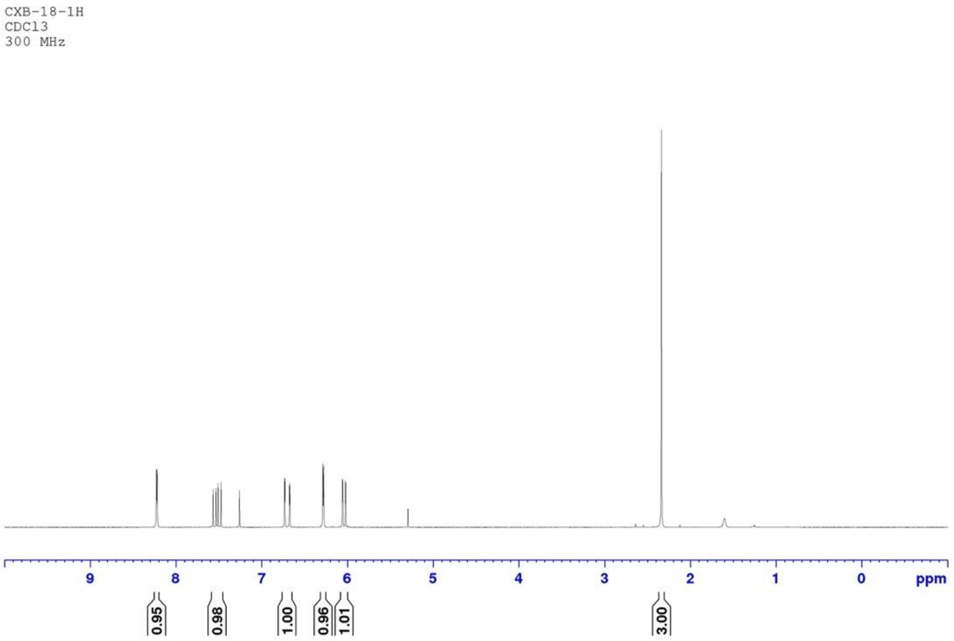

Figure S58. ^{1}H NMR ($CDCl_3$) of compound **2b**.

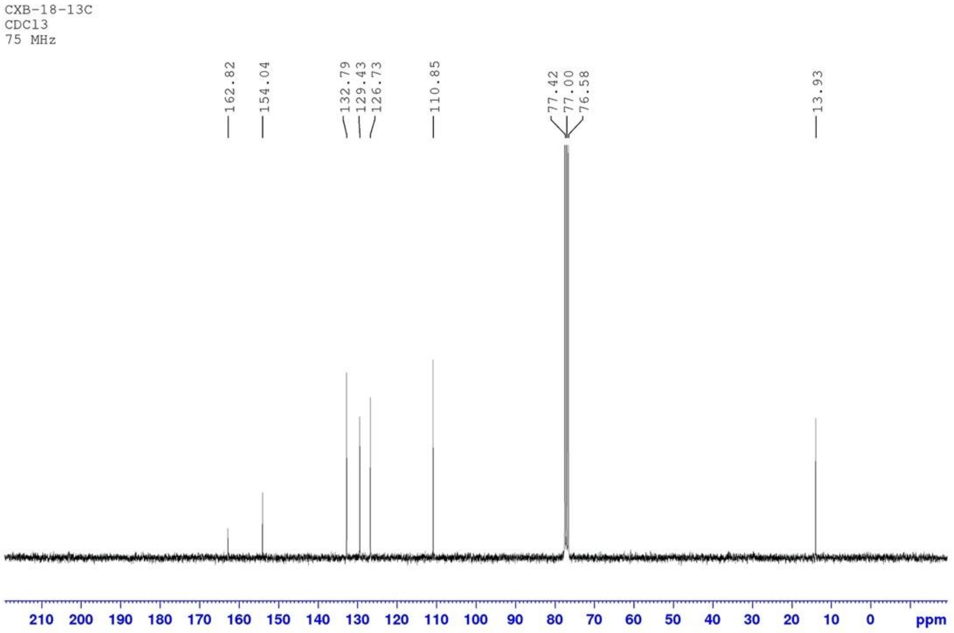

Figure S59. ^{13}C NMR ($CDCl_3$) of compound **2b**.

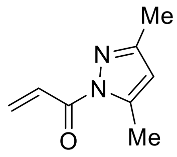

2c

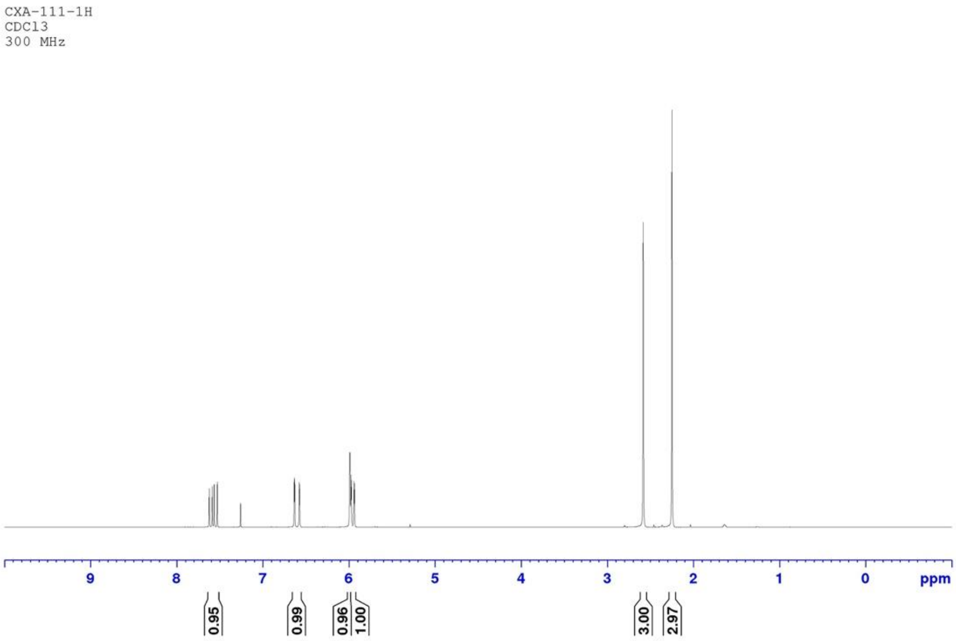

Figure S60. ^{1}H NMR ($CDCl_3$) of compound **2c**.

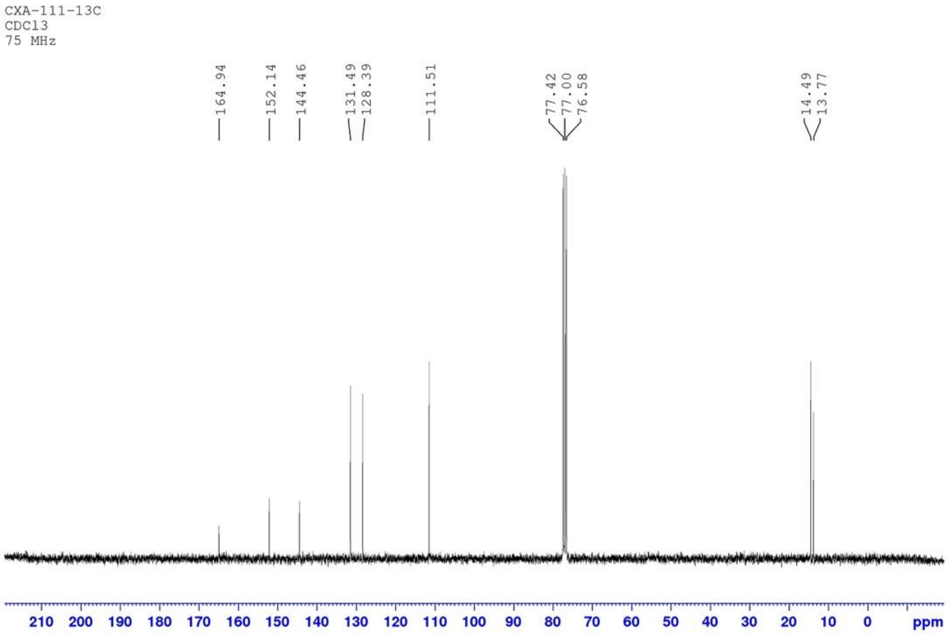

Figure S61. ^{13}C NMR ($CDCl_3$) of compound **2c**.

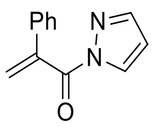

2d

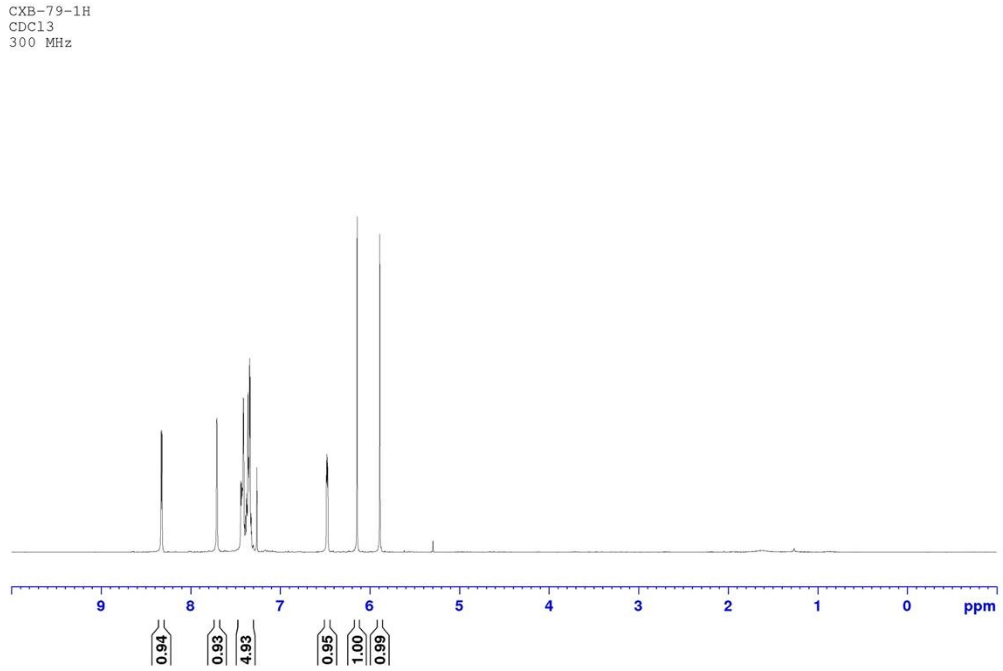

Figure S62. ^{1}H NMR ($CDCl_3$) of compound **2d**.

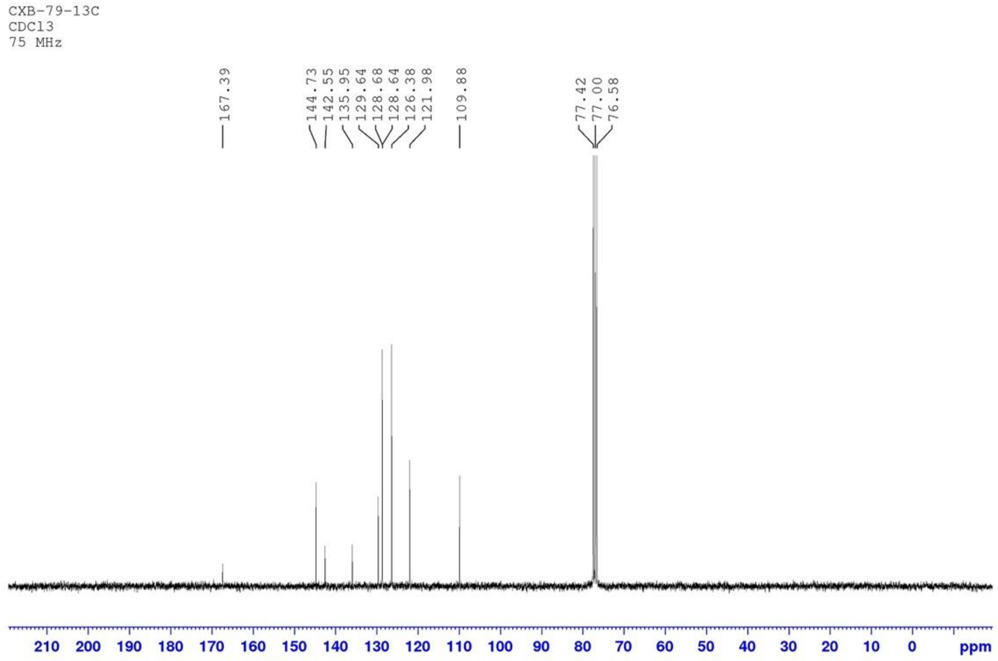

Figure S63. ^{13}C NMR ($CDCl_3$) of compound **2d**.

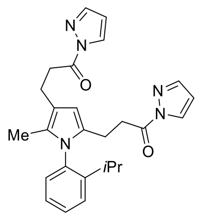

3aa’

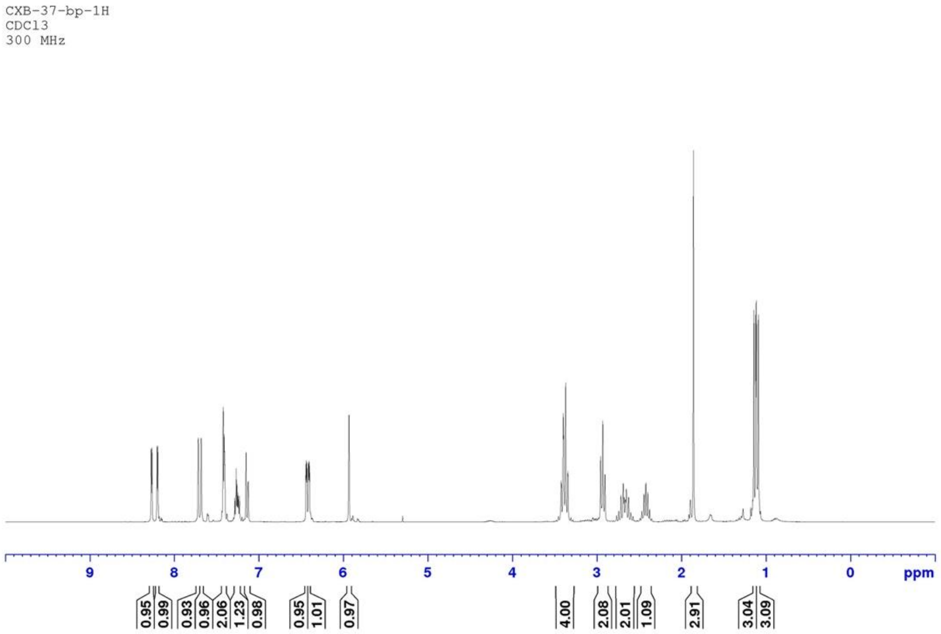

Figure S64. ^{1}H NMR ($CDCl_3$) of compound **3aa’**.

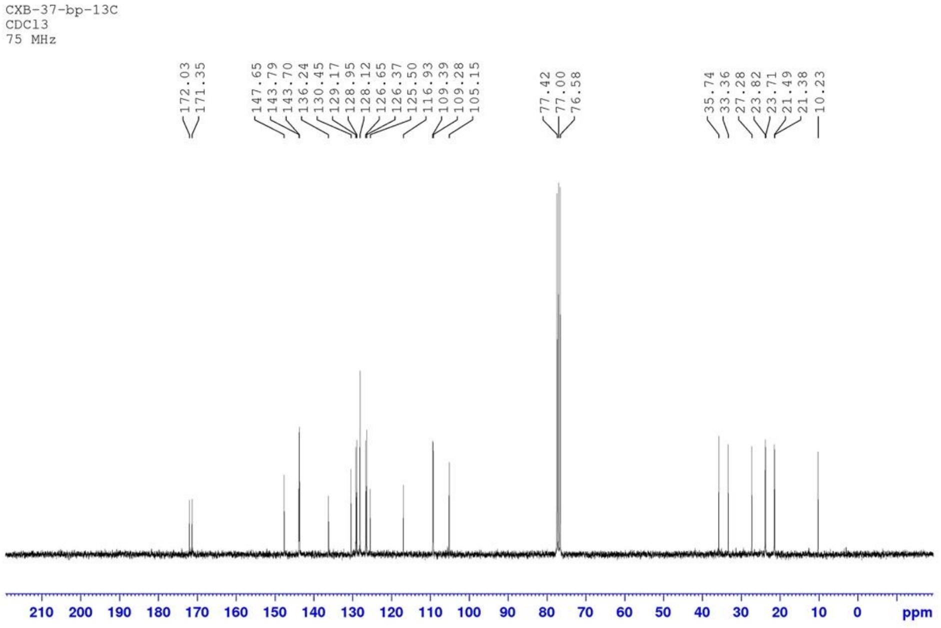

Figure S65. ^{13}C NMR ($CDCl_3$) of compound **3aa’**.

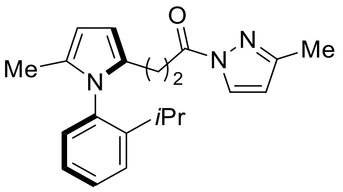

3ab

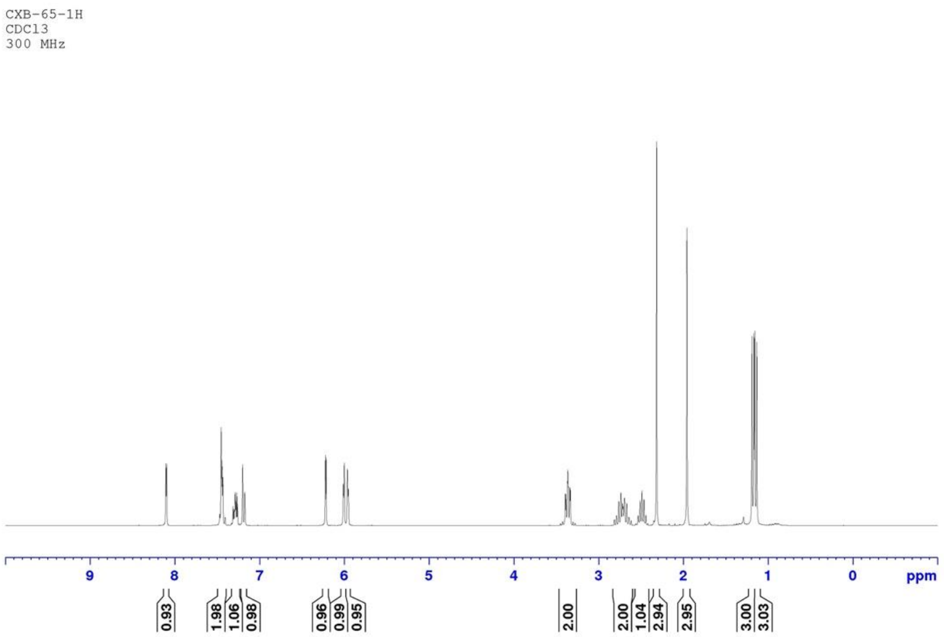

Figure S66. ^{1}H NMR ($CDCl_3$) of compound **3ab**.

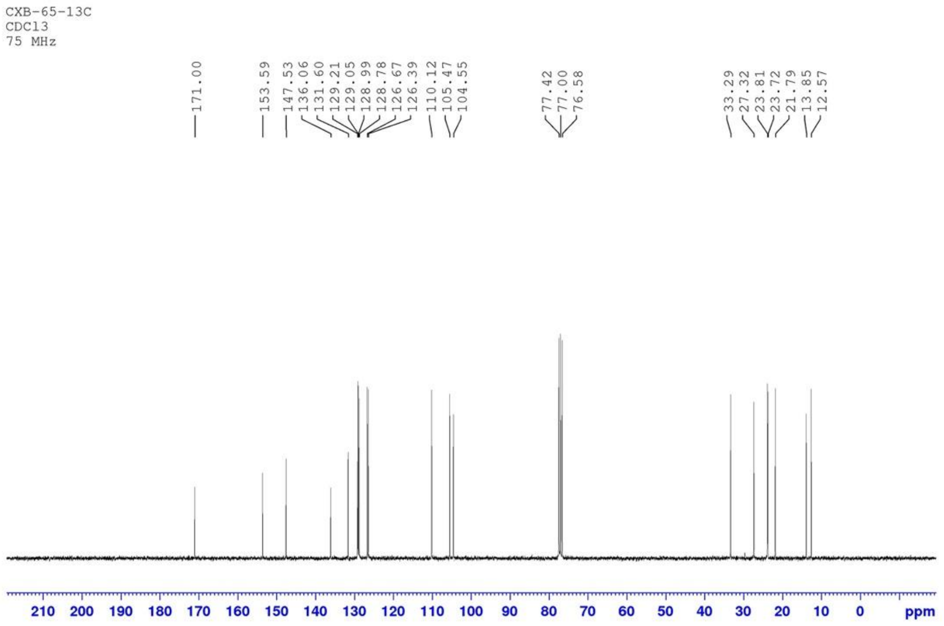

Figure S67. ^{13}C NMR ($CDCl_3$) of compound **3ab**.

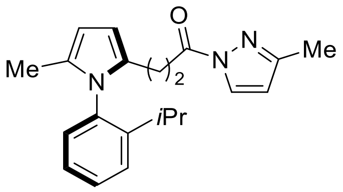

3ab

[CHIRALCEL OD-H, 25 °C, *i*PrOH/hexane = 2/98 (v/v), 1.0 mL/min, 254 nm]

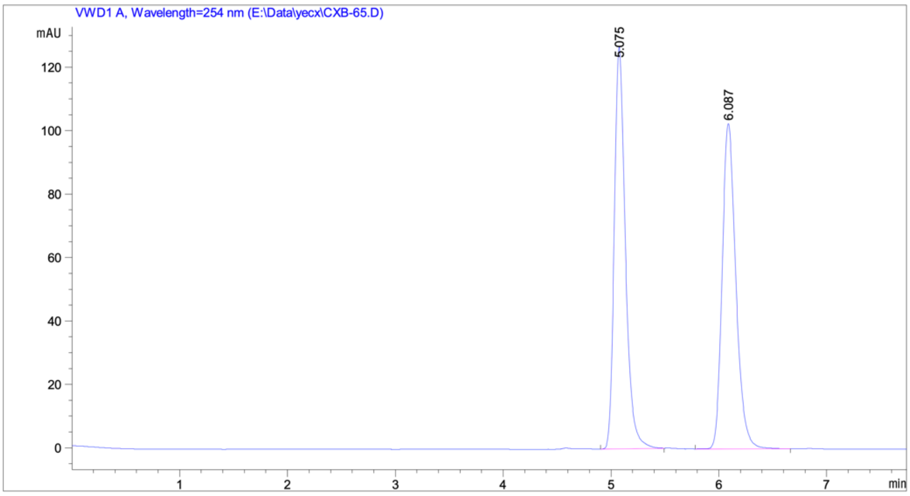

Peak #	RetTime [min]	Type	Width [min]	Area [mAU*s]	Height [mAU]	Area %
1	5.075	BB	0.1085	906.80054	126.74860	50.0057
2	6.087	BB	0.1351	906.59491	102.57146	49.9943

Figure S68. Racemate of compound **3ab**.

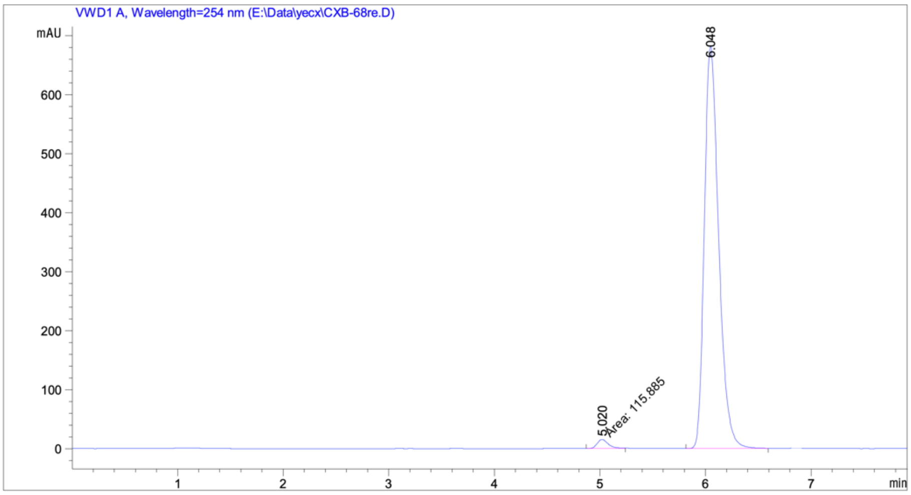

Peak #	RetTime [min]	Type	Width [min]	Area [mAU*s]	Height [mAU]	Area %
1	5.020	MF	0.1254	115.88484	15.40024	1.7706
2	6.048	BB	0.1451	6428.90186	681.20020	98.2294

Figure S69. Enantioenriched mixture of compound **3ab**.

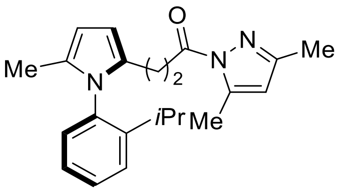

3ac

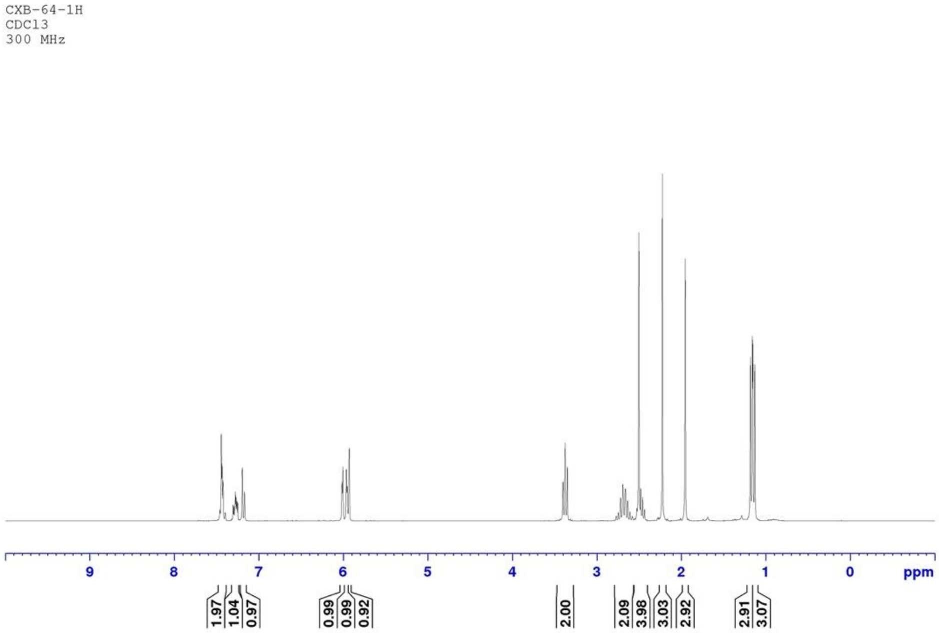

Figure S70. ^{1}H NMR ($CDCl_3$) of compound **3ac**.

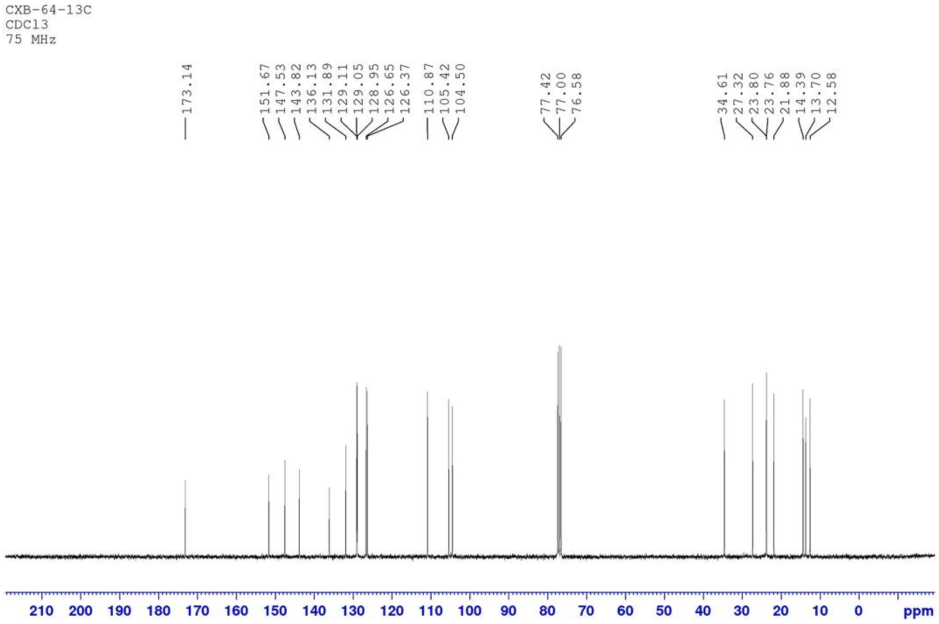

Figure S71. ^{13}C NMR ($CDCl_3$) of compound **3ac**.

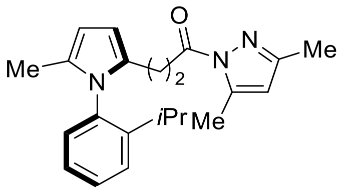

3ac

[CHIRALCEL OD-H, 25 °C, *i*PrOH/hexane = 1/99 (v/v), 1.0 mL/min, 254 nm]

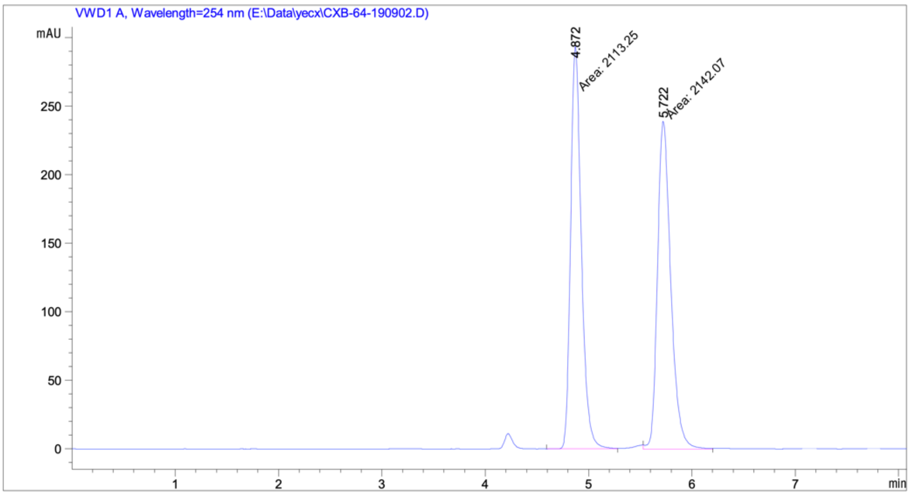

Peak #	RetTime [min]	Type	Width [min]	Area [mAU*s]	Height [mAU]	Area %
1	4.872	MF	0.1199	2113.25220	293.65958	49.6614
2	5.722	FM	0.1492	2142.06689	239.22525	50.3386

Figure S72. Racemate of compound **3ac**.

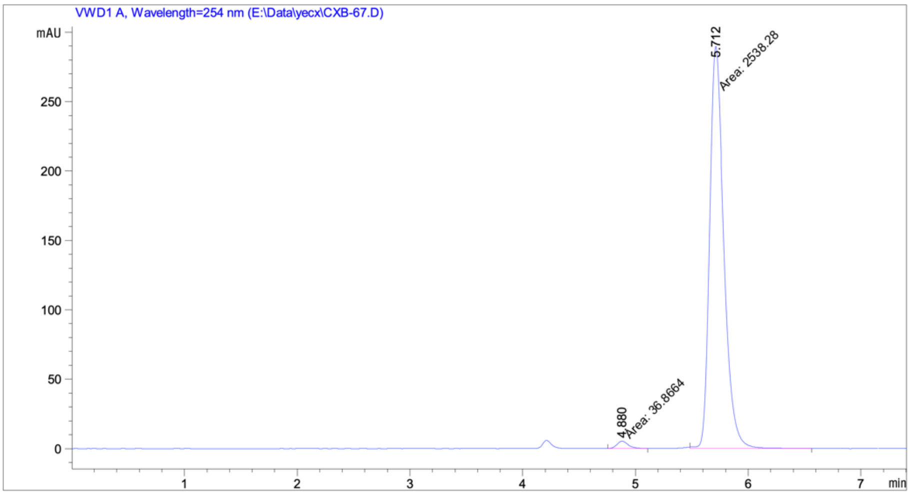

Peak #	RetTime [min]	Type	Width [min]	Area [mAU*s]	Height [mAU]	Area %
1	4.880	FM	0.1158	36.86640	5.30811	1.4316
2	5.712	FM	0.1460	2538.28198	289.74600	98.5684

Figure S73. Enantioenriched mixture of compound **3ac**.

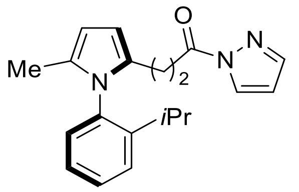

3aa

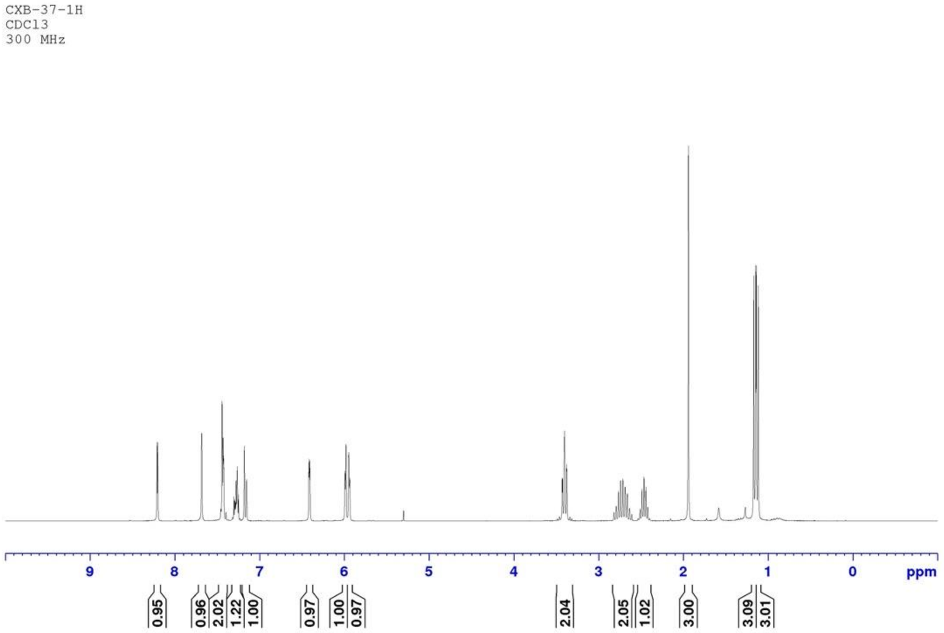

Figure S74. ^{1}H NMR ($CDCl_3$) of compound **3aa**.

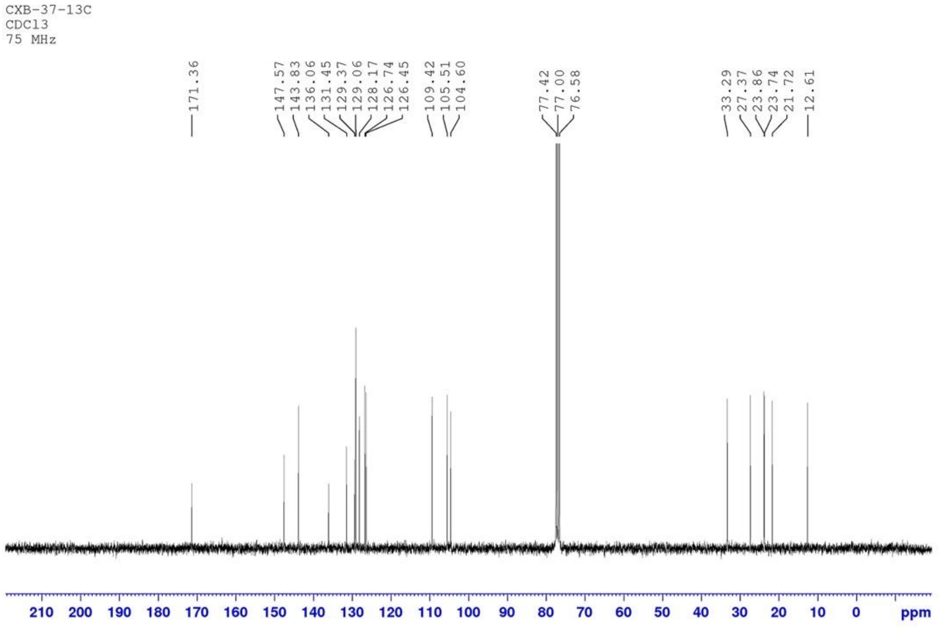

Figure S75. ^{13}C NMR ($CDCl_3$) of compound **3aa**.

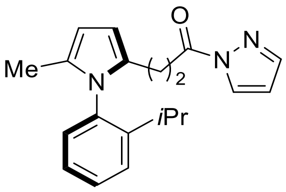

3aa

[CHIRALCEL OD-H, 25 °C, *i*PrOH/hexane = 2/98 (v/v), 1.0 mL/min, 254 nm]

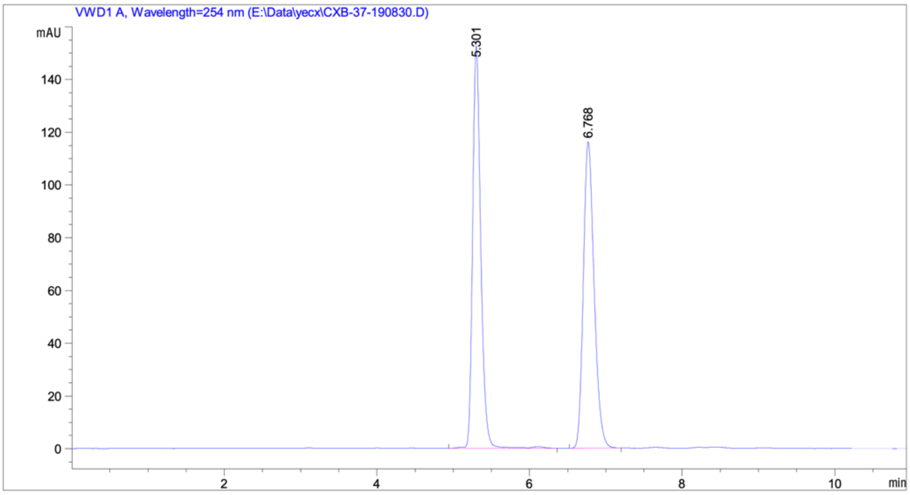

Peak #	RetTime [min]	Type	Width [min]	Area [mAU*s]	Height [mAU]	Area %
1	5.301	VV R	0.1130	1136.04651	152.41487	49.9310
2	6.768	BB	0.1503	1139.18811	116.26014	50.0690

Figure S76. Racemate of compound **3aa**.

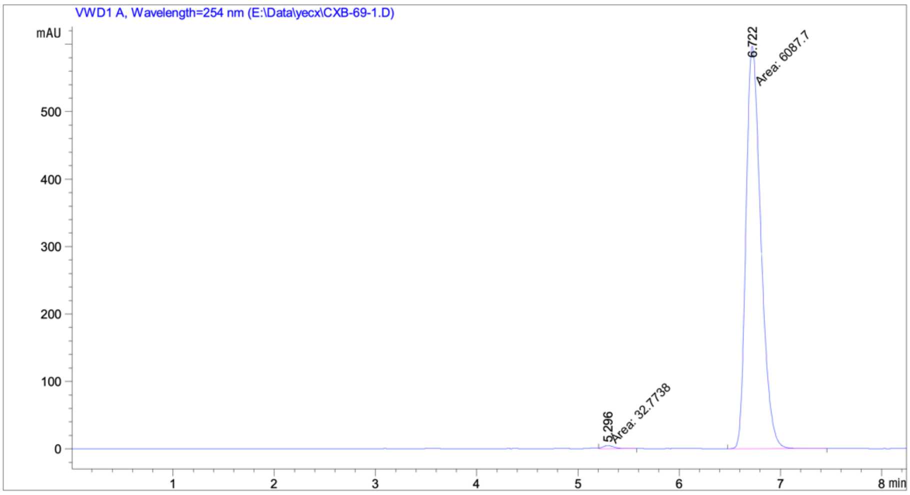

Peak #	RetTime [min]	Type	Width [min]	Area [mAU*s]	Height [mAU]	Area %
1	5.296	FM	0.1217	32.77381	4.48863	0.5355
2	6.722	MM	0.1701	6087.70020	596.37640	99.4645

Figure S77. Enantioenriched mixture of compound **3aa**.

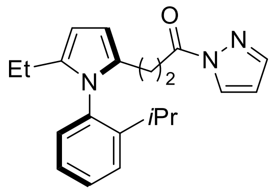

3b

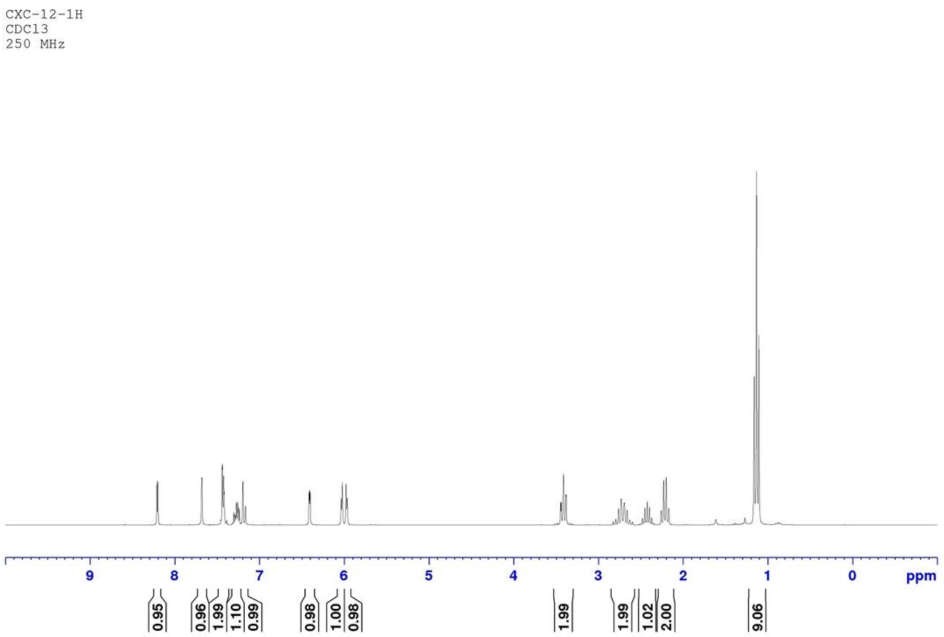

Figure S78. ^{1}H NMR ($CDCl_3$) of compound **3b**.

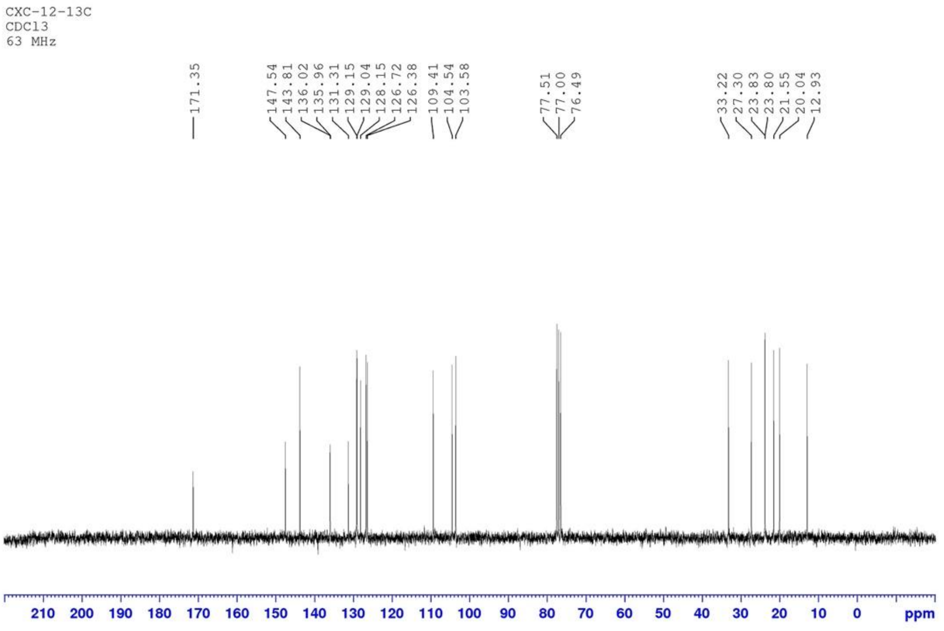

Figure S79. ^{13}C NMR ($CDCl_3$) of compound **3b**.

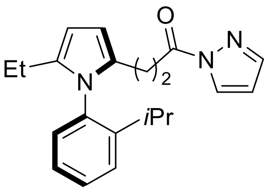

3b

[CHIRALCEL OD-H, 25 °C, *i*PrOH/hexane = 2/98 (v/v), 1.0 mL/min, 254 nm]

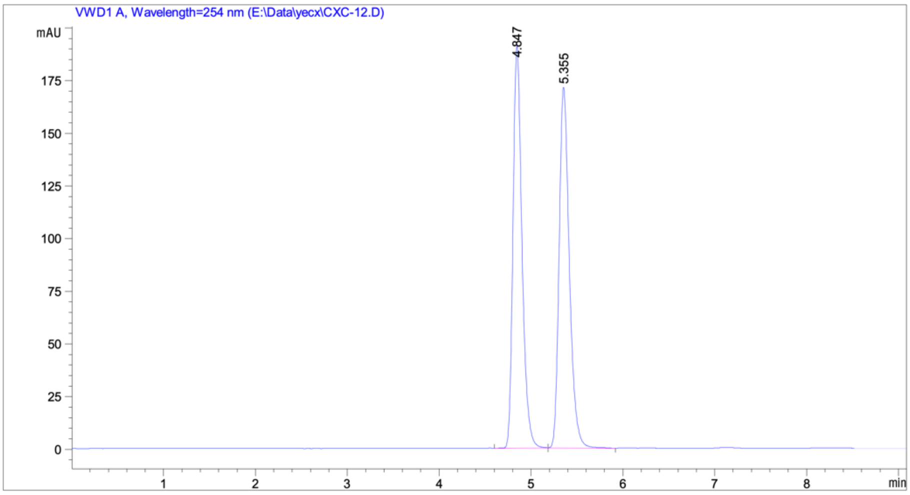

Peak #	RetTime [min]	Type	Width [min]	Area [mAU*s]	Height [mAU]	Area %
1	4.847	BB	0.1032	1294.57019	190.74664	49.5658
2	5.355	BB	0.1178	1317.25281	171.19235	50.4342

Figure S80. Racemate of compound **3b**.

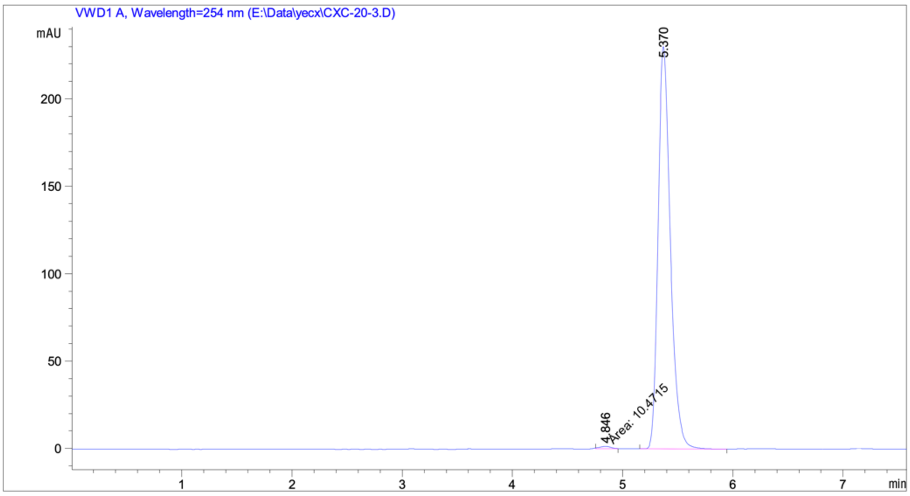

Peak #	RetTime [min]	Type	Width [min]	Area [mAU*s]	Height [mAU]	Area %
1	4.846	MF	0.1215	10.47149	1.43678	0.5836
2	5.370	BV R	0.1184	1783.73279	230.08961	99.4164

Figure S81. Enantioenriched mixture of compound **3b**.

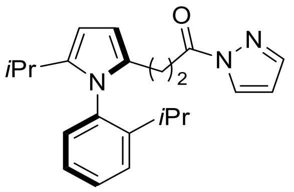

3c

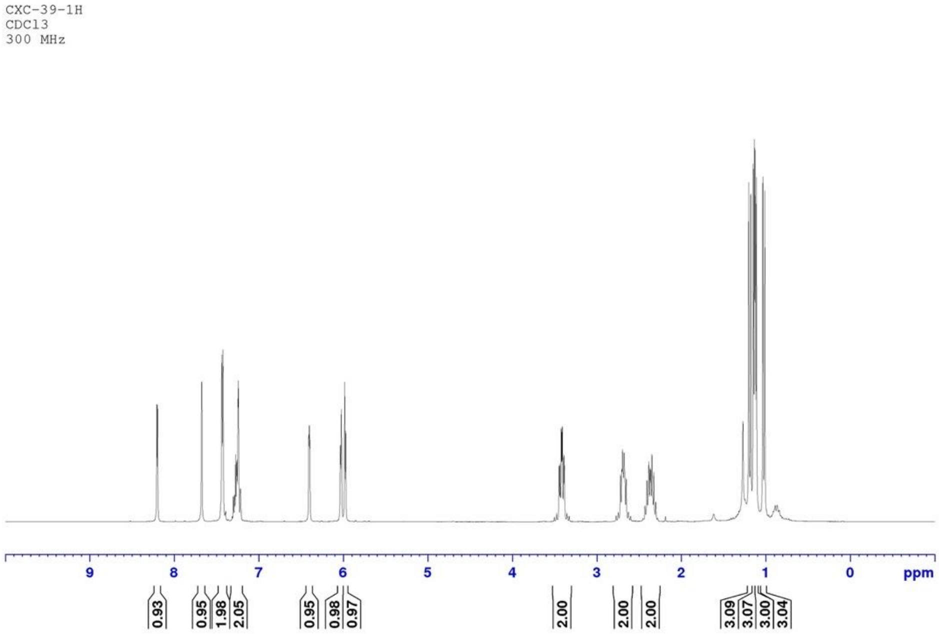

Figure S82. ^{1}H NMR ($CDCl_3$) of compound **3c**.

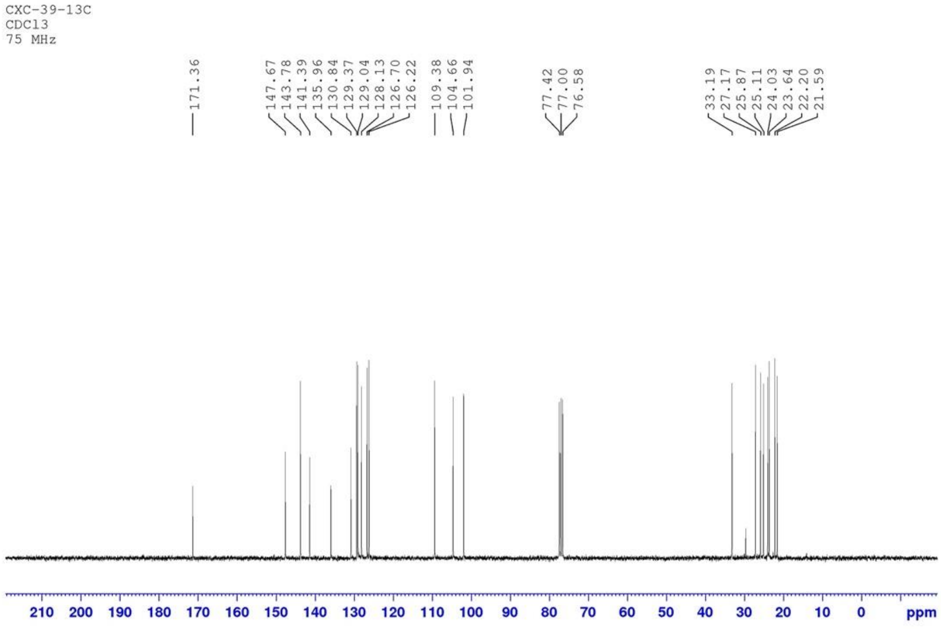

Figure S83. ^{13}C NMR ($CDCl_3$) of compound **3c**.

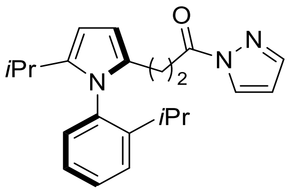

3c

[CHIRALCEL OD-H, 25 °C, *i*PrOH/hexane = 1/99 (v/v), 1.0 mL/min, 254 nm]

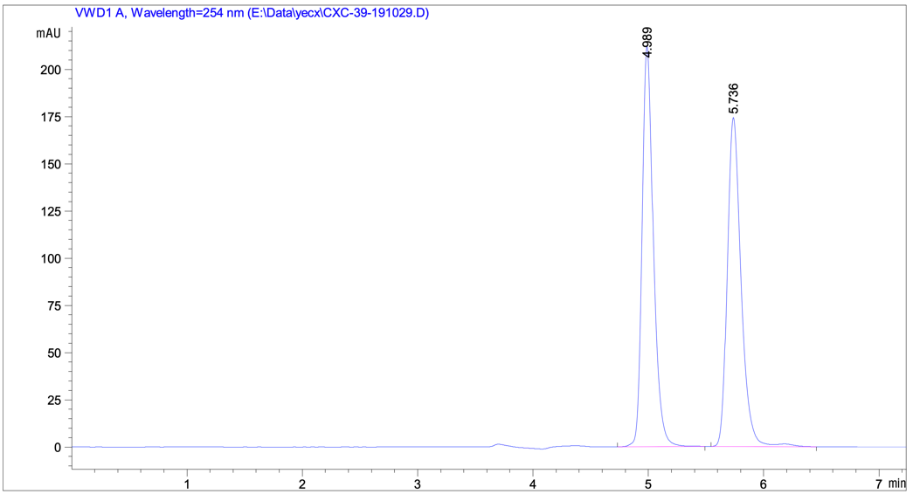

Peak #	RetTime [min]	Type	Width [min]	Area [mAU*s]	Height [mAU]	Area %
1	4.989	BB	0.1030	1434.46118	211.78912	49.7567
2	5.736	BV R	0.1270	1448.48914	174.16809	50.2433

Figure S84. Racemate of compound **3c**.

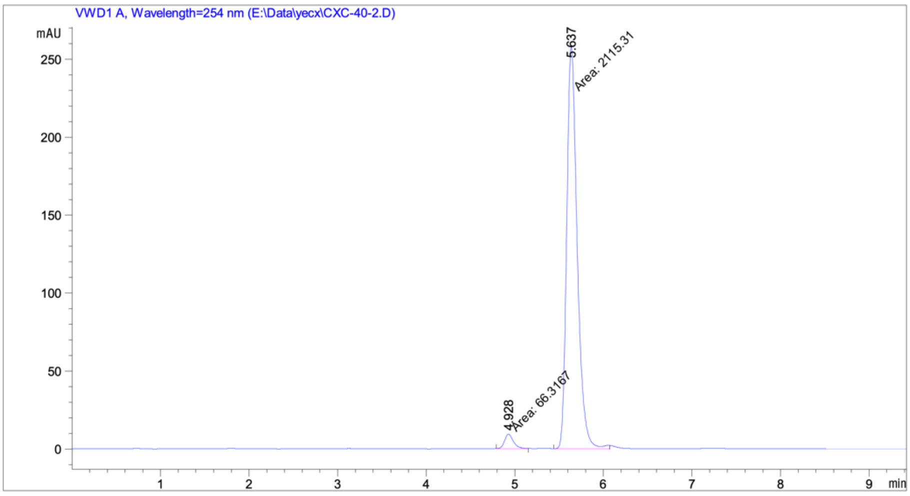

Peak #	RetTime [min]	Type	Width [min]	Area [mAU*s]	Height [mAU]	Area %
1	4.928	MF	0.1175	66.31670	9.40925	3.0398
2	5.637	MF	0.1369	2115.30908	257.61020	96.9602

Figure S85. Enantioenriched mixture of compound **3c**.

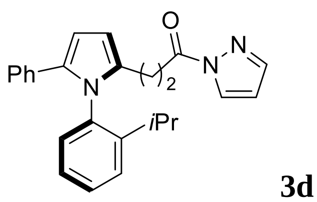

3d

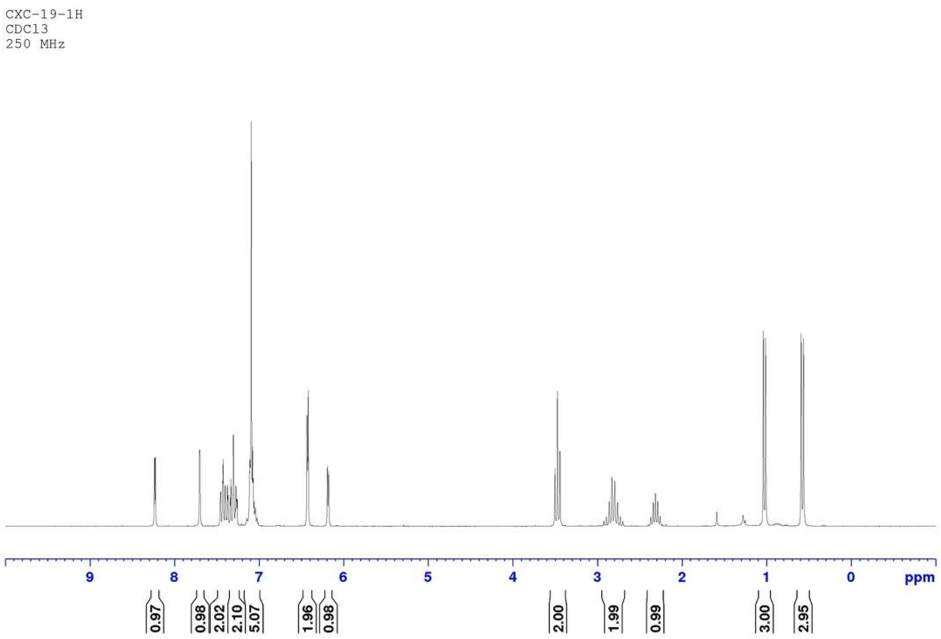

Figure S86. ^{1}H NMR ($CDCl_3$) of compound **3d**.

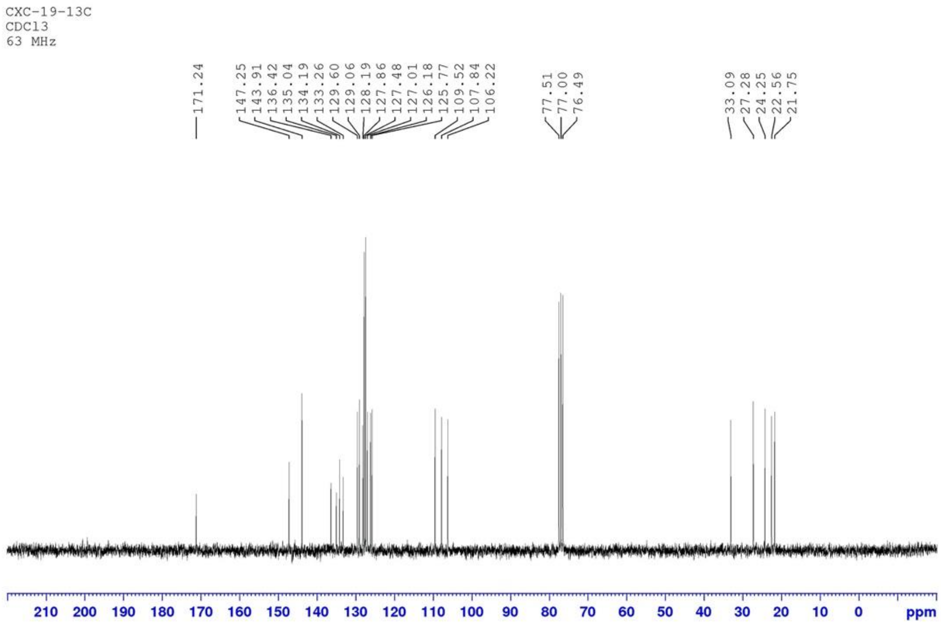

Figure S87. ^{13}C NMR ($CDCl_3$) of compound **3d**.

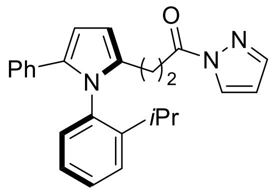

3d

[CHIRALCEL OD-H, 25 °C, *i*PrOH/hexane = 1/99 (v/v), 1.0 mL/min, 254 nm]

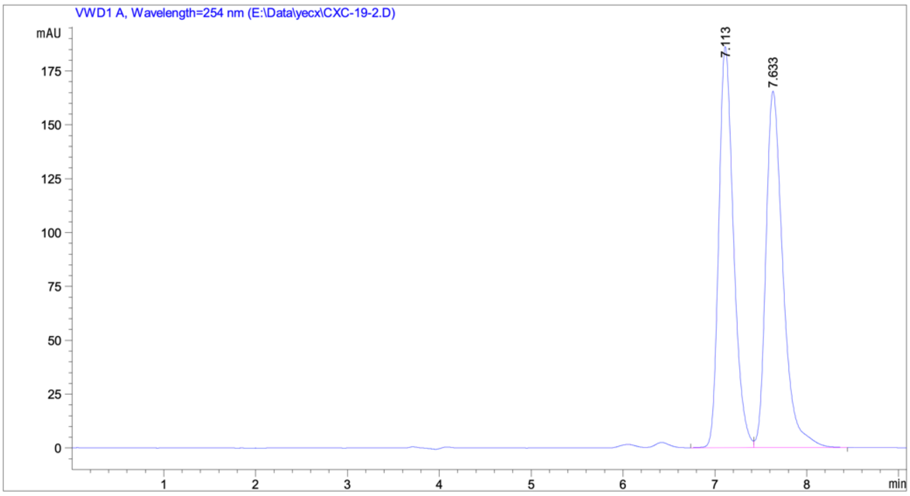

Peak #	RetTime [min]	Type	Width [min]	Area [mAU*s]	Height [mAU]	Area %
1	7.113	BV	0.1702	2077.97754	186.15077	49.2753
2	7.633	VB	0.1968	2139.09595	165.45715	50.7247

Figure S88. Racemate of compound **3d**.

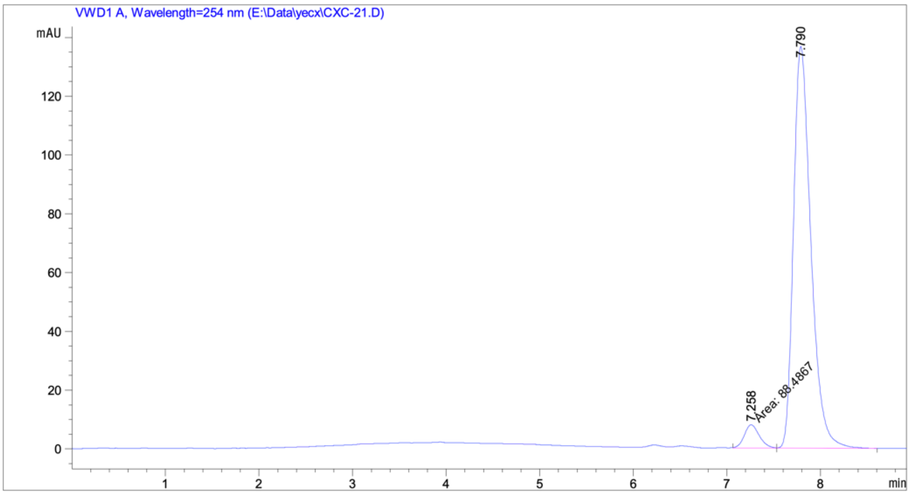

Peak #	RetTime [min]	Type	Width [min]	Area [mAU*s]	Height [mAU]	Area %
1	7.258	FM	0.1858	88.48666	7.93632	4.7697
2	7.790	VB	0.1978	1766.71008	136.66870	95.2303

Figure S89. Enantioenriched mixture of compound **3d**.

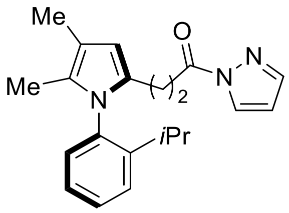

3e

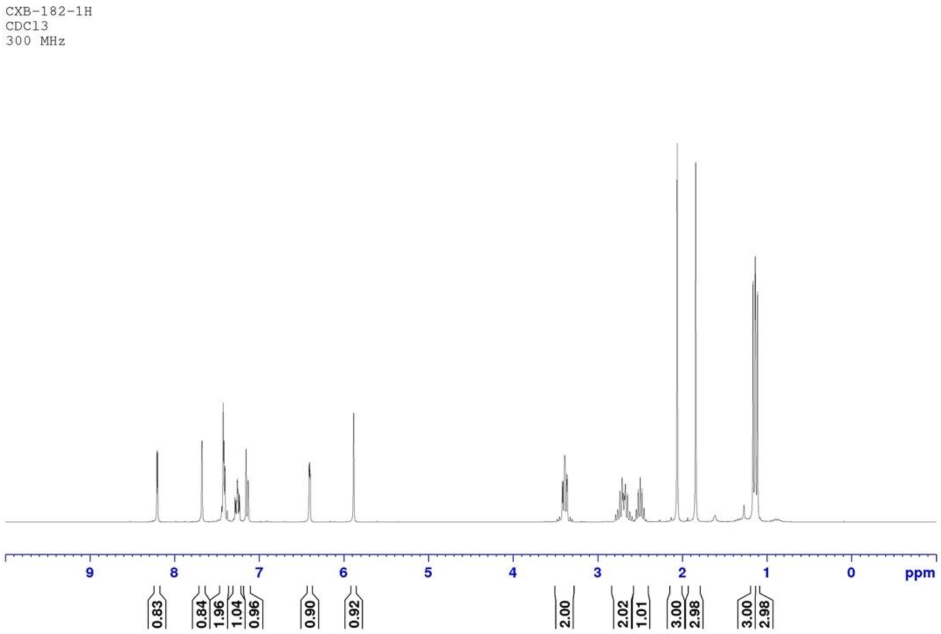

Figure S90. ^{1}H NMR ($CDCl_3$) of compound **3e**.

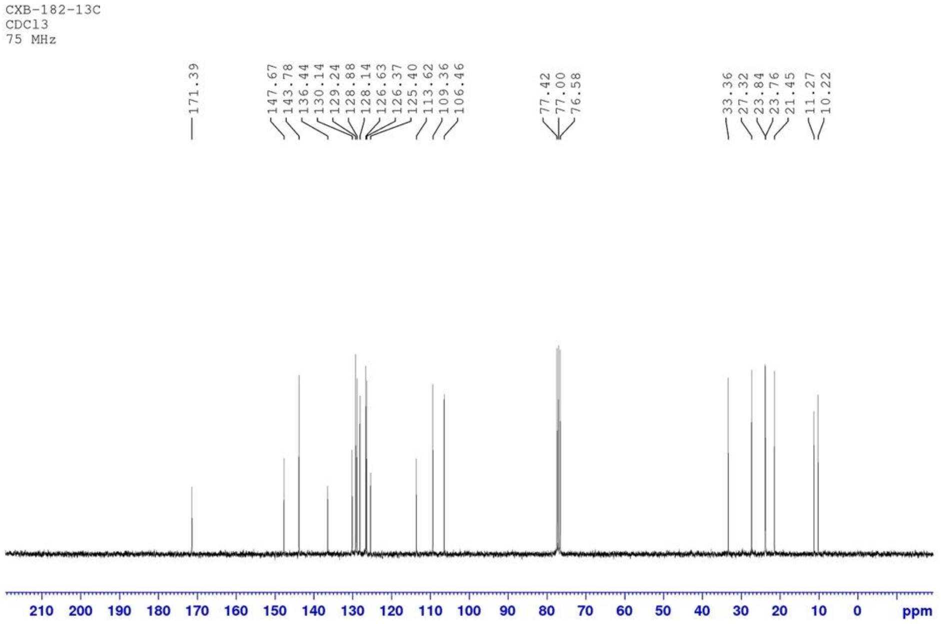

Figure S91. ^{13}C NMR ($CDCl_3$) of compound **3e**.

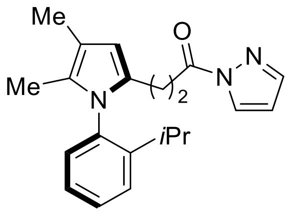

3e

[CHIRALCEL OD-H, 25 °C, *i*PrOH/hexane = 2/98 (v/v), 1.0 mL/min, 254 nm]

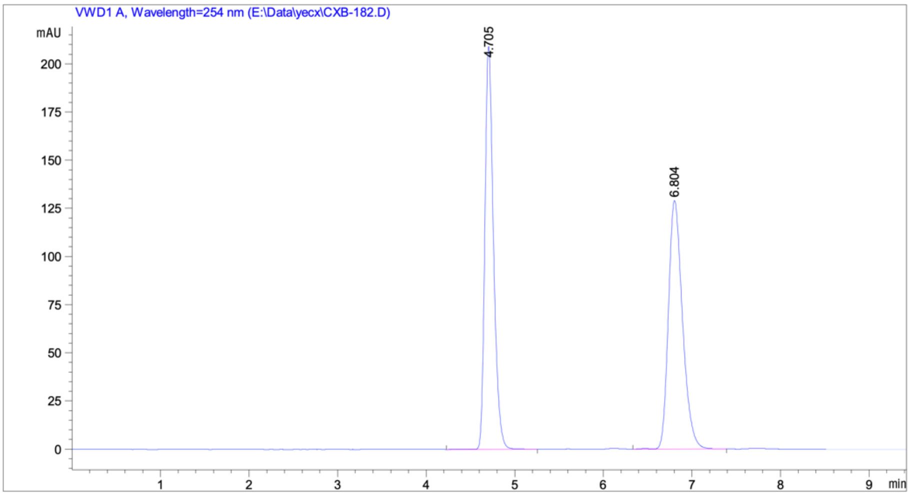

Peak #	RetTime [min]	Type	Width [min]	Area [mAU*s]	Height [mAU]	Area %
1	4.705	VB R	0.1019	1415.18835	209.20575	49.7937
2	6.804	VB R	0.1700	1426.91431	129.07408	50.2063

Figure S92. Racemate of compound **3e**.

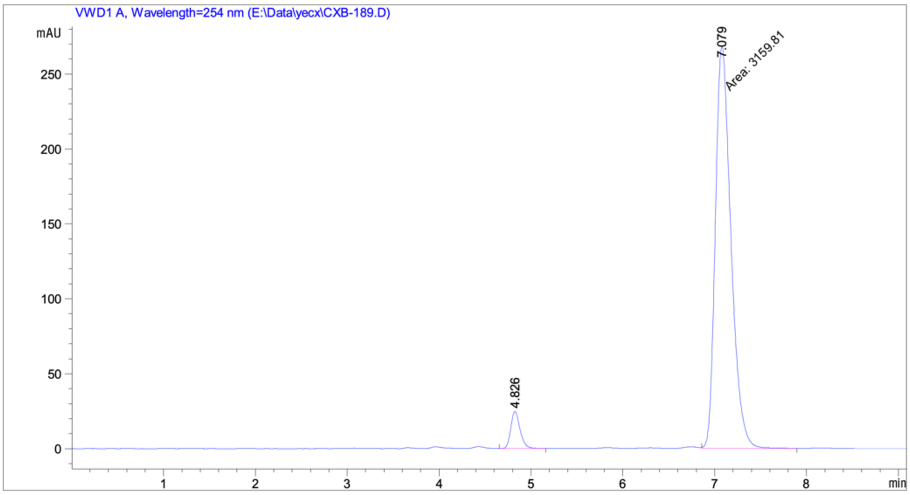

Peak #	RetTime [min]	Type	Width [min]	Area [mAU*s]	Height [mAU]	Area %
1	4.826	BB	0.1072	171.04887	24.56711	5.1353
2	7.079	FM	0.1963	3159.81055	268.25720	94.8647

Figure S93. Enantioenriched mixture of compound **3e**.

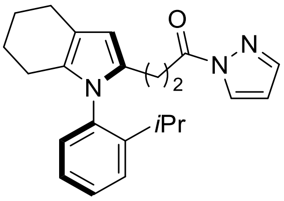

3f

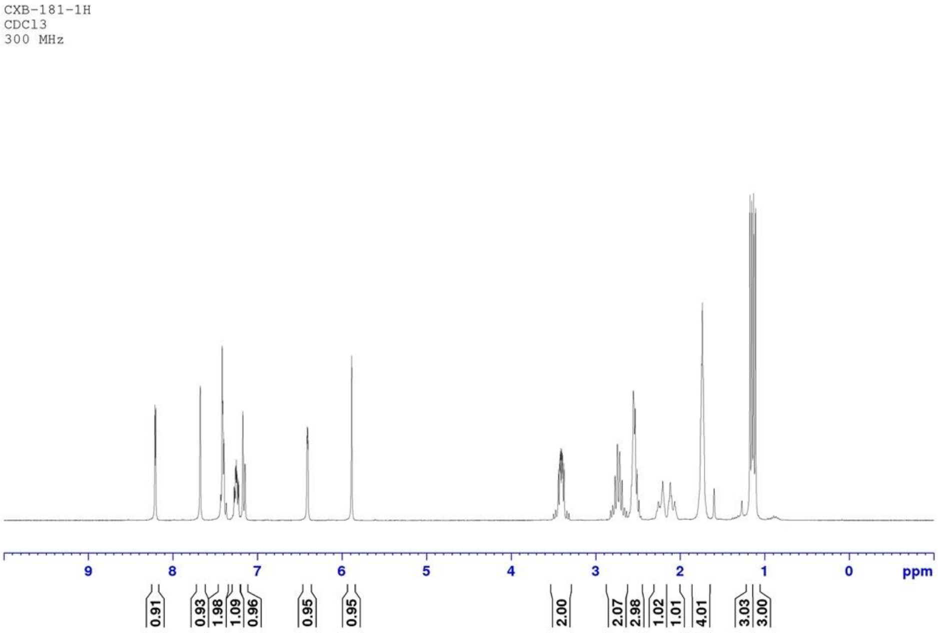

Figure S94. ^{1}H NMR ($CDCl_3$) of compound **3f**.

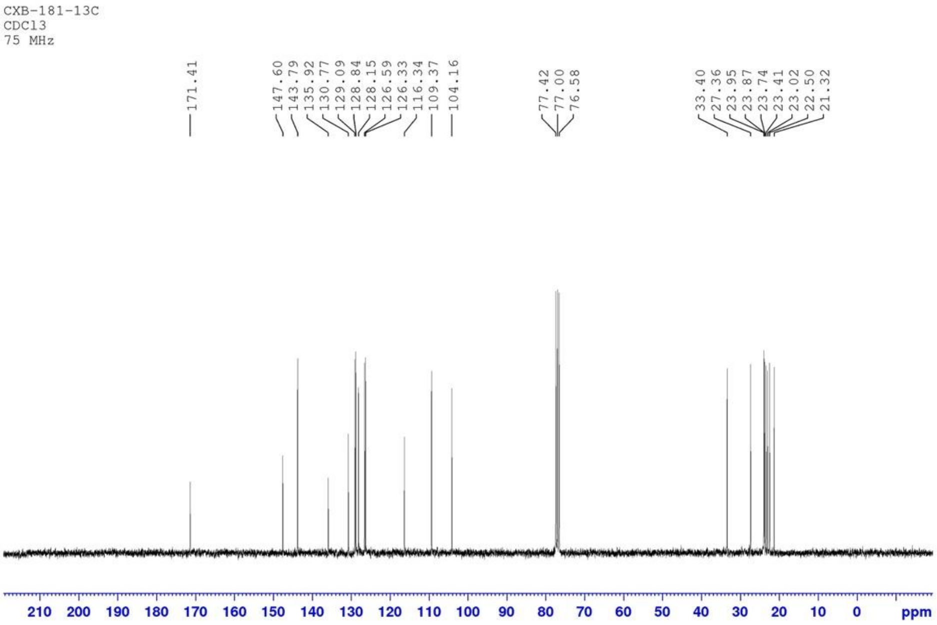

Figure S95. ^{13}C NMR ($CDCl_3$) of compound **3f**.

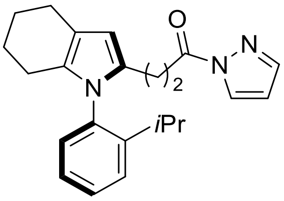

3f

[CHIRALCEL OD-H, 25 °C, *i*PrOH/hexane = 2/98 (v/v), 1.0 mL/min, 254 nm]

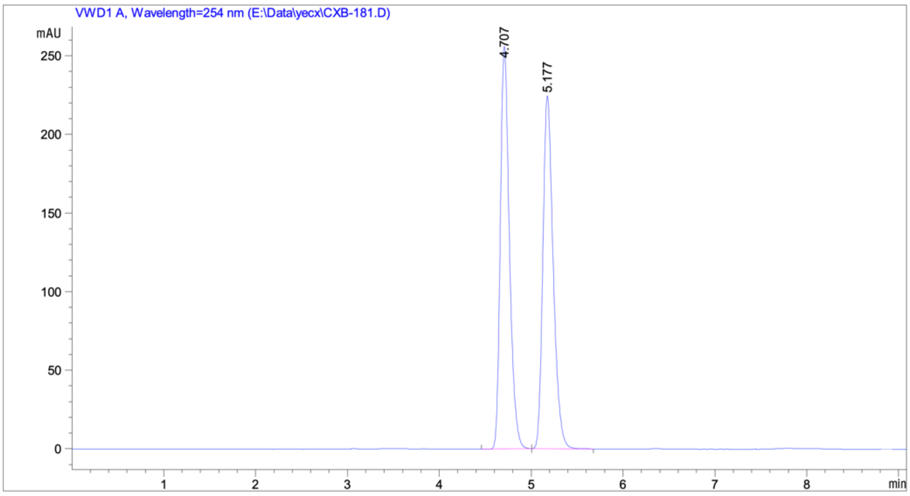

Peak #	RetTime [min]	Type	Width [min]	Area [mAU*s]	Height [mAU]	Area %
1	4.707	BB	0.1039	1752.19421	255.86079	49.9211
2	5.177	BB	0.1194	1757.73376	224.42897	50.0789

Figure S96. Racemate of compound **3f**.

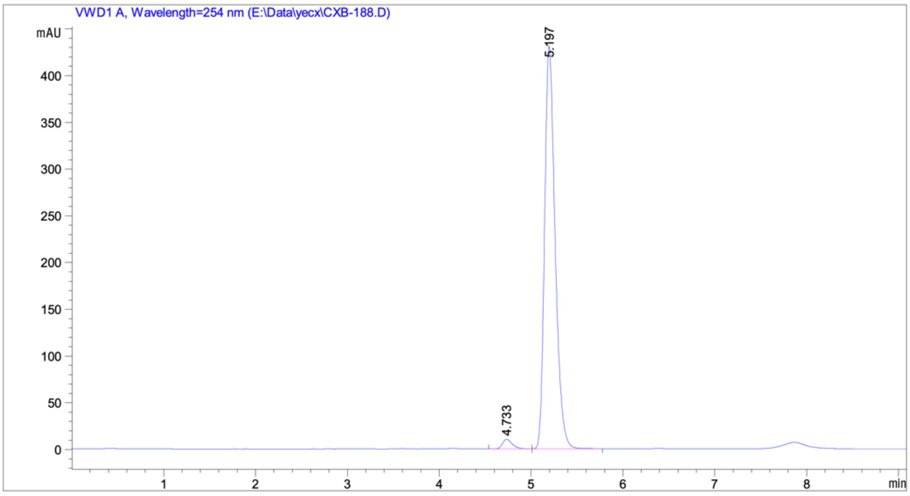

Peak #	RetTime [min]	Type	Width [min]	Area [mAU*s]	Height [mAU]	Area %
1	4.733	BV	0.1104	75.83353	10.35921	2.1627
2	5.197	VB	0.1209	3430.61865	430.64307	97.8373

Figure S97. Enantioenriched mixture of compound **3f**.

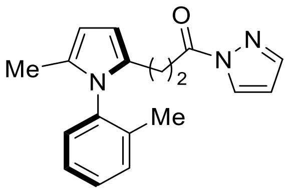

3g

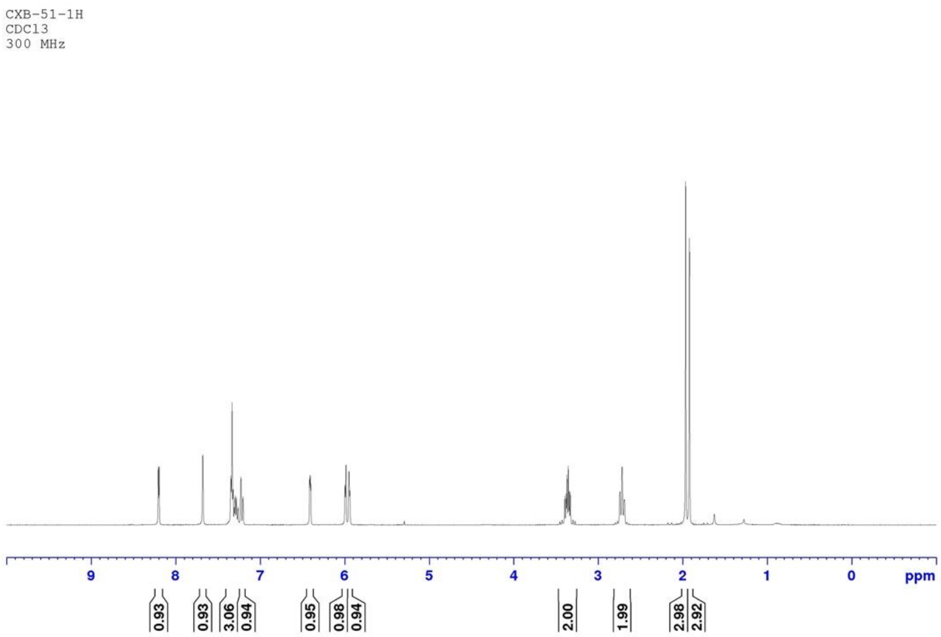

Figure S98. ^{1}H NMR ($CDCl_3$) of compound **3g**.

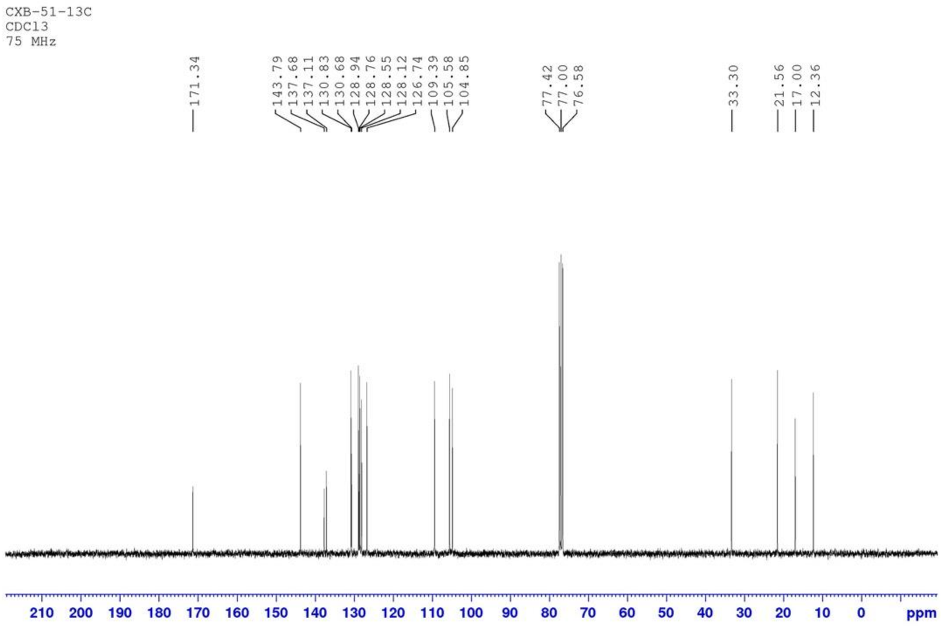

Figure S99. ^{13}C NMR ($CDCl_3$) of compound **3g**.

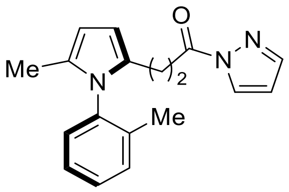

3g

[CHIRALCEL OD-H, 25 °C, *i*PrOH/hexane = 2/98 (v/v), 1.0 mL/min, 254 nm]

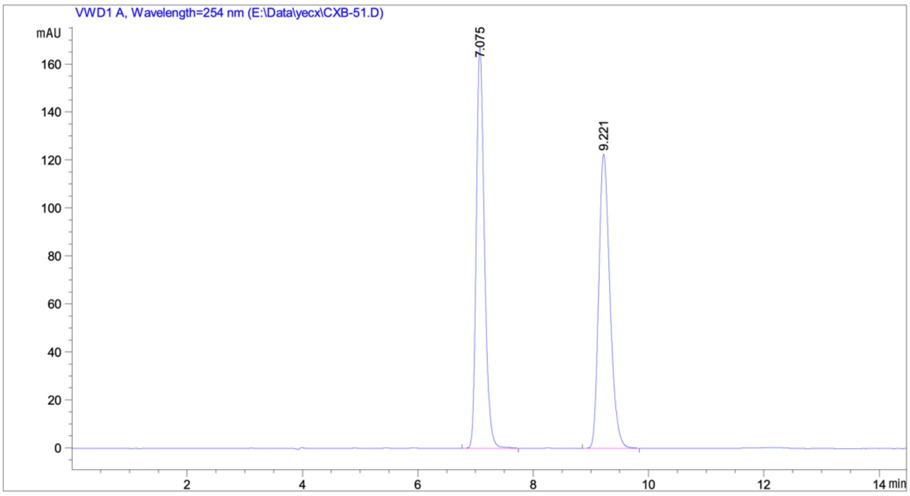

Peak #	RetTime [min]	Type	Width [min]	Area [mAU*s]	Height [mAU]	Area %
1	7.075	BB	0.1522	1652.46326	167.34598	50.0247
2	9.221	BB	0.2071	1650.82983	122.59717	49.9753

Figure S100. Racemate of compound **3g**.

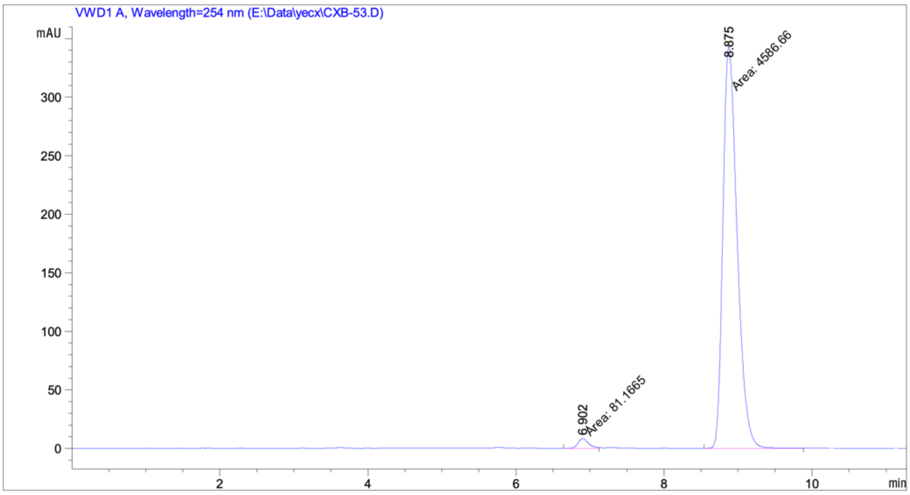

Peak #	RetTime [min]	Type	Width [min]	Area [mAU*s]	Height [mAU]	Area %
1	6.902	MF	0.1622	81.16649	8.33989	1.7388
2	8.875	MM	0.2228	4586.66162	343.10922	98.2612

Figure S101. Enantioenriched mixture of compound **3g**.

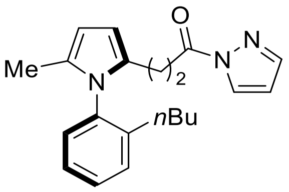

3h

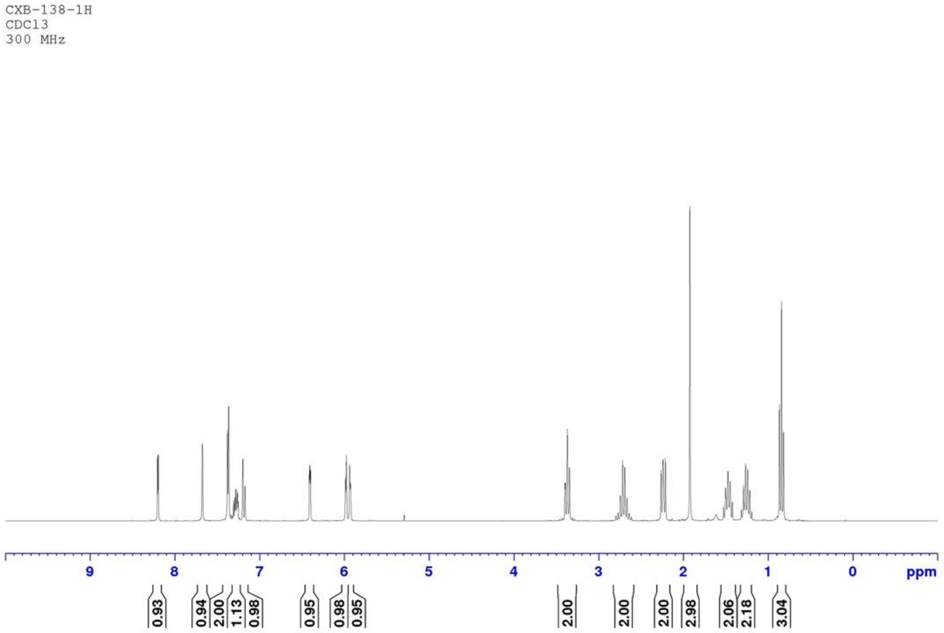

Figure S102. ^{1}H NMR ($CDCl_3$) of compound **3h**.

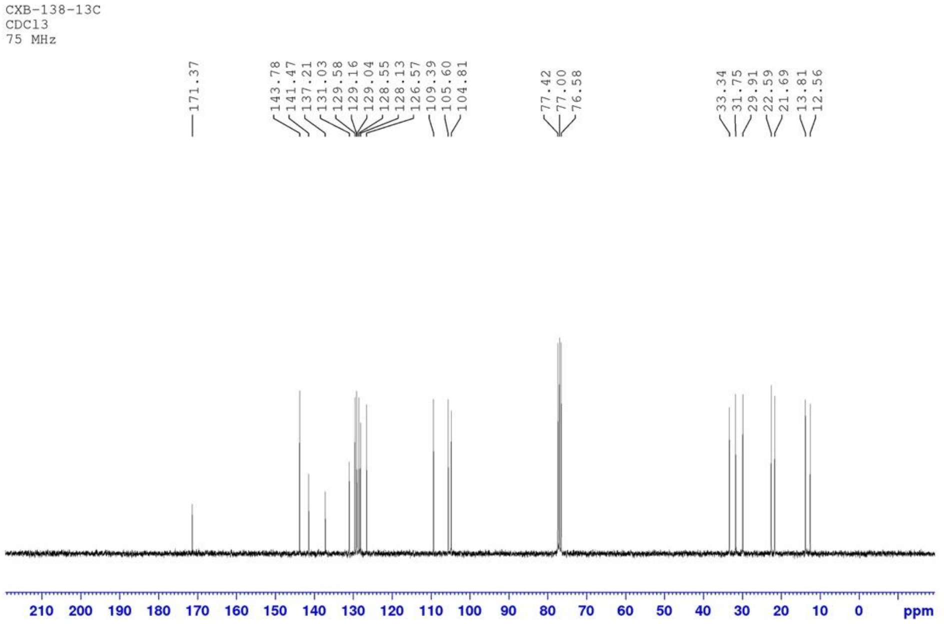

Figure S103. ^{13}C NMR ($CDCl_3$) of compound **3h**.

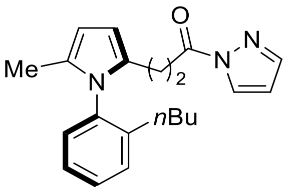

3h

[CHIRALCEL OD-H, 25 °C, *i*PrOH/hexane = 2/98 (v/v), 1.0 mL/min, 254 nm]

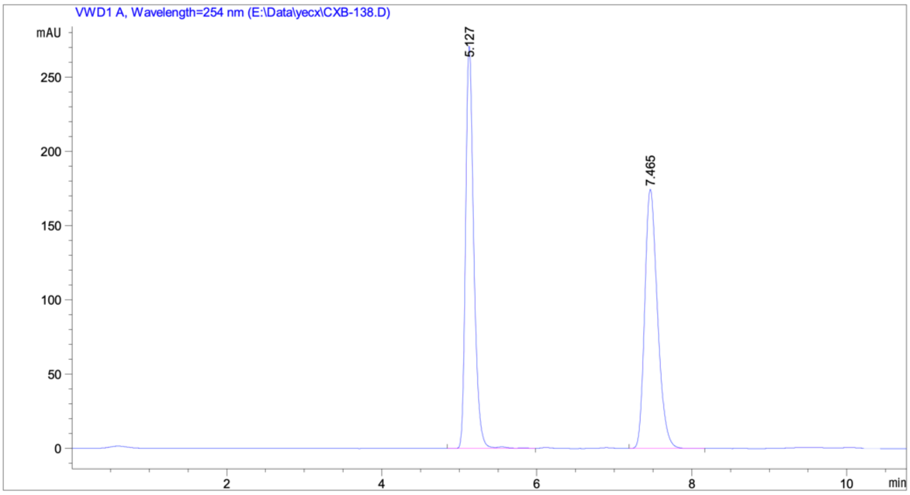

Peak #	RetTime [min]	Type	Width [min]	Area [mAU*s]	Height [mAU]	Area %
1	5.127	VV R	0.1118	1991.83313	270.70880	49.9220
2	7.465	BV R	0.1766	1998.05823	174.49200	50.0780

Figure S104. Racemate of compound **3h**.

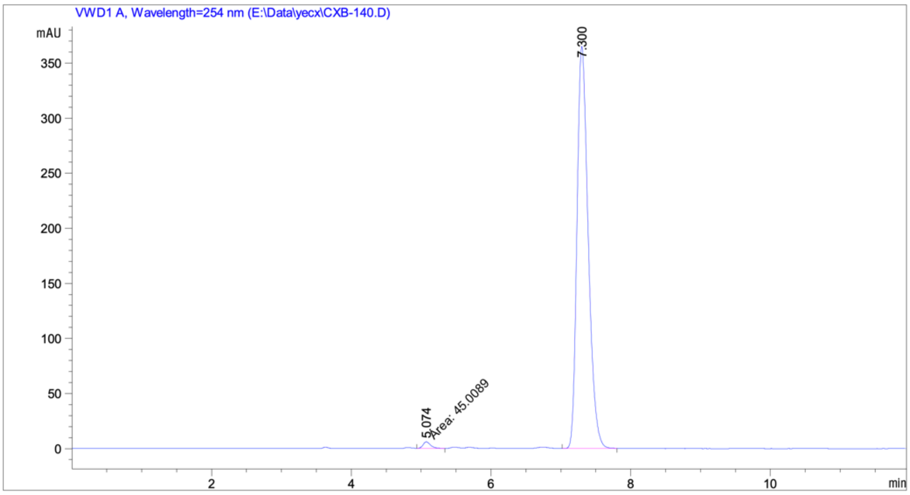

Peak #	RetTime [min]	Type	Width [min]	Area [mAU*s]	Height [mAU]	Area %
1	5.074	FM	0.1238	45.00885	6.05928	1.0938
2	7.300	BB	0.1721	4069.75269	364.91241	98.9062

Figure S105. Enantioenriched mixture of compound **3h**.

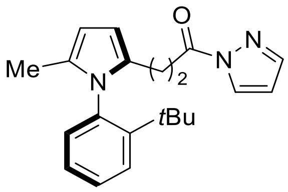

3i

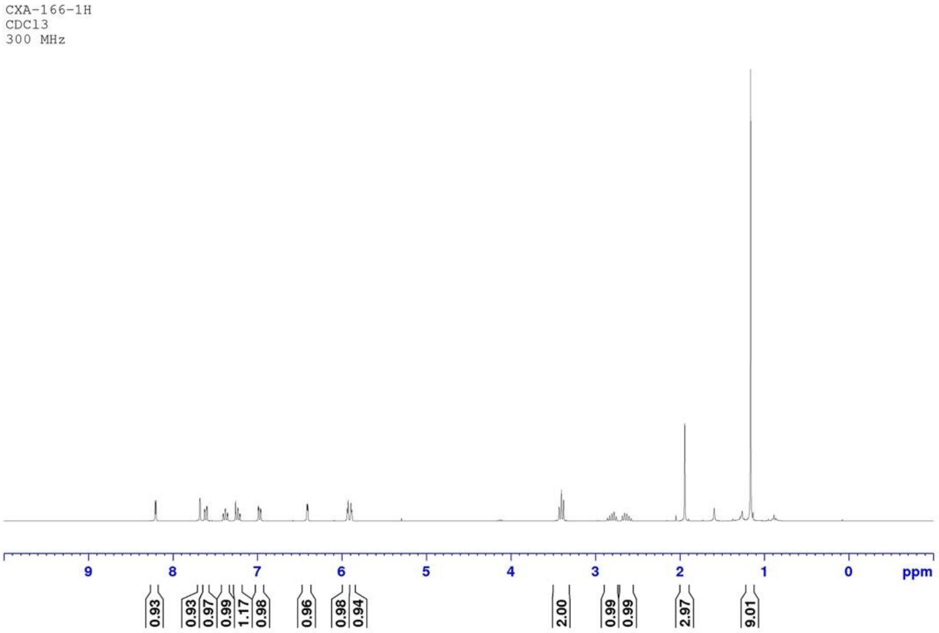

Figure S106. ^{1}H NMR ($CDCl_3$) of compound **3i**.

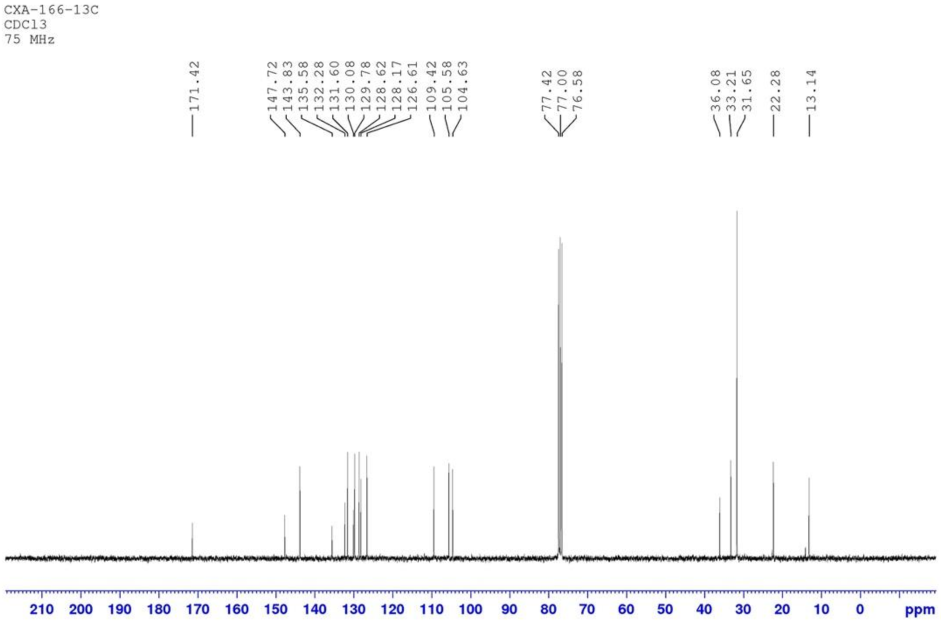

Figure S107. ^{13}C NMR ($CDCl_3$) of compound **3i**.

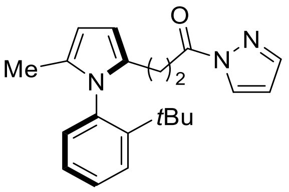

3i

[CHIRALCEL OD-H, 25 °C, *i*PrOH/hexane = 2/98 (v/v), 1.0 mL/min, 254 nm]

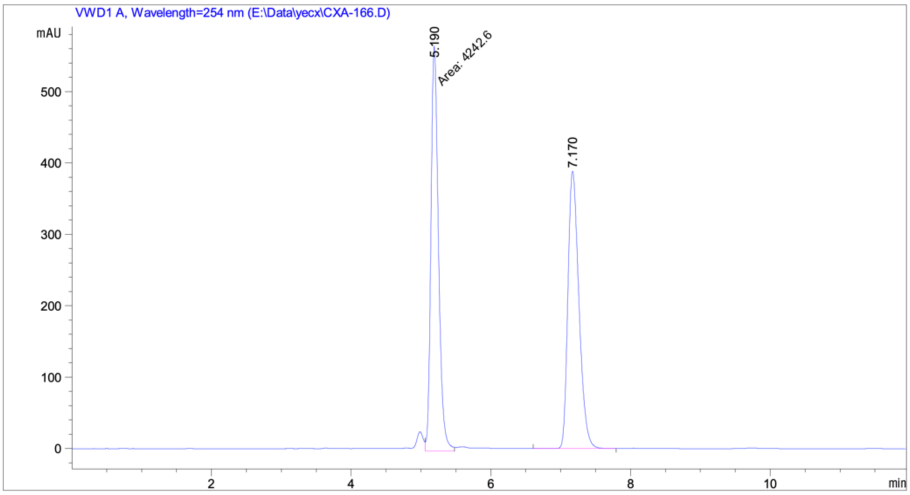

Peak #	RetTime [min]	Type	Width [min]	Area [mAU*s]	Height [mAU]	Area %
1	5.190	MM	0.1248	4242.59668	566.56812	49.9442
2	7.170	VB R	0.1698	4252.08057	388.16129	50.0558

Figure S108. Racemate of compound **3i**.

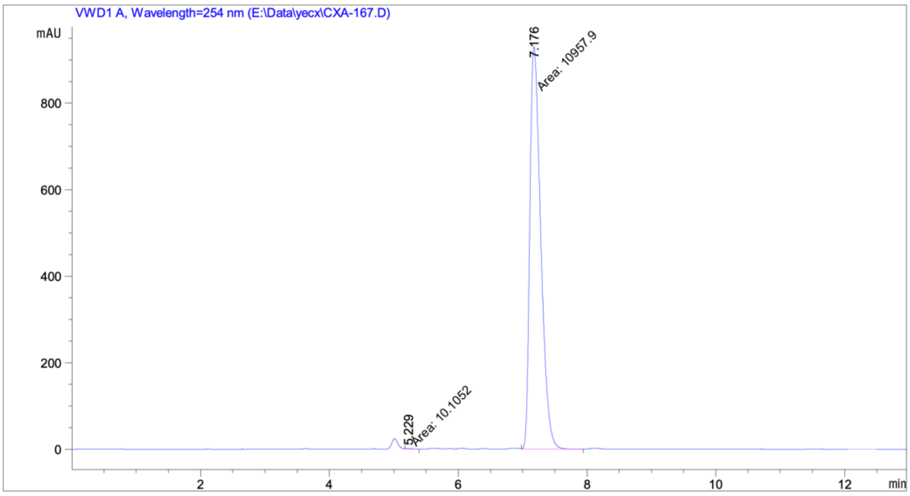

Peak #	RetTime [min]	Type	Width [min]	Area [mAU*s]	Height [mAU]	Area %
1	5.229	FM	0.1174	10.10524	1.43441	0.0921
2	7.176	FM	0.1963	1.09579e4	930.33545	99.9079

Figure S109. Enantioenriched mixture of compound **3i**.

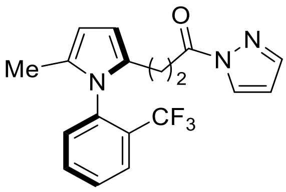

3j

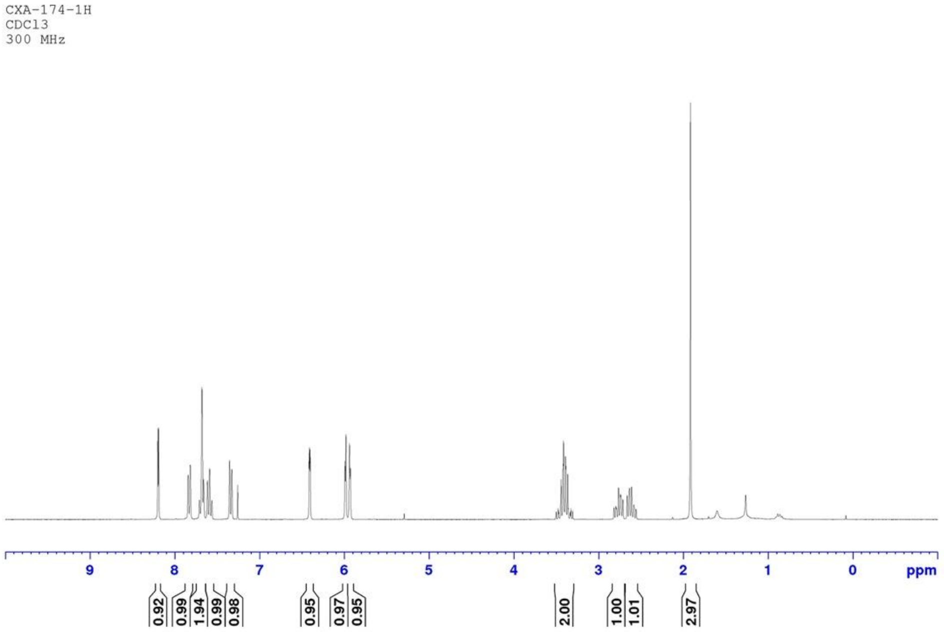

Figure S110. ^{1}H NMR ($CDCl_3$) of compound **3j**.

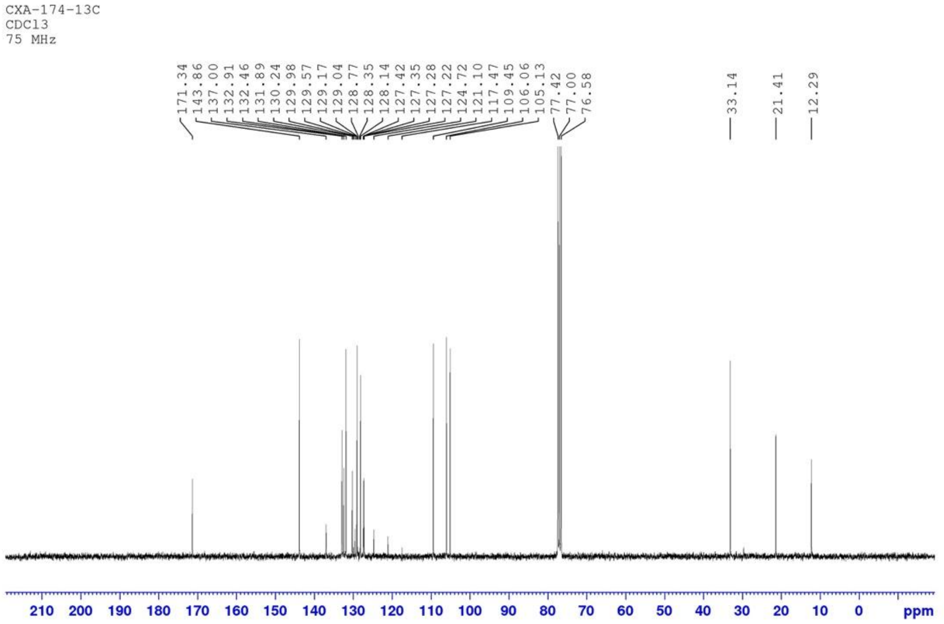

Figure S111. ^{13}C NMR ($CDCl_3$) of compound **3j**.

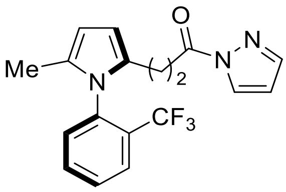

3j

[CHIRALCEL OD-H, 25 °C, *i*PrOH/hexane = 5/95 (v/v), 1.0 mL/min, 254 nm]

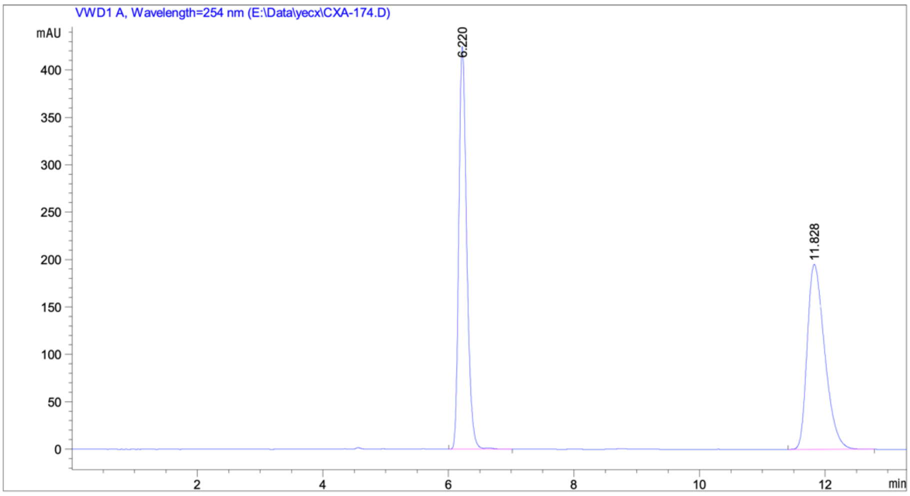

Peak #	RetTime [min]	Type	Width [min]	Area [mAU*s]	Height [mAU]	Area %
1	6.220	BV R	0.1342	3720.55322	424.77737	49.6005
2	11.828	BB	0.2990	3780.48999	195.16263	50.3995

Figure S112. Racemate of compound **3j**.

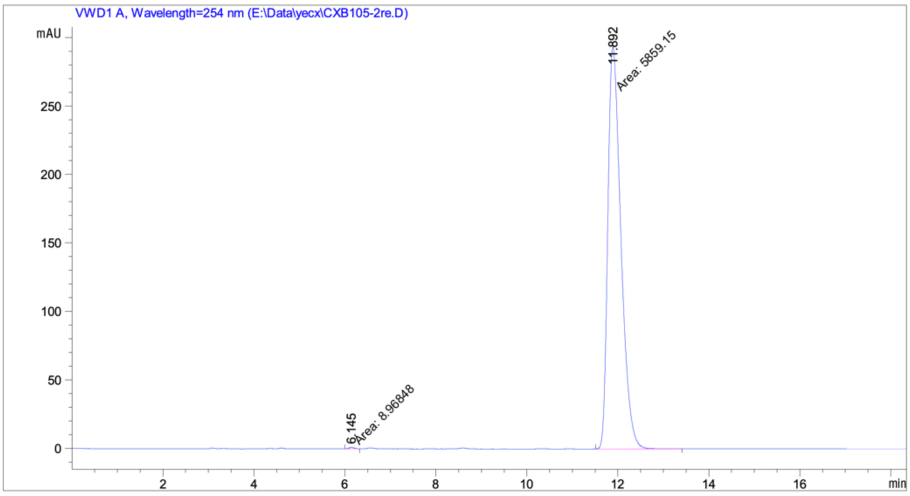

Peak #	RetTime [min]	Type	Width [min]	Area [mAU*s]	Height [mAU]	Area %
1	6.145	MM	0.1412	8.96848	1.05869	0.1528
2	11.892	MM	0.3325	5859.14795	293.64984	99.8472

Figure S113. Enantioenriched mixture of compound **3j**.

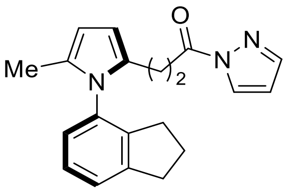

3k

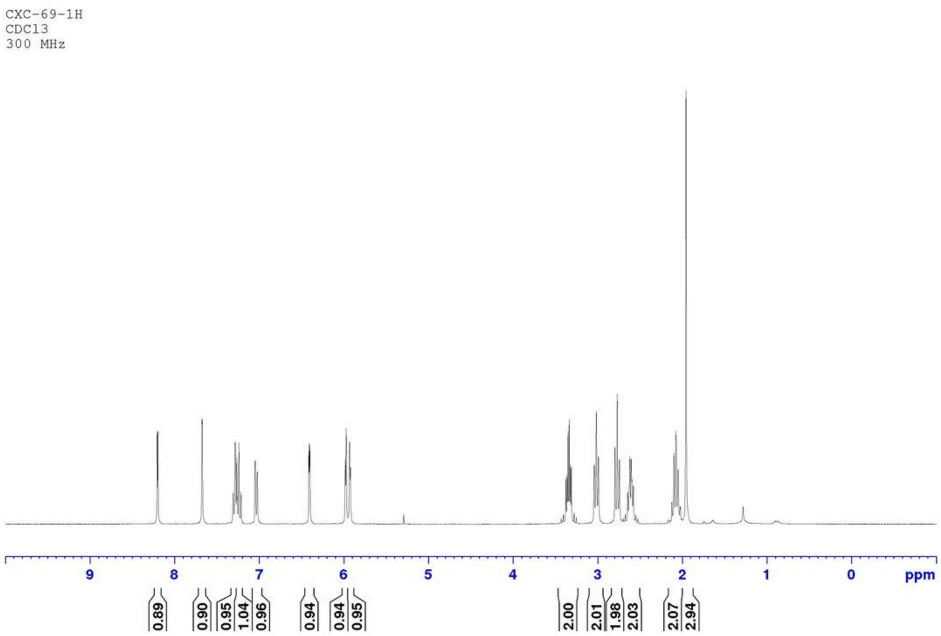

Figure S114. ^{1}H NMR ($CDCl_3$) of compound **3k**.

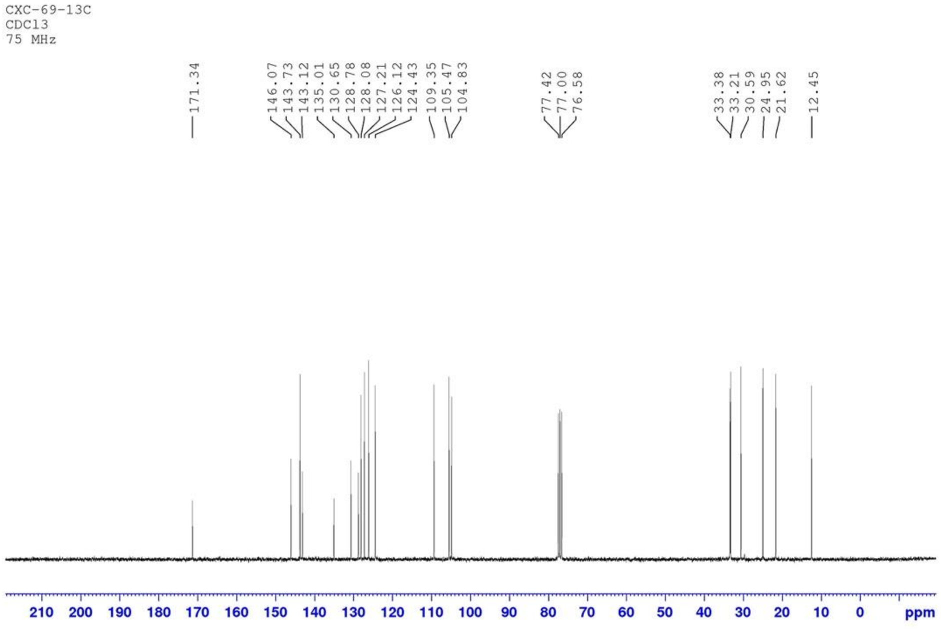

Figure S115. ^{13}C NMR ($CDCl_3$) of compound **3k**.

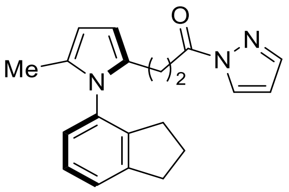

3k

[CHIRALCEL OD-H, 25 °C, *i*PrOH/hexane = 2/98 (v/v), 1.0 mL/min, 254 nm]

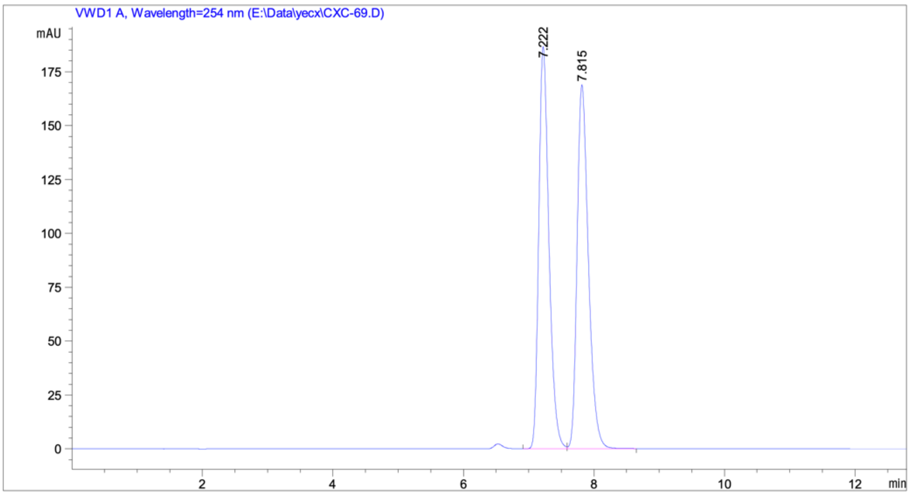

Peak #	RetTime [min]	Type	Width [min]	Area [mAU*s]	Height [mAU]	Area %
1	7.222	VV R	0.1652	2017.34045	186.47314	49.8180
2	7.815	VV R	0.1845	2032.07678	168.77789	50.1820

Figure S116. Racemate of compound **3k**.

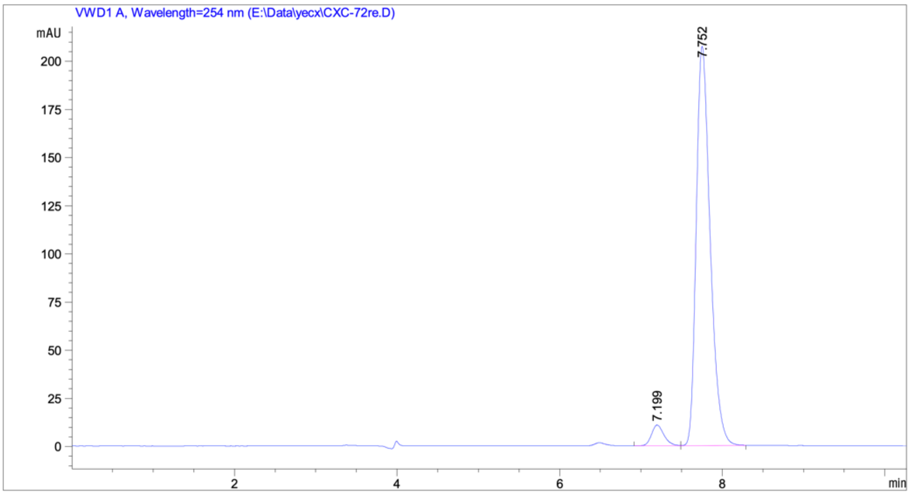

Peak #	RetTime [min]	Type	Width [min]	Area [mAU*s]	Height [mAU]	Area %
1	7.199	BV	0.1611	113.30573	10.74228	4.3983
2	7.752	VB	0.1827	2462.81055	207.12704	95.6017

Figure S117. Enantioenriched mixture of compound **3k**.

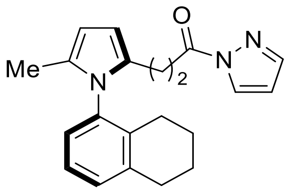

3l

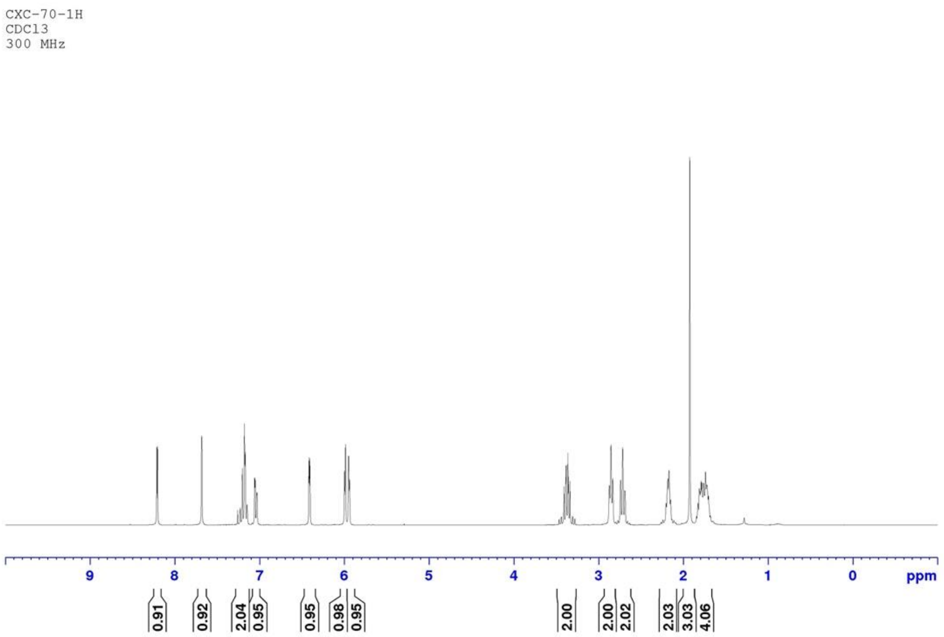

Figure S118. ^{1}H NMR ($CDCl_3$) of compound **3l**.

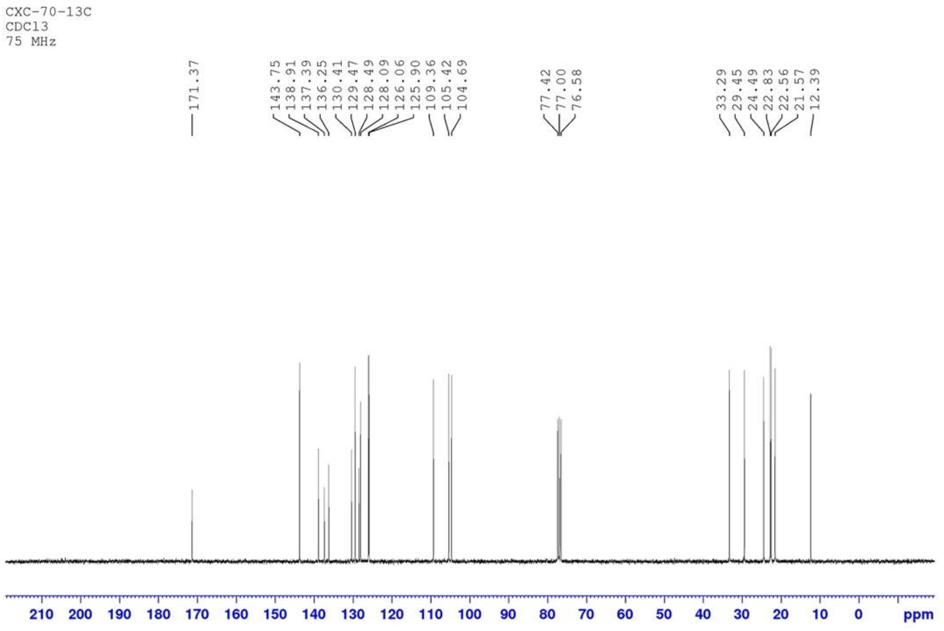

Figure S119. ^{13}C NMR ($CDCl_3$) of compound **3l**.

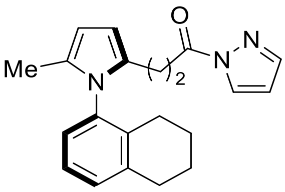

3l

[CHIRALCEL OD-H, 25 °C, *i*PrOH/hexane = 2/98 (v/v), 1.0 mL/min, 254 nm]

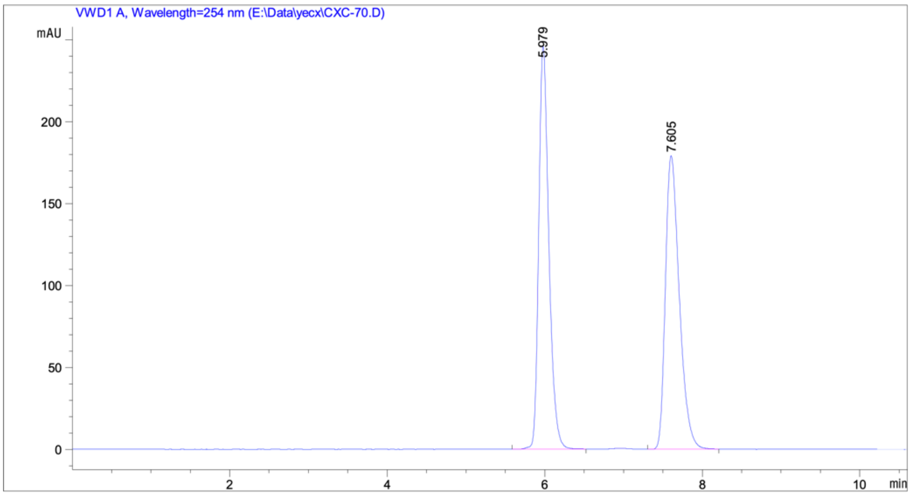

Peak #	RetTime [min]	Type	Width [min]	Area [mAU*s]	Height [mAU]	Area %
1	5.979	BB	0.1361	2190.06104	245.50771	49.9499
2	7.605	BB	0.1880	2194.45166	179.04564	50.0501

Figure S120. Racemate of compound **3l**.

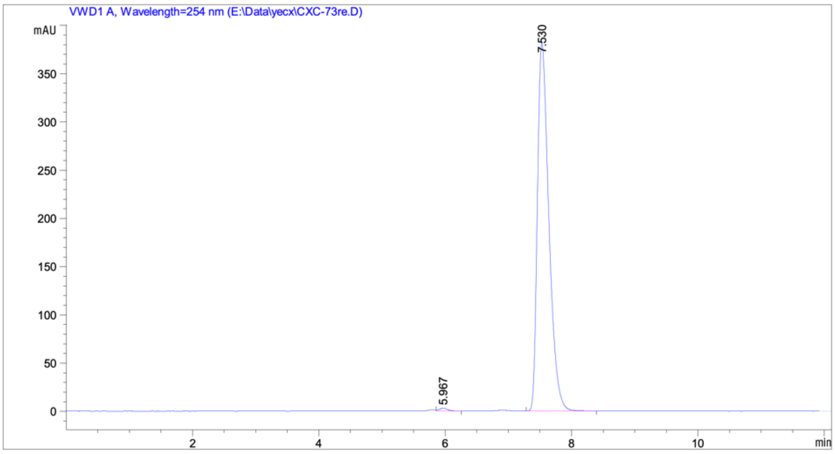

Peak #	RetTime [min]	Type	Width [min]	Area [mAU*s]	Height [mAU]	Area %
1	5.967	VB	0.1385	26.54969	2.93463	0.5497
2	7.530	BB	0.1929	4803.25977	381.51334	99.4503

Figure S121. Enantioenriched mixture of compound **3l**.

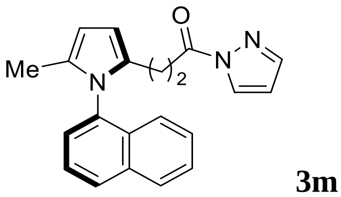

3m

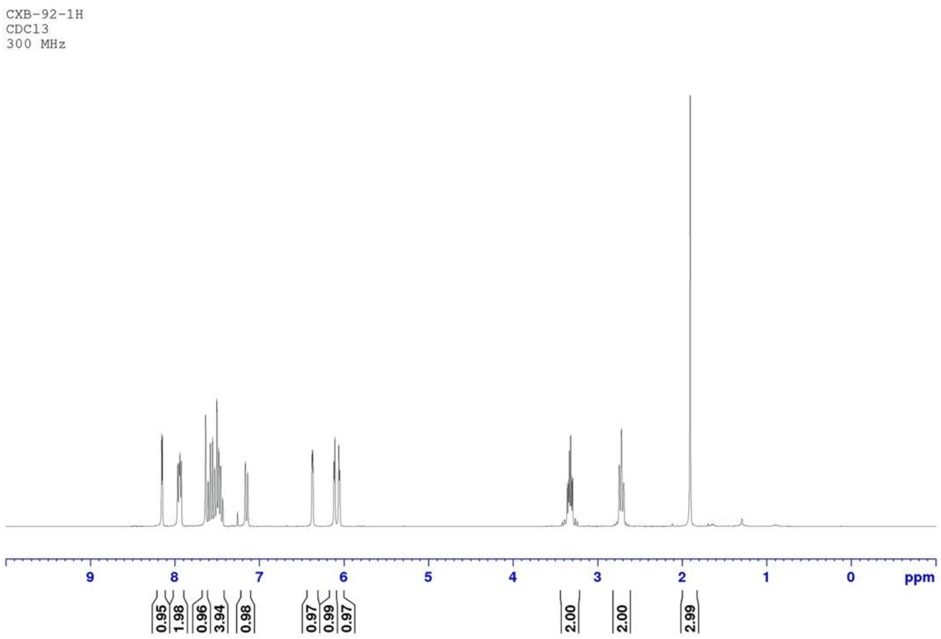

Figure S122. ^{1}H NMR ($CDCl_3$) of compound **3m**.

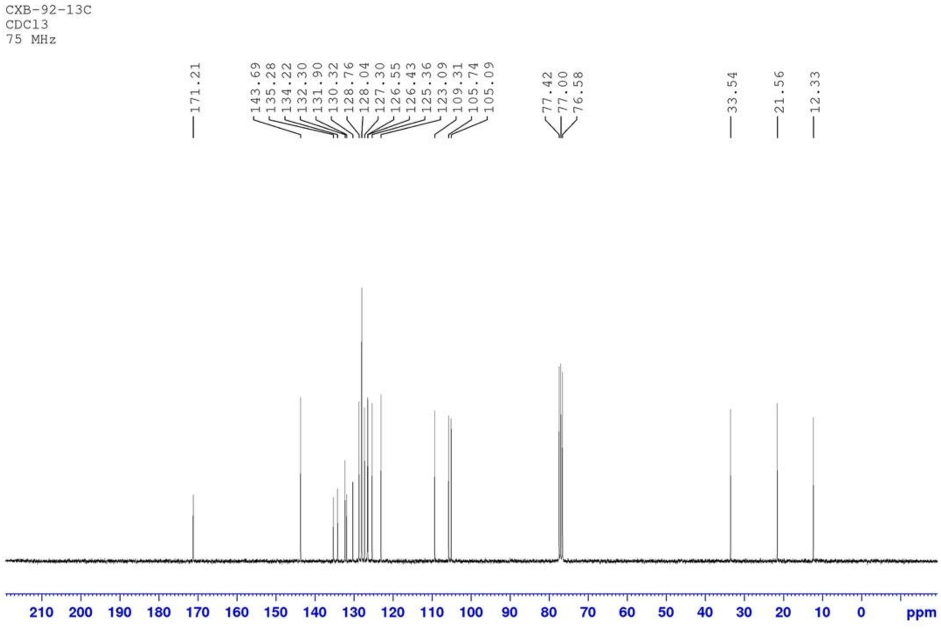

Figure S123. ^{13}C NMR ($CDCl_3$) of compound **3m**.

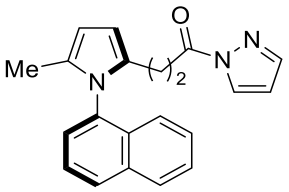

3m

[CHIRALCEL OD-H, 25 °C, *i*PrOH/hexane = 5/95 (v/v), 1.0 mL/min, 254 nm]

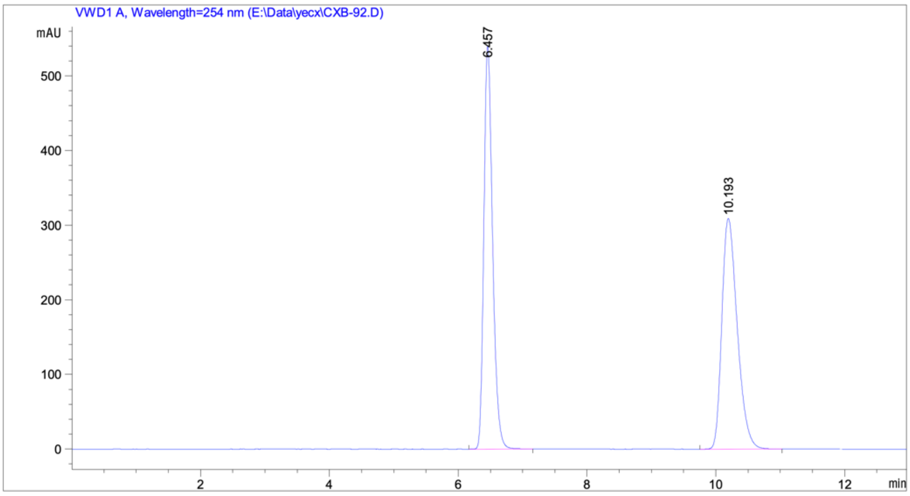

Peak #	RetTime [min]	Type	Width [min]	Area [mAU*s]	Height [mAU]	Area %
1	6.457	BV	0.1441	5091.81787	539.47314	49.8078
2	10.193	BB	0.2568	5131.12061	308.95670	50.1922

Figure S124. Racemate of compound **3m**.

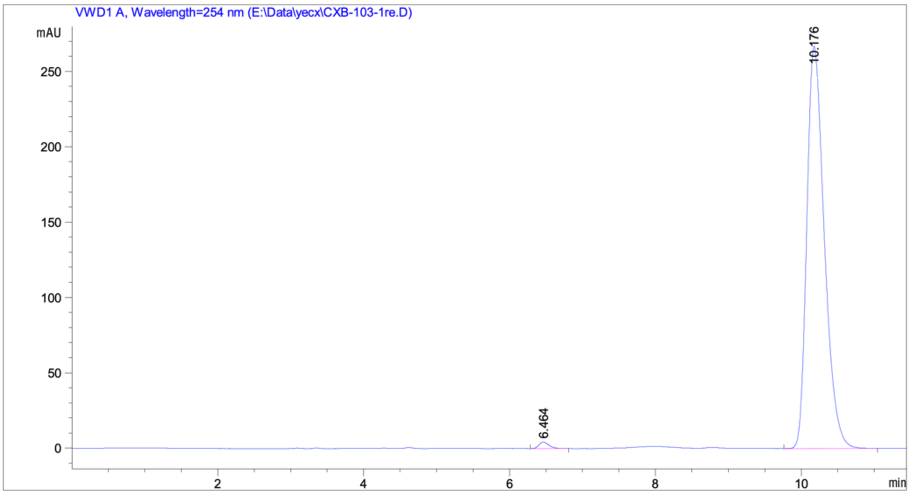

Peak #	RetTime [min]	Type	Width [min]	Area [mAU*s]	Height [mAU]	Area %
1	6.464	BB	0.1446	41.06021	4.33111	0.9151
2	10.176	BB	0.2554	4445.68896	266.81372	99.0849

Figure S125. Enantioenriched mixture of compound **3m**.

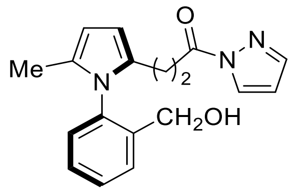

3n

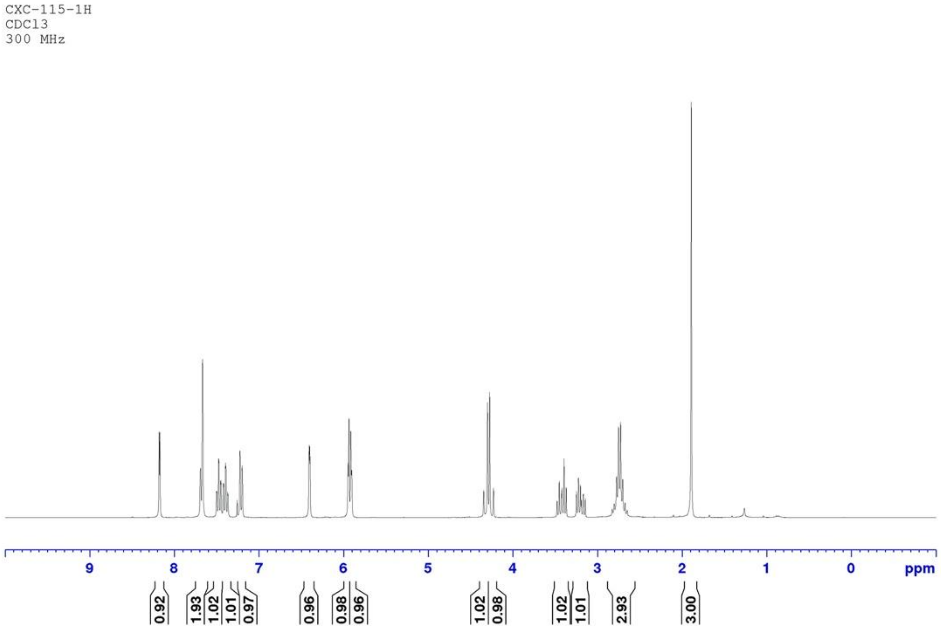

Figure S126. ^{1}H NMR ($CDCl_3$) of compound **3n**.

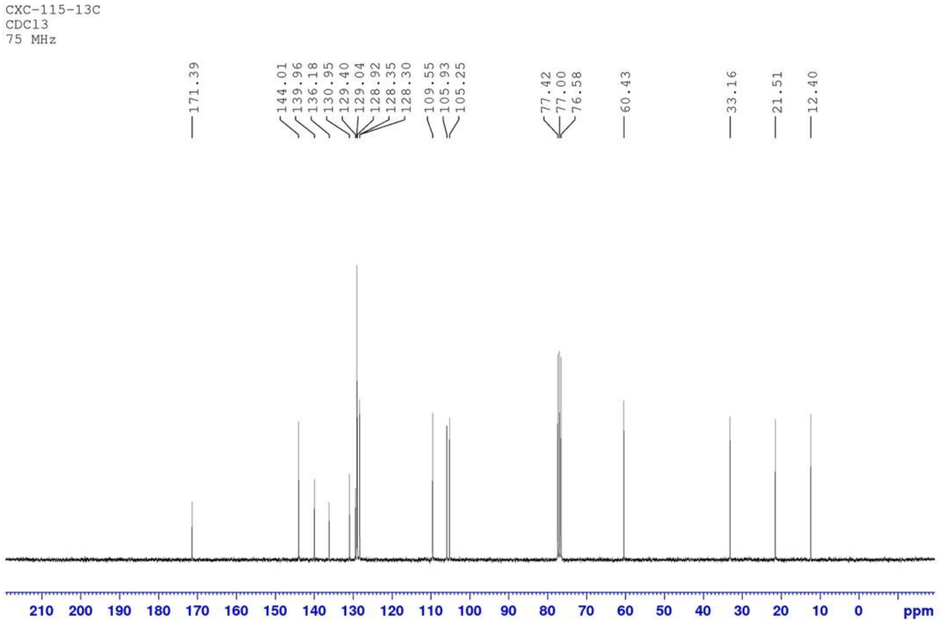

Figure S127. ^{13}C NMR ($CDCl_3$) of compound **3n**.

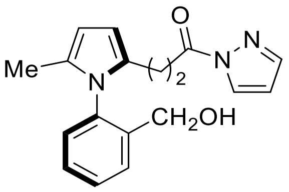

3n

[CHIRALPAK IG, 25 °C, *i*PrOH/hexane = 20/80 (v/v), 1.0 mL/min, 254 nm]

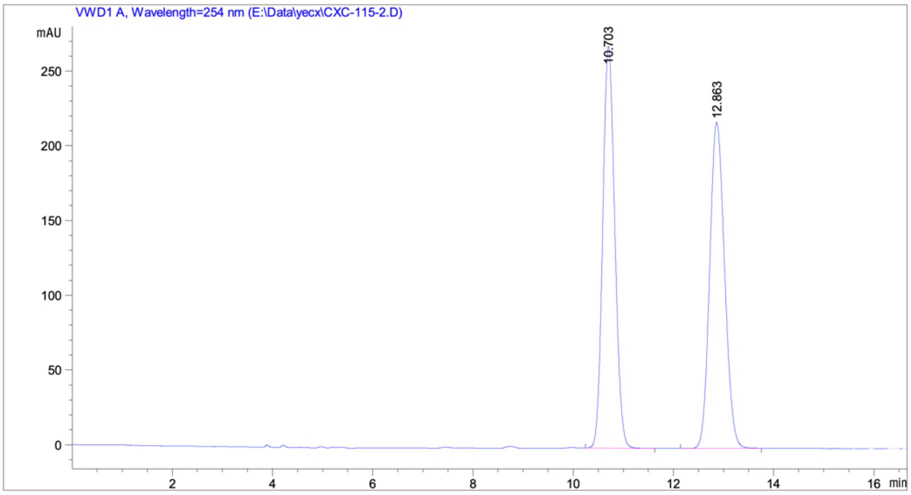

Peak #	RetTime [min]	Type	Width [min]	Area [mAU*s]	Height [mAU]	Area %
1	10.703	BV R	0.2612	4497.89404	268.82132	49.9744
2	12.863	VB R	0.3216	4502.49463	218.31296	50.0256

Figure S128. Racemate of compound **3n**.

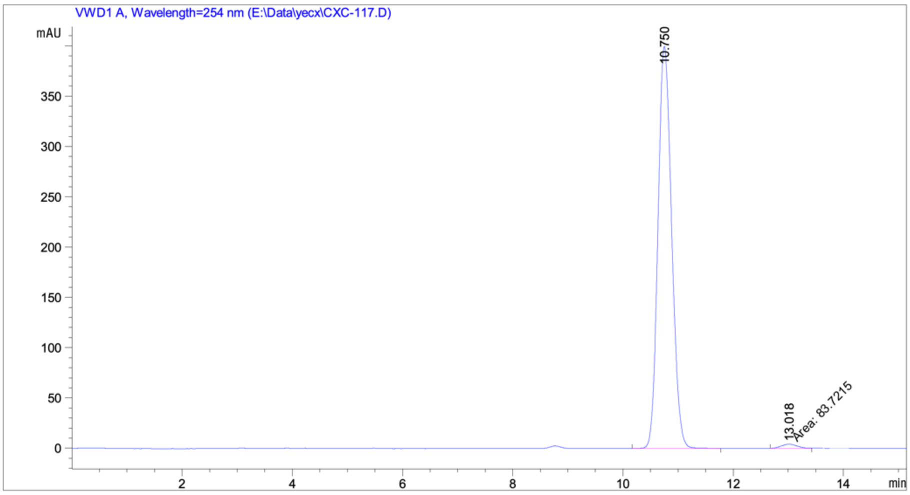

Peak #	RetTime [min]	Type	Width [min]	Area [mAU*s]	Height [mAU]	Area %
1	10.750	BV R	0.2687	6875.50684	399.66144	98.7970
2	13.018	MM	0.3347	83.72150	4.16904	1.2030

Figure S129. Enantioenriched mixture of compound **3n**.

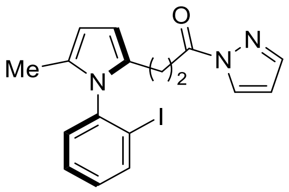

3o

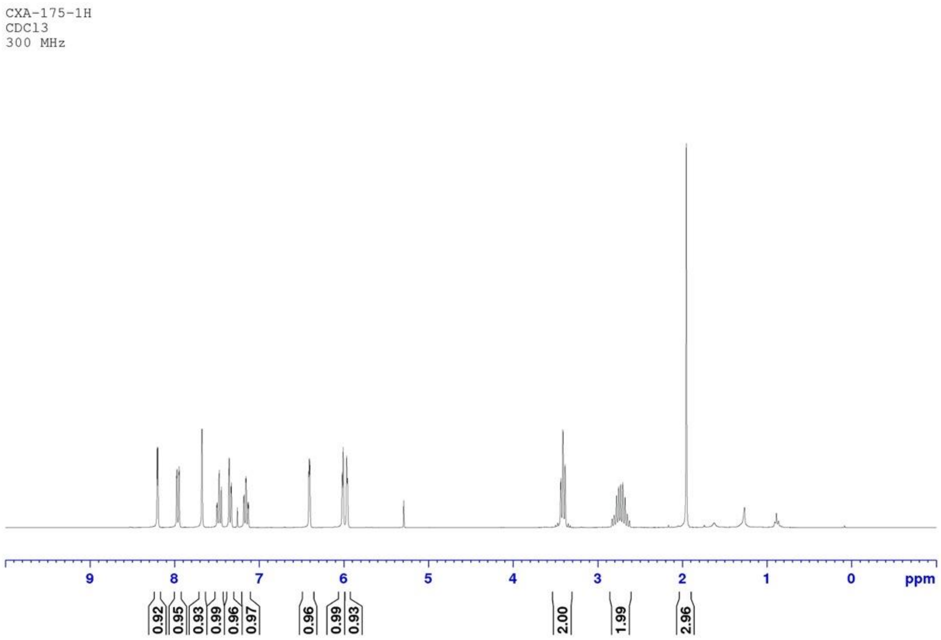

Figure S130. ^{1}H NMR ($CDCl_3$) of compound **3o**.

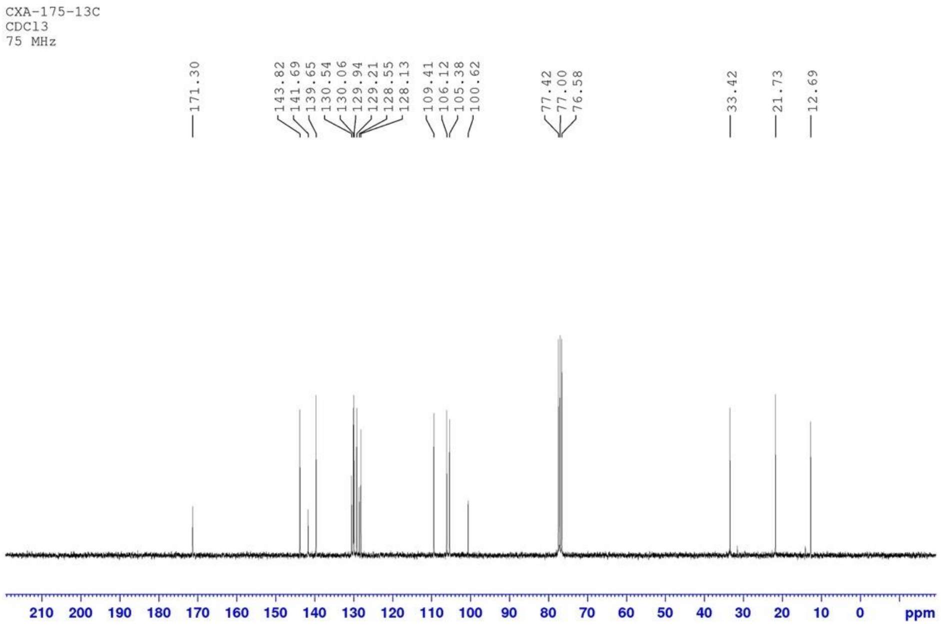

Figure S131. ^{13}C NMR ($CDCl_3$) of compound **3o**.

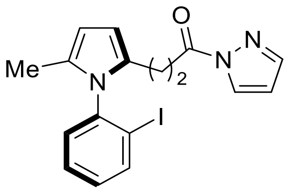

3o

[CHIRALCEL OD-H, 25 °C, *i*PrOH/hexane = 5/95 (v/v), 1.0 mL/min, 254 nm]

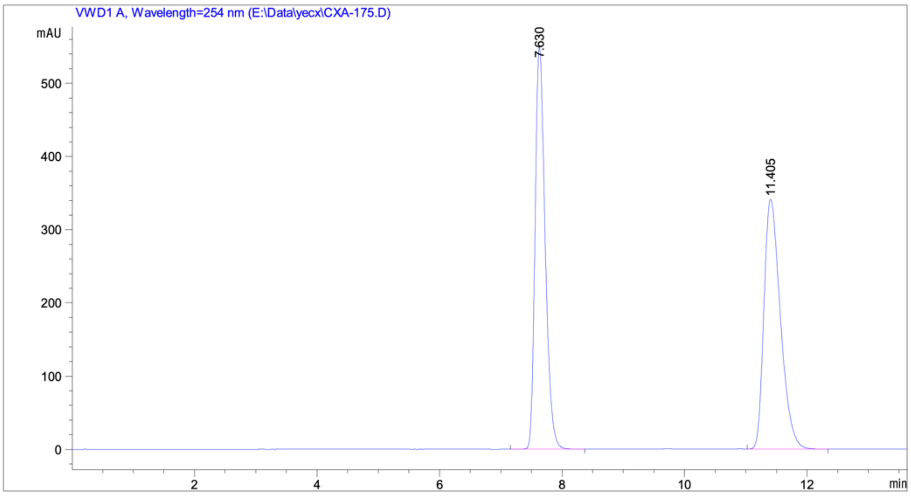

Peak #	RetTime [min]	Type	Width [min]	Area [mAU*s]	Height [mAU]	Area %
1	7.630	BB	0.1750	6267.07227	549.87085	49.5916
2	11.405	BB	0.2889	6370.29053	341.04092	50.4084

Figure S132. Racemate of compound **3o**.

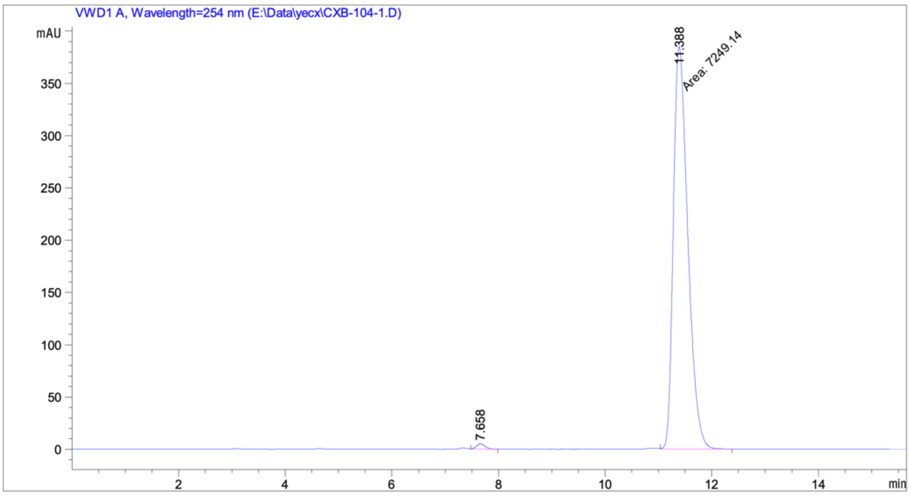

Peak #	RetTime [min]	Type	Width [min]	Area [mAU*s]	Height [mAU]	Area %
1	7.658	VB	0.1703	56.83191	5.05128	0.7779
2	11.388	FM	0.3140	7249.13623	384.81876	99.2221

Figure S133. Enantioenriched mixture of compound **3o**.

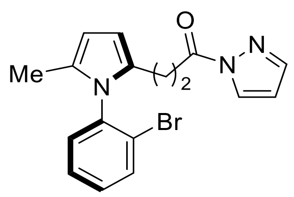

3p

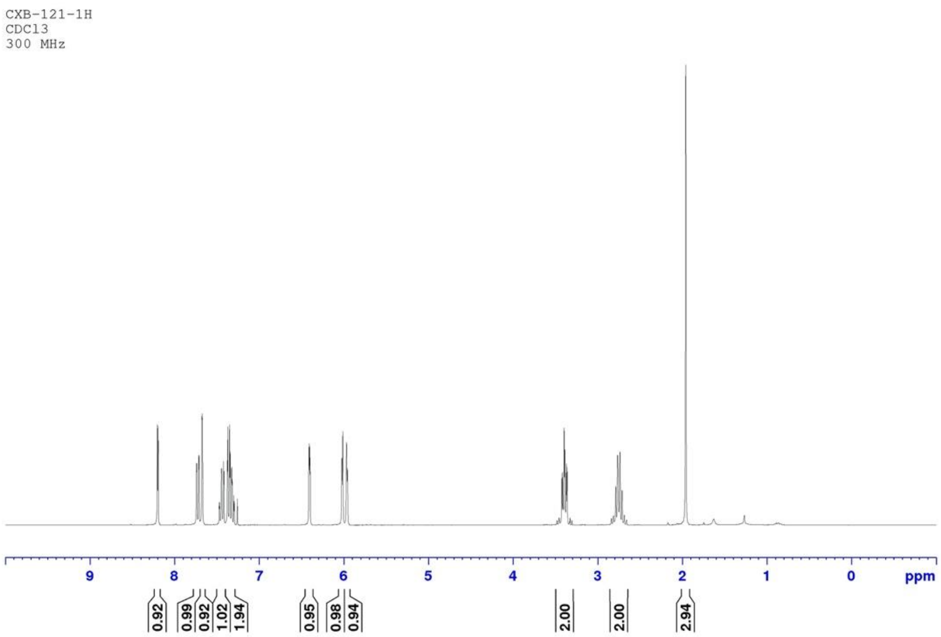

Figure S134. ^{1}H NMR ($CDCl_3$) of compound **3p**.

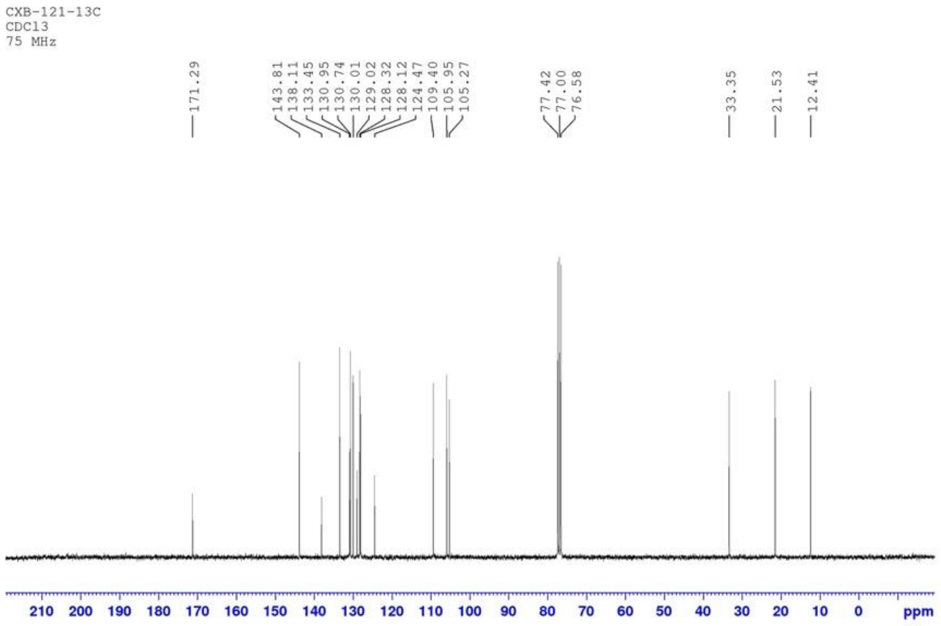

Figure S135. ^{13}C NMR ($CDCl_3$) of compound **3p**.

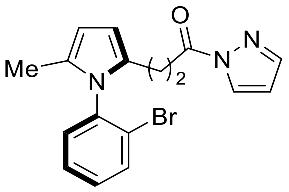

3p

[CHIRALCEL OD-H, 25 °C, *i*PrOH/hexane = 5/95 (v/v), 1.0 mL/min, 254 nm]

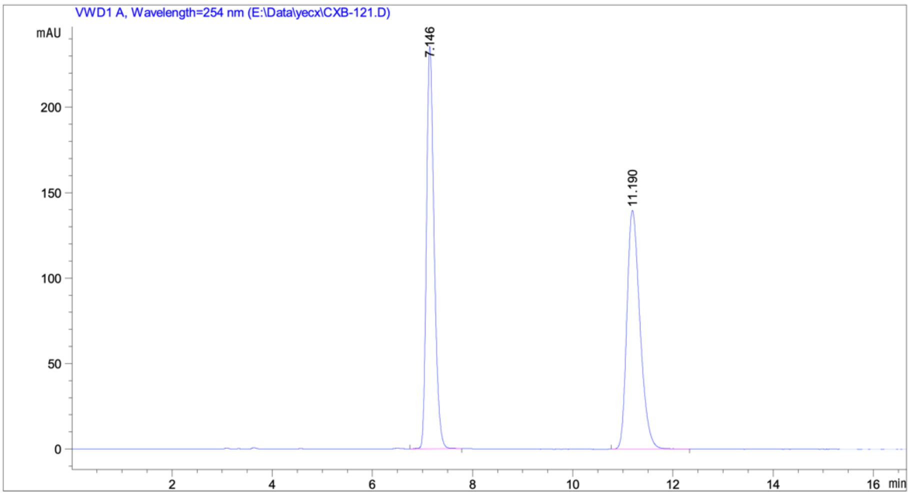

Peak #	RetTime [min]	Type	Width [min]	Area [mAU*s]	Height [mAU]	Area %
1	7.146	BB	0.1629	2500.52124	235.42589	49.6983
2	11.190	BV R	0.2783	2530.88379	139.72812	50.3017

Figure S136. Racemate of compound **3p**.

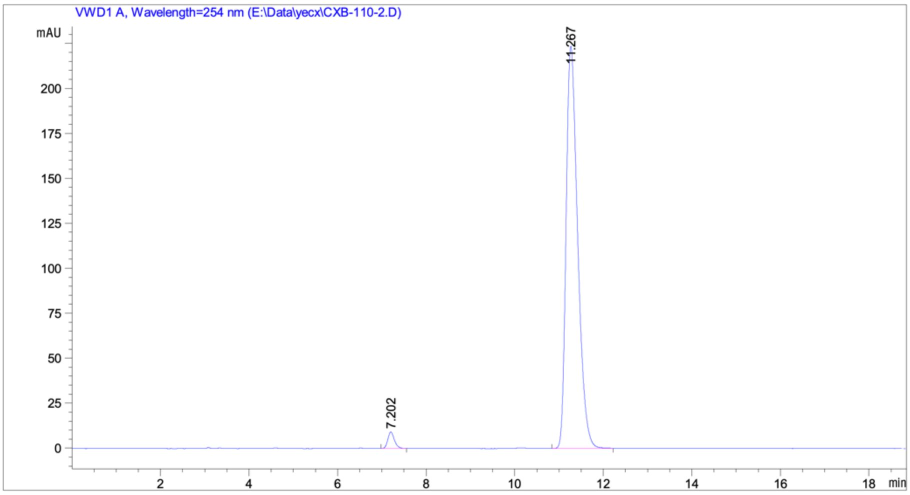

Peak #	RetTime [min]	Type	Width [min]	Area [mAU*s]	Height [mAU]	Area %
1	7.202	BB	0.1577	93.58282	9.12381	2.2576
2	11.267	BB	0.2785	4051.69604	223.47353	97.7424

Figure S137. Enantioenriched mixture of compound **3p**.

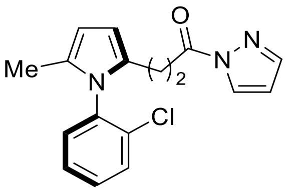

3q

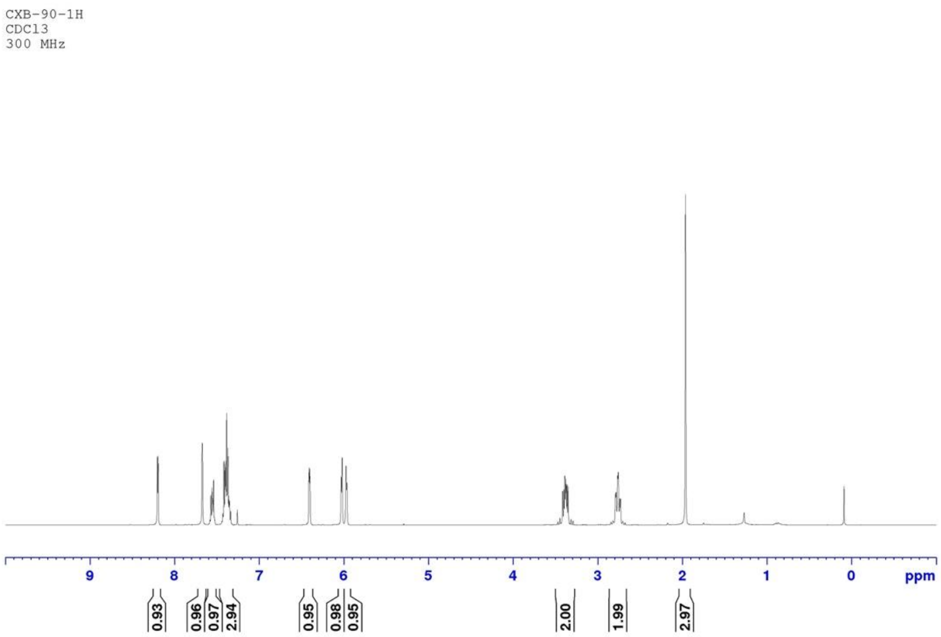

Figure S138. ^{1}H NMR ($CDCl_3$) of compound **3q**.

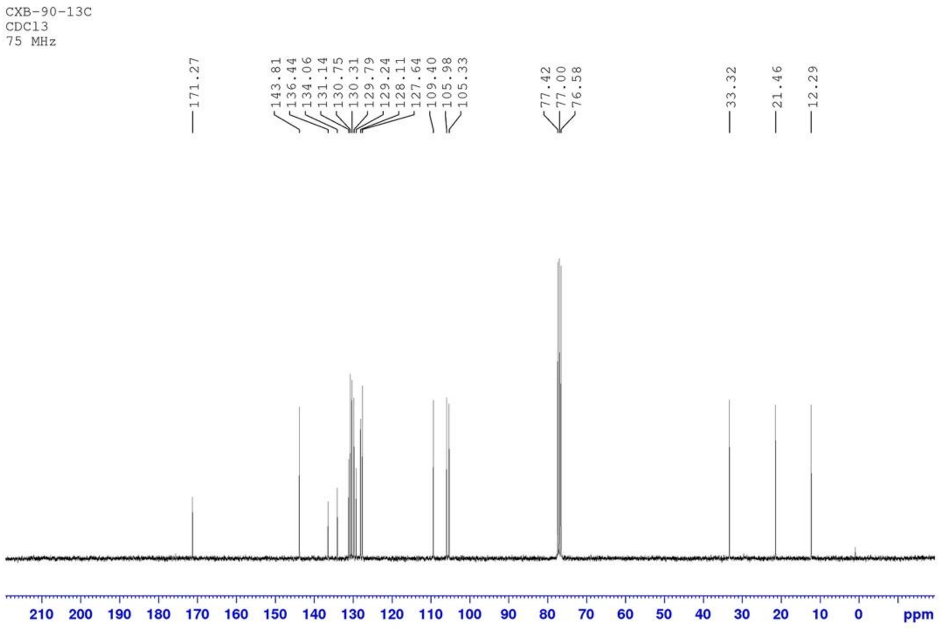

Figure S139. ^{13}C NMR ($CDCl_3$) of compound **3q**.

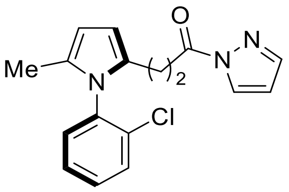

3q

[CHIRALCEL OD-H, 25 °C, *i*PrOH/hexane = 5/95 (v/v), 1.0 mL/min, 254 nm]

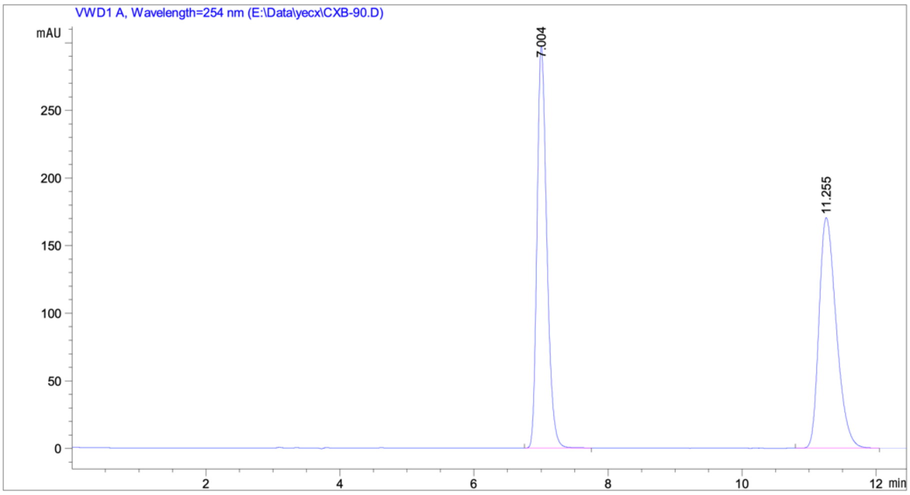

Peak #	RetTime [min]	Type	Width [min]	Area [mAU*s]	Height [mAU]	Area %
1	7.004	BB	0.1553	3011.11890	296.92722	49.7194
2	11.255	BB	0.2765	3045.10278	170.39160	50.2806

Figure S140. Racemate of compound **3q**.

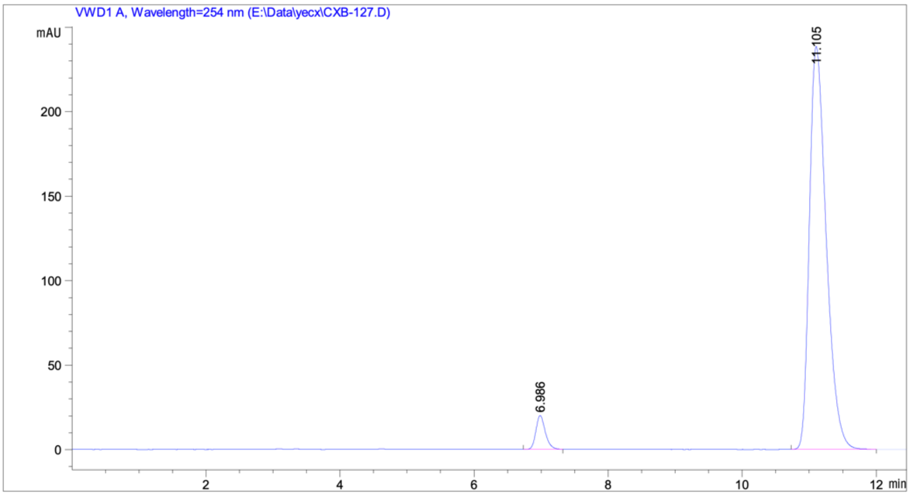

Peak #	RetTime [min]	Type	Width [min]	Area [mAU*s]	Height [mAU]	Area %
1	6.986	BB	0.1485	194.42029	19.98570	4.4656
2	11.105	BB	0.2685	4159.31885	238.41685	95.5344

Figure S141. Enantioenriched mixture of compound **3q**.

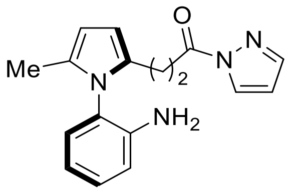

3r

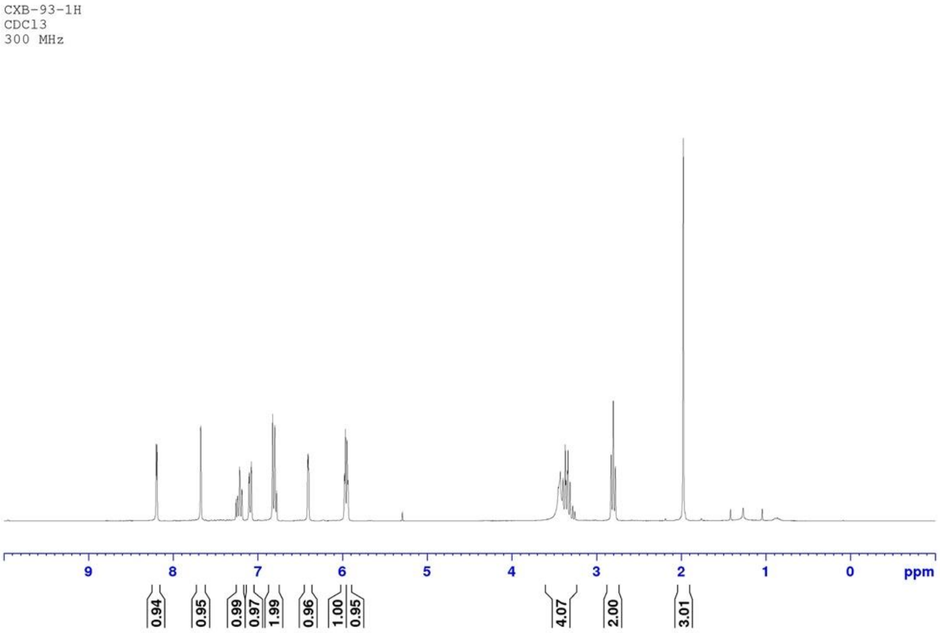

Figure S142. ¹H NMR ($CDCl_3$) of compound **3r**.

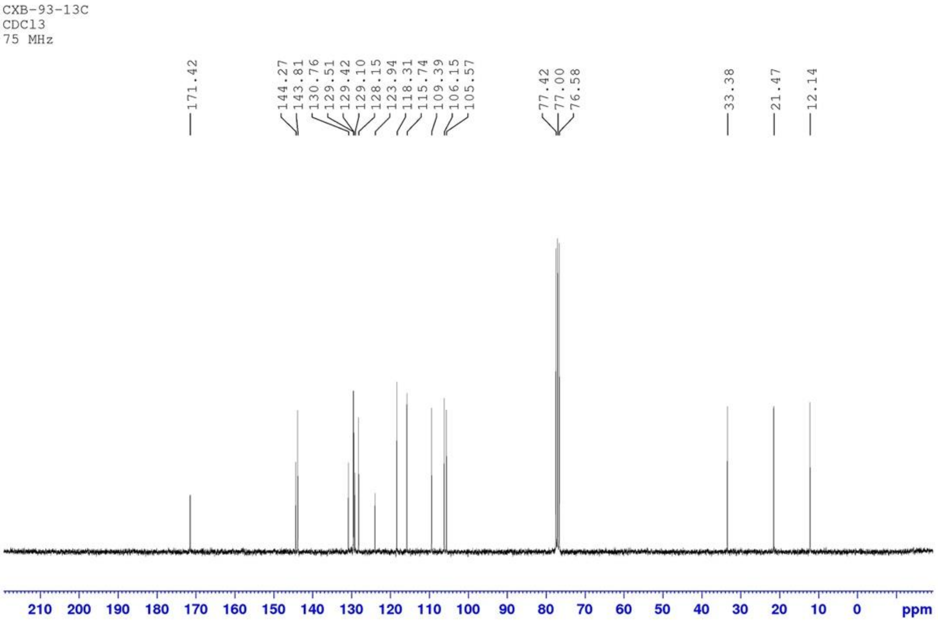

Figure S143. ¹³C NMR ($CDCl_3$) of compound **3r**.

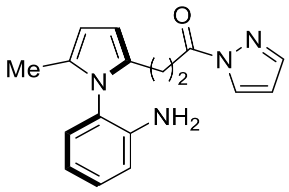

3r

[CHIRALCEL OD-H, 25 °C, *i*PrOH/hexane = 10/90 (v/v), 1.0 mL/min, 254 nm]

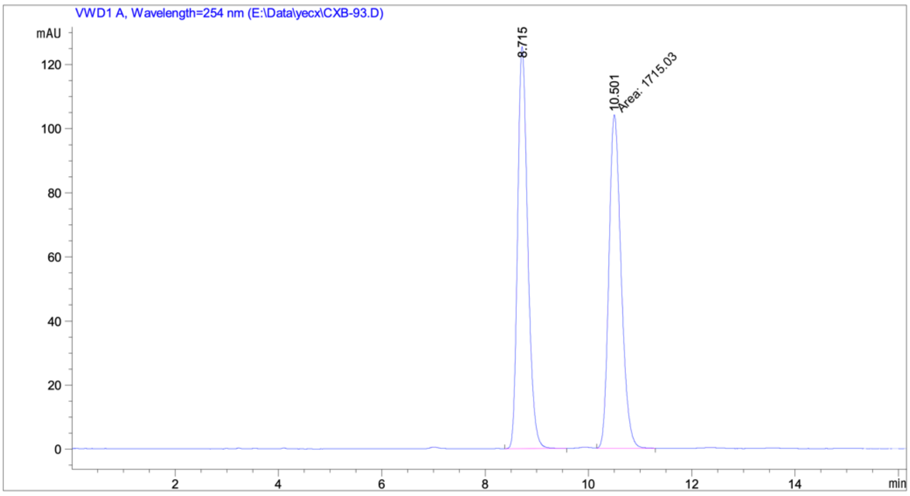

Peak #	RetTime [min]	Type	Width [min]	Area [mAU*s]	Height [mAU]	Area %
1	8.715	BV R	0.2100	1708.24988	125.35653	49.9010
2	10.501	FM	0.2748	1715.02966	104.00482	50.0990

Figure S144. Racemate of compound **3r**.

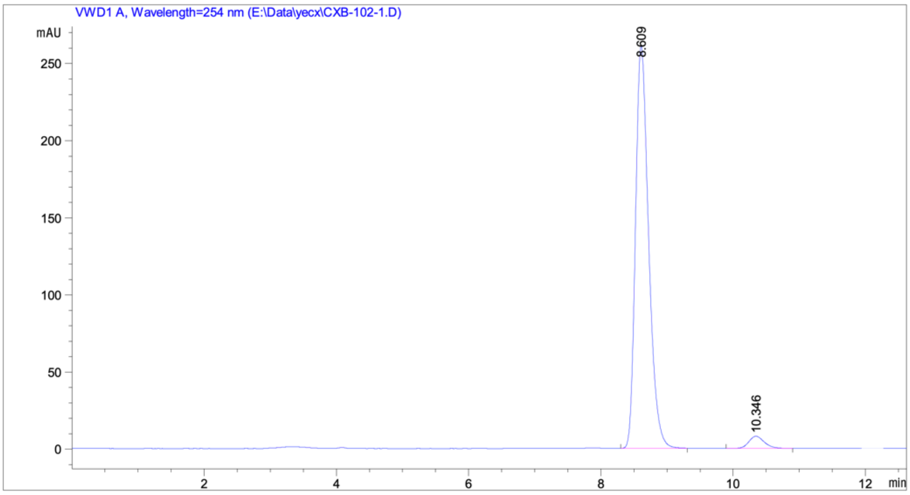

Peak #	RetTime [min]	Type	Width [min]	Area [mAU*s]	Height [mAU]	Area %
1	8.609	BB	0.2119	3594.28003	260.61368	96.5663
2	10.346	BB	0.2440	127.80503	7.88923	3.4337

Figure S145. Enantioenriched mixture of compound **3r**.

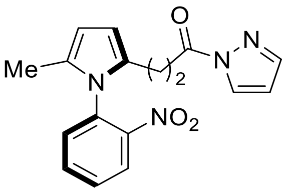

3s

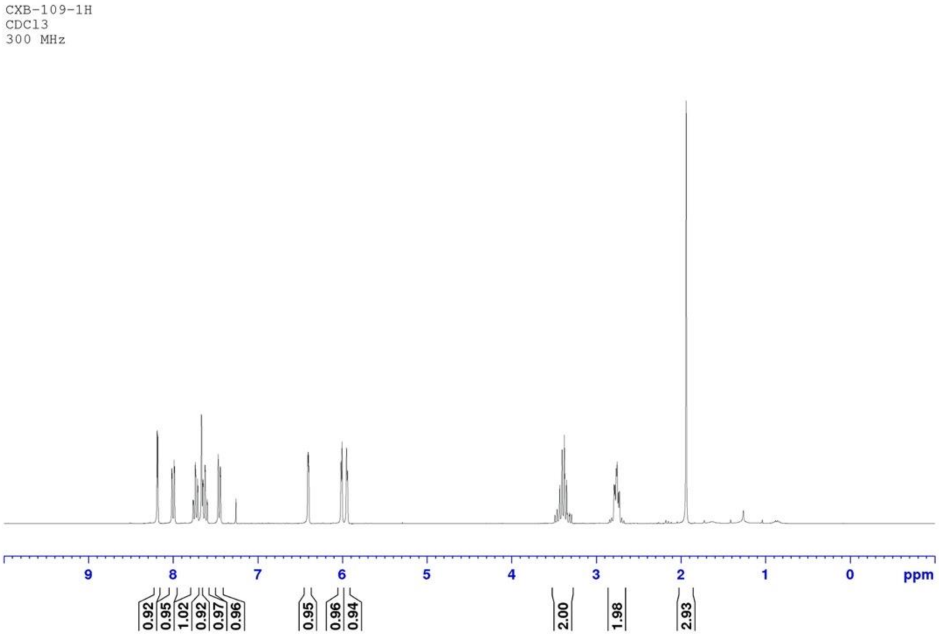

Figure S146. ^{1}H NMR ($CDCl_3$) of compound **3s**.

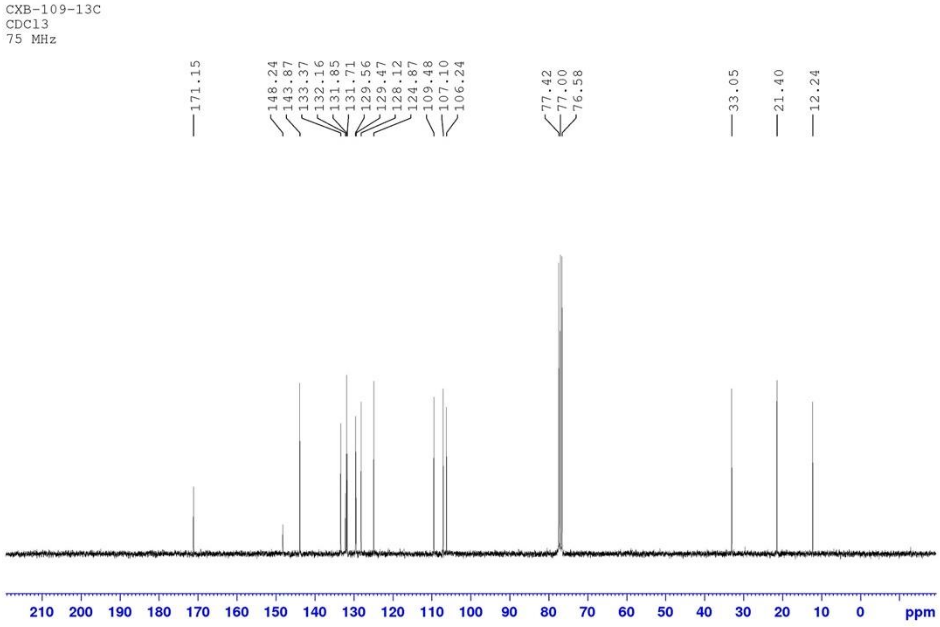

Figure S147. ^{13}C NMR ($CDCl_3$) of compound **3s**.

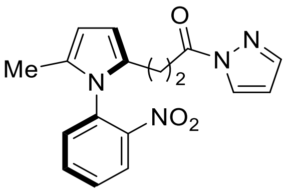

3s

[CHIRALCEL OD-H, 25 °C, *i*PrOH/hexane = 20/80 (v/v), 1.0 mL/min, 254 nm]

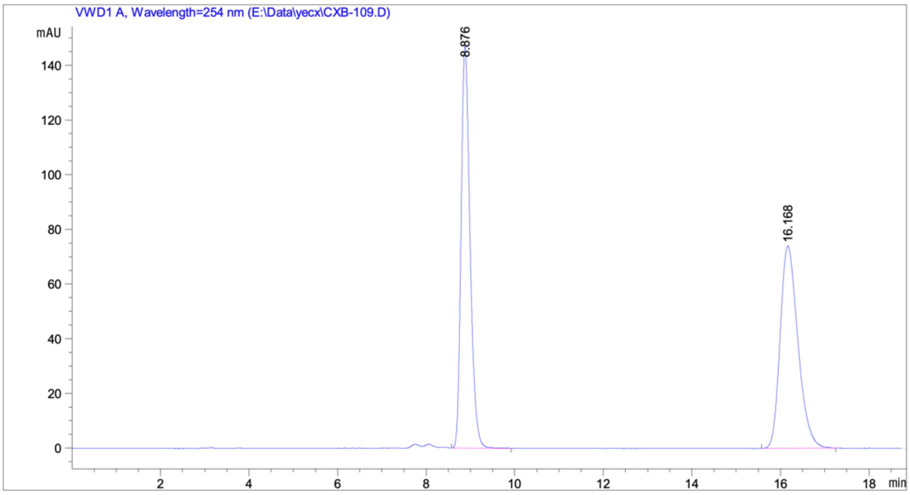

Peak #	RetTime [min]	Type	Width [min]	Area [mAU*s]	Height [mAU]	Area %
1	8.876	BV R	0.2147	2061.95850	146.90955	50.0269
2	16.168	BB	0.4246	2059.73804	74.03328	49.9731

Figure S148. Racemate of compound **3s**.

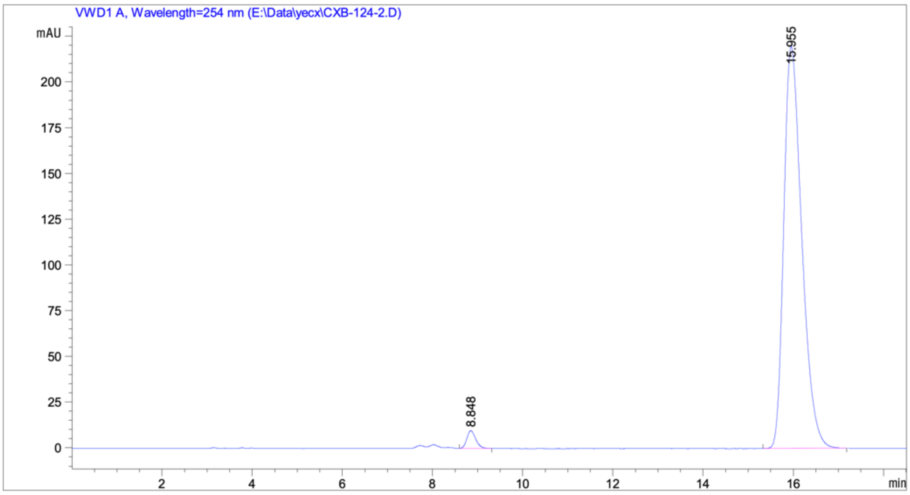

Peak #	RetTime [min]	Type	Width [min]	Area [mAU*s]	Height [mAU]	Area %
1	8.848	BB	0.2106	132.90848	9.71358	2.1339
2	15.955	BB	0.4277	6095.56494	219.69946	97.8661

Figure S149. Enantioenriched mixture of compound **3s**.

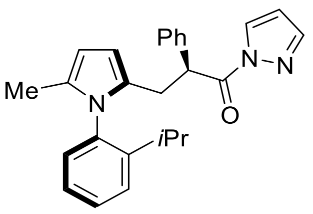

3ad

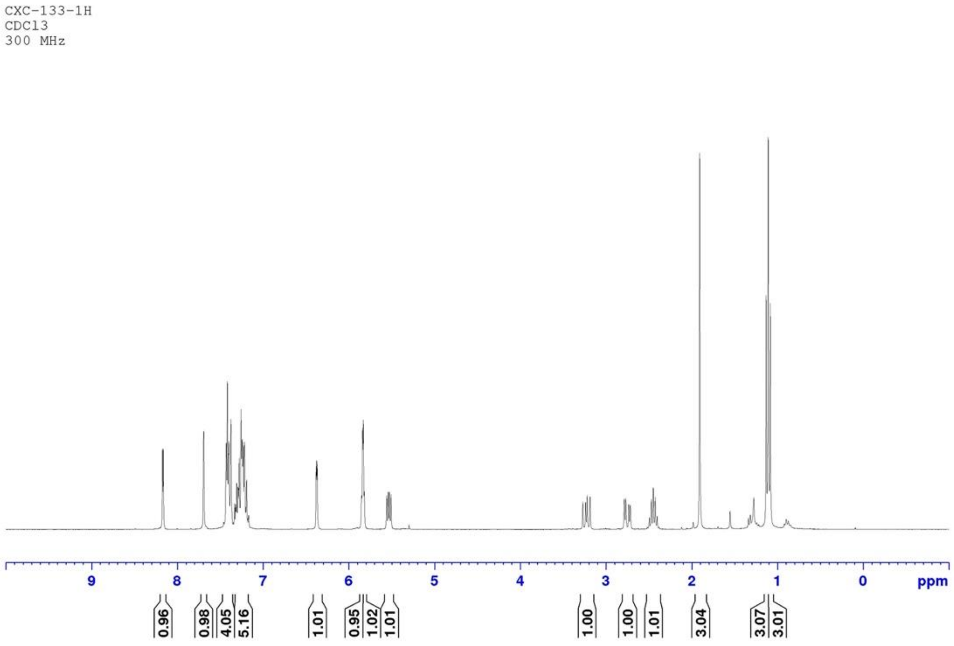

Figure S150. ^{1}H NMR ($CDCl_3$) of compound **3ad**.

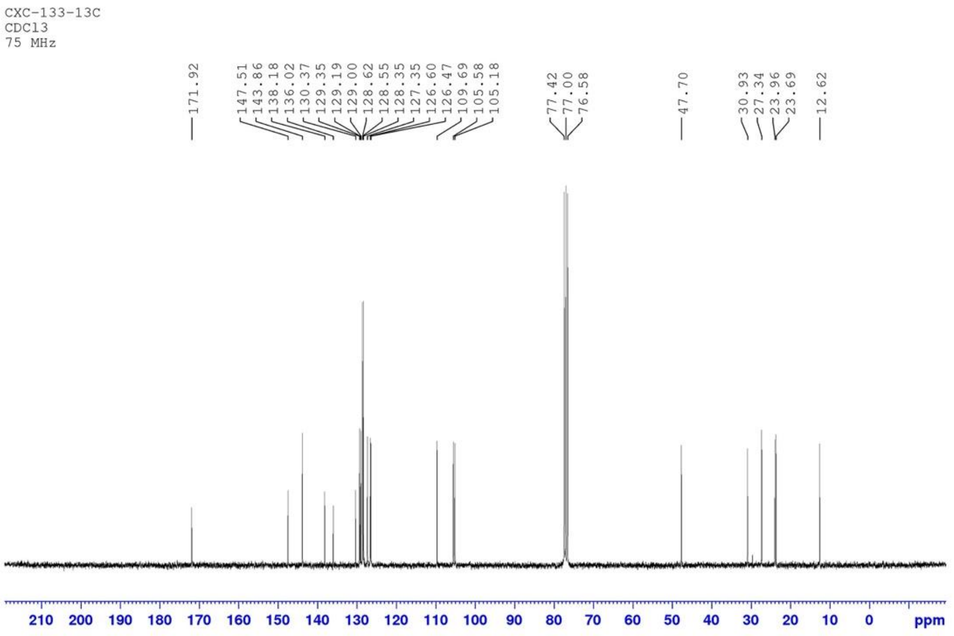

Figure S151. ^{13}C NMR ($CDCl_3$) of compound **3ad**.

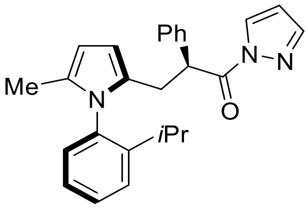

3ad

[CHIRALCEL OD-H, 25 °C, *i*PrOH/hexane = 0.6/99.4 (v/v), 1.0 mL/min, 254 nm]

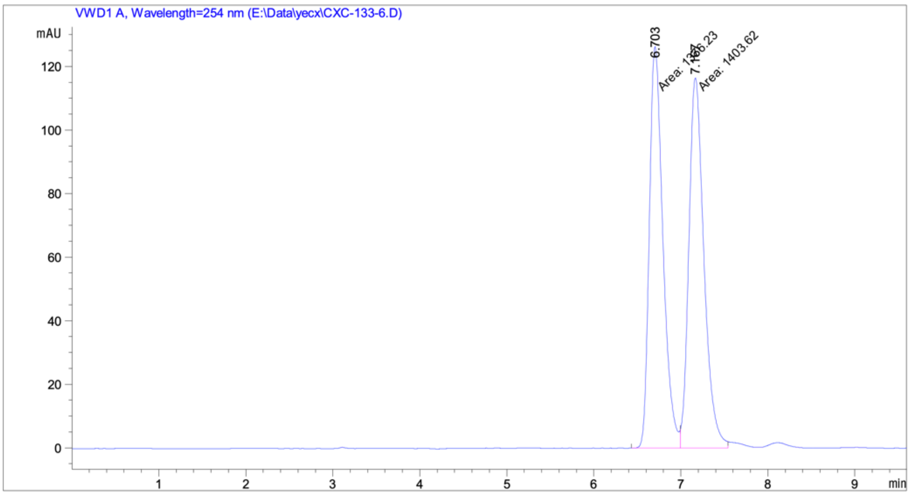

Peak #	RetTime [min]	Type	Width [min]	Area [mAU*s]	Height [mAU]	Area %
1	6.703	MF	0.1816	1376.23120	126.28593	49.5074
2	7.167	MF	0.2010	1403.61670	116.41238	50.4926

Figure S152. Racemate of compound **3ad**.

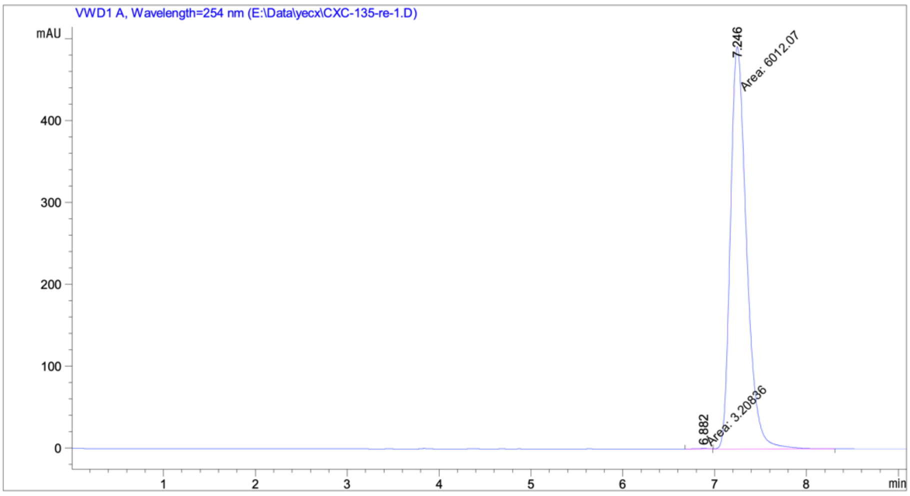

Peak #	RetTime [min]	Type	Width [min]	Area [mAU*s]	Height [mAU]	Area %
1	6.882	MM	0.1239	3.20836	4.31701e-1	0.0533
2	7.246	FM	0.2040	6012.07031	491.07440	99.9467

Figure S153. Enantioenriched mixture of compound **3ad**.

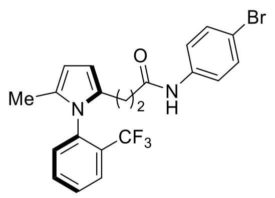

4j

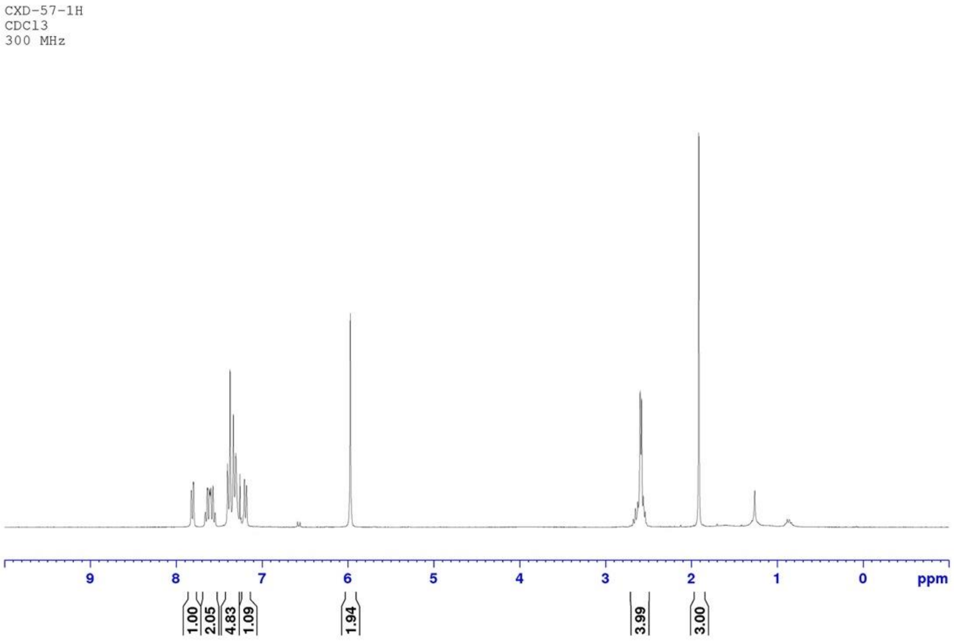

Figure S154. ^{1}H NMR ($CDCl_3$) of compound **4j**.

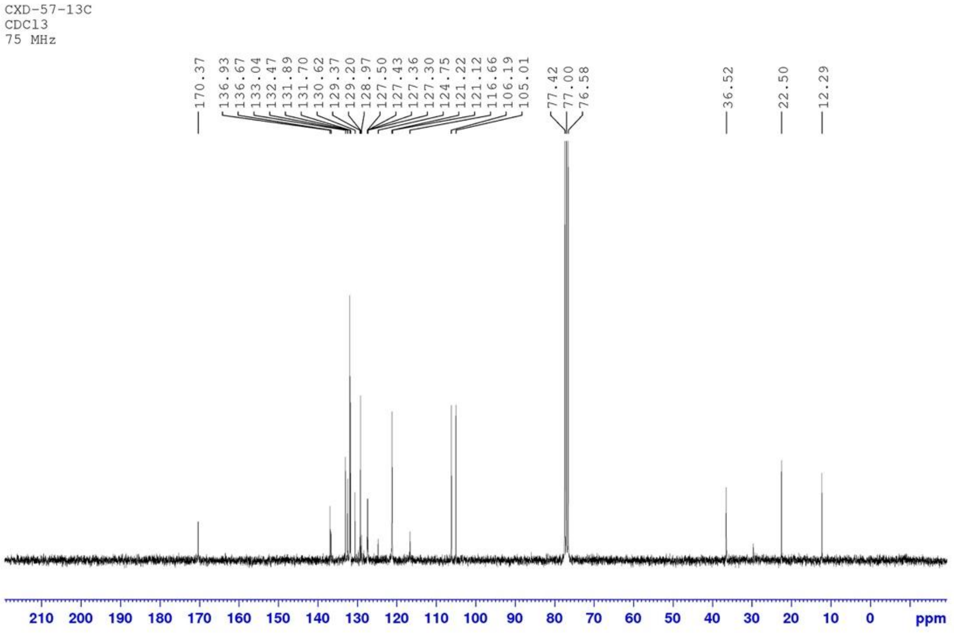

Figure S155. ^{13}C NMR ($CDCl_3$) of compound **4j**.

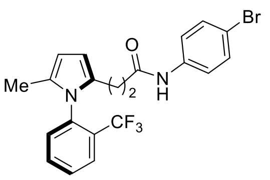

4j

[CHIRALCEL OD-H, 25 °C, *i*PrOH/hexane = 30/70 (v/v), 1.0 mL/min, 254 nm]

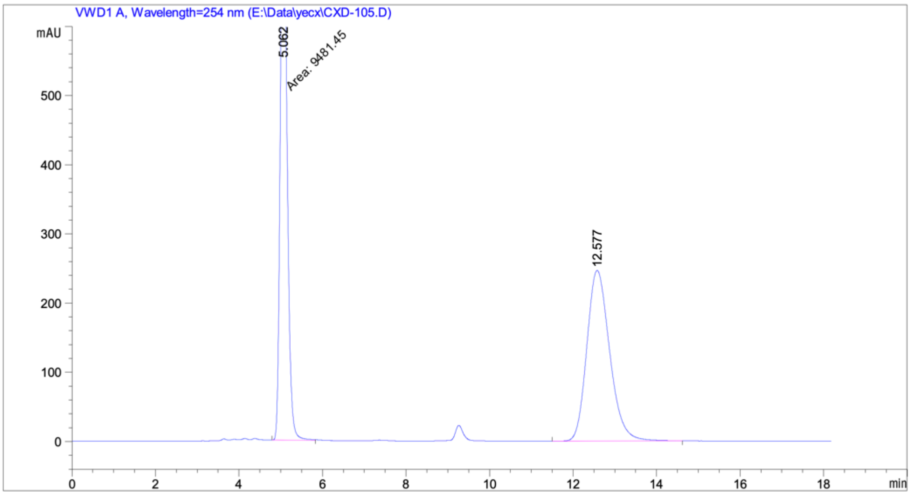

Peak #	RetTime [min]	Type	Width [min]	Area [mAU*s]	Height [mAU]	Area %
1	5.062	MF	0.1858	9481.44531	850.56744	50.3534
2	12.577	VV R	0.5873	9348.34863	246.59645	49.6466

Figure S156. Racemate of compound **4j**.

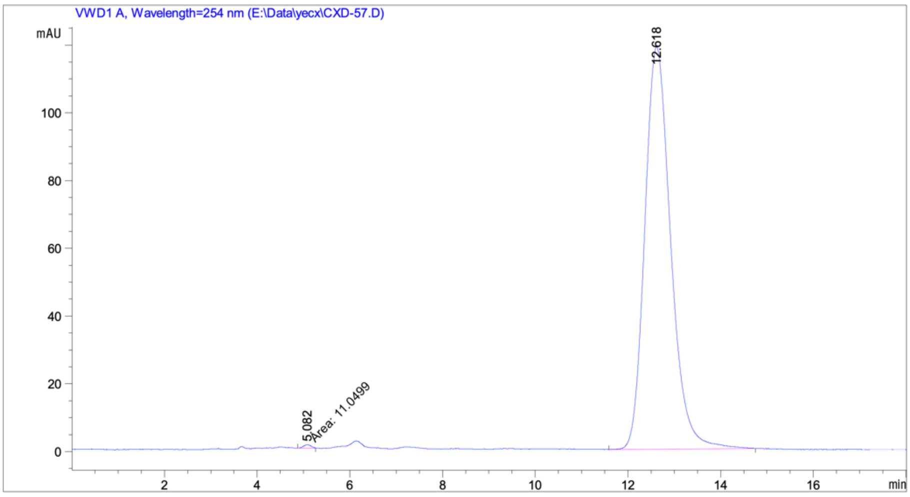

Peak #	RetTime [min]	Type	Width [min]	Area [mAU*s]	Height [mAU]	Area %
1	5.082	MM	0.1836	11.04994	1.00299	0.2388
2	12.618	BB	0.5955	4616.66797	118.75996	99.7612

Figure S157. Enantioenriched mixture of compound **4j**.

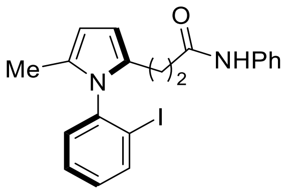

4o

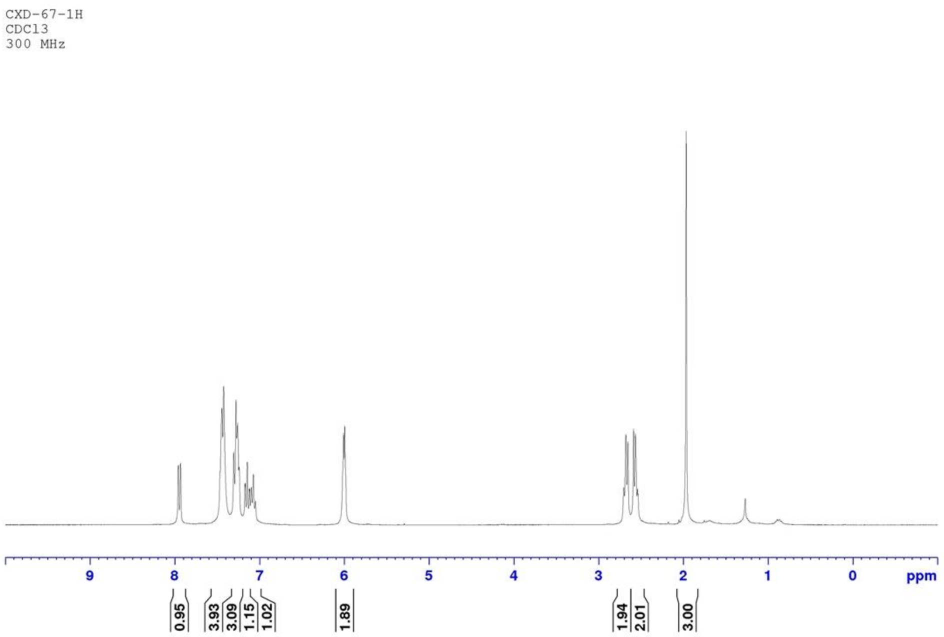

Figure S158. ^{1}H NMR ($CDCl_3$) of compound **4o**.

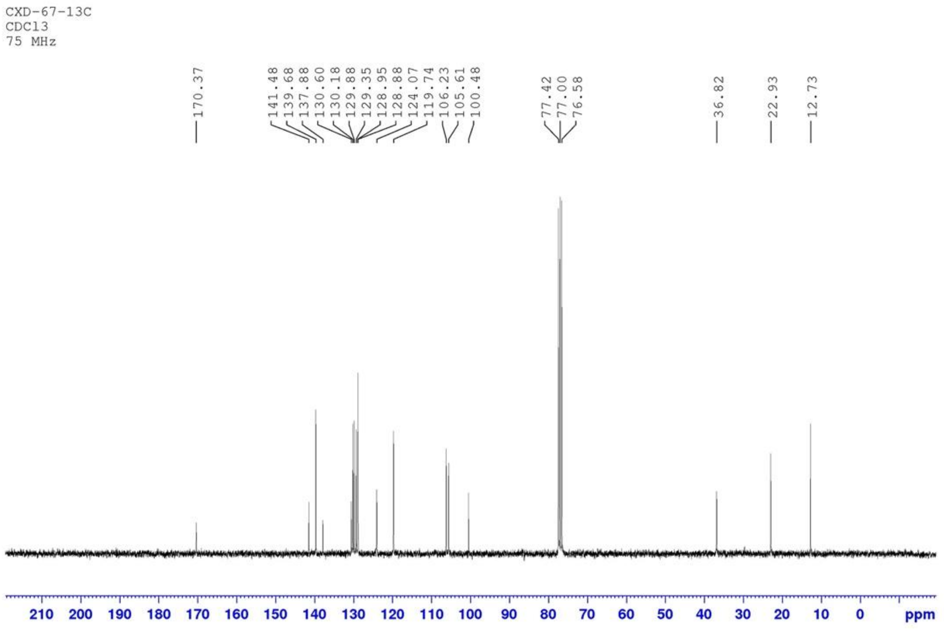

Figure S159. ^{13}C NMR ($CDCl_3$) of compound **4o**.

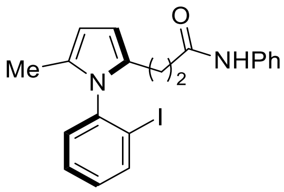

4o

[CHIRALCEL OD-H, 25 °C, *i*PrOH/hexane = 20/80 (v/v), 1.0 mL/min, 254 nm]

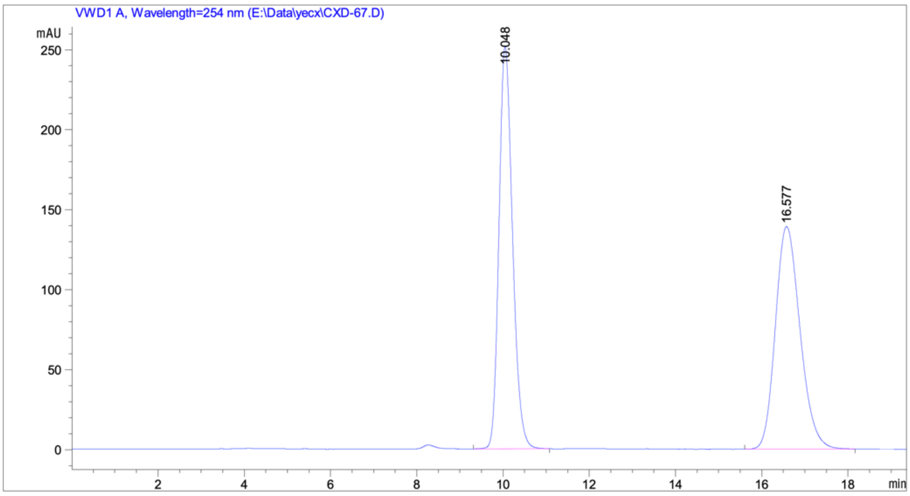

Peak #	RetTime [min]	Type	Width [min]	Area [mAU*s]	Height [mAU]	Area %
1	10.048	BV R	0.3377	5490.11768	251.44572	49.8264
2	16.577	VV R	0.6185	5528.37402	139.16653	50.1736

Figure S160. Racemate of compound **4o**.

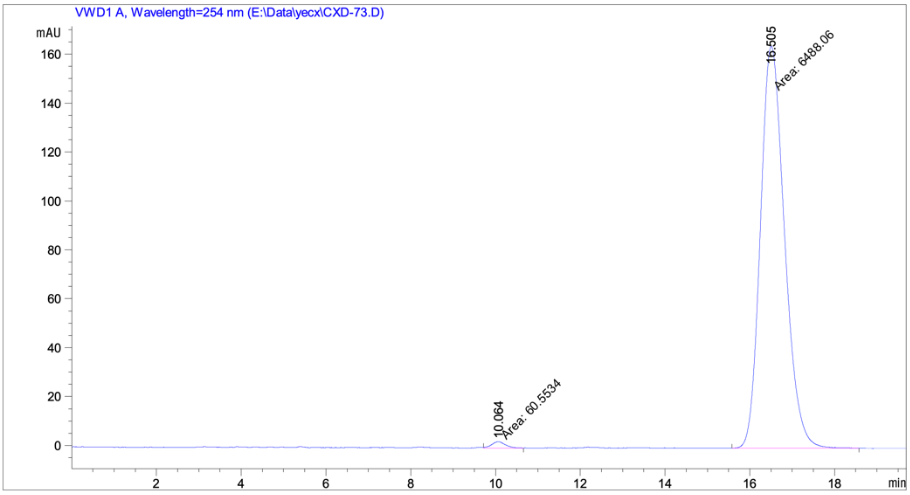

Peak #	RetTime [min]	Type	Width [min]	Area [mAU*s]	Height [mAU]	Area %
1	10.064	FM	0.3827	60.55343	2.63740	0.9247
2	16.505	MM	0.6578	6488.06445	164.39037	99.0753

Figure S161. Enantioenriched mixture of compound **4o**.

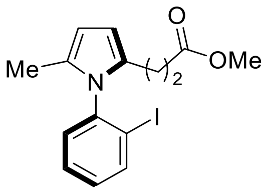

5o

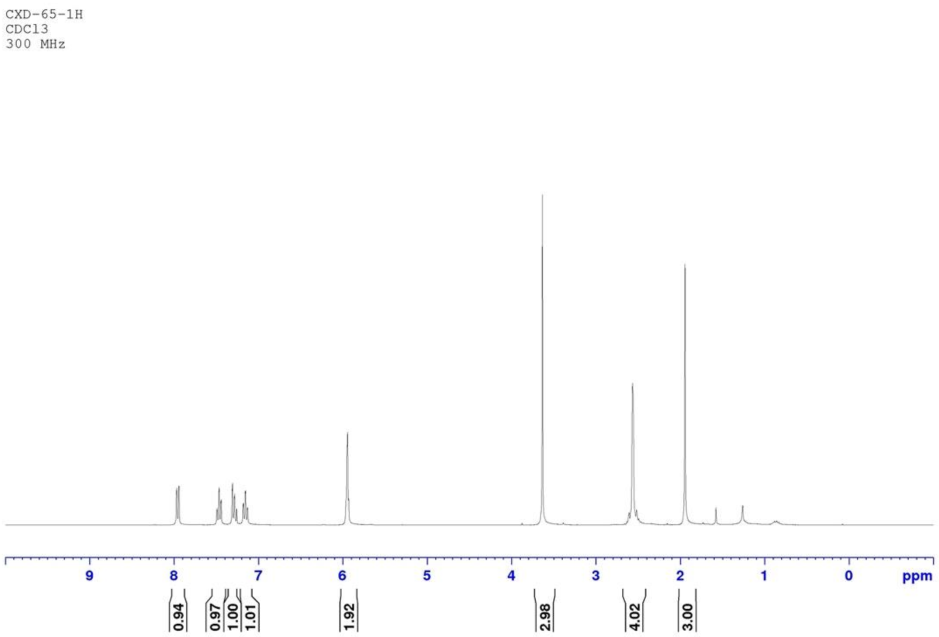

Figure S162. ^{1}H NMR ($CDCl_3$) of compound **5o**.

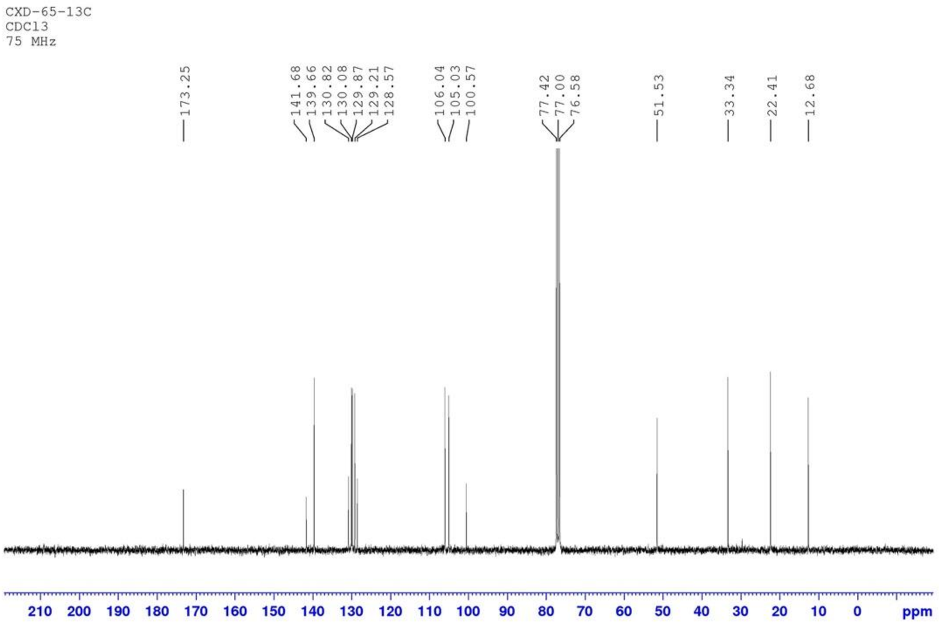

Figure S163. ^{13}C NMR ($CDCl_3$) of compound **5o**.

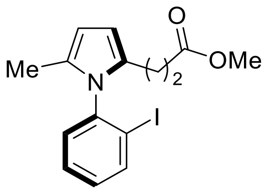

5o

[CHIRALCEL OD-H, 25 °C, *i*PrOH/hexane = 20/80 (v/v), 1.0 mL/min, 254 nm]

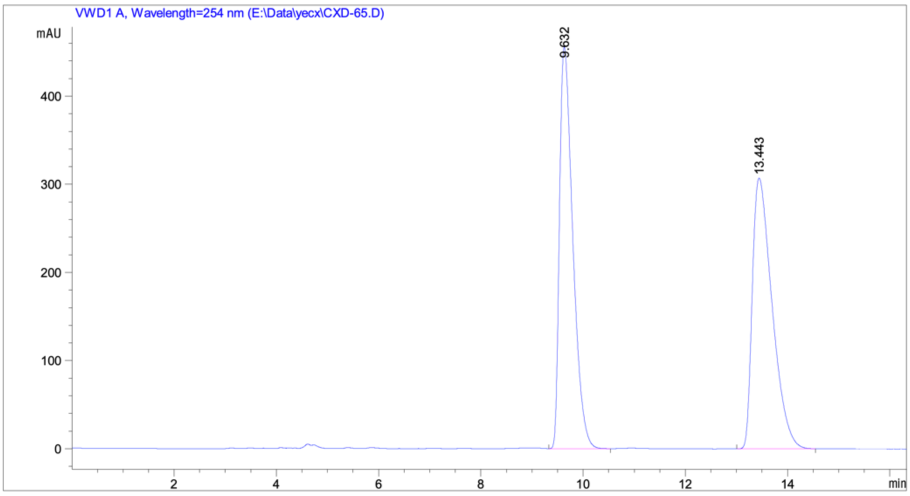

Peak #	RetTime [min]	Type	Width [min]	Area [mAU*s]	Height [mAU]	Area %
1	9.632	VB	0.2767	8234.50586	455.87482	49.9531
2	13.443	BB	0.4122	8249.97656	307.33008	50.0469

Figure S164. Racemate of compound **5o**.

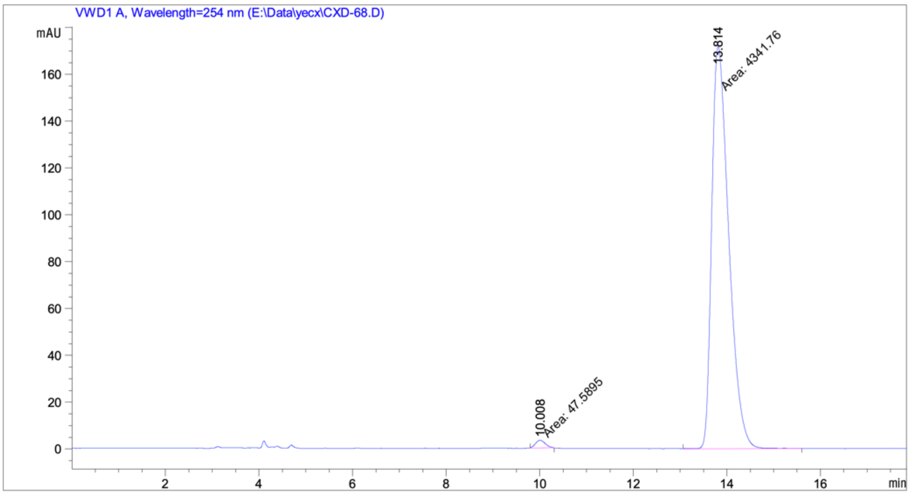

Peak #	RetTime [min]	Type	Width [min]	Area [mAU*s]	Height [mAU]	Area %
1	10.008	MM	0.2424	47.58949	3.27257	1.0842
2	13.814	MM	0.4213	4341.76367	171.74837	98.9158

Figure S165. Enantioenriched mixture of compound **5o**.

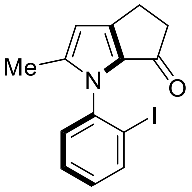

6o

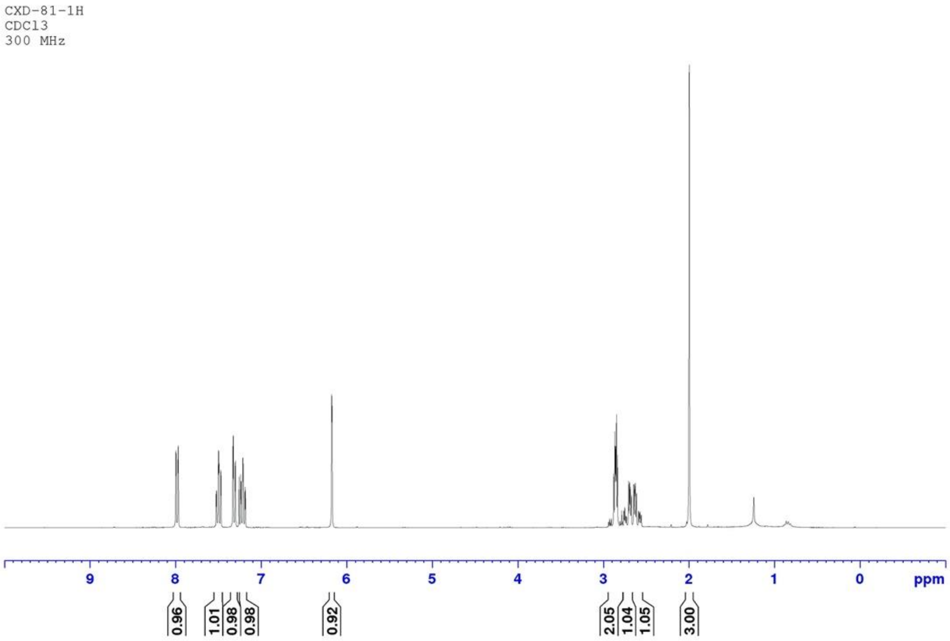

Figure S166. ^{1}H NMR ($CDCl_3$) of compound **6o**.

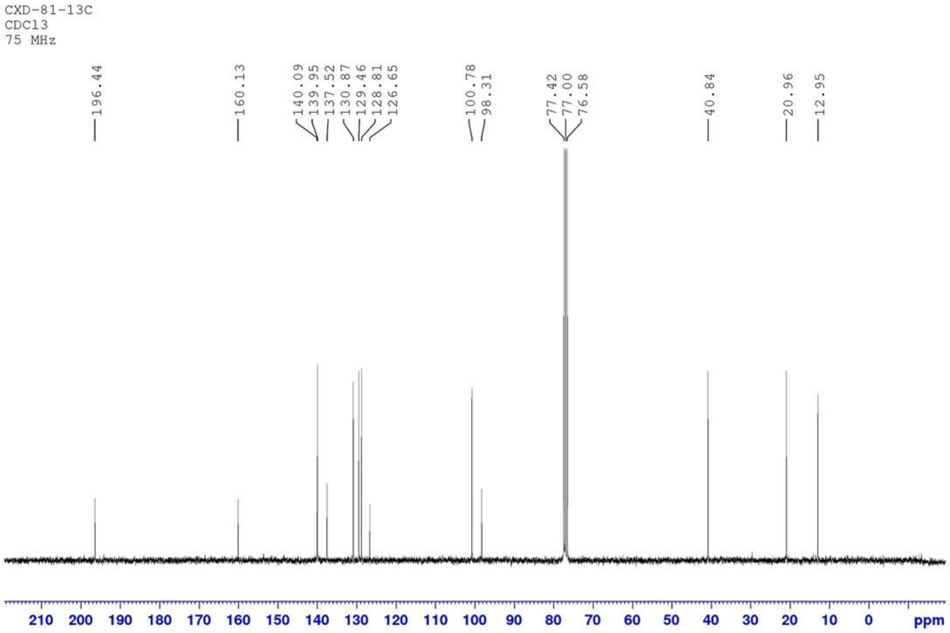

Figure S167. ^{13}C NMR ($CDCl_3$) of compound **6o**.

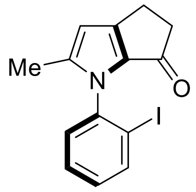

6o

[CHIRALPAK IG, 25 °C, *i*PrOH/hexane = 30/70 (v/v), 1.0 mL/min, 254 nm]

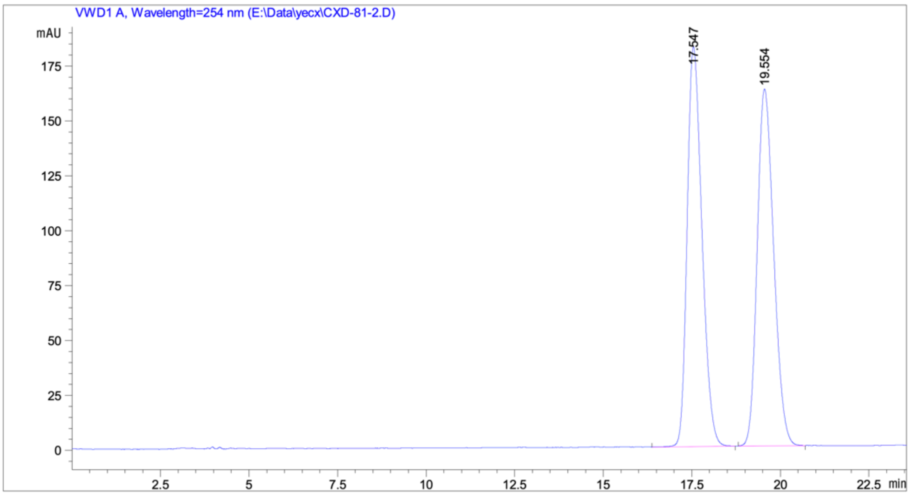

Peak #	RetTime [min]	Type	Width [min]	Area [mAU*s]	Height [mAU]	Area %
1	17.547	VB R	0.4499	5271.83594	182.04161	50.0810
2	19.554	BB	0.5053	5254.77734	162.46982	49.9190

Figure S168. Racemate of compound **6o**.

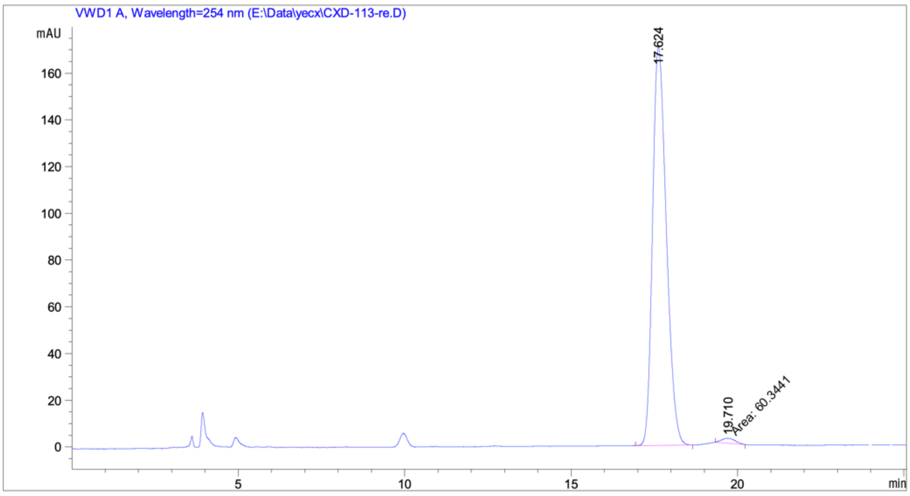

Peak #	RetTime [min]	Type	Width [min]	Area [mAU*s]	Height [mAU]	Area %
1	17.624	BV R	0.4413	4918.77002	170.61707	98.7881
2	19.710	MM	0.4689	60.34408	2.14508	1.2119

Figure S169. Enantioenriched mixture of compound **6o**.

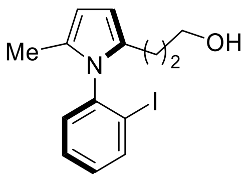

7o

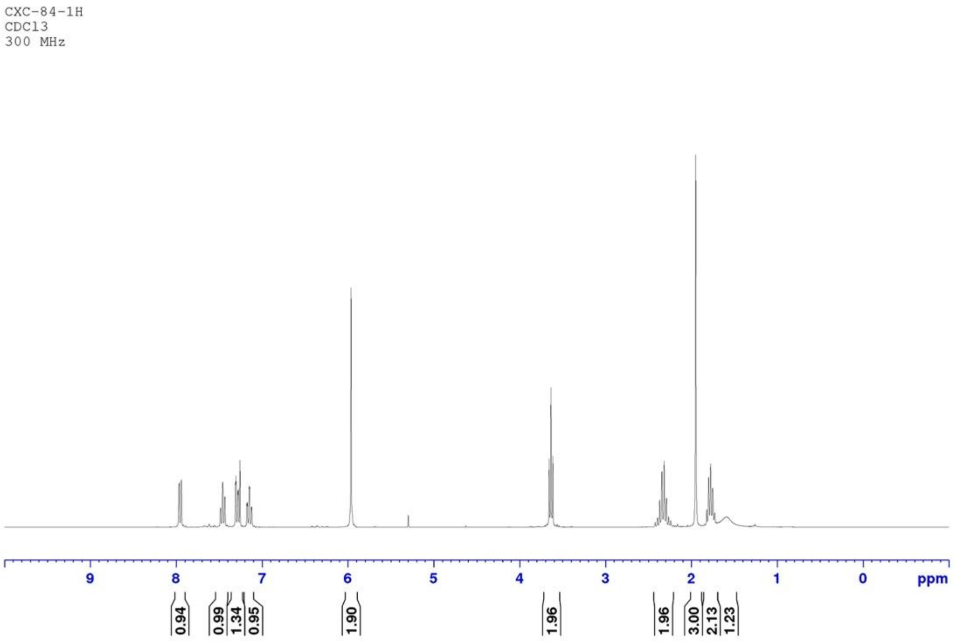

Figure S170. ^{1}H NMR ($CDCl_3$) of compound **7o**.

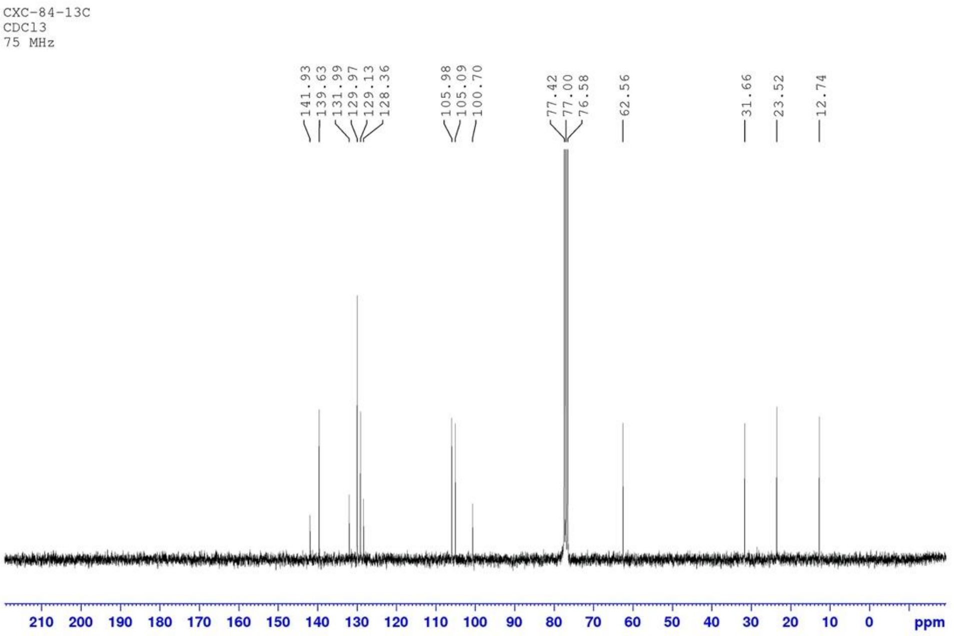

Figure S171. ^{13}C NMR ($CDCl_3$) of compound **7o**.

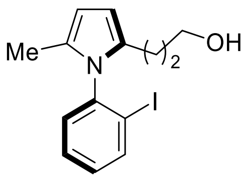

7o

[CHIRALCEL OD-H, 25 °C, *i*PrOH/hexane = 20/80 (v/v), 1.0 mL/min, 254 nm]

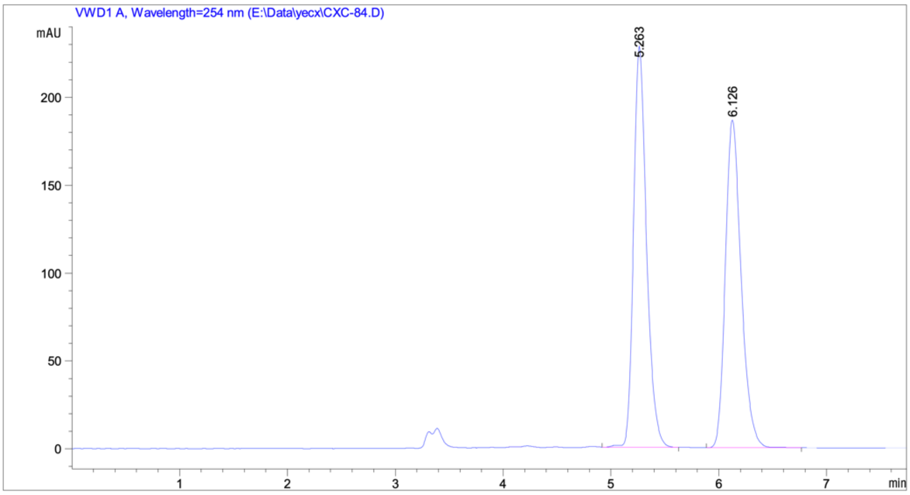

Peak #	RetTime [min]	Type	Width [min]	Area [mAU*s]	Height [mAU]	Area %
1	5.263	VB R	0.1267	1910.32495	228.08640	50.1926
2	6.126	BV R	0.1556	1895.66077	186.42726	49.8074

Figure S172. Racemate of compound **7o**.

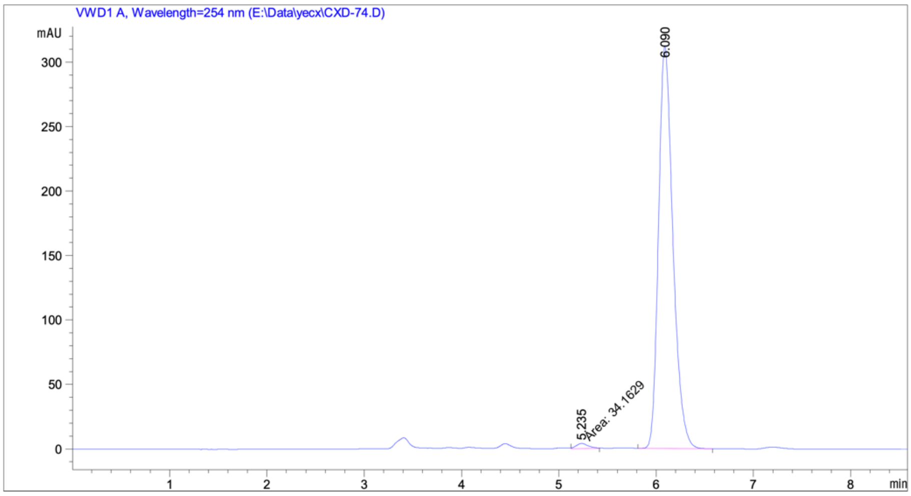

Peak #	RetTime [min]	Type	Width [min]	Area [mAU*s]	Height [mAU]	Area %
1	5.235	MF	0.1441	34.16289	3.95155	1.0551
2	6.090	BB	0.1569	3203.80688	311.65204	98.9449

Figure S173. Enantioenriched mixture of compound **7o**.

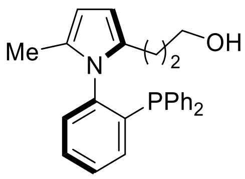

8o

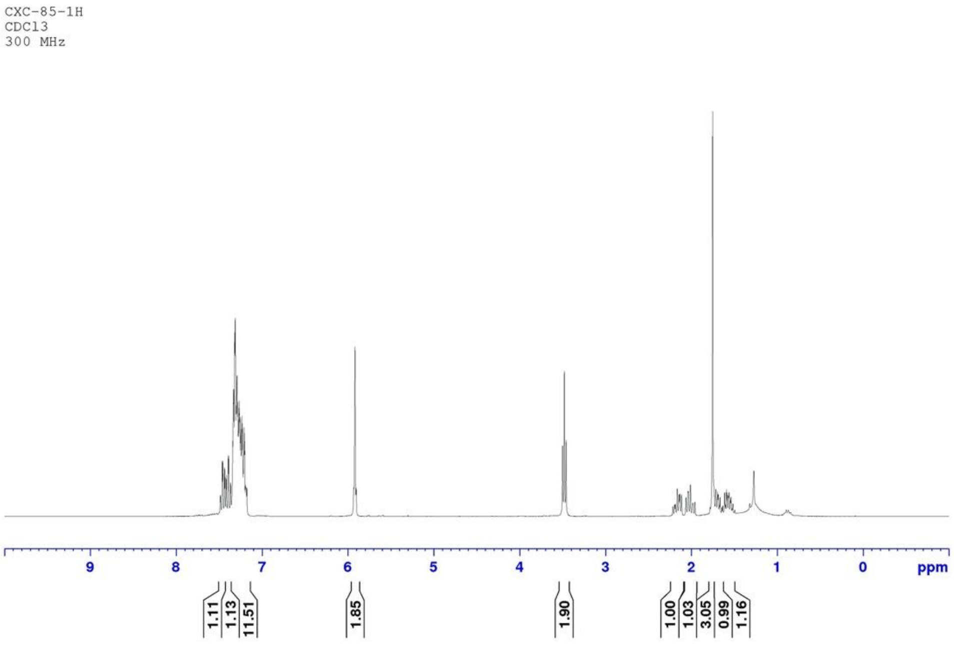

Figure S174. ^{1}H NMR ($CDCl_3$) of compound **8o**.

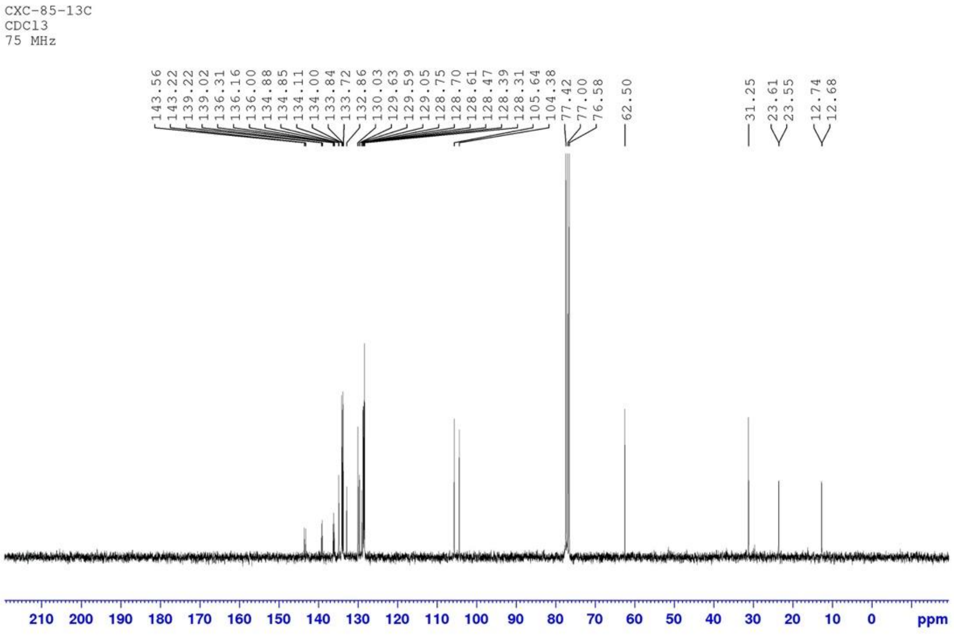

Figure S175. ^{13}C NMR ($CDCl_3$) of compound **8o**.

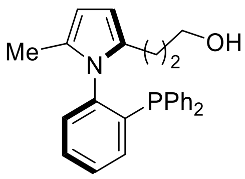

8o

[CHIRALCEL OD-H, 25 °C, *i*PrOH/hexane = 20/80 (v/v), 1.0 mL/min, 254 nm]

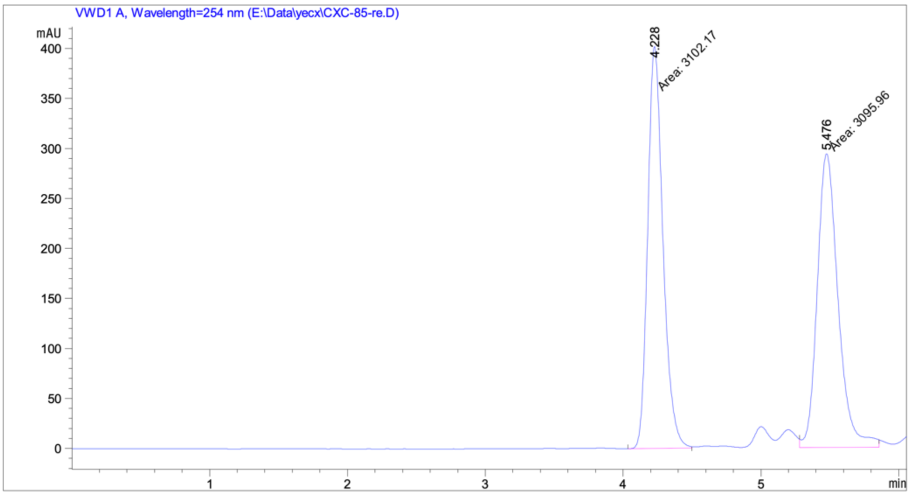

Peak #	RetTime [min]	Type	Width [min]	Area [mAU*s]	Height [mAU]	Area %
1	4.228	MM	0.1286	3102.17163	402.06274	50.0501
2	5.476	MF	0.1755	3095.96240	293.94882	49.9499

Figure S176. Racemate of compound **8o**.

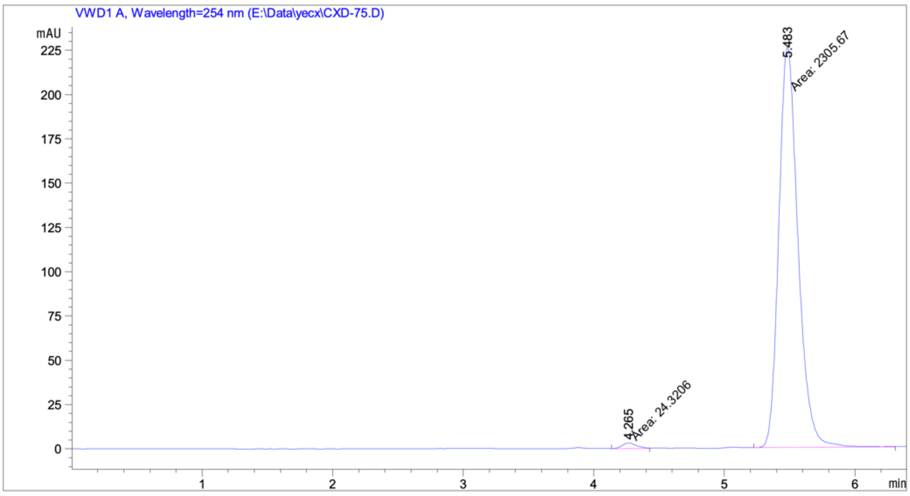

Peak #	RetTime [min]	Type	Width [min]	Area [mAU*s]	Height [mAU]	Area %
1	4.265	MF	0.1287	24.32059	3.14996	1.0438
2	5.483	MM	0.1699	2305.66968	226.18324	98.9562

Figure S177. Enantioenriched mixture of compound **8o**.

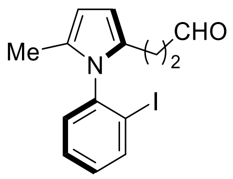

9o

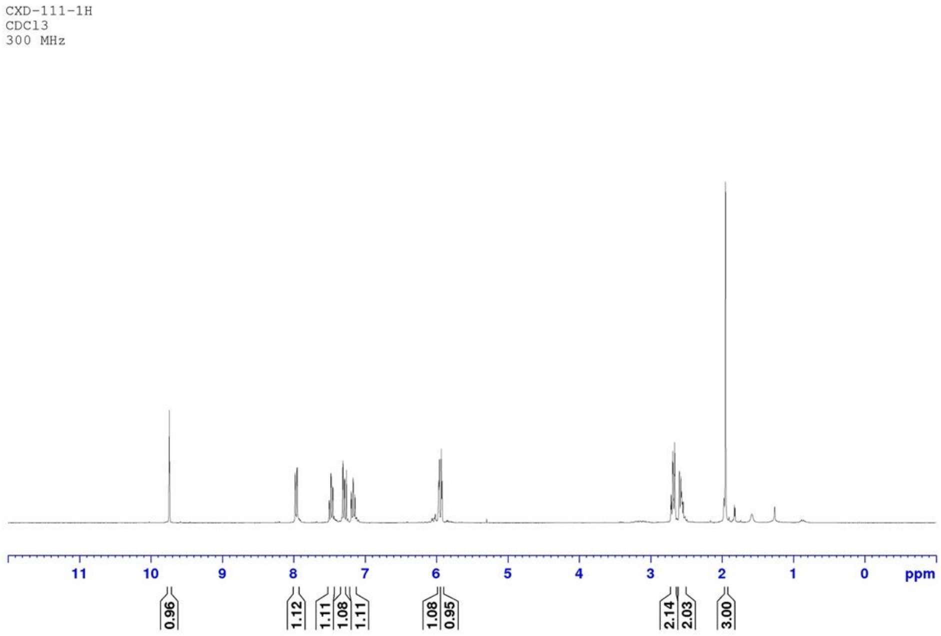

Figure S178. ^{1}H NMR ($CDCl_3$) of compound **9o**.

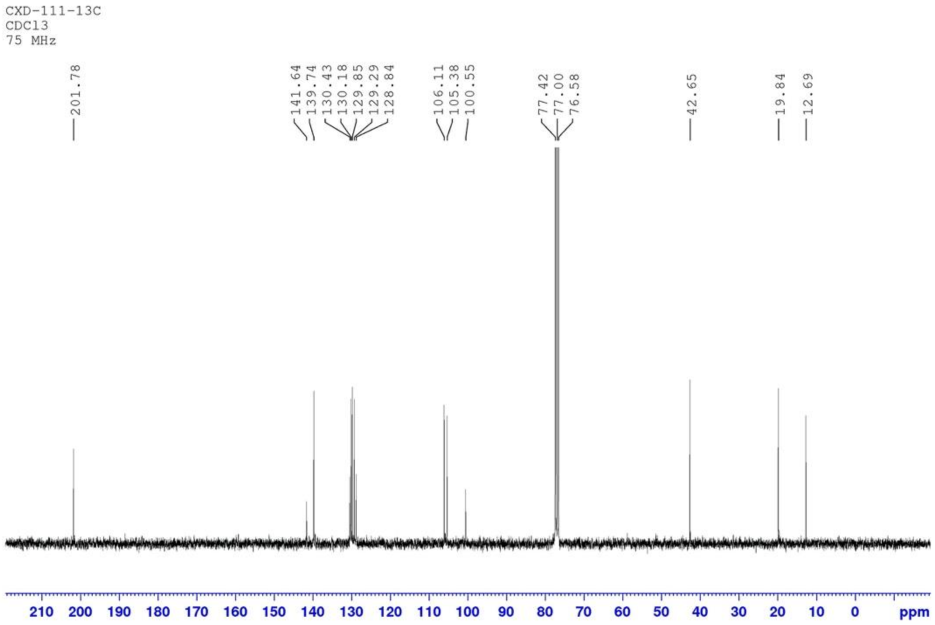

Figure S179. ^{13}C NMR ($CDCl_3$) of compound **9o**.

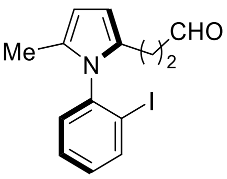

9o

[CHIRALPAK IG, 25 °C, MTBE/hexane = 25/75 (v/v), 1.0 mL/min, 254 nm]

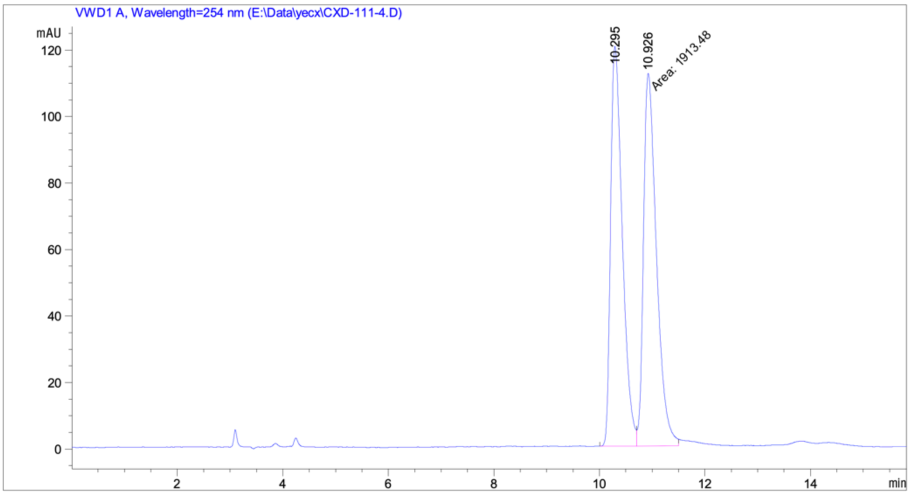

Peak #	RetTime [min]	Type	Width [min]	Area [mAU*s]	Height [mAU]	Area %
1	10.295	BV	0.2362	1856.50610	120.21661	49.2443
2	10.926	MF	0.2847	1913.48340	112.02649	50.7557

Figure S180. Racemate of compound **9o**.

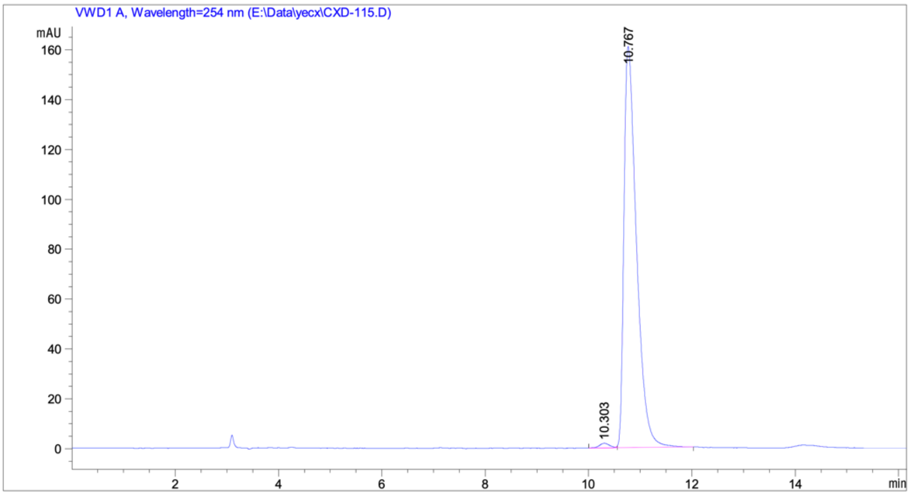

Peak #	RetTime [min]	Type	Width [min]	Area [mAU*s]	Height [mAU]	Area %
1	10.303	BV E	0.1975	26.10905	1.91030	0.9541
2	10.767	VV R	0.2555	2710.42334	160.91139	99.0459

Figure S181. Enantioenriched mixture of compound **9o**.

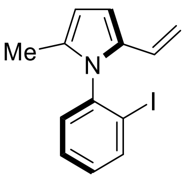

10o

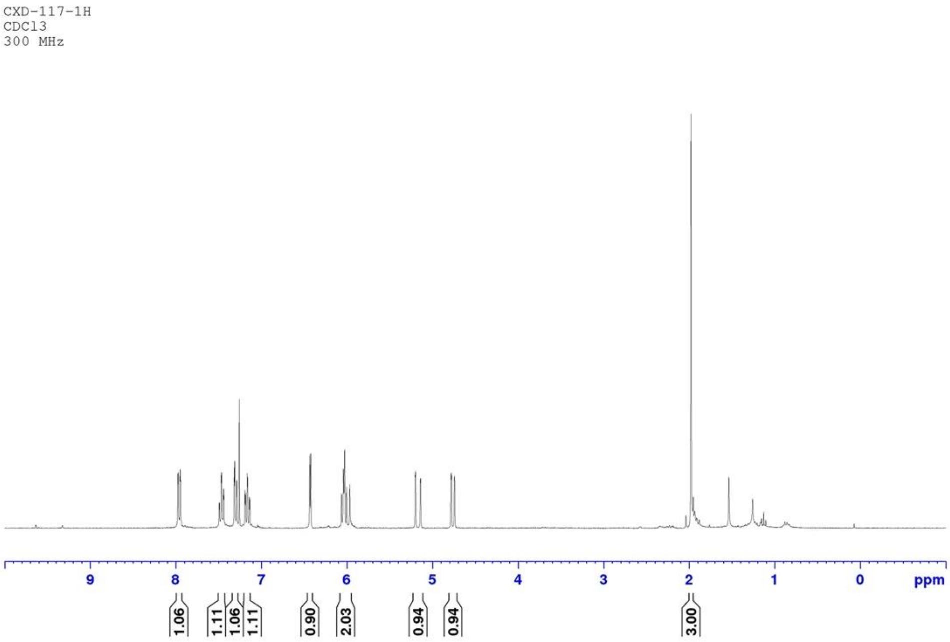

Figure S182. ^{1}H NMR ($CDCl_3$) of compound **10o**.

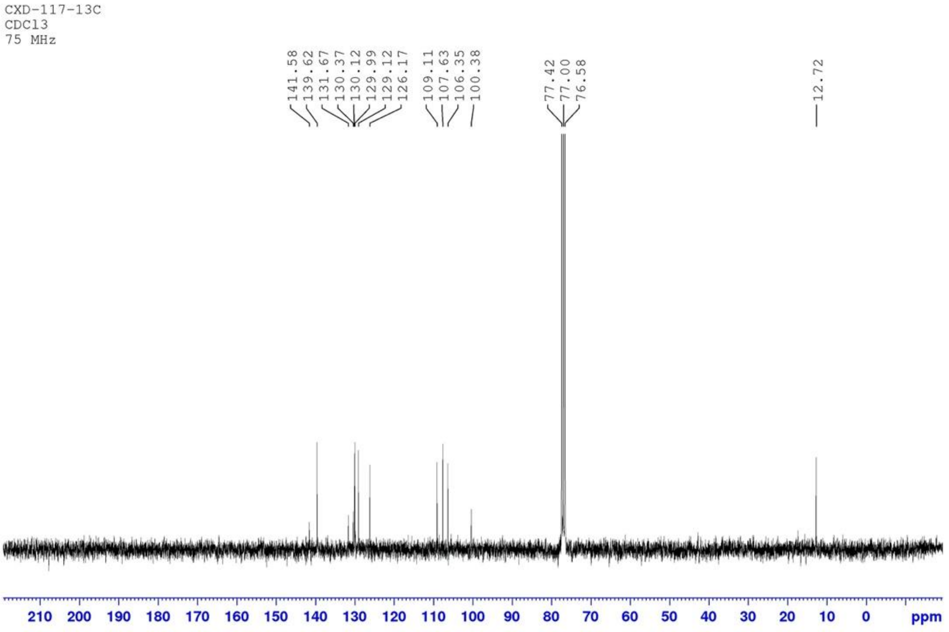

Figure S183. ^{13}C NMR ($CDCl_3$) of compound **10o**.

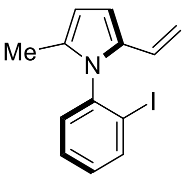

10o

[CHIRALCEL OD-H, 25 °C, *i*PrOH/hexane = 2/98 (v/v), 1.0 mL/min, 254 nm]

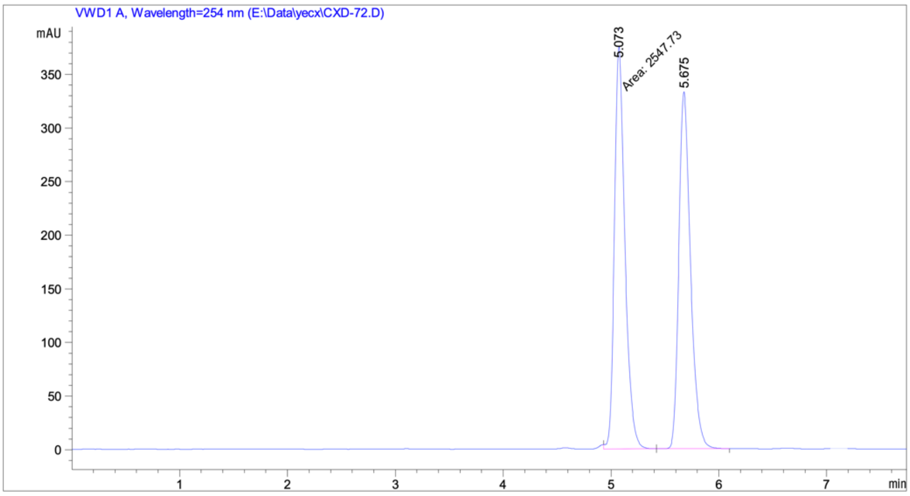

Peak #	RetTime [min]	Type	Width [min]	Area [mAU*s]	Height [mAU]	Area %
1	5.073	FM	0.1126	2547.73413	375.36588	50.3942
2	5.675	BB	0.1149	2507.87280	333.01685	49.6058

Figure S184. Racemate of compound **10o**.

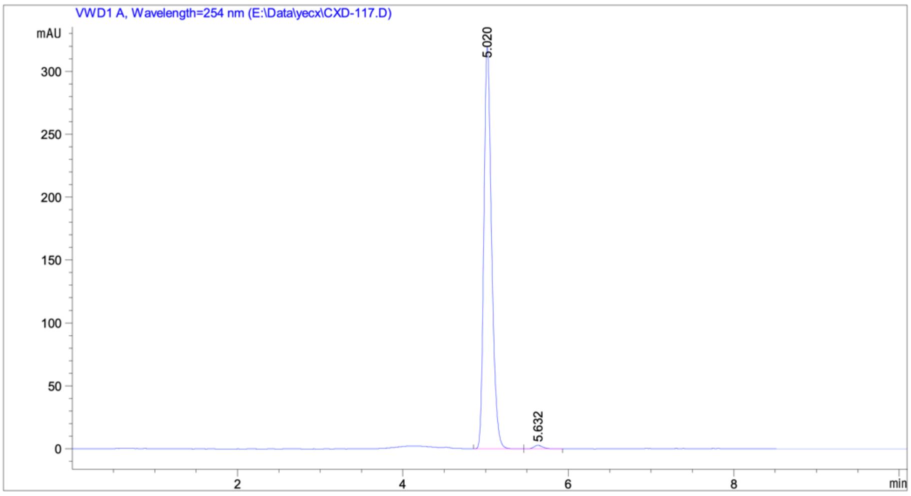

Peak #	RetTime [min]	Type	Width [min]	Area [mAU*s]	Height [mAU]	Area %
1	5.020	BV R	0.0988	2076.95825	319.64157	99.0445
2	5.632	BV R	0.1105	20.03623	2.76584	0.9555

Figure S185. Enantioenriched mixture of compound **10o**.
